# Genome Mining and Expression Analysis of Carboxylesterase and Glutathione S-Transferase Genes Involved in Insecticide Resistance in Eggplant Shoot and Fruit Borer, *Leucinodes orbonalis* (Lepidoptera: Crambidae)

**DOI:** 10.3389/fphys.2020.594845

**Published:** 2020-11-19

**Authors:** B. Kariyanna, A. Prabhuraj, R. Asokan, A. Agrawal, R. Gandhi Gracy, P. Jyoti, T. Venkatesan, M. Bheemanna, B. Kalmath, J. R. Diwan, Y. Pampanna, M. Mohan

**Affiliations:** ^1^Department of Agricultural Entomology, University of Agricultural Sciences, Raichur, India; ^2^ICAR-National Bureau of Agricultural Insect Resources, Bengaluru, India; ^3^ICAR-Indian Institute of Horticultural Research, Bengaluru, India; ^4^Department of Genetics and Breeding, University of Agricultural Sciences, Raichur, India; ^5^Department of Horticulture, University of Agricultural Sciences, Raichur, India

**Keywords:** *Leucinodes orbonalis*, insecticide resistance, genome, arboxylesterase, gene expression, glutathione S-transferase, carboxylesterase

## Abstract

The shoot and fruit borer, *Leucinodes orbonalis* (Lepidoptera: Crambidae) is the major cause of low productivity in eggplant and insecticides being the mainstay of management of *L. orbonalis*. However, field control failures are widespread due to the evolution of insecticide resistance. Taking advantage of the whole genome sequence information, the present study investigated the level of insecticide resistance and the expression pattern of individual carboxylesterase (CE) and glutathione S-transferases (GSTs) genes in various field collected populations of *L. orbonalis*. Dose-mortality bioassays revealed a very high level of resistance development against fenvalerate (48.2–160-fold), phosalone (94-534.6-fold), emamectin benzoate (7.2–55-fold), thiodicarb (9.64–22.7-fold), flubendiamide (187.4–303.0-fold), and chlorantraniliprole (1.6–8.6-fold) in field populations as compared to laboratory-reared susceptible iso-female colony (Lo-S). Over-production of detoxification enzymes *viz.*, CE and GST were evident upon enzyme assays. Mining of the draft genome of *L. orbonalis* yielded large number of genes potentially belonging to the CE and GST gene families with known history of insecticide resistance in other insects. Subsequent RT-qPCR studies on relative contribution of individual genes revealed over-expression of numerous GSTs and few CEs in field populations, indicating their possible involvement of metabolic enzymes in insecticide resistance. The genomic information will facilitate the development of novel resistance management strategies against this pest.

## Highlights

- High levels of multiple insecticide resistance in field collected *Leucinodes orbonalis* populations.
- Enhanced midgut activities of carboxylesterase and glutathione S-transferase enzymes.
- Qualitative changes in esterase banding pattern in resistant populations.
- Mining and retrieval of 94 carboxylesterase and 33 glutathione S-transferase full length gene sequences from draft assembled genome and transcriptome of *L. orbonalis.*
- Over-expression of large number of glutathione S-transferase and few carboxylesterase genes in resistant field collected populations.

## Introduction

Eggplant or aubergine (*Solanum melongena*), also called as brinjal, is one of the most popular vegetable crops in south-east Asia especially India, China, and Bangladesh. The most important limiting factor in brinjal cultivation is the damage caused by shoot and fruit borer, *Leucinodes orbonalis* Guenee (Lepidoptera: Crambidae). It was first described from India and now it is distributed all over Asia, Africa, and in few parts of Europe (Mally et al., 2015). In India and Bangladesh, it causes severe yield losses up to 93% despite best management practices (Kodandaram et al., 2017; Prodhan et al., 2018). Surveys in India and Bangladesh indicated that farmers spray chemical insecticides up to 84 times during a 6–7 month cropping season. A very high level of pesticide load at a concentration 40–450 times higher than the maximum residue limit (MRL) in marketed brinjal fruits poses major health concerns (Srinivasan, 2008; Latif et al., 2010; Kariyanna et al., 2020b). It is also considered as a pest of quarantine significance to the number of countries outside its native range. *L. orbonalis* has high reproductive potential with overlapping generations. Studies reporting high level of insecticide resistance development in *L. orbonalis* might be attributed to the long-term indiscriminate use of various insecticides on eggplant (Kodandaram et al., 2017; Shirale et al., 2017).

Resistant insects overcome the toxic effect of insecticide molecules by adopting one or more mechanisms ranging from cuticular thickening, nerve impenetration, altered production of metabolic enzymes, target site insensitivity, enhanced excretion through ABC transporters as well as by gut symbionts (Mohan et al., 2015). The enhanced detoxification of insecticides by metabolic enzymes *viz.*, carboxylesterases (CE) and glutathione S-transferases (GSTs) are commonly reported in resistant populations of many insect species and mites (Ranson et al., 2002; Enayati et al., 2005; Oakeshott et al., 2005; Perry et al., 2011; Das and Dutta, 2014; Akıner and Ekşi, 2015; Benoit et al., 2015). The resistant individuals metabolize the insecticides faster due to higher catalytic rate or enhanced production of the enzyme as a consequence of increased transcription or gene duplication (Panini et al., 2016; Kariyanna et al., 2020a). The GSTs are phase II metabolic enzymes and are distributed in most of the animals (Hayes and Pulford, 1995). Among several subclasses of GST, the enzymes which are frequently associated with the insecticide resistance are Delta and Epsilon classes (Friedman, 2011; Lumjuan et al., 2011). Similarly, enhanced metabolism of organophosphates, carbamates, and pyrethroids are frequently associated with CEs through gene amplification, upregulation, mutations by coding sequence, or a combination of all these mechanisms (Zhang et al., 2012; Cui et al., 2015).

Considering the economic and social impact of *L. orbonalis*, genetically modified eggplant expressing insecticidal *cry1Ac* gene from *Bacillus thuringiensis* has been developed (Shelton et al., 2018), but, a moratorium on its commercialization was imposed by Indian Government due to public concern. Though *Bt* eggplant is commercialized in Bangladesh way back in 2014, yet the area under cultivation is <2500 Ha (Shelton et al., 2018) due to various reasons. Baring *Bt* brinjal, the insecticides remain the sole method of controlling *L. orbonalis* in all the eggplant growing countries. With the availability of genome sequence, the present study investigated the level of insecticide and the expression pattern of CE and GST genes from field collected insecticide resistant *L. orbonalis* populations, to pinpoint the key metabolic genes involved in insecticide degradation.

## Materials and Methods

### Insect Collection and Maintenance

The field populations of *L. orbonalis* larvae were collected during 2017–2018 from intensive eggplant growing regions of India *viz.*, Raichur (16.2120° N, 77.3439° E), Dharmapuri (12.0933° N, 78.2020° E), Bhubaneshwar (20.2961° N, 85.8245° E), Pune (18.5204° N, 73.8567° E), and Varanasi (25.3176° N, 82.9739° E). All the field populations were reared under laboratory conditions at 27 ± 2°C, 60–70% relative humidity (RH), and a photoperiod of 14:10 h (L:D) on a natural diet and the F_1_ individuals were used for bioassay and biochemical studies. The insecticide susceptible iso-female line (National Accession number: NBAIR-IS-CRA-01A), designated as Lo-S (65th generation) was originally derived from *L. orbonalis* collected near Bengaluru (12.9716° N, 77.5946° E) and maintained at insect genomic resources laboratory at ICAR-NBAIR.

### Insecticide Resistance Bioassays

Insecticides fenvalarate (20% EC), emamectin benzoate (5% EC), phosalone (35%), thiodicarb (75%), flubendiamide (20%), and chlorantraniliprole (18.5%) were selected for conducting dose-mortality bioassays based on their usage history on brinjal by farmers. Filter paper residue assay (Cheng et al., 2010) with essential modifications was used for insecticide resistance bioassays on early second instar larvae of *L. orbonalis*. Five to seven appropriate concentrations of each of the selected insecticides were prepared based on the dose bracketing technique. The filter paper discs (4.5 cm diameter) were dipped in the appropriate dilutions and dried vertically under shade for 1 h. Then, the discs were placed individually in plastic containers (5 cm diameter) and 10 larvae were released in each container. Moistened filter paper discs without insecticide were used in control. After 24 h, the larvae were transferred to untreated natural diet. All the assays were replicated and repeated at least thrice on alternate days. The mortality of the larvae was assessed after 48 h. The pooled larval mortality data were subjected to probit analysis using the software POLO (LeOra, 1987) and the lethal concentration to kill 50% of the test larvae (LC_50_) was calculated for each population. Resistance ratios (RRs) were calculated with the following formula: LC_50_ of field population/LC_50_ of insecticide susceptible Lo-S population.

### Preparation of Midgut Homogenate

The starved late second instar larvae were used for the preparation of midgut homogenate. Midguts were dissected and homogenized with homogenization buffer (0.1 M sodium phosphate buffer pH 7.8 containing 1 mM each of DTT, EDTA, PTU, and PMSF). The content was centrifuged at 12,000 rpm for 20 min and the clear supernatant was used as a enzyme source for estimating the titers of carboxylesterase and GST. The total protein content of the preparations was assessed by Coomassie brilliant blue G-250 dye-binding method using bovine serum albumin (BSA) as the standard (Bradford, 1976).

### Assays on Carboxylesterase and Glutathione S-Transferase

The activity of carboxylesterase was determined using the method described by van Asperen (1962) with essential modifications and α-naphthyl acetate as a substrate. The assay mixture in a 96 well microplate (iMark, Biorad microplate reader) consisted of an appropriate amount of midgut homogenate, 0.1 M sodium phosphate buffer pH 7.8 and 800 μl of 3 mM α-naphthyl acetate containing 0.3 mM eserine. The mixture was incubated at 30°C for 30 min. Finally, 200 μl of 0.1% tetrazotized o-dianisidine (Fast blue B) in 3.5% sodium dodecyl sulfate was added and incubated for 20 min at room temperature in dark. The α-naphthol formation was measured at 590 nm and the enzyme activity was computed from α-naphthol standard curve.

Glutathione S-transferase activity was determined using 1-chloro-2,4-dinitrobenzene (CDNB) as a substrate (Kao et al., 1989). The assay mixture composed of 50 mM each of CDNB and L-glutathione reduced and 10 μl gut homogenate. The change in absorbance was measured at 340 nm for 5 min and the enzyme activity in terms of nmoles of CDNB conjugated per minute per mg protein using the molar extinction coefficient of 5.3 mM^–1^ (optimized for the path length: 0.55 cm).

### Esterase Isozyme Studies

The native polyacrylamide gel electrophoresis (PAGE) with 10% resolving gel was performed as per Davis (1964) to visualize the esterase isozymes from the 2nd instar midgut larval homogenates of the *L. orbonalis* populations tested. Midgut homogenates with 20 μg protein were loaded onto the native PAGE and run at a constant voltage of 70 for 1.5 h. Gel was briefly stained with freshly prepared 0.05% (w/v) α-naphthyl acetate and 0.1% (w/v) fast blue B in 50 mM phosphate buffer pH 7.8.

### Genome Mining and Phylogenetic Analysis

The draft assembled genome (NCBI Bioproject ID: PRJNA377400) of *L. orbonalis* was used as query for extracting the gene sequences of glutathione S-transferases (GSTs) and carboxylesterases (CEs). The hmm model for carboxylesterase and GST (CE-PF00135, PF02230, and GST-C PF00043, GST-N PF02798, GST-PF13417, PF14497, PF17171, PF17172) were obtained from PFAM database^1^. A total of 94 and 33 putative CE and GST sequences were mined using hmm search with HMMER3 software package^2^. Among them, the genes with known history of involvement in insecticide resistance in other insects were selected and further confirmed with NCBI BLASTX. The GST and CE protein sequences of *Drosophila melanogaster* was downloaded from its genome database (Marygold et al., 2013), GenBank^3^ and Uniprot^4^. We combined these putative GST protein sequences with *D. melanogaster*’s annotated protein sequences for each of the six subclasses of GST (Epsilon, Sigma, Omega, Delta, Zeta, and Theta). The sequences were multiple aligned using CLUSTALW and a maximum likelihood tree with 1000 bootstrap value was constructed using MEGA7^5^ and TreeDyn online server^6^ was used to visualize the tree. Similarly, 94 CE protein sequences under seven subclasses (α-esterases, gliotactin, glutactin, neurolignin, juvenile hormone esterases, acetylcholinesterases, and cricklet) were combined, multiple aligned using MAFFT online server using conserved domain feature and phylogenetic tree of CE genes from *L. orbonalis* using *D. melanogaster* as a reference were constructed with the help of CLC workbench (QIAGEN Bioinformatics^7^) using maximum likelihood approach.

### Quantitative Real-Time PCR

The late 2nd instar larvae were used for RNA extraction (ISOLATE II RNA mini kit) by following the manufacturer’s guidelines (Bioline). Purity and concentration of total RNA were measured in a spectrophotometer (NanoDrop Lite, Thermo Fisher Scientific) and denatured agarose gel (Masek et al., 2005). RNA samples with an A260/A280 ratio ranging from 1.8 to 2.0 and A260/A230 ratio >2.0 were used for cDNA preparation. First strand of complementary DNA was synthesized from 4 μg of total RNA using Revert AID first strand cDNA synthesis kit (Thermo Fisher Scientific^TM^, Lithuania) following the suppliers guidelines and stored at −80°C till further use.

qPCR primers for 25 GST and 16 CE gene sequences were designed using Primer 3.0 software. The detailed information of the primers used in the current study is listed in Tables 1, 2. To remove the primer dimer and confirm the efficiency, OligoEvaluator^TM^ sequence analysis tool (accessed on July 2018^8^) was used. Primer specificity analysis was observed by single peak in the melting curve and single band from the agarose gel of the all candidate genes of CE and GST ranged from 90.36 to 104.58% (Tables 1, 2). Quantitative real-time PCR (RT-qPCR) amplifications were performed in 20 μL reaction consisted of 10 μL 2×SYBR^®^ Premix EX TaqTM II (Tli RNaseH Plus, TAKARA^®^, Japan), 1 μl cDNA and gene specific primer pair. The parameters used for RT-qPCR are: One cycle of 95°C for 3 min; 35 cycles of 95°C for 30 s, 53°C for 45 s, and 72°C for 1 min; a final cycle of 72°C for 10 min using Roche 480II machine. All the samples including control (no template) and internal control were performed in triplicates. The PCR products were electrophoresed on 2.0% agarose gel in a 1.0×TAE buffer. Relative expression levels for the CE and GST genes were calculated by 2^–ΔΔ*CT*^ method. The 28SR3 (28S ribosomal protein S3 mitochondrial) (Kariyanna et al., 2019) gene was used as an internal control to normalize the expression of target genes.

**TABLE 1:** Primer details for carboxylesterase (CE) genes.

**Sl. No**	**Sequence**	**Primer sequence 5′→3′**		**Length**	**GC (%)**	**Tm (°C)**	**Sequence length**	**Product size**	**E (%)**
1.	Contig8459-0.14	F	GTGTCAAACGTGTTCAGAATC	21	43	54	2796	100	91.43
		R	TACCCAGGAAAGTAGTGTAGT	21	43	54			
2.	Contig12636-0.8	F	CTTGCACTCTGTTCACTAACT	21	43	54	2712	95	88.17
		R	TCTGTCGTCGTTGTTGTATTC	21	43	54			
3.	Contig11486-0.8	F	TCACATGAGACTGGGTAGAA	20	48	55	3936	95	103.22
		R	CTCTGTAGTGTCTGTCGTAGA	21	45	54			
4.	Contig4177-0.23	F	AACCTGTTCTCACAAGCTATC	21	48	55	1146	95	98.05
		R	AGTCTAGTACCTCTCAGGATTG	22	45	55			
5.	Contig5480-0.2	F	TCCCTCACACTAACGAAAGA	20	45	55	6498	95	98.12
		R	TCCAGGTATTCAGGAGTGATAG	22	45	55			
6.	Contig11205-0.1	F	AACGTGGAACCTTACTTCAC	20	45	54	597	100	97.60
		R	CACCTTCGTCTTCGTTGTAA	20	45	54			
7.	Contig4653-0.41	F	TGCAAGTGACTGTGAACGAAG	21	48	58	2417	179	101.88
		R	AGCGTTACCAGGGATGTCTTT	21	48	58			
8.	Contig132-0.69	F	ATCCCTCTGGAACCTCAAAAA	21	43	54	3375	179	88.81
		R	GGGAACATTCTGAAAGGGAAG	21	48	54			
9.	Contig3761-0.1	F	TGTGGGCTAACTTCGCTAAAA	21	43	55	681	233	90.70
		R	TTCGATTTTTCTCACCACACC	21	43	56			
10.	Contig3761-0.2	F	CCTGGCTCTGAAACTGACTTG	21	52	55	1251	150	99.31
		R	TTGATAGGAGGAGCAGCGTAA	21	43	55			
11.	Contig9545-0.25	F	CAAAGAACTTCAACTTCACT	20	35	49	555	193	101.67
		R	TTTTTCGTCAGGTTTGTGAGG	21	43	55			
12.	Contig9545-0.26	F	TCCTTCATTCCTGCAAAGAGA	21	43	55	804	167	100.15
		R	AGTAGTCACCCACCACGTCAG	21	57	60			
13.	Contig11588-0.37	F	CTGGTAACGCTGGTATCAAA	20	45	54	1269	111	97.11
		R	CAGCTGATTGACCGAAGATAG	21	48	55			
14.	Contig5480-0.52	F	ACCAATCAGACGACTCACACC	21	52	59	1863	157	90.48
		R	GCGATGTTCACCAGTTTTGAT	21	43	55			
15.	Contig5480-0.3	F	GGTCCTGTGAAAGGTTACAA	20	45	55	663	94	92.15
		R	TGTATTTGTCAGCACCAGTAG	21	43	55			
16.	Contig11205-0.4	F	GAAGGTGAAATCGTGAACAAC	21	43	54	225	165	97.11
		R	TTGGTATGATGATGAACCGTGT	22	41	56			

**TABLE 2:** Primer details for glutathione S-transferase (GST) genes.

**Sl. No**	**Sequence**	**Primer sequence 5′→3′**		**Length**	**GC%**	**Tm (°C)**	**Sequence length**	**Product size**	**E (%)**
1.	Contig7206-0.56	F	GCCTCAAGACTGCATGAAAAG	21	48	56	600	223	98.59
		R	GTCAGCCAGAGTGATTTCGTC	21	52	58			
2.	Contig2323-0.22	F	TTCACCTCCTTGCAGATCAGT	21	48	58	375	160	87.80
		R	GAAGTCACCGTCTTTCAGCAG	21	52	58			
3.	Contig8855-0.81	F	ACGACATCCTGAACACTCTGC	21	52	59	657	165	98.93
		R	TTCTCCATTGTCTCACCCAAG	21	48	57			
4.	Contig2841-0.35	F	CGCTATGAGATTCTGCCCTTAC	22	50	57	762	120	104.58
		R	GAGAAGTCGAACAGCCATTCA	21	48	57			
5.	Contig2596-0	F	TGCTACCTGGTGGACAAATTC	21	48	57	639	236	92.92
		R	CCCATTTAGTGTCGTTCAGGA	21	48	56			
6.	Contig14202-0.24	F	TATCCTGGCTCTGAACGCTAA	21	48	58	717	171	105.55
		R	TCGTCCAGGTATTCCACAGTC	21	52	59			
7.	Contig1970-0.34	F	GACGCTCTGAGACTGTTCGAC	21	57	60	564	190	96.96
		R	CTTCGTAACCAGGAGCAGTTG	21	52	58			
8.	Contig1510-0.48	F	ATCGGTTGGCTGAACACTATG	21	48	57	726	227	95.00
		R	AAAGTAGCCAGCATTTGAGCA	21	43	57			
9.	Contig2478-0.25	F	TCTGGGACTCACACGCTATCT	21	52	56	654	208	100.59
		R	ATTTGAGCCAGGTTTTCAGGT	21	43	60			
10.	Contig3755-0.39	F	CGCTATCATGCAATACGTGTG	21	48	56	654	183	102.61
		R	AGACCCAGAGGAGTTCTTTCG	21	52	59			
11.	Contig3537-0.3	F	ACAAATTCAACCTGCTGCCTA	21	43	56	423	227	96.58
		R	CAGTTTACCAGCGAAGTGACC	21	52	58			
12.	Contig10769-0.3	F	TCACTGGCTATCGCTAGATACA	22	45	57	621	116	98.89
		R	CGTTTGACCAGAAGTCGAAGAT	22	45	57			
13.	Contig1010-1.18	F	GACGCTCTGAGACTGTTCGAC	21	57	60	648	190	98.00
		R	CTTCGTAACCAGGAGCAGTTG	21	52	58			
14.	Contig9166-0.22	F	ACAAATCAGTGGCTGCTAAA	20	40	54	345	97	95.31
		R	GGCAGGTAGTACAGTTTGATAG	22	45	54			
15.	Contig6423-0.33	F	TGCTACCTGGTGGACAAATTC	21	48	57	645	236	89.22
		R	CCCATTTAGTGTCGTTCAGGA	21	48	56			
16.	Contig199-0.91	F	TGTGAAAGCTCTGGGTGAATC	21	48	57	567	233	99.14
		R	GAAGTCCACGTTCATGTCGAT	21	48	57			
17.	Contig8048-0.1	F	TCAATCGAAGAAGACCTGGAA	21	43	55	486	150	98.83
		R	CAGCAGTTGAGGGTACACGTT	21	52	60			
18.	Contig11878-0.11	F	TGCTACCTGGTGGACAAATTC	21	48	57	540	236	98.55
		R	CCCATTTAGTGTCGTTCAGGA	21	48	56			
19.	Contig7006-0	F	CGCTAGATACCTGGCTAACAAA	22	45	56	606	98	92.55
		R	CCCAGAAGTCGTAGATGTTCAG	22	50	57			
20.	Contig3023-0.16	F	GCTGCTTACGAAGGTAGAATG	21	48	55	618	173	99.14
		R	GCAGGGAAGTGTTCTTTAGGG	21	52	58			
21.	Contig2596-0	F	TGCTACCTGGTGGACAAATTC	21	48	57	639	235	101.31
		R	CCCATTTAGTGTCGTTCAGGA	21	48	56			
22.	Contig2478-0.15	F	CATCACACGCTATCACTACTT	21	43	54	456	102	97.25
		R	GCAGTCTTTGGTCGATTCT	19	47	54			
23.	Contig3291-0.7	F	TATCGTGTCAGGTATCCAAC	20	45	53	402	179	92.16
		R	GTCAGCCAGAGTCAGTTGGTC	21	57	60			
24.	Contig4841-1.1	F	TGGACGACACTAGACCTAAA	20	45	54	327	81	93.78
		R	GTTGGATACCTGACACGATAG	21	48	54			
25.	Contig2323-0.23	F	CTCACACGCTATCACTACTTAC	22	45	54	645	101	95.56
		R	GCAGTCTTTGGTCGATTCT	19	47	54			

## Results

### Insecticide Resistance Monitoring

The LC_50_ values against fenvalerate, phosalone, thiodicarb, emamectin benzoate, flubendiamide and chlorantraniliprole indicated that there is a large shift in susceptibility of field-populations of *L. orbonalis* as compared to the laboratory reared susceptible iso-female colony (Lo-S). The field collected populations exhibited 3.6–160-fold resistance against fenvalerate, 7.3–534.6-fold resistance against phosalone, 7.0–55.0-fold resistance against emamectin benzoate, 2.0–22.7-fold resistance against thiodicarb, 29.5–303.0-fold resistance against flubendiamide, and 1.6–8.6-fold resistance against chlorantraniliprole. However, the susceptibility level of all the field populations and the Lo-S did not vary significantly against chlorantraniliprole based on the overlapping fiducial limit values. High level of resistance development against phosalone and flubendiamide was observed in *L. orbonalis* collected from Varanasi whereas other field populations, the RRs were non-significant as compared to Lo-S. *L. orbonalis* populations from Bhubaneswar showed a significantly very high levels of resistance against fenvalerate, phosalone and flubendiamide as compared to Lo-S. The population of *L. orbonalis* collected from high hills near Pune recorded less RR and the susceptibility status was almost on par with Lo-S population (Table 3).

**TABLE 3:** Resistance pattern of field populations of *L. orbonalis* against various insecticides.

**Insecticide**	**Population**	**LC50 (ppm)**	**Slope ± SE**	Fiducial limits		**χ^2^ heterogeneity (DF)**	**Resistance ratio**
				**Lower**	**Upper**		
Fenvalerate	Bhubaneswar	259.2	2.6 ± 0.52	125.61	623.8	7.01 (3)	160
	Raichur	93.2	3.1 ± 0.2 2	56.1	125.8	3.92 (3)	57.5
	Dharmapuri	78.4	2.5 ± 0.23	53.2	145	2.81 (3)	48.3
	Pune	5.9	2.1 ± 0.11	2.51	16.32	2.91 (3)	3.6
	Varanasi	153.2	1.6 ± 0.23	75.2	432.4	3.37 (4)	94.6
	Bengaluru (Lo-S)	1.62	2.2 ± 0.42	0.85	3.57	2.62 (4)	–
Phosalone	Bhubaneswar	641.5	2.61 ± 0.22	283.51	2564.6	6.90 (3)	534.6
	Raichur	190.2	1.9 ± 0.2 1	110.1	280.5	3.90(3)	158.5
	Dharmapuri	113	3.3 ± 0.56	56.4	203.2	0.89 (3)	94.2
	Pune	8.81	1.7 ± 0.08	4.34	24.81	3.48 (3)	7.3
	Varanasi	435.8	1.9 ± 0.21	210.5	1202.8	1.37 (3)	363.2
	Bengaluru (Lo-S)	1.2	1.2 ± 0.21	0.521	2.42	1.91 (3)	–
Emamectin benzoate	Bhubaneswar	0.072	1.4 ± 0.09	0.291	0.204	3.4 (3)	7.2
	Raichur	0.1	1.1 ± 0.10	0.07	0.2	1.19 (3)	10
	Dharmapuri	0.07	1.5 ± 0.31	0.03	0.1	2.66 (3)	7
	Pune	0.012	1.8 ± 0.09	0.005	0.045	1.07 (3)	1.2
	Varanasi	0.55	1.2 ± 0.28	0.231	1.821	6.89 (4)	55
	Bengaluru (Lo-S)	0.01	1.4 ± 0.07	0.004	0.051	3.17 (3)	–
Thiodicarb	Bhubaneswar	211.6	1.71 ± 0.83	116.23	621.78	1.91 (3)	18.2
	Raichur	263.2	2.1 ± 0.21	121.3	467.9	2.3 (3)	22.7
	Dharmapuri	161.9	2.05 ± 0.30	83.82	341.7	7.2 (3)	13.9
	Pune	23.6	2.0 ± 0.12	32.01	184.8	1.3 (3)	2.03
	Varanasi	112	2.2 ± 0.18	56.82	456.8	1.7 (4)	9.6
	Bengaluru (Lo-S)	11.61	1.73 ± 0.32	5.462	23.56	4.7 (3)	–
Flubendiamide	Bhubaneswar	69.7	1.20 ± 0.08	36.34	173.35	3.7 (3)	303.0
	Raichur	43.1	1.72 ± 0.10	22.39	93.72	2.9 (3)	187.4
	Dharmapuri	61.5	1.3 ± 0.13	35.65	129.39	7.2 (3)	267.4
	Pune	6.79	1.10 ± 01.0	3.02	17.93	14.6 (4)	29.5
	Varanasi	61.3	1.79 ± 0.08	20.32	173.8	17.5 (4)	266.5
	Bengaluru (Lo-S)	0.23	–	–	–	–	–
Chlorantraniliprole	Bhubaneswar	2.29	0.66 ± 0.16	0.28	6.4	4.08 (3)	6.9
	Raichur	0.80	0.59 ± 0.16	0.02	2.9	2.80 (3)	2.4
	Dharmapuri	2.84	0.63 ± 0.15	0.41	7.94	4.92 (3)	8.6
	Pune	1.76	0.6 ± 0.16	0.12	5.59	3.08 (3)	5.3
	Varanasi	0.60	0.47 ± 0.15	0.002	3.2	5.68 (3)	1.6
	Bengaluru (Lo-S)	0.33	0.81 ± 0.26	0.003	1.24	1.55 (3)	–

### Activities of Detoxification Enzymes

Quantitative differences in the titer of GST and CE were determined using α-naphthyl acetate and 1-chloro-2,4-dinitrobenzene (CDNB) as substrates, respectively. Significant level of elevated GST activities (4.1–8.9-fold) was observed in field collected *L. orbonalis* as compared to susceptible Lo-S population. However, the overproduction of CE has not pronounced much (1.5–3.2-fold), but there was a significant level of overproduction in two field populations based on Mann–Whiteney U-test. The results indicated that GST activity was altered more profoundly than the CE (Table 4).

**TABLE 4:** Metabolic enzymes activities in the midgut of *L. orbonalis* populations.

***L. orbonalis* population**	**Glutathione -S- transferase**		**Carboxylesterase**	

	**Specific activity (nMoles/min/mg protein)**	**Fold variation as compared to Lo-S**	**Specific activity (μMoles/mg/min)**	**Fold variation as compared to Lo-S**
Bhubaneswar	70.5 ± 3.6*	5.6	6.02 ± 0.21*	2.8
Raichur	73.0 ± 3.6*	5.8	6.83 ± 0.21*	3.2
Dharmapuri	112.3 ± 11.2*	8.9	5.78 ± 0.33*	2.7
Varanasi	51.8 ± 2.8*	4.1	3.52 ± 3.4	1.7
Pune	103.4 ± 9.5*	8.2	3.28 ± 0.33	1.5
Bengaluru (Lo-S)	12.62 ± 1.3	–	2.12 ± 0.13	–

*Indicates significant differences by Mann–Whiteney U-test (*p* < 0.05) as compared to Lo-S population.

**FIGURE 1:**
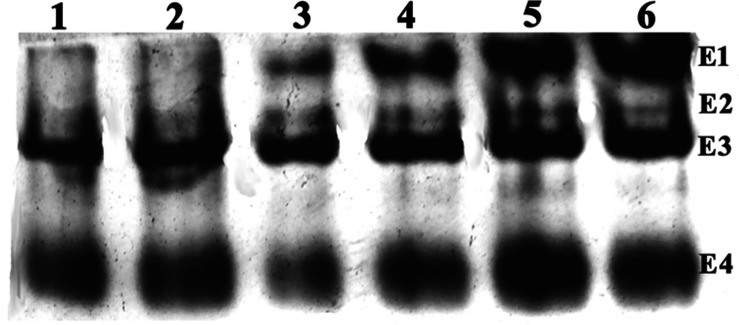
The Native PAGE of the esterase isozyme pattern of *L. orbonalis* (1. Lo-S, 2. Pune, 3. Varanasi, 4. Raichur, 5. Bhubaneswar, 6. Dharmapuri).

The qualitative differences in carboxylesterase isozymes were visualized under native PAGE after incubation of the gels in the α-naphthyl acetate as a substrate. Among the four major esterase activity bands (E_1_ to E_4_), the high molecular weight E_1_ and E_2_ bands were absent in Lo-S and Pune populations and faint in case of Varanasi population. However, the E_1_ band was very prominent and intense in other field populations indicating its over-production nature (Figure 1).

### Differential Expression of CE Genes

**FIGURE 2:**
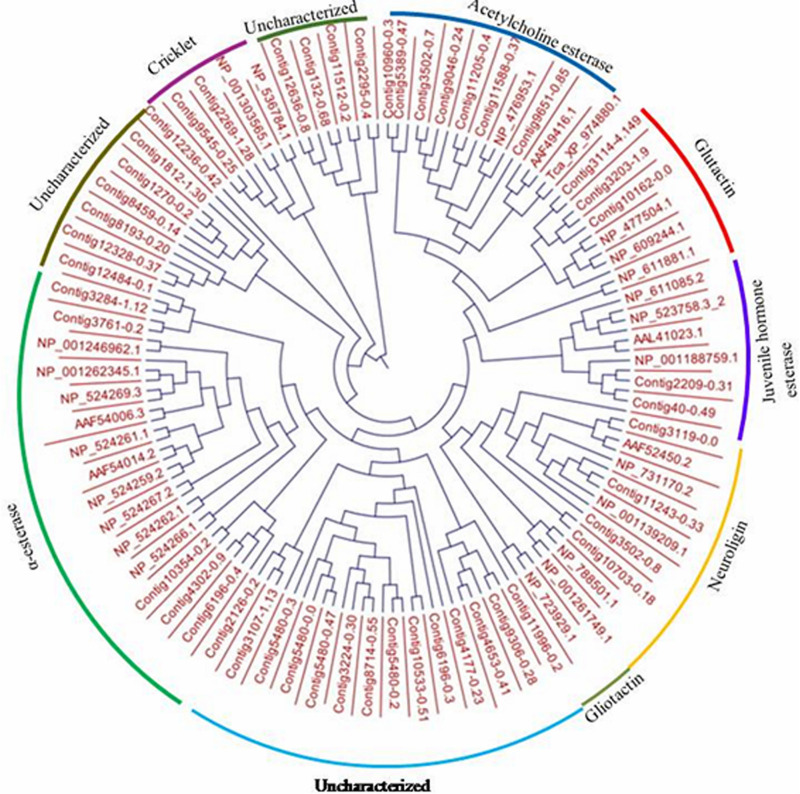
Phylogenetic relationships of 94 CE proteins of *L. orbonalis* with selected *D. melanogaster* protein representative of seven characterized carboxylesterase classes. Putative CE proteins in *L. orbonalis* were identified using hmmsearch against PFAM CE families (CE PF00135). The Maximum likelihood tree was constructed using CLC workbench.

**FIGURE 3:**
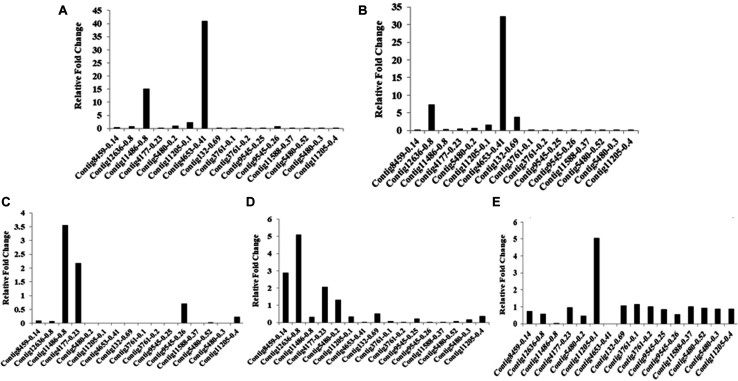
Transcription profiling of carboxylesterase genes in field collected populations of *L. orbonalis* depicted as fold change over the susceptible Lo-S colony. **(A)** Bhubaneswar, **(B)** Dharmapuri, **(C)** Pune, **(D)** Raichur, **(E)** Varanasi.

**FIGURE 4:**
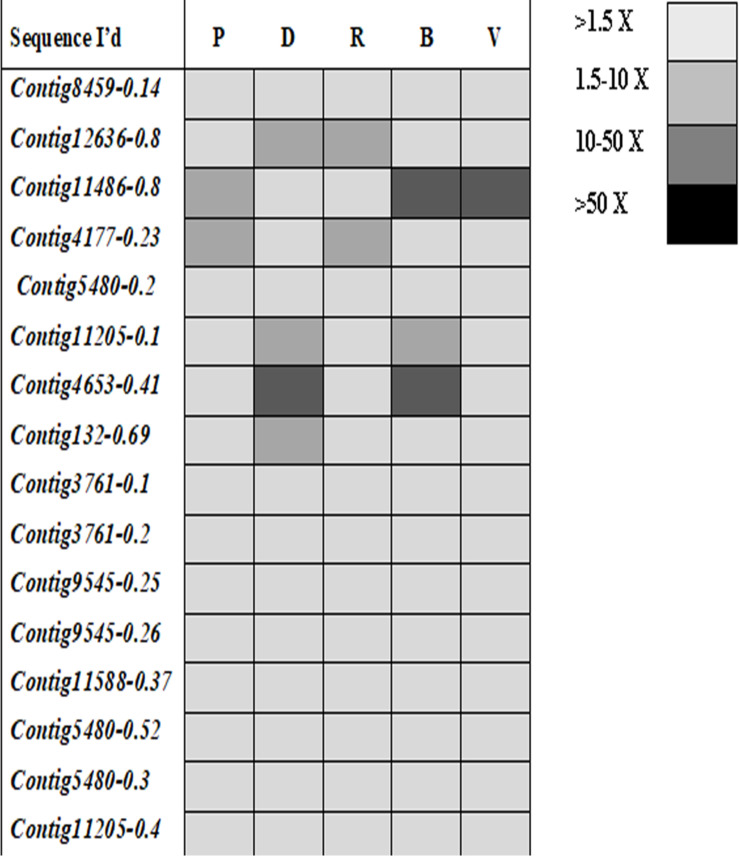
Expression profiles (fold changes over Lo-S population) of carboxylesterase genes across field collected *L. orbonalis* populations (P, Pune; D, Dharmapuri; R, Raichur; B, Bhubaneswar; V, Varanasi). The fold changes are indicated in different shades indicate significant difference as per Mann–Whiteney U-test *p*-value < 0.05.

Ninety-four CE genes were identified from the genome and transcriptome sequences of *L. orbonalis*. They were classified under seven subfamilies *viz*., α-esterases, gliotactin, glutactin, neurolignin, juvenile hormone esterases, acetylcholinesterases, and cricklet like orthologs (Figure 2). The length of the gene sequences ranged from 225 to 6498 bp. Expression profiling of 16 genes (homologous to resistant genes in other insects with ≥90% query coverage) was examined from the mRNA samples derived from late second instar larvae of five field-collected *L. orbonalis* populations by RT-qPCR. The CE genes contig11486-0.8 and contig4653-0.41 were over-expressed more than 10-fold as compared to susceptible Lo-S population (Figures 3, 4).

### Differential Expression of Glutathione S-Transferase Genes

**FIGURE 5:**
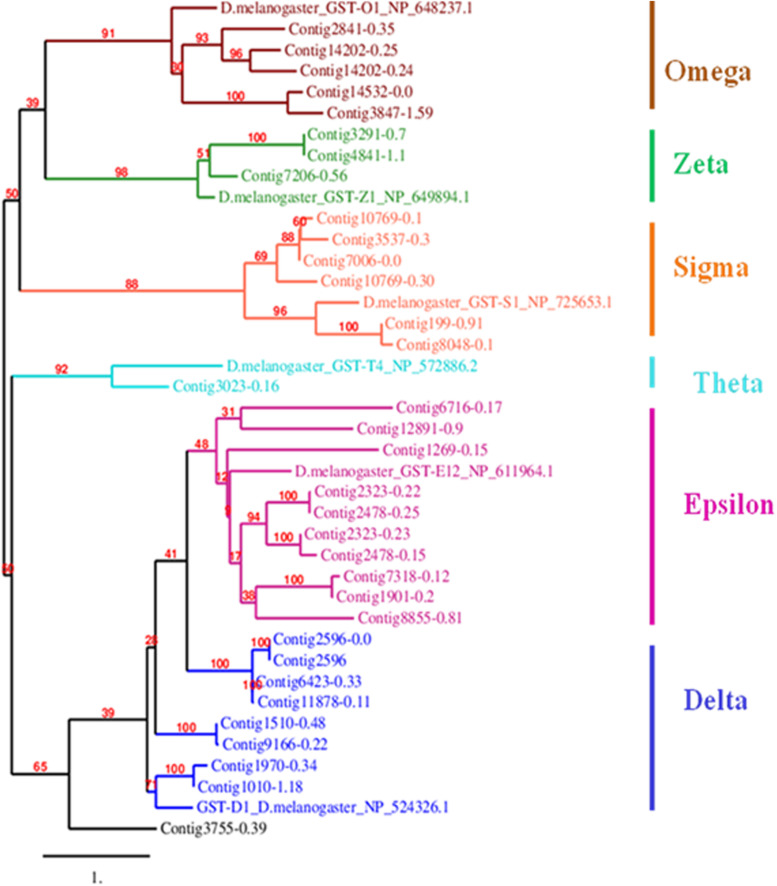
Phylogenetic tree of 34 predicted *L. orbonalis* glutathione S-transferase proteins with *D. melanogaster* genes representative of the six characterized GST classes. Putative GST proteins in *L. orbonalis* were identified using hmmsearch against PFAM GST families (GST-C PF00043, GST-N PF02798), the Maximum Likelihood tree with 1000 bootstraps was constructed using MEGA7 and TreeDyn online server was used to visualize the tree. Number on each node is bootstrap values in percent. Scale = 1 amino acid substitution per site.

**FIGURE 6:**
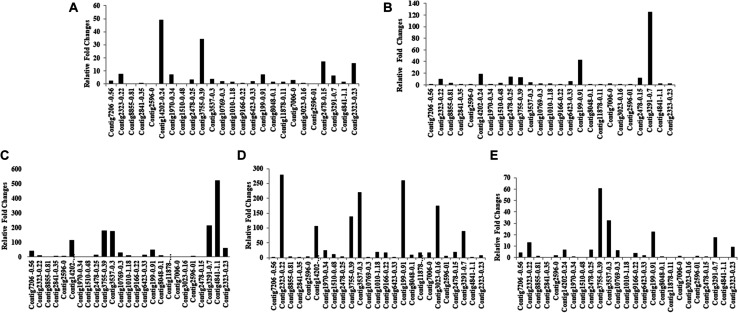
Transcription profiling of glutathione -S- transferase genes in field collected populations of *L. orbonalis* depicted as fold change over the susceptible Lo-S colony. **(A)** Bhubaneswar, **(B)** Dharmapuri, **(C)** Pune, **(D)** Raichur, **(E)** Varanasi.

**FIGURE 7:**
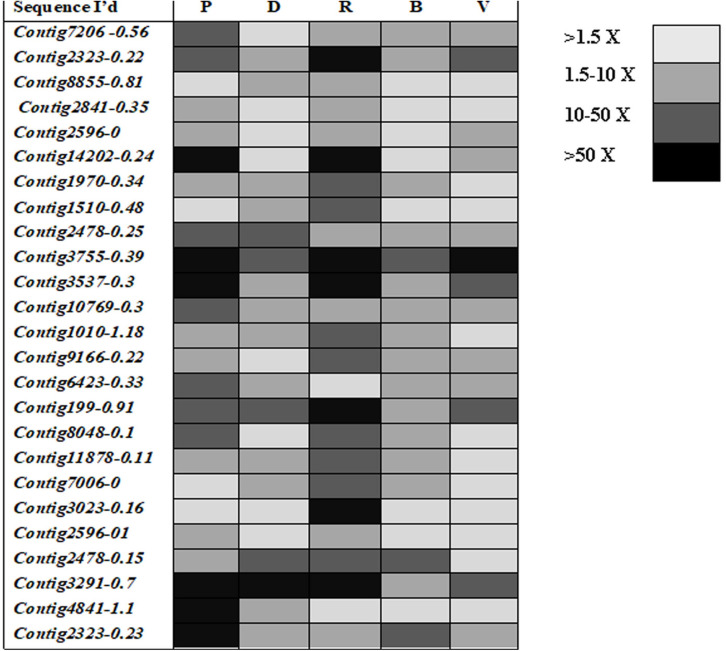
Expression profiles (fold changes over Lo-S population) of glutathione -S- transferase genes across field collected *L. orbonalis* populations (P, Pune; D, Dharmapuri; R, Raichur; B, Bhubaneswar; V, Varanasi). The fold changes are indicated in different shades indicate significant difference as per Mann–Whiteney U-test *p*-value < 0.05.

Mining of genome and transcriptome data of *L. orbonalis* yielded 33 unigenes codings for GSTs. Lengths of the identified GST unigene sequences ranged from 370 to 5,048 bp. The phylogenetic analysis revealed that the predicted GST genes are represented under all six classes of GST *viz.*, Epsilon (10 genes), Sigma (6 genes), Omega (5 genes), Delta (8 genes), Zeta (3 genes), and Theta (1 gene) (Figure 5). RT-qPCR analysis of 25 GST genes (homologous to other insects resistant genes) revealed that many of them were over-expressed in larvae collected from the field. The GST genes named contig2323-0.22, contig14202-0.24, contig3755-0.39, contig3537-0.3, contig199-0.91, contig3023-0.16, contig3291-0.7, contig4841-1.1, and contig2323-0.23 were very highly expressed (>50-fold) in one or more resistant field-collected *L. orbonalis* populations (Figures 6, 7) over the susceptible Lo-S population.

## Discussion

Damage caused by *L. orbonalis* is the major limiting factor in realizing maximum productivity of eggplant in many Asian countries including India, China, and Bangladesh. The high reproductive potential and shorter life cycle of the pest coupled with the evolution of multiple insecticide resistance pose a major challenge in profitable cultivation of eggplant (Prodhan et al., 2018). The insecticide resistance levels detected in the field collected populations ranged from low (>10-fold) to moderate (10–100-fold) and high (>100-fold) against fenvalerate (48.2–160-fold), phosalone (94–534.58-fold), emamectin benzoate (7–55-fold), thiodicarb (9.64–22.67-fold), and flubendiamide (29.51–363.91-fold). The resistance levels reflect the occurrence of differential selection pressure associated with long term history of insecticide use against *L. orbonalis*. However, the response to chlorantraniliprole was similar among the resistant field collected populations with RRs below 10-fold. The efficacy of chlorantraniliprole has been reported earlier (Kodandaram et al., 2013; Munje et al., 2015). The chlorantraniliprole and flubendiamide are anthranilic and phthalic diamides, respectively, are the newer insecticides with little or no cross-resistance among them and also with other classes of insecticides. They are the activators of the ryanodine receptor.

Insect employs various mechanisms to nullify the toxic effect of xenobiotic compounds. Insect metabolic enzymes play a major role in detoxification of plant allelochemicals and pesticidal compounds in addition to other physiological roles. Insect pests have evolved large reservoirs of various detoxification enzymes. Glutathione S-transferases mediated detoxification can be direct or by the metabolism of secondary products generated from other detoxification enzymes. The insecticides are metabolized rapidly into readily excretable water-soluble metabolites by reductive dehydrochlorination or by conjugation with reduced glutathione (Pavlidi et al., 2018). The carboxylesterase is a phase-1 detoxification enzyme that mainly hydrolyses organophosphates, carbamates, and synthetic pyrethroids.

The quantitative mechanisms underlying metabolic resistance is characterized by over-production due to gene amplification or transcriptional upregulation (Mohan et al., 2015; Feyereisen, 2015; Pavlidi et al., 2018). A qualitative mechanism occurs as a result of changes in the enzymatic characteristics. The present investigation on the enhanced metabolism of insecticides in resistant populations of *L. orbonalis* resistance is very prominent as inferred by elevated GST (1.2–2.6-fold) and carboxylesterases (6.3–13.1-fold) titers. The involvement of CEs and GSTs in insecticide resistance is commonly reported in many other insect species (Mohan et al., 2015; Pavlidi et al., 2018).

With the advent of genome and transcriptome information, multiple GSTs and carboxylesterases encoding genes have been characterized from insects and mites such as *Plutella xylostella, Culex* spp., *Tribolium castaneum, Bombyx mori, Leptinotarsa decemlineata, Tetranychus cinnabarinus, Musca domestica* and in many other insect species (Yang and Liu, 2011; Wang et al., 2014; Cui et al., 2015; Meisel and Scott, 2018; Pavlidi et al., 2018). *L. orbonalis* genome has large expansion of CE and GST genes which is comparable to many other insects. However, the GSTs have prominent role in insecticide resistance as compared to CEs. mRNA level of at least nine GSTs were over-expressed >50-fold where as none of the CEs showed very high levels of expression in the field collected resistant populations. The over-expression of few CE genes in field populations, especially from Varanasi, Raichur, and Bhubaneswar, also aligned with additional high molecular weight esterase band (E1) probably contributed to overall resistance. A similar high molecular weight esterase band associated with B-esterase type was reported from diamondback moth (Mohan and Gujar, 2003). In addition to qualitative changes in esterase banding pattern, there is a significant increase in the production of the CEs in at least three field populations. The over-production and appearance of new esterase isozymes might be the consequence of increased transcription or gene duplication events (Malathi et al., 2015; Panini et al., 2016).

Most of the GST gene expansions are from epsilon and delta subclasses together accounting for 18 genes (54.5%) out of 33 genes identified from the draft genome of *L. orbonalis*. These two GST subclasses are insect-specific, widely expanded in many other insect species whereas other classes have wider taxonomic distribution (Friedman, 2011). There were at least nine GSTs over-expressed >50-fold in one or more of the field-collected resistant populations of *L. orbonalis*. In *Anopheles gambiae, Apis mellifera, B. mori, D. melanogaster, Nasonia vitripennis*, and *T. castaneum* respectively 28, 8, 23, 37, 19, 35 GSTs and 51, 24, 76, 35, 41, 49 CEs were identified (Yu et al., 2008, 2009; Oakeshott et al., 2010). Similarly, the gene expansion of cytochromoe p450 monooxygenases (CYP) and their role in insecticide resistance in *L. orbonalis* were recently documented (Kariyanna, 2019; Kariyanna et al., 2020a). Together with the results of present and earlier studies it is very much evident that multiple metabolic enzymes contribute toward overall insecticide resistance in *L. orbonalis*. However, in many insect species including *L. orbonalis*, it is difficult to distinguish whether the expansion of these metabolic genes and their involvement in insecticide detoxification are driven from ancestry or adaptation to stress or both.

## Conclusion

In conclusion, the present study provides the first genomic level information on GST and CE gene families of *L. orbonalis* and their role in insecticide resistance. The information further supports insect resistance management through use of reduced risk pesticides including biologicals.

## Data Availability Statement

The datasets presented in this study can be found in online repositories. The names of the repository/repositories and accession number(s) can be found in the article/Supplementary Material.

## Author Contributions

BKr: conducted the experiment, analysis, manuscript writing, materials and methods, and hypothesis. AP: analysis, material methods, and corrections. RA: analysis and technical suggestion, manuscript correction, and resource supply. AA and PJ: bioinformatics and analysis. RG and BKl: resources supply and, technical guidance and suggestions. TV: resources supply, technical guidance and suggestions, and reviews and literatures. MB and JD: analysis, technical guidance and suggestions, and reviews and literatures. MM: designing experiment, resources supply, manuscript corrections, analysis, material and methods, technical guiding, and suggestions. YP: technical guidance and suggestions, and reviews and literatures. All authors contributed to the article and approved the submitted version.

## Conflict of Interest

The authors declare that the research was conducted in the absence of any commercial or financial relationships that could be construed as a potential conflict of interest.

## Footnotes

1 http://pfam.xfam.org

2 http://hmmer.janelia.org

3 http://www.ncbi.nlm.nih.gov/

4 http://www.uniprot.org/

5 http://www.megasoftware.net

6 www.treedyn.org

7 https://www.qiagenbioinformatics.comproductsclc-main-workbench

8 http://www.sigmaaldrich.com/life-science/custom-oligos/custom-dna/learning-center/calculator.html

## Supplementary Material

Supplementary Table 1 Highlights of the research.

Supplementary Table 2 Sequence information GST and CE from *L. orbonalis*.

Supplementary Table 3 Resistance genes of GST and CE from *L. orbonalis*.

Supplementary Table 4 Detailed sequence information of CE from *L. orbonalis*.

Supplementary Table 5 Detailed sequence information of GST from *L. orbonalis*.

Supplementary Table 6 NCBI ID and description for the Reference protein taken in GST and CE phylogeny.

Supplementary Table 7 Comparative characterization *L. orbonalis* CE genes with other group based on the nr hit.

Supplementary Table 8 Descriptive information of the *L. orbonalis* CE sequences.

Supplementary Presentation 1 Graphical representation of the manuscript.
