# Supplementary material for: Genome Mining and Expression Analysis of Carboxylesterase and Glutathione S-Transferase Genes Involved in Insecticide Resistance in Eggplant Shoot and Fruit Borer, Leucinodes orbonalis (Lepidoptera: Crambidae)
Source: Front Physiol. 2020 Nov 19;11:594845. doi: 10.3389/fphys.2020.594845 (PMC7713791; doi:10.3389/fphys.2020.594845)
Supplement: Supplementary Table 1 — Highlights of the research. [file Table_1.DOC]

**GLUTATHIONE S-TRANSFERASE**

1. **>Contig7206**

ATGGCTAAATCATCATTCAGAAACAACCCTGTGCTGTACTCATACTGGAGATCATCATGC

TCATGGAGAGTGAGAATCGCTCTGAACCTGAAAGAAATCCCTTACGACATCAAAGCTGTG

TCACTGATCAAAGGTGGTGGTGAACAACACTGCAACGAATACAGAGAAGTGAACCCTATG

GAACAAGTGCCTTCACTGTGCATCGACGGTCACACTCTGGTGGAATCACTGTCAATCATG

CACTACCTGGAAGAAACTAGACCTCAAAGACCTCTGATGCCTCAAGACTGCATGAAAAGA

GCTAAAGTGAGAGAAATCTGCGAAGTGATCTCATCAGGTATCCAACCTCTGCAAAACCTG

ATCGTGCTGATCTACGTGGGTGAAGAAAAAAAAAAAGAATGGGCTCAACACTGGATCAAC

AGAGGTTTCAGAGCTGTGGAAAAACTGCTGTCAGCTTCAGCTGGTAAATACTGCGTGGGT

GACGAAATCACTCTGGCTGACTGCTGCCTGGTGCCTCAAGTGTTCAACGCTAGAAGATTC

CACGTGGACCTGAGACCTTTCCCTATCATCCTGAGAATCGACAGAGAACTGGAAAACCAC

CCTGCTTTCAGAGCTGCTCACCCTTCAGCTCAACCTGACTGCCCTCCTGAAGTGGCTAAA

1. **>Contig2323**

ATGGTGCTGACTCTGTACAAAAAAGACACTTCACCTCCTTGCAGATCAGTGTTCATGACT

ATCCACGCTCTGCACATCACTGACGTGAACTACATCGACGTGTACCTGCCTGCTGGTGAA

CACCTGACTGAAGAATTCATCAAACTGAACCCTCAACACACTGTGCCTCTGCTGAAAGAC

GGTGACTTCCTGATCTGGGACTCACACGCTATCTGCGGTTACCTGGTGTCAAAATACGGT

GAAAACGACCACCTGTACCCTAGAGACCCTAAAAAAAGAGCTATCGTGGACCAAAGACTG

CACTTCGACTCAGGTGTGCTGTTCAACGCTCTGAAAACTACTGTGGTGCCTCTGCTGTAC

GCTGGTGAAAAACTG

1. **>Contig2323**

ATGGTGCTGACTCTGTACAAACTGGACGCTTCACCTCCTGTGAGAGCTGTGAACATGGTG

ATCGAAGCTCTGGGTCTGGAAGTGGAAACTGTGACTGTGAACATCCTGAACAAAGACAAC

CACAAAGACGAATACCTGCAAATGAACCCTCAACACACTATCCCTACTCTGAAAGACGAC

GACTTCGTGATCTGGGACTCACACGCTATCACTACTTACCTGATCACTAAATACGGTAAA

GACGACTCACTGTACCCTTCAGACCCTAAAATCAGAGCTAGAATCGACCAAAGACTGCAC

TTCGACTCAGGTGTGCTGTTCGCTACTCTGAGAAACGCTGTGGGTTCAATCCTGTACCAA

GGTGAAACTACTCTGACTCCTGAACACTCAGAAAAAATCAAAAACGCTTACGAATTCACT

GAAAAATTCCTGACTTCACAATGGCTGGTGGGTGACCACGTGACTATCGCTGACATCTGC

TGCGTGGCTACTATCTCATCAATGAACGTGCTGCTGCCTATCGACGAAGGTGTGTACCCT

AACCTGGCTGCTTGGGTGGAAAGATGCTCAGCTCTGGACTTCTACATCAAAGGTAACAAA

CCTGGTCTGGAACAATTCACTCAACTGATCAAATCAAAACTGAGA

1. **>Contig8855**

CTGTACAAAATCAACCTGGGTGCTGCTTCAAGAACTGCTATGATGGTGCTGGACATCTTC

AACGTGCCTGTGGAACTGATCGAAGTGTCACTGCTGAAAAAAGAAAACCTGACTCCTGAA

TTCCTGCAAGTGAACCCTTGCCACACTGTGCCTGTGCTGGTGGACGAAGACATCACTATC

AGAAACTCACACGCTATCTGCATCTACATCACTGAAGTGTACGGTCAAAACTCAAACCTG

TACCCTAAAGACCTGATCCAAAGAACTAACGTGAACCAACTGCTGTTCTACAACGAATCA

GTGCTGATCTCAAGATTCGGTAAACTGTCAGTGTCAGTGTTCAGAGGTGCTAACGAAATC

TCAGAAACTCAAGTGAACGAAATCAAAGAATCATACGACATCCTGAACACTCTGCTGACT

AAAACTCAATTCCTGGCTTGCGACTTCATCACTATCGCTGACATCGCTGCTTTCTCAAAC

GTGTCAGGTCTGGTGGAAATCGTGCCTCTGGACCTGTCAAGAAACGCTAAACTGGACACT

TGGGTGAGACAATGGAGAAACAACCTGATCGCTAAAAAAATCAACACTCCTGCTACTAAA

CTGTTCAAAAAAAACCCTAAAGCTAGACTGAACCTGTACCAAGTGAGAAAACTGGTG

1. **>Contig2841**

ATGTCAGAAAAACACCTGAAATCAGGTGACGTGCTGCCTCAATACAACGGTAAACTGAGA

CTGTTCGCTATGAGATTCTGCCCTTACGCTGAAAGATCAGTGCTGGTGCTGAACTCAAAA

GGTCTGGACTACGACCTGGTGTTCGTGAACCTGGACCACAAACCTGAATGGCTGTTCGAC

TTCTCACCTAAAGGTACTGTGCCTGCTCTGGAATACGAACAAGGTAAAGCTATCTTCGAC

TCAAACATCATCAACGTGTACCTGGACGAAAAATACCCTGAAAAACCTCTGCAAGCTGCT

GACCCTCTGAGAAGAGCTCAAGACAAACTGATCGTGGAAAACTTCGCTGCTGCTCAATCA

GCTTACTACACTGCTGCTTTCAACTCACAAGCTCTGCAACCTTCACACATCGAAAACTAC

CACAAAGGTCTGGAAGGTCTGCAAAAAGAACTGGAAGTGAGAGGTACTAAATTCCTGCAC

GGTGACGACGCTGGTTGGGTGGACCTGACTATCTGGCCTTTCCTGGAAAGATTCCTGGCT

CTGCCTCTGCTGGGTAAACCTGAATTCGCTATCGACAAATCAAAATACGGTCTGCTGTCA

ACTTACATCGAAAACATGAAAAACGTGCCTGCTGTGAAATCATACTGCCTGGCTCCTGAA

ACTCACGCTAAATTCACTGAATCAAGAGCTAAAGGTGACCCTGACTACAACATGCTGGAC

ACTTCAGCTGTGTGCTGCATGAGACCTAGAAAAAAAAAAGAA

1. **>Contig14532**

TTCCCTGTGAAAATCCCTGTGCTGGAAATCCCTACTGACCACGGTGACAAATTCCTGTTC

GAATCAGTGGTGATCTGCGACTACCTGGACGAAAGATACCCTAGAAACCCTCTGCACTCA

AAAGACCCTTACGTGAAAGCTCAAGACAGACTGCTGATCGAAAGATTCAACGAACTGATC

AAAGGTTCACTGGAATGCTTCGACACTAACTTCGCTTTCGGTTCAGAACAAATCGTGCAA

ACTCTGGACATCTTCGAAAAAGAACTGGCTCTGAGAGGTACTAACTACTTCGGTGGTAAC

AGACCTGGTATGCTGGACTACATGATCTGGCCTTGGGTGGAAAGACTGTACCTGCTGAGA

TGCCTGAACGAAAAAAAATTCGACGAAAAAAGATCACTGTTCCCTAACTTCGCTGACTGG

GGTGACCAAATGCAACTGGACGAAGTGGTGAAAAAACACTCATCATCACCTGCTGAATAC

TTCGACTACTACAAAAACGCTAGAGCTCACTCAATGGGTTACTACCTG

1. **>Contig2596**

ATGTCACTGAAACTGTACCACTTCCCTATCGCTGTGCACCACGCTGTGCTGTACCTGCTG

CAAGAACAATCAGGTTTCAGATCAAAAACTAACTCAGAATTCATCAAAAAAGAACAACTG

TCAGACTCATTCATCAAAATCAACCCTCAACACTGCGTGCCTACTCTGGAAGACGACGGT

TTCGTGCTGTGGGAATCAAGAGCTATCGCTTGCTACCTGGTGGACAAATTCGCTAAAGAC

GACCAACTGTACCCTAAAGACCTGGAAAAAAGAGCTCTGGTGAACCAAAGACTGTACTTC

GACTCATCATCACTGTACGTGAAAATCAGAGCTATCTGCTTCCCTATCCTGTTCCTGGGT

GAAACTGAAATCAAAAAAAACCTGAAAGACGACCTGAACGGTACTCTGGTGTTCCTGGAC

CAATTCCTGAACGACACTAAATGGGTGGCTGGTGACTCAATCACTATCGCTGACACTTCA

ATCTACGCTTCACTGTCATCAATCCTGGCTGTGGGTTGGGACATCACTAAATTCCCTAAC

ATCCAAAGATGGTCAAGAATCGTGAACCTGCCTGGTTACGCTGAAAACGAAGAAGGTGCT

AAAGCTTTCGGTGAAGCTGTGAAAAAAAACCTGAAACAA

1. **>Contig7318**

ATGCCTATCAAAATCTACAAAGCTGACGTGTCACCTCCTGCTAGAGGTGCTCTGATGGCT

CTGGAACTGCTGGGTCTGAACTACGAAACTGAAGACCTGAACGTGATGAAAGGTGACACT

AGAACTCCTGAATACACTGCTCTGAACCCTGAACACACTATCCCTTTCCTGGTGGACGGT

GACTTCAAACTGGGTGACTCACACGCTATCATCACTTACCTGGTGTCAAAATACGGTGGT

GACAAAAGATCAACTCTGTACCCTTCAGCTGTGGAAGAAAAAGCTATCATCGACCAAAGA

CTGTTCCTGGACGCTGACGTGCTGGGTTTCCTGGAAAAATACCTGAACCAAAGAGAATTC

CTGGCTGCTAACCACATGACTGTGGCTGAACTGTCACTGGTGGCTTCAATCTCAACTCTG

GAAACTTTCATCTCACTGGACGAATTCCCTAAACTGAAAGCTTGGTTCGAAAAACTGAAA

AAATACGAACTGGTGCCTGAATCAGAA

1. **>Contig14202**

ATGATCGCTTCAACTGCTTCACTGGCTGGTCACATCCTGGTGACTTCATTCAAACTGCTG

TCACCTGTGGGTCAAGCTCTGGGTTACGTGCTGCCTTACTTCAACGCTATGGCTTCAAAA

ATCAACTTCAACACTAAACACCTGGCTAAAGGTGACCCTCTGCCTCCTTACACTGGTAAA

CTGAGAGTGTACAACATGAGATTCTGCCCTTACGCTCAAAGAACTATCCTGGCTCTGAAC

GCTAAACAAATCGACTACGAAGTGGTGAACATCAACCTGGCTAACAAACCTGAATGGCTG

ACTTCAAAATCAGCTTTCGGTAAAGTGCCTTCACTGGAAATCGAAGAAAACGTGTGCATC

TACGAATCACTGATCACTGTGGAATACCTGGACGACGTGTACCCTCAAAGACCTCTGATG

TCAAAAGACCCTGTGCAAAAAGCTATGGACAAAATCATCGTGGAAGCTATGTCACCTGTG

TACACTCTGATCATCGCTGCTATCAAATCACCTGACTCAATCTCAGAAGCTATGAAAGAC

GCTTACTACAAAGCTCTGGACTTCCTGCAAGAACAACTGAAAAAAAGAGGTACTAAATTC

CTGGGTGGTTCAGAACCTGGTTTCGCTGACTACATGATCTGGCCTTGGTTCGAAAGAATC

CACCTGATCGACGAAATCGACGGTCTGTCAATCGACTCACAAAAATACAAACCTATCCTG

GACTACTTCGGTGCTATGCTGGAAGACCCTGCTGTGAGACAATACCTGATCCCTAAAGAA

GTGCAACAACAATTCGTGGAATCATACAAAAGAGGTCAACCTATCAACTACGACCTGCTG

CTGGAACAA

1. **>Contig14202**

ATGCACCTGATCTTCTTCTTCTCAGGTGACCCTCTGCCTCCTTACAACGGTACTCTGAGA

GTGTACAACATGAGATTCTGCCCTTTCGCTCAAAGAACTATCCTGGCTCTGAACGCTAAA

CAAGTGCACTACGAAGTGGTGAACATCAACCTGATGAACAAACCTGACTGGCTGTTCGAA

AAATCACCTTTCGGTAAAGTGCCTGCTCTGGAAATCAAAGAAGGTCAAACTATCTTCGAA

TCACTGGTGACTGTGGAATACCTGGACGAAGTGTACCCTAACAGACAACTGCTGTCAAAA

GACCCTGTGGAAAGAGCTTGCGACAGAATGACTGTGGAAGCTTCAGTGGCTATCCACAAC

CTGTTCTTCAAACTGGTGAAAAGACCTGAAGAAGTGTCAGACGAAAACATCGCTGCTTTC

AGAAAAGCTCTGGACTTCATCCAAGCTAAACTGGTGAAAAGAGGTTCAAAATTCCTGTTC

GGTAAAGAACCTGGTTACGTGGACTACATGATCTGGCCTTGGTTCGAAAGACTGCAAGCT

CTGGAAGACTCAAGAGCTCAACTGGACAAACAAAAATACGACACTCTGCTGAAATACATC

AACAACATGCTGCTGGACCCTTCAGTGTCAAAATACCTGGTGCCTGGTGACGTGTTCAAA

AAATTCCACTCAGGTTTCCTGCTGTCATCAGGTCCTGACTACGAACTGCTGAACAAA

1. **>Contig1970**

CTGTACCCTGAAGAAGCTAAAGCTAGAGCTCTGGTGGACCAAAGACTGTACTTCGACATC

GGTACTCTGTACCAAAGATTCTCAGACTACTTCCTGAACCCTCAACACACTGTGCCTACT

CTGGTGGACGACGGTTTCTCAATCTGGGAATCAAGAGCTATCATCACTTACCTGGTGAAC

AAATACGGTAAAGGTAACCCTCTGTACCCTGAAGAAGCTAAAGCTAGAGCTCTGGTGGAC

CAAAGACTGTACTTCGACATCGGTACTCTGTACCAAAGATTCTCAGACTACTTCGAAAAA

CTGGCTAAAGTGGACGACGCTCTGAGACTGTTCGACGTGATCCTGGAAGGTCAAAAATAC

TCAGCTGGTAACAACCTGACTGTGGCTGACCTGTCACTGGTGGCTTCAATCTCATCATTC

GAAGCTTCAGACATCAACTTCCTGAAATACAACAACGTGAAAAGATGGTACGAAACTGTG

AAATCAACTGCTCCTGGTTACGAAGAAGCTAACGGTAAAGGTCTGCAAGCTTTCAAAGAA

CTGGTGAACAACATGCTGAAAAAA

1. **>Contig1510**

ATGGACAAATCAGTGGCTGCTAAAATCCAATCAGCTGACAACGCTGTGGCTAGATCAAAA

AACAAAATGCCTACTCAACCTATCAAACTGTACTACCTGCCTCCTTCACCTCCTTGCAGA

GCTGTGATGATGACTGCTAGAGTGCTGGGTATCGAACTGGAACTGATCGTGACTAACCTG

ATGGAAGGTGCTCACCTGGAACCTGAATTCGTGAAAATGAACCCTCAACACACTATCCCT

ACTATGGACGACTCAGGTTTCATCCTGTGGGAATCAAGAGCTATCATGACTTACCTGGTG

AACGCTTACGGTAGAGACGACACTCTGTACCCTAAAAACCCTAGACTGAGAGCTATCGTG

GACCAAAGACTGAACTTCGACCTGGGTACTCTGTTCATGAGATACATCAACCTGTACGGT

CCTATGCTGTTCAAAGGTGACAAACTGGACGACGAAAAAGCTGCTAAACTGAACGAAGCT

ATCGGTTGGCTGAACACTATGCTGGACGGTAAAGCTTTCGTGGCTGGTGACAACATGACT

GTGGCTGACATCTCAATGGTGGTGGTGTTCACTTGCCTGGAAGCTTTCGAATACGACTTC

TCACAATACGAAAACGTGACTAAATGGTTCGGTGTGATGAAAAAATCACTGGAACCTTAC

GGTTACTCAGAAATCGACGAAGCTGGTGCTCAAATGCTGGCTACTTTCCTGAAATCACAC

GCTAAC

1. **>Contig2478**

ATGGTGCTGACTCTGTACAAAAAAGACACTTCACCTCCTTGCAGATCAGTGTTCATGACT

ATCCACGCTCTGCACATCACTGACGTGAACTACATCGACGTGTACCTGCCTGCTGGTGAA

CACCTGACTGAAGAATTCATCAAACTGAACCCTCAACACACTGTGCCTCTGCTGAAAGAC

GGTGACTTCCTGATCTGGGACTCACACGCTATCTGCGGTTACCTGGTGTCAAAATACGGT

GAAAACGACCACCTGTACCCTAGAGACCCTAAAAAAAGAGCTATCGTGGACCAAAGACTG

CACTTCGACTCAGGTGTGCTGTTCAACGCTCTGAAAACTACTGTGGTGCCTCTGCTGTAC

GCTGGTGAAAAATCATTCAGACCTGAAAACCTGGCTCAAATGAAAACTGCTTACGAATTC

ATGGAAAAATTCCTGACTACTCCTTGGCTGGCTGGTAAAGAAGTGACTCTGGCTGACATC

TGCTGCGTGTCATCAATCTCATCAATGAACGAAATCTTCCCTATCGAAGAATCAATCTAC

CCTCACCTGACTGGTTGGCTGAAAAGATGCTCAGAACAAGAATTCTTCGTGAAAGGTAAC

TCAAAACAAATCCTGCTGTTCAGAGAAATGATCACTAACAAACTGTCACAAAAC

1. **>Contig3755**

ATGGTGCTGAAACTGTACGGTGTGTCAGACGGTCCTCCTTCACTGTCAGTGAGACAAGCT

CTGACTCACCTGCAAATCCCTTTCGAACTGATCAACATGAACCCTCAAAAAGAAATCCCT

GTGCTGGACGACGAAGGTTTCTACCTGGGTGAATCAAACGCTATCATGCAATACGTGTGC

GACAAATACAAACCTTCATCACCTCTGTACCCTCAACAACCTAAAGCTAGAGCTCTGGTG

AACCACAGACTGTGCTTCAACCTGTCAACTTACTACGCTCACATCTCAGCTTACACTATG

GCTCCTATCTTCTTCGACTACGAAAGAACTCCTCTGGGTCTGAAAAAAGTGCACATCGCT

CTGGACGTGTTCGAAACTTACCTGGAAAGACTGGGTACTGGTCACGCTGCTGCTGACCAC

CTGACTATCGCTGACTTCCCTCTGATCAACTCAACTATGACTCTGGAAGCTATCGACTTC

GACTTCAAAAAATACAAAAGAATCCACAAATGGTACAACGACTTCAAATCAAACTACCCT

GAACTGTGGAAAATCTCAGAAGGTGCTCTGAAAGAAATCCAACACTTCGCTGCTAACCCT

CCTGACCTGACTCACATGGACCACCCTATCCACCCTATCAGAAAAAACAACAAA

1. **>Contig10769**

ATGGCTAGAAAACTGCAATACTTCAACCTGAACGGTCTGGCTGAACCTATCAGACTGATG

CTGCACTACGCTGGTTCAGAATTCGAAGACGTGAGATACGACTTCAAATCATGGCCTATC

AAATCAGTGAAAGACTCACTGCCTTACGGTCAAATGCCTATCTACGAAGAAAACGGTAGA

GTGCTGAACCAATCACTGGCTATCGCTAGATACATCGCTTCACAAAACGGTCTGCTGCCT

ACTGACTCATGGGACCAAGCTATCCTGGACGCTACTGTGAACAACATCTTCGACTTCTGG

TCAAACGTGGTGTCATACGTGAAAGAAACTGACGCTGAAAAAAAAAAAGCTATCAAAAAA

CAAATCCTGGAAGAACTGGTGGACTACTACTTCTCAAGATTCGAAAAAGAACTGAACGCT

CACGGTGGTTTCTTCGTGGGTCAACTGTCATGGGCTGAATTCGTGCTGGTGGGTATCATG

GAATCATGCGACCTGTTCCTGGAAGAAAAAATCTCATACCAAAACTACCCTGCTGTGCAA

GGTCTGTTCAACAAAATCCTGTCACTGCCTGGTGTGAAAGAATACGTGGCTTCAAGACCT

CCTTACGTGTGGCCTCAAAAC

1. **>Contig3537**

ATGGAAGAAACTACTCTGTTCCCTTTCGAAAGAATCGCTTCACCTTCAGCTCTGCCTTAC

GGTCAACTGCCTCTGTACGAAGAAGGTGACAAATCACTGAACCAATCACTGGCTATCGCT

AGATACCTGGCTAACAAATTCAACCTGCTGCCTATCGACCCTTGGCAACAAGCTGTGCTG

GACGCTGTGGTGCTGAACATCTACGACTTCTGGGGTAAAATCCTGCCTTACTTCAGAGAA

CAAGACCCTGTGAAAAAAGCTGCTATCAGAGAAGAAATCATCAACGAAGTGATCCCTTTC

TACTTCTCAAGATTCGAAAGAGAACTGAAAGAAGCTAACGGTCACTTCGCTGGTAAACTG

TCATGGGTGATCTCATCACTGCTGGAACTGCTGAAACAACTGACTGCTACTGAAACTTGC

CAC

1. **>Contig3847**

ATGACTATCACTAACGCTGGTGGTATCCCTGGTTCATCACCTCCTCCTCCTCTGGGTGAC

CAACTGAGACTGTACCACGTGGACATGAACCCTTACGGTCACAGAGTGCTGCTGATCCTG

GAAGCTAAAAAAGTGAAACACGAAGTGTACAGACTGGACCCTCTGAGACTGCCTGAATGG

TTCAGAGCTAGAAACCCTAGACACGACGACGGTGCTACTCACAAATTCCTGTTCGAATCA

GTGGTGATCTGCGACTACCTGGACGAAAGATACCCTAGAAACCCTAGACACTCAAAAGAC

CCTTACGTGAAAGCTAGAAGATTCATCCACAGAGAACTGATCAAAGGTTCACTGGAATGC

TTCGACACTAACTTCGCTTTCGGTTCAGAACAAATCGTGCAAACTCTGGACATCTTCGAA

AAAGAACTGGCTCTGAGAGGTATGCTGGACTACATGATCTGGCCTTGGGTGGAAAGACTG

TACCTGCTGAGATGCCTGAACGAAAAAAAATTCGACGAAAAAAGATCATTCATGCTGAAA

TGGCAATGGGCTGGTCACATCTGCAGAAGAACTTCAGGTAGATGGTCAAGAAGAGTGCTG

GAATGGAGACCTAGAAGAGGTAAACCTTCAGTGGGTCCTATGGACGGTAGACTGGGTAAA

GGTGGTTGGATCAGAAAAGCTGAAGACAGAGACATCTGGAGAGAACTGAGACTGCTGAGA

GACTCAAGAATGTCACAAAACAACGGTAGATGGTCAAGAAGAGTGTGGTCAGGTGACCCT

TTCGGTCAAAGATCAGTGGCTAGAAGAGACATCTGCGCTGAATGGGTGCTGTCAATCACT

ATCTCATACGCTATGATCTCAAAACTGAAATGGCAATGGGCTGGTCACATCGTGAGAAGA

ACTGACGGTAGATGGGGTGGTAAAGTGCTGGAATGGAGACCTAGAAACGGTAAAAGATCA

GTGGGTAGACCTCTGACTAGATGGACTGACCTGGTGAAAGTGGCTGGTTCAAGATGGATC

TCAACTGCTAGAGACAGATCAGCTTGGAAATCACTGGGTGAAGCTTTCGTGCAACAATGG

ACT

1. **>Contig1010**

ATGGGTATCGACCTGTACTACGTGCCTGGTTCAGCTCCTTGCAGAGCTGTGCTGCTGACT

GCTAGAGCTCTGAACATCAACCTGAACCTGAAACTGGTGGACCTGCACCACGGTGAACAC

CTGAAACCTGAATACCTGAAACTGAACCCTCAACACACTGTGCCTACTCTGGTGGACGAC

GGTTTCTCAATCTGGGAATCAAGAGCTATCATCACTTACCTGGTGAACAAATACGGTAAA

GGTAACCCTCTGTACCCTGAAGAAGCTAAAGCTAGAGCTCTGGTGGACCAAAGACTGTAC

TTCGACATCGGTACTCTGTACCAAAGATTCTCAGACTACTTCTACCCTCAAGTGTTCGGT

GGTGCTCCTGCTGACAAAGAAAAACTGGCTAAAGTGGACGACGCTCTGAGACTGTTCGAC

GTGATCCTGGAAGGTCAAAAATACTCAGCTGGTAACAACCTGACTGTGGCTGACCTGTCA

CTGGTGGCTTCAATCTCATCATTCGAAGCTTCAGACATCAACTTCCTGAAATACAACAAC

GTGAAAAGATGGTACGAAACTGTGAAATCAACTGCTCCTGGTTACGAAGAAGCTAACGGT

AAAGGTCTGCAAGCTTTCAAAGAACTGGTGAACAACATGCTGAAAAAA

1. **>Contig1269**

ATGTTCTCAACTGTGAACAAATGCAAAATCCTGATGACTACTTGCTCATCAAACATCTAC

ATCAGAACTCTGACTAACGGTAAAACTCTGACTCTGTACGGTGACAACGCTTCACCTCCT

GTGAGATTCGTGCAAATGACTGCTTCATTCCTGGACATCAAAATGAACTTCCACAAAATC

GACCTGTTCAAAGGTGAAAACAAAACTGACTCATACGCTAAAGTGCCTGCTCTGGCTACT

GAAAACTCAGTGATCACTGACTCACACGCTATCGCTCTGTACCTGTGCAGACAAGTGCCT

GACCAAGACCTGTACCCTAAAGACACTCTGTCAAGAGCTAACGTGGACCAAATGCTGTTC

CTGAACGCTGGTTCACTGTTCCCTCTGGACAGATCAGTGTTCACTGACTACTTCGCTGGT

AAACCTCTGCACGACGACAAACTGGAAGAATGGAGACAACTGCTGGACTACCTGGAACTG

AGACTGAAAAACCACCAATGGATCGCTGGTGACAAAATCACTCTGTCAGACCTGTGCTGC

GGTTCAACTGTGTCATCACTGCAACTGCTGGTGCCTCCTACTGACAAACACGACAACTTC

AAAGACTGGATGAGAAGACTGGAACAAATCCCTTGCTTCTCAATGAACAAACAAGGTATC

GCTAGACTGTCAGAATTCGTGGAAAAACTGAAA

1. **>Contig6423**

ATGTCACTGAAACTGTACCACTTCCCTATCTCAGGTCCTTCAAGAGGTGCTCTGCTGGCT

GCTAGAGCTATCGGTGTGCCTATCCAAATCCAAATCGTGAACCTGTTCAAAAAAGAACAA

CTGTCAGACTCATTCATCAAAATCAACCCTCAACACTGCGTGCCTACTCTGGAAGACGAC

GGTTTCGTGCTGTGGGAATCAAGAGCTATCGCTTGCTACCTGGTGGACAAATTCGCTAAA

GACGACCAACTGTACCCTAAAGACCTGGAAAAAAGAGCTCTGGTGAACCAAAGACTGTAC

TTCGACTCATCATCACTGTACGTGAAAATCAGAGCTATCTGCTTCCCTATCCTGTTCCTG

GGTGAAACTGAAATCAAAAAAAACCTGAAAGACGACCTGAACGGTACTCTGGTGTTCCTG

GACCAATTCCTGAACGACACTAAATGGGTGGCTGGTGACTCAATCACTATCGCTGACACT

TCAATCTACGCTTCACTGTCATCAATCCTGGCTGTGGGTTGGGACATCACTAAATTCCCT

AACATCCAAAGATGGGTGAAAGACTGCGAATCACTGCCTGGTTACGCTGAAAACGAAGAA

GGTGCTAAAGCTTTCGGTGAAGCTGTGAAAAAAAACCTGAAACAA

1. **>Contig9166**

ATGGACAAATCAGTGGCTGCTAAAATCCAATCAGCTGACAACGCTGTGGCTAGATCAAAA

AACAAAATGCCTACTCAACCTATCAAACTGTACTACCTGCCTCCTTCACCTCCTTGCAGA

GCTGTGATGATGACTGCTAGAGTGCTGGGTATCGAACTGGAACTGATCGTGACTAACCTG

ATGGAAGGTGCTCACCTGGAACCTGAATTCGTGAAAATGAACCCTCAACACACTATCCCT

ACTATGGACGACTCAGGTTTCATCCTGTGGGAATCAAGAGCTATCATGACTTACCTGGTG

AACGCTTACGGTAGAGACGACACTCTGTACCCTAAAAACCACGAC

1. **>Contig199**

ATGCCTAAAGTGAAATACTCATACTTCCCTGTGAAAGCTCTGGGTGAATCAGCTAGACTG

CTGCTGTCATACGGTGGTCAAGAATTCGAAGACAACAGAGTGTCAATGGAAGACTGGCCT

CAATTCAAACCTAAAATCGACGGTAAAAAATACGCTCAATCAGGTGCTATCGCTAGATAC

CTGGGTAGAAAATACGGTATCGCTGGTTCATCAATCGAAGAAGACCTGGAAATCGACATG

AACGTGGACTTCATCAACGACATCAGAGCTAAAGCTGCTCTGGTGGCTTACGAAGCTGAC

CCTGAACTGAAAAAAAAAAAACACGAAGACTTCTCAAAAAACGTGTACCCTCAACTGCTG

AAAAGACTGTCAGAACTGTTCGAATCAAACAACGGTCACCTGACTTGGGGTGACTTCGTG

TTCGCTGGTATGTACGACTACCTGAAAATGATGATGCAATCACCTGACCTGGACGCTCAA

TACCCTATCTTCAAAAAAGTGGCTGACAACGTGTACTCACTGCCTCAACTGCAATCATAC

CTGGCTGCTGCTCCTAAAACTGACTTC

1. **>Contig1901**

ATGCCTATCAAAATCTACAAAGCTGACGTGTCACCTCCTGCTAGAGGTGCTCTGATGGCT

CTGGAACTGCTGGGTCTGAACTACGAAACTGAAGACCTGAACGTGATGAAAGGTGACACT

AGAACTCCTGAATACACTGCTCTGAACCCTGAACACACTATCCCTTTCTCAAAATACGGT

GGTGACAAAAGATCAACTCTGTACCCTTCAGCTGTGGAAGAAAAAGCTATCATCGACCAA

AGACTGTTCCTGGACGCTGGTAACATCTTCCCTAAATTCAGAGCTCTGGTGATGGAAGTG

GTGGTGGGTGCTAAAAGAAAAGACGTGGTGGAAAAAGCTATCGACGACGTGAAAGACGTG

CTGGGTATCCTGGAAAAATACCTGAACCAAAGAGAATTCCTGGCTGCTAACCACCTGACT

GTGGCTGACGTGTCACTGGTGGCTTCAATCTCAACTCTGGAAACTTTCATCTCACTGGAC

GAATTCCCTAAACTGAAAGCTTGGTTCGAAAAACTGAAAAAATACGACTGGTACCAAAAA

GCTAACGAACCTGGTCTGAAAATCATCAGAGGTTTCTTCAAAGTGGAAATCGACAAAATC

CTGAACCAA

1. **>Contig11878**

ATGTCACTGAAACTGTACCACTTCCCTATCTCAGGTCCTTCAAGAGGTGCTCTGCTGGCT

GCTAGAGCTATCGGTGTGCCTATCCAAATCCAAATCGTGAACCTGTTCAAAAAAGAACAA

CTGTCAGACTCATTCATCAAAATCAACCCTCAACACTGCGTGCCTACTCTGGAAGACGAC

GGTTTCGTGCTGTGGGAATCAAGAGCTATCGCTTGCTACCTGGTGGACAAATTCGCTAAA

GACGACCAACTGTACCCTAAAGACCTGGAAAAAAGAGCTCTGGTGAACCAAAGACTGTAC

TTCGACTCATCATCACTGTACGTGAAAATCAGAGCTATCTGCTTCCCTATCCTGTTCCTG

GGTGAAACTGAAATCAAAAAAAACCTGAAAGACGACCTGAACGGTACTCTGGTGTTCCTG

GACCAATTCCTGAACGACACTAAATGGGTGGCTGGTGACTCAATCACTATCGCTGACACT

TCAATCTACGCTTCACTGTCATCAATCCTGGCTGTGGGTTGGGACATCACTAAAGTGTCA

1. **>Contig8048**

ATGCTGGACACTTACCAACACAACACTGTGTTCGGTCAACTGCCTCTGCTGGAAATCGAC

GGTAAAAAATACGCTCAATCAGGTGCTATCGCTAGATACCTGGGTAGAAAATACGGTATC

GCTGGTTCATCAATCGAAGAAGACCTGGAAATCGACATGAACGTGGACTTCATCAACGAC

ATCAGAGCTAAAGCTGCTCTGGTGGCTTACGAAGCTGACCCTGAACTGAAAAAAAAAAAA

CACGAAGACTTCTCAAAAAACGTGTACCCTCAACTGCTGAAAAGACTGTCAGAACTGTTC

GAATCAAACAACGGTCACCTGACTTGGGGTGACTTCGTGTTCGCTGGTATGTACGACTAC

CTGAAAATGATGATGCAATCACCTGACCTGGACGCTCAATACCCTATCTTCAAAAAAGTG

GCTGACAACGTGTACAAACTGCCTCAACTGCAATCATACCTGGCTGCTGCTCCTAAAACT

GACTTC

1. **>Contig7006**

ATGGCTAAAAAACTGCACTACTTCCACCTGAACGGTATCGCTGAATCAATCAGATACATC

CTGCACTACGCTGGTGAAAAATTCGAAGACGTGAGATACGAAATCTCAGCTTGGCCTATC

CAATCAGTGAAAGAATCACTGCCTTACGGTCAACTGCCTCTGTACGAAGAAGGTGACAAA

TCACTGAACCAATCACTGGCTATCGCTAGATACCTGGCTAACAAATTCAACCTGCTGCCT

ATCGACCCTTGGCAACAAGCTGTGCTGGACGCTGTGGTGCTGAACATCTACGACTTCTGG

GGTAAAATCCTGCCTTACTTCAGAGAACAAGACCCTGTGAAAAAAGCTGCTATCAGAGAA

GAAATCATCAACGAAGTGATCCCTTTCTACTTCTCAAGATTCGAAAGAGAACTGAAAGAA

GCTAACGGTCACTTCGCTGGTAAACTGTCATGGGCTGACTTCATCCTGGTGGGTATCGTG

GAAGCTGCTAACCTGCTGCTGAAACACGTGATCGAAAAAGACTACCCTGTGGTGGAATCA

CTGATCAAAAAAGTGCAATCACTGCCTGGTGTGAAAGAATACATCGCTTCAAGAAAACCT

TACGCT

1. **>Contig3023**

ATGGTGATCAGACTGTACTACGACCTGATGTCACAACCTTCAAGAACTCTGTACATCCTG

TTCAAAACTATCAAATGCGACTGGGAACCTAAATACGTGAACCTGAGAAAAGGTGAACAC

TACTCAGACGACTTCACTAAAATCAACAAAATCCAAAGAGTGCCTGTGATCGACCACAAC

GGTTTCATCCTGTCAGAATCAGTGGCTATCGTGAAATACCTGTCAAGAGAAAACATCATC

CCTGAATCACTGTACCCTAGAGAATCAAAACTGAGAGCTAGAGTGGACGAATTCCTGGAA

TGGCACCACATCGGTCTGAGACTGCACTGCGCTATGTACTTCAGAATCAAATACATGGAC

CCTATCCTGTTCGGTAGAAAAACTGAAGCTAAACAAATCGCTGCTTACGAAGGTAGAATG

GTGAAAGCTCTGGAAGACTTCGACGAAAAATGGCTGGGTAGAGGTAACCAATACGTGGTG

GGTGACACTATCACTATCGCTGACCTGTTCGCTGCTACTGAACTGGAACAACCTAGAATG

GCTGGTTACGACCCTAAAGAACACTTCCCTGCTATCGGTACTTGGGCTAAAAAAGTGAGA

GAACACTTCAACCCTCACTACGACGAAGCTCACGTGGTGGTGAACAAAATCATCGAAAAA

CAAAGACAATCAAAACTG

1. **>Contig6716**

ATGGACCTGGAATTCATCGAACAAAGAGACCTGAACCCTGTGCTGAGAGAACAAGACACT

CCTGAAATGAGAAAAACTCCTACTTTCCTGGGTACTATGACTGAACTGACTAGAGAAACT

CTGGGTGGTATCAAAAACGCTTACTCAATGCTGGAAGCTTACCTGTCAACTTCAACTTAC

ATCGCTGGTGACTCAATGACTATCGCTGACATCTCAACTATCACTACTGTGTCAGCTCTG

GACGGTCTGCTGCCTGTGGACGAAATCAAA

1. **>Contig2596**

ATGTCACTGAAACTGTACCACTTCCCTATCGCTGTGCACCACGCTGTGCTGTACCTGCTG

CAAGAACAATCAGGTTTCAGATCAAAAACTAACTCAGAATTCATCAAAAAAGAACAACTG

TCAGACTCATTCATCAAAATCAACCCTCAACACTGCGTGCCTACTCTGGAAGACGACGGT

TTCGTGCTGTGGGAATCAAGAGCTATCGCTTGCTACCTGGTGGACAAATTCGCTAAAGAC

GACCAACTGTACCCTAAAGACCTGGAAAAAAGAGCTCTGGTGAACCAAAGACTGTACTTC

GACTCATCATCACTGTACGTGAAAATCAGAGCTATCTGCTTCCCTATCCTGTTCCTGGGT

GAAACTGAAATCAAAAAAAACCTGAAAGACGACCTGAACGGTACTCTGGTGTTCCTGGAC

CAATTCCTGAACGACACTAAATGGGTGGCTGGTGACTCAATCACTATCGCTGACACTTCA

ATCTACGCTTCACTGTCATCAATCCTGGCTGTGGGTTGGGACATCACTAAATTCCCTAAC

ATCCAAAGATGGTCAAGAATCGTGAACCTGCCTGGTTACGCTGAAAACGAAGAAGGTGCT

AAAGCTTTCGGTGAAGCTGTGAAAAAAAACCTGAAACAA

1. **>Contig12891**

ATGGCTGCTGTGGCTATCCCTACTCTGCTGAGAGGTTACAGAGAACCTACTTCACAACAA

ACTAGAGACATCACTGAAGCTTTCGAAGCTGTGGAAAGATACCTGAACCAAAGAAGATTC

CTGGCTGGTTCAAACGTGACTCTGGCTGACCTGGCTGTGGGTGCTACTGCTACTGCTACT

AACATCCTGCACCCTATCGACACTGACAAATACCCTAGATTCGCTGAATGGCTGAAAGAA

CTGAACGAAATGCCTTGCTTCAAATACATCAACGCTGAAGGTCTGTCATACCTGGAAAAA

TTCCTGGCTAAAGCTAAAGAAATGAAAGTGTCAAAATCAAAA

1. **>Contig2478**

TCATCACACGCTATCACTACTTACCTGATCACTAAATACGGTAAAGACGACTCACTGTAC

CCTTCAGACCCTAAAATCAGAGCTAGAATCGACCAAAGACTGCACTTCGACTCAGGTGTG

CTGTTCGCTACTCTGAGAAACGCTGTGGGTTCAATCCTGTACCAAGGTGAAACTACTCTG

ACTCCTGAACACTCAGAAAAAATCAAAAACGCTTACGAATTCACTGAAAAATTCCTGACT

TCACAATGGCTGGTGGGTGACCACGTGACTATCGCTGACATCTGCTGCGTGGCTACTATC

TCATCAATGAACGTGCTGCTGCCTATCGACGAAGGTGTGTACCCTAACCTGGCTGCTTGG

GTGGAAAGATGCTCAGCTCTGGACTCAACTCTGAAAGAAACTAACCTGGACCTGAACAAC

TCACTGAACTCACTGAAAGCTAACTGCGACAAATTC

1. **>Contig3291**

ATGTCAGACATCAGAGTGGAACTGCCTCCTGAACAAAGAAGATCATCATCAAGATTCGAA

ATCTACCTGTCAGAAAAATTCAACTACGGTGTGACTCTGGTGGAATCAGTGGCTATCATC

CAATACCTGGACGACACTAGACCTAAACCTGCTCTGTTCCCTCAATCACCTCTGCAAAAA

GCTAGAACTATCGTGTCAGGTATCCAACCTCTGCAAAACGTGGGTCTGAAATCATCATTC

GAAAGAGAAGAAGACTTCCAAAAATTCTCAAAATCATGGTGCGACAGAGGTCTGCAAACT

GTGGAAGAACTGCTGAAAGCTTCAGCTGGTTCATACTGCGTGGGTGACCAACTGACTCTG

GCTGACCTGTGCCTGGTGCCTCAAATCTTCAACGCTACTACT

1. **>Contig4841**

GGTGTGACTCTGGTGGAATCAGTGGCTATCATCCAATACCTGGACGACACTAGACCTAAA

CCTGCTCTGTTCCCTCAATCACCTCTGCAAAAAGCTAGAACTATCGTGTCAGGTATCCAA

CCTCTGCAAAACGTGGGTCTGAAATCATCATTCGAAAGAGAAGAAGACTTCCAAAAATTC

TCAAAATCATGGTGCGACAGAGGTCTGCAAACTGTGGAAGAACTGCTGAAAGCTTCAGCT

GGTTCATACTGCGTGGGTGACCAACTGACTCTGGCTGACCTGTGCCTGGTGCCTCAAATC

TTCAACGCTACTACTAGATACCTGAAC

**CORBOXYLESTERASE SEQUENCES**

1.

>Contig8193

ATGGGTCAAGAAGACTGCATGATCCTGAACATCTACACTCCTCTGAACATCCCTTCAGAC

AAACCTATCCCTGTGATGGTGTTCATCCACGGTGGTGGTTTCTTCCAAAGATCATCATCA

AGAATGATCTACGGTCCTGAATACCTGACTTCAAAAGGTGTGATCCTGGTGACTCTGAAC

TACAGACTGAACATCCAAGGTTTCCTGTGCCTGGCTCTGAAAACTCTGCTGGAAACTAGA

GACGAAAGACCTGGTTGCAGAACTGAAGTGGACAAAGAAAACATCAGAGCTTTCGGTGGT

AACCCTGAAAACGTGACTATCTTCGGTGAATCAGCTGGTGGTGCTTCAATCTCATACCAC

GTGATCTCACCTATGTCAAAAGGTCTGTTCCACAAAGCTATCATCCAATCAGGTTCATCA

CTGTCAGCTTGGGCTTTCCAATACAAACCTGTGTACGCTGCTTCACTGCTGGCTAAAACT

ATGGGTTTCGTGTCAAAAGACGCTCACGAACTGTACAACTTCCTGATCGCTCAATCAGAC

CACGACCTGATCGTGAACAGAGTGCCTAGATCAGAAAACAACACTATCCTGTCAGAAGTG

CTGTTCACTCCTTGCGCTGAACAAAACATCGAAGGTGTGGAACCTTTCCTGACTAACTCA

CCTTACGACTCATACATCAAAGGTGACTTCAACCAAGTGCCTGTGCTGATGGGTGCTAAC

ACTCAAGAAGGTCTGCTGTTCGCTGGTATGGACAGAGAAGACATGATCGTGAACGTGAAA

ATCGACAAAGCTTTCCAAAGAACTTGGATCTCAAGACTGATGAAACAAGAATGGCTG

**2**

>Contig9651

ATGGCTGCTCCTGCTCTGTTCCCTCCTAAAGTGACTTGGTCAGGTTCACCTCCTAAAGAC

CTGGCTTTCCTGTGCAACCACAGATCAGCTGTGAGACAATCACTGAAACCTCTGCTGCCT

GCTTGCGCTTGCGCTGAACTGCTGCTGCAAGACTGCGAAGTGGCTGCTAAAATCGAACCT

GGTTACGTGTGCGGTAGAAGAAGACTGGCTGCTGACGGTTCAGAATACGCTTCATTCAGA

GGTATCCCTTACGCTAAACAACCTCTGGGTAGACTGAGATTCCAAGTGGAAAGACCTTGG

TTCCTGTCACTGGACTCAAAAAACATCCCTGGTAACACTGCTGAAGGTTGCTCAGACAGA

GCTAGATGGCTGCAAAGAAACGCTAAATCATTCGGTGGTGACCCTGCTCAAGTGACTCTG

GCTGCTGAAAGAGGTAGATCACACGGTACTCACACTGTGCACATCACTTGCATCCAAAGA

ACTTTCCAAAAA

**3**

>Contig7738

ATGAAATACGAAAAAAGAATCGTGCTGTTCACTCTGTTCGCTCTGAACCTGATCGACCAA

CCTGCTCCTGAAGTGAAAATCGACCAAGGTATCCTGTCAGGTAAAATCTCAACTGACGGT

TCATACTTCGAATACACTGGTATCCCTTACGCTTCAACTAACTCAGGTAACAGATTCAAA

GCTCCTCTGCCTCCTCCTAAATGGGAAGGTGTGTACAAAGCTACTGACGAAATGCACTCA

TGCCCTCAACTGTTCATCTTCGGTATCGTGGTGGGTTCAGAAGACTGCCTGAAAATCAAC

GTGTACGTGCCTGCTAAATCAAAAGGTCCTCTGCCTGTGATGGTGTACATCCACGGTGGT

GCTTTCATCCTGGGTTCAGGTATCGAAGAAGCTCCTGGTAACGCTGGTCTGAAAGACCAA

ATCGCTGCTCTGAGATGGATCAAAAAAAACATCAGATCATTCGGTGGTGACCCTGACAAC

ATCACTATCTTCGGTGAATCAGCTGGTGGTACTTCAACTTCAATCCTGGTGGCTTCAGAA

ACTACTACTGGTCTGTTCAACAGAGCTATCGTGCAATCAGGTTCATCAATCGCTAACTGG

TCAATCAACAGAAAACCTGTGTGGGTGGCTTCACTGCTGACTAAAGCTCTGGGTTACGAC

ACTGAAGACCCTAACAAAATCTACCAAATCCTGTCAAAACTGCCTTACAGAGAACTGACT

TCACTGAACCCTGTGAAACCTCTGGGTAAATACTTCGACACTCTGCTGCTGCACCTGCCT

TGCGTGGAAAAATCATTCCCTGGTGTGGAACCTGTGCTGACTGACCTGCCTTACAACCTG

ATCAGAAAAAACTCAAGAAACATCCCTCTGATCTACGGTACTATGTCAAGAGAAGGTTAC

TTCCTGACTTCAATGGAAAACGAAACTACTCTGGAAGACAGAAACAACAGATACCTGTTC

GCTTCAGACCTGGTGTTCGAATCACAAACTGACGCTGAAATCACTGCTACTGCTATCAAA

AAATTCTACTTCGGTGAAGAAAGAGTGTCAAGAGAAAACACTCTGACTCTGGCTAAACTG

TACACTGAAATCTACTTCGAAATGCCTGCTATCCTGGAATCAGAAGCTTGCCTGGAACAC

ACTAACTCAACTGTGTACAACTACTACTTCGACTACTCAGGTAACAGAAACGTGCTGAAA

TCAAGAACTGGTTACAAACACGAAGAAGGTGCTTGCCACGGTGACGACGACATCTTCTAC

CTGTTCGACTCAAGACTGTGGCCTTTCCCTAAATCAAAAGAAGACATGAAAATGATCGAA

TGGGTGACTAAAATGTGGACTAACTTCGCTAAATACGGTGACCCTTCACCTGCTGCTGCT

AACGACCTGCCTGTGAAATGGATCCCTTCAAACAAAGACTCACTGAACTTCCTGCACATC

AAAGACAAAATGCAAATGGGTCCTATCCCTAACCCTGAATCATACAAAAACCTGGAAAGA

TACCTGTGCAAAATC

**4**

>Contig8714

ATGGAATACGGTAACATCGAACCTCCTTCACAACTGGTGAACCAAGAAAAAAACATCGTG

GCTGTGACTTTCAACTACAGACTGGGTCCTTTCGGTTTCCTGTGCCTGGGTACTCCTGAC

ATCCCTGGTAACGCTGGTATGAGAGACCAACTGGCTGCTCTGAAATGGGTGCAACAAAAC

GTGGCTGCTTTCGGTGGTAACCCTAACGAAGTGACTATCGCTGGTTGCTCAGCTGGTGGT

GCTTCAGTGGACCTGCACATCCTGGCTAAAGCTTCAAACGGTCTGTTCAACAAAGTGATC

ACTCAATCAGGTGCTAACATCGGTGCTTTCGCTGTGCAAACTGACCCTACTCTGAACGCT

AGAAACTACGCTAAAAGACTGGGTTACGCTGGTCCTGACACTCTGGAAGCTCTGGAAGAA

TTCTACAAAAACATCTCAAACGAAGAACTGCTGTCACTGACTCTGCACGACAACAAAAAC

GTGGAAATCACTATGGCTCCTTGCCTGGAAAGAGACATCGGTATCGAAAGATTCCTGGAA

GACACTCCTATCAACATCATCGAAAAAGGTGAATTCACTAGATACCCTATCCTGTACGGT

TGGGCTGACATGGAAGGTATCCTGAGAGTGTACTCATTCGACTCATGGAAAAACGAAATG

AACGAAGAATTCACTAAATTCCTGCCTTCAGACCTGAAATTCGAATCAGAAGAACAAAAA

CAAAGAATCGCTGACAAAGTGAAACAATTCTACTTCGACGGTGACGTGACTAACGACAGA

ATCCTGAACTACATCGACTACTCATCAGACGTGATGTTCAACGTGGCTATGCAAAGAGCT

GTGACTATGCAAGTGGAAGCTGGTAACAACGCTATGTACGTGGGTGTGTACTCATTCACT

GACAAAAACACTTCAATCATCCCTTACACTAACGAAAGAGGTGCTACTCACTGCGACCAA

ACTGTGGCTATCCTGGACCTGGACGAATCAACTCTGTCACCTGAATACCTGGAACTGAGA

AGAGCTTGGAGAGCTGTGTGGCTGAACTTCATCTCAACTGGTGACCCTACTCCTGACGGT

AACATCCCTCCTCCTTTCCCTAAATGGGAAGCTGCTAGAGCTAACAGAACTCCTTGCATG

GAAATCGGTAAAACTATCAGAGTGTACCCTGGTCCTTACGAACCTGAAAGACAACAACTG

TGGGACGAAATCTACGACCAACACAAAAGACACCCTATCGCTCCTTACTACTTCAACGAA

GAAGAATCAGTGGAC

**5**

>Contig8714

ATGCCTGGTCTGGACGTGTTCGCTTTCTACTCAATCCCTTACGCTAAAGCTCCTTCAGGT

AGAGACAAATACAAACCTCCTCTGCCTGCTCCTACTTGGACTACTCCTTTCGAAGCTACT

GAAAAAAACGTGCTGTGCCACCAATTCAACATCTTCCCTCCTGGTATGTTCGAATACGTG

GAAGACTGCCTGGTGGTGTCAGTGTACACTCCTGACACTAACGCTACTAACCTGCCTGTG

GTGGTGGTGATCCACGGTGGTGGTTTCCAATCAGACTTCGGTAACTACGAATCACCTTCA

CAACTGGTGGACCAAGGTAAAAACATCATCGGTGTGACTCTGAACTACAGACTGGGTCCT

ATCGGTTTCCTGTGCCTGGGTACTCCTGACGTGCCTGGTAACGCTGGTATGAGAGACCAA

CTGGCTGCTCTGAAATGGGTGCAACAAAACATCGCTGCTTTCGGTGGTAACCCTAACGAC

GTGACTATCGCTGGTTGCTCAGCTGGTGGTGCTTCAGTGGACCTGCACATGCTGTCAAAA

GCTTCAAGAGGTCTGTTCCACAAAATCATCGGTCAATCAGGTGGTAACATCGGTGCTTTC

GCTGTGCAAGTGGACCCTACTGCTTCAGCTAGAAAACACGCTAGAGACATCGGTTACACT

GGTCCTGACACTCTGGAAGGTGTGGAAGAATTCTACAAATCAATCCCTTACGAACAACTG

CTGTCAAAAGACCTGCACGAAAACAAAGACGTGTCAATCGTGATGGCTCCTTGCGTGGAA

AGAGACATCGGTATCGAAAGATTCCTGGAAGAAACTCCTATCTCAATCATCAAAAAAGGT

GACTACATCAGATACCCTGTGCTGTACGGTTGGGCTGCTATGGAAGGTATCTTCAGACTG

GAATACTTCGAAAACTGGAAAAAAGAAATGAACGAAGACTTCACTAACTTCATGCCTACT

GACCTGCAATTCAACAACGAAGAACACAAAAGACAAGTGTCAGACAAAGTGAAACAATTC

TACTTCGGTGGTAACGTGACTAACGACAACATCATCAGATTCGTGGACTACAACTCAGAC

GTGATGTTCAACGTGGCTATGCAAAGAGCTGTGACTATGCAAGTGGAAAACGGTCACAAC

GCTATCAACCCTACTCCTGACGGTAACATCCCTCCTCTGTTCCCTAAATGGGAAGCTGCT

TCAGCTAACAGAACTCCTTGCATGGACATCGGTAACCCTATCAGAATCTACCCTGGTCCT

TTCGACCTGGAAAGACAAAAATTCTGGGACGAAATCTACGACCAATACAAAAAAGAACCT

GTGCCTCCTTACGACTTCAACGTGACTAGAAAAATGAAAGTGTTCCTGTTCCTGCTGGCT

GCTCTGGTGGCTGTGGAAGCTCAAAGAATCCCTAAAGTGGTGCAAATCAAACAAGGTCTG

GTGAGAGGTTACTCATTCGACCACTACGACGCTTTCGCTTTCTTCGGTATCCCTTACGCT

ACTGCTCCTACTGGTGCTGAAAAATTCAAACCTCCTCTGCCTCCTCCTACTTGGAACGGT

ATCTTCGAAGCTGTGGACAGATACGTGTTCTGCCAACAACTGTCAATCTTCCCTGAAGGT

CAATTCAGATTCGTGGAAGACTGCCTGGCTATCAACGTGTTCGCTCCTAACACTAACCAA

ACTAACCTGCCTGTGGTGGTGGTGATCCACGGTGGTGGTTTCCAATTCGGTTTCGGTAAC

ACTGAAACTCCTGGTCAACTGATCACTGAAGAAAAAAACGTGATCGGTGTGACTTTCAAC

TACAGACTGGGTCCTCACGGTTTCCTGTGCCTGGGTACTCCTGACATCCCTGGTAACGCT

GGTATGAGAGACCAAATCGCTGCTCTGAAATGGGTGAAAGAAAACATCGCTGCTTTCGGT

GGTAACCCTAACGACATCACTATCGCTGGTTGCTCAGCTGGTGGTGCTTCAGTGGACCTG

CTGATGCTGTCAAAAGCTGCTGACGGTCTGTTCAACAAAGTGATCGGTCAATCAGGTGCT

AACATCGGTGCTTTCGCTGTGCAAGTGGACCCTACTGAAAACGCTAGATGGTACGCTAAA

TCAATCAACTACGACGGTCCTGACGACATCGAAGCTATCGAAGAATTCTACAAAAACATC

TCATACAAACAACTGTTCTCATACGACCTGAACGCTCAAAAAGACGTGCACGTGGTGATG

TCACCTTGCATGGAAAGAGACCTGGGTATCGAAAGATTCCTGGACGACACTCCTATCAAC

ATCCTGAAATCAGGTAACTACACTAGATACCCTGTGCTGTACGGTTGGGCTGGTATGGAA

GGTCTGTACAGAATCCAAAACTTCGACGACTGGATCATCCACATGAACCAAGACTTCACT

CAATTCATCCCTACTGACCTGAAATTCGAATCACCTGAACAAAAACAACAAATCGCTGAC

AAAGTGAAAGCTTTCTACTTCGGTGACACTCCTGTGAACAACTCAGACGTGCTGAACTAC

ATCGACTTCAACTCAGACGTGATGTTCAACGTGGCTATGCAAAGATCAGTGACTATGCAA

GTGGAAGCTGGTAACAACGCTATGTACGTGGGTGTGTACTCATTCACTGACAACAACACT

AACATCATCCCTCACACTAACGTGAGAGGTGCTAACCACTGCGAACAAACTAGAGCTGTG

TTCGACCTGAACGACAACACTCTGACTCCTGAATACATCGAACTGAGAACTGCTATGAGA

AAAATGTGGCTGAACTTCATCCACACTGGTGACCCTACTCCTGCTAACAACATCCCTCCT

CTGTTCCCTAAATGGGAACCTGCTGCTGCTAACAGAACTCCTTGCCTGGACATCGGTAGA

ACTATCCAAGTGCTGAACACTCCTTACGACGAAGAAAGACAAAGATTCTGGGACGACATC

TACGACAGATTCAAAAGAGAACCTGTGGCTCCTTACTCATTCTCATCAGCTTCATCACTG

GTGTACGGTGGTGTGCTGTTCTCATCATTCCTGGCTATCATCACTATGATCATCTCA

**6**

>Contig8459

ATGTCAGCTCTGCTGATGTCACTGGACTACAAAGGTGTGCTGTGCTGCTTCCTGGAACCT

GACTGCATCTTCGGTATCAACACTGACCCTCTGGTGCTGATCCAACAAGGTCTGGTGAGA

GGTCAAAGAGCTACTGACGGTGACTACACTACTTTCCTGGGTATCCCTTACGCTGTGGTG

GACGAAGACAACCCTTTCGGTCCTGCTACTCCTCACACTGCTTTCGAAGAACAAGTGTAC

AAAGCTTACAACGGTTCAATCGCTTGCCCTCAAACTCCTCTGTTCAACCACATCCCTGAA

GGTGGTGCTCAATCACTGGACTGCCTGAGACTGAACATCTACGTGCCTAACGAAGCTACT

TCAAAAGCTCCTCTGCCTGTGCTGGCTTGGATCCACGGTGGTCTGTTCGGTTACGGTTTC

GGTGGTGAATTCGGTGTGAGAGACCTGGTGAAACAAGGTATCCTGGTGGTGACTGTGAAC

TACAGACTGGGTCCTTACGGTTTCATGTGCCTGAACACTCCTACTATGCCTGGTAACCAA

GGTCTGAAAGACCAATTCATGGCTCTGAGATGGATCAGAAACAACATCGCTTCATTCGGT

GGTAACCCTTACAACGTGACTCTGGCTGGTCAATCAGCTGGTGCTTGCTCAGCTATCCTG

CACCTGTACTCATCAAGAGAAAAACTGTACCACAAAGTGATCGCTGAATCAGGTACTCCT

CTGAACCCTGGTCTGTTCGTGGACGGTGACGCTAACGCTGCTATCAAACTGGCTAACTAC

CTGGGTATGAACACTTCAGACACTGACTCAGCTCTGAAATTCCTGGCTACTTCACCTCAC

ACTCTGGTGACTGGTGCTGCTTCAGCTCTGAACATCCAATTCAGAGCTTGCAAAGAAAAA

TCATTCTCAGGTGTGGACCACTTCGTGGACTCAGACCCTTACTCAATGACTAACGAAAGA

AAAATCAGAAACACTCCTATCCTGATCGGTCACACTTCAAAAGAATCAATCAACACTATC

AAAAACTTCCACGACTACTTCAACAGAGACCCTTTCTTCGAAAACGTGGCTAACAACTTC

AACTTCGACTCAGACAAACTGAACAAAGTGGCTTCAACTATCAGACACTTCTACATCGGT

GACAAAGGTATCACTGAACAAGTGACTTCAAAACTGGAAGACTTCGAATCAGACTTCGTG

TTCAACCACCCTATCCAAGTGACTGCTATGAACCTGCTGAAAGAAAACGCTAACCCTGTG

TACGAATACATGTTCTCATACGTGGGTGTGAAAAGAGCTAAACCTCTGCTGGACTCAGAA

CTGCTGGTGCCTGTGGCTGAATTCGCTGGTGCTGTGCACGCTAGACAATACGAAGCTAAC

CAATACGTGTCAAACGTGTTCAGAATCGACCCTCTGGTGCTGATCCAACAAGGTCTGGTG

AGAGGTCAAAGAGCTACTGACGGTGACTACACTACTTTCCTGGGTATCCCTTACGCTGTG

GTGGACGAAGACAACCCTTTCGGTCCTGCTACTCCTCACACTGCTTTCGAAGAACAAGTG

TACAAAGCTTACAACGGTTCAATCGCTTGCCCTCAAACTCCTCTGTTCAACCACATCCCT

GAAGGTGGTGCTCAATCACTGGACTGCCTGAGACTGAACATCTACGTGCCTAACGAAGCT

ACTTCAAAAGCTCACTACAGATCATGGCACGGTTTCATGGCTGTGTACTCAGTGATGGAC

TCAGGTGAAAACTCAGTGAACAACATCGCTTCATTCGGTGGTAACCCTTACAACGTGACT

CTGGCTGGTCAATCAGCTGGTGCTTGCTCAGCTATCCTGCACCTGTACTCATCAAGAGAA

AAACTGTACCACAAAGTGATCGCTGAATCAGGTACTCCTCTGAACCCTGGTCTGTTCGTG

GACGGTGACGCTAACGCTGCTATCAAACTGGCTAACTACCTGGGTATGAACACTTCAGAC

ACTGACTCAGCTCTGAAATTCCTGCAACTGGCTCCTACTCTGGTGACTGGTGCTGCTTCA

GCTCTGAACATCCAATTCAGAGCTTGCAAAGAAAAATCATTCTCAGGTGTGGACCACTTC

GTGGACTCAGACCCTTACTCAATGACTAACGAAAGAAAAATCAGAAACACTCCTATCCTG

ATCGGTCACACTTCAAAAGAATCAATCAACACTATCAAAAACTTCCACGACTACTTCAAC

AGAGACCCTTTCTTCGAAAACGTGGCTAACAACTTCAACTTCGACTCAGACAAACTGAAC

AAAGTGGCTTCAACTATCAGACACTTCTACATCGGTGACAAAGGTATCACTGAACAAGTG

ACTTCAAAACTGGAAGACTTCGAATCAGACTTCGTGTTCAACCACCCTATCCAAGTGACT

GCTATGAACCTGCTGAAAGAAAACGCTAACCCTGTGTACGAATACATGTTCTCATACGTG

GGTGACTCAGGTGAAGAAGGTGCTGGTCACTCATCAGAACTGAACTACCTGTTCAACATG

CTGGGTAAAACTCAAAGAACTGCTGAAGACCAACTGATCGCTGACAGAATCACTTCACTG

TGGGCTAACTTCGTGAAATTCGGTAACCCTACTCCTAGACACAACAACCCTCTGTCAGTG

ACTTGGAACCCTATCTCAACTGTGACTAGACCTTACCTGGTGATCGACAAAGACATCAGA

CTGGAATCAAGAGTGTACAACGAAAGAATGGCTTTCTGGGAACTGTTCCACGACAACTAC

GGTACTTACAACAAACACAAAAGACAATGCACTATC

7

>Contig12636

ATGCACCTGTTCATCCTGCTGTCAGTGCTGGCTCTGGCTCCTCTGCCTGCTAAAGCTTGG

AAAGGTACTAGAAACGCTACTGAACACGGTCCTATCTGCATCCAATACGACGTGGTGACT

AAACACATCCTGCCTGGTTCAGAAGACTGCCTGTTCCTGAACGTGTACACTCCTAAACTG

AAACCTACTAAACCTCTGCCTGTGCTGTTCTTCATCCACGGTGGTGGTTGGAAATCAGGT

TCAGGTAACGACGACGAATACGGTCCTGACTTCCTGGTGCAATACGACATCGTGCTGATC

ACTATCAACTACAGACTGGACGTGTTCGGTTTCCTGACTATGGACAACGAAGACGTGCCT

GGTAACGCTGGTATGAAAGACCAAGTGCTGGCTCTGAAATGGGTGAACAACAACATCAAA

TACTTCGGTGGTGACCCTGAAAACATCACTATCATGGGTCAATCAGCTGGTTCAGCTTCA

GTGATCTACCACATGATGTCACCTATGTCAAAAGGTCTGTACAAAAGAGCTATCGCTATG

TCAGGTGTGCCTCTGTCAGAATGGGCTCAACCTTTCGAAGCTCCTAGAAGATCATTCTCA

CTGGGTACTATCCTGGGTAACCCTACTACTGACCCTAAAGAACTGATCAAATTCCTGAGA

AACGTGCCTGCTGAAAACCTGCTGAACACTAAACCTGCTGTGATCTCACAAGAAGAACTG

TGGATCAACCCTATCAAAATGTACGCTCTGGTGCCTGTGGTGGAAAGAGACTTCGGTCAA

GAAAGATTCCTGGTGGAAACTCCTACTGAATCAATCATCAACGGTAACGTGAGAGACCAA

GAACTGATCATCGGTCTGACTTCAATGGAATTCATCTACGCTATCCCTTGGCTGGAAGAC

GACCACGTGGCTAGATTCAACCTGGCTCTGGACCTGCTGGTGCCTCTGGACCTGCTGAGA

GAAATCACTCCTAAAGAAGTGGGTGCTCTGTCAGACGTGATCAGAAAAAGATACTTCGGT

GACAAACCTATCACTAACTCATCAATCGTGGGTTTCGTGCACCTGTTCCACGACACTCAA

TACGTGCACTCAACTAACAGATACCTGAAATACCTGCAACCTGGTAAATCAGACAAATAC

TTCTACAACTTCTTCACTGTGTCAGGTAGAAACCTGTACACTAAATACGGTGACAAATAC

GGTATCCACGGTTGCGGTCACACTGACGACCTGAGATACCTGTTCGACGCTAAATCATCA

AAAACTTCAGTGGACCCTAACTCAATCGAATACAGACTGATCAAACAAACTTGCACTCTG

TTCACTAACTTCGTGAAATACGGTGAAGACTGCCTGTTCCTGAACGTGTACACTCCTTGG

ATCAACCCTAAATCACCTCTGCCTGTGCTGTTCTTCATCCACGGTGGTGCTTGGAAATCA

GGTTCAGGTAACGACGACAACTACGGTCCTGACTTCCTGATGCACTACGACATCATCCTG

GTGACTATCAACTACAGACTGGACGTGCTGGGTTTCCTGTCACTGGACACTAAAGACGTG

CCTGGTAACGCTGGTATGAAAGACCAAGTGCTGGCTCTGAAATGGGTGCACGACAACATC

GGTAACTTCGGTGGTGACCCTAACCAAGTGACTATCATGGGTCAATCAGCTGGTGGTGCT

TCAGTGATCTACCACATGATGTCAACTGTGTCAAGAGGTCTGTTCAAAAGAGCTATCTCA

ATGTCAGGTGTGCCTCTGTCAGACTGGGCTTTCCCTTTCGAACAAACTACTAGAGCTTAC

GAACTGGCTAAAACTATGGGTCAAAACATCGACAACTCAGAAGACCTGCTGAACTACCTG

CAAAACGTGCCTGCTCACGACCTGCTGAACACTAAACCTGCTGTGCTGTCATCAGAAGTG

TACTGGAACAACGGTATCAAAATGTTCTCATTCGTGCCTGTGATCGAAAAAGACTTCGGT

AAAGAACAATTCCTGGTGGAACCTATCAAAGAAGCTCTGGTGAACGGTAACATCGAAGAC

ACTGACCTGCTGATCGGTTACACTGACCTGGAATACCTGATCGCTGTGCCTCTGCTGGAA

TCATACGGTCTGGCTGACTACAACATGTTCAAAGAACTGCTGGTGCCTAGAGAAATCCTG

CTGGAAATCTCACCTAAAAGAATGCTGTCACTGGCTGAAAAAATCCACAAAGCTTACTTC

GGTAACCAATTCATCAACAACACTACTATGCTGAACTTCCAAAACTACGGTGGTGACCAC

ATCTTCATCAACTCAGTGCTGAGATTCCTGAGATACCTGCCTAAAGGTAGAGCTAAAAGA

TACCTGTACAAATTCTTCACTGAATCATCAAGAAACAGATACCTGAAAAACGCTGAAAAA

TACAACATCCACGGTTGCGCTCACACTGACGACCTGATGTACCTGTTCGACGCTAAACAA

GAAGCTACTGCTATGAACGTGGACTCAGACGAATACCAACTGATCAACCAAACTTGCACT

CTGTTCACTAACTTCGTGAAATACGGTAACCCTACTCCTGACGACGCTCTGGGTGTGACT

TGGCCTGAATACAACAACGACGACAGACCTTACCTGTCAATCAACAACAAACTGACTACT

GGTACTCACCTGGACTTCGACGTGACTAACTTCTGGGACGACATCTACAGATCATGCGAC

ATCGAACTGCCT

8

>Contig10354

CTGAACGCTCTGGGTTTCCTGTCACTGGACTCAAAAAACATCCCTGGTAACAACGGTCTG

AGAGACGCTCTGACTGCTCTGAGATGGCTGCAAAGAAACGCTAAATCATTCGGTGGTGAC

CCTGCTCAAGTGACTCTGGGTGGTCAATCAGCTGGTGGTGTGATGGCTCACATCCTGTCA

ATCTCACCTGCTTCAAAAGGTCTGTTCAAAAGA

9

>Contig4304

ATGCCTGGTCTGGACGTGTTCGCTTTCTACTCAATCCCTTACGCTAAAGCTCCTTCAGGT

AGAGACAAATACAAACCTCCTCTGCCTGCTCCTACTTGGACTACTCCTTTCGAAGCTACT

GAAAAAAACGTGCTGTGCCACCAATTCAACATCTTCCCTCCTGGTATGTTCGAATACGTG

GAAGACTGCCTGGTGGTGTCAGTGTACACTCCTGACACTAACGCTACTAACCTGCCTGTG

GTGGTGGTGATCCACGGTGGTGGTTTCCAATCAGACTTCGGTAACTACGAATCACCTTCA

CAACTGGTGGACCAAGGTAAAAACATCATCGGTGTGACTCTGAACTACAGACTGGGTCCT

ATCGGTTTCCTGTGCCTGGGTACTCCTGACGTGCCTGGTAACGCTGGTATGAGAGACCAA

CTGGCTGCTCTGAAATGGGTGCAACAAAACATCGCTGCTTTCGGTGGTAACCCTAACGAC

GTGACTATCGCTGGTTGCTCAGCTGGTGGTGCTTCAGTGGACCTGCACATGCTGTCAAAA

GCTTCAAGAGGTCTGTTCCACAAAATCATCGGTCAATCAGGTGGTAACATCGGTGCTTTC

GCTGTGCAAGTGGACCCTACTGCTAGAGTGGAAGAATTCTACAAATCAATCCCTTACGAA

CAACTGCTGTCAAAAGACCTGCACGAAAACAAAGACGTGTCAATCGTGATGGCTCCTTGC

GTGGAAAGAGACATCGGTATCGAAAGATTCCTGGAAGAAACTCCTATCTCAATCATCAAA

AAAGGTGACTACATCAGATACCCTGTGCTGTACGGTTGGGCTGCTATGGAAGGTATCTTC

AGACTGGAATACTTCGAAAACTGGAAAAAAGAAATGAACGAAGACTTCACTAACTTCATG

CCTACTGACCTGCAATTCAACAACGAAGAACACAAAAGACAAGTGTCAGACAAAGTGAAA

CAATTCTACTTCGGTGGTAACGTGACTAACGACAACATCATCAGATTCGTGGACTACAAC

TCAGACGTGATGTTCAACGTGGCTATGCAACAAATCCCTCCTGACGGTAACATCCCTCCT

CTGTTCCCTAAATGGGAAGCTGCTTCAGCTAACAGAACTCCTTGCATGGACATCGGTAAC

CCTATCAGAATCTACCCTGGTCCTTTCGACCTGGAAAGACAAAAATTCTGGGACGAAATC

TACGACCAATACAAAAAAGAACCTGTGCCTCCTTACGACTTCAACGTGACTAGAAAAATG

AAAGTGTTCCTGTTCCTGCTGGCTGCTCTGGTGGCTGTGGAAGCTCAAAGAATCCCTAAA

GTGGTGCAAATCAAACAAGGTCTGGTGAGAGGTTACTCATTCGACCACTACGACGCTTTC

GCTTTCTTCGGTATCCCTTACGCTACTGCTCCTACTGGTGCTGAAAAATTCAAACCTCCT

CTGCCTCCTCCTACTTGGAACGGTATCTTCGAAGCTGTGGACAGATACGTGTTCTGCCAA

CAACTGTCAATCTTCCCTGAAGGTCAATTCAGATTCGTGGAAGACTGCCTGGCTATCAAC

GTGTTCGCTCCTAACACTAACCAAACTAACCTGCCTGTGGTGGTGGTGATCCACGGTGGT

GGTTTCCAATTCGGTTTCGGTAACACTGAAACTCCTGGTCAACTGATCACTGAAGAAAAA

AACGTGATCGGTGTGACTTTCAACTACAGACTGGGTCCTCACGGTTTCCTGTGCCTGGGT

ACTCCTGACATCCCTGGTAACGCTGGTATGAGAGACCAAATCGCTGCTCTGAAATGGGTG

AAAGAAAACATCGCTGCTTTCGGTGGTAACCCTAACGACATCACTATCGCTGGTTGCTCA

GCTGGTGGTGCTTCAGTGGACCTGCTGATGCTGTCAAAAGCTGCTGACGGTCTGTTCAAC

AAAGTGATCGGTCAATCAGGTGCTAACATCGGTGCTTTCGCTGTGCAAGTGGACCCTACT

GAAAACGCTAGATGGTACGCTAAATCAATCAACTACGACGGTCCTGACGACATCGAAGCT

ATCGAAGAATTCTACAAAAACATCTCATACAAACAACTGTTCTCATACGACCTGAACGCT

CAAAAAGACGTGCACGTGGTGATGTCACCTTGCATGGAAAGAGACCTGGGTATCGAAAGA

TTCCTGGACGACACTCCTATCAACATCCTGAAATCAGGTAACTACACTAGATACCCTGTG

CTGTACGGTTGGGCTGGTATGGAAGGTCTGTACAGAATCCAAAACTTCGACGACTGGATC

ATCCACATGAACCAAGACTTCACTCAATTCATCCCTACTGACCTGAAATTCGAATCACCT

GAACAAAAACAACAAATCGCTGACAAAGTGAAAGCTTTCTACTTCGGTGACACTCCTGTG

AACAACTCAGACGTGCTGAACTACATCGACTTCAACTCAGACGTGATGTTCAACGTGGCT

ATGCAAAGATCAGTGACTATGCAAGTGGAAGCTGGTAACAACGCTATGTACGTGGGTGTG

TACTCATTCACTGACAACAACACTAACATCATCCCTCACACTAACGTGAGAGGTGCTAAC

CACTGCGAACAAACTAGAGCTGTGTTCGACCTGAACGACAACACTCTGACTCCTGAATAC

ATCGAACTGAGAACTGCTATGAGAAAAATGTGGCTGAACTTCATCCACACTGGTGACCCT

ACTCCTGCTAACAACATCCCTCCTCTGTTCCCTAAATGGGAACCTGCTGCTGCTAACAGA

ACTCCTTGCCTGGACATCGGTAGAACTATCCAAGTGCTGAACACTCCTTACGACGAAGAA

AGACAAAGATTCTGGGACGACATCTACGACAGATTCAAAAGAGAACCTGTGGCTCCTTAC

TCATTCTCATCAGCTTCATCACTGGTGTACGGTGGTGTGCTGTTCTCATCATTCCTGGCT

ATCATCACTATGATCATCTCA

10

>Contig11996

ATGAACTGCATGGACACTTGCGTGATCGAAACTACTGACGGTCCTGTGAGAGGTTACATC

GAAAAATCAGAAGGTACTTACTACAAATTCAAAAAAGTGCCTTACGCTAAACCTCCTATC

GGTCCTCTGAGATTCAGACCTCCTTGCCCTGTGACTCCTTGGAAAGAAGAACTGAACTGC

ACTGAAGACGCTCCTCTGCCTCTGCAATGCAGAGGTTCAAAACTGGTGGGTTCAGAAGAC

TGCCTGTACATCGAACTGATCACTCCTGAAATCGTGCAAGACCACCCTCTGCCTGTGATG

TTCTGGATCGGTACTTACGTGTTCTCATCATCAATCGACAACCTGCTGGACCCTACTCTG

CTGATCGACAACGGTGTGATCTTCGTGAGATGCGGTTTCAGACTGGGTCCTTTCGGTTTC

CTGTCAATCAACGAATTCACTGCTCCTGGTAACTGCGGTCTGAAAGACATCGTGATGGCT

CTGAAATGGGTGCAAAACAACATCAACAAATTCGGTGGTGACCCTAACAACGTGACTATC

TTCGGTTCATCAACTGGTGGTTCAGCTGCTCACCTGCTGATGCTGTCACCTATGGCTTCA

GGTCTGTTCCACAAATGCATCATCCAATCATCATCAGCTTTCAACAACTGGTCACTGGCT

AAAAACCCTTCACAACCTGTGATGGAACTGGCTGAAAAACTGGGTATCAAAAAATCACAC

AAAGTGGAAGTGGTGGAAGAACTGAGAACTCTGTCAGCTGACAAAATCATGCTGGCTTTC

AGAGCTCTGGAACACGACAACAAAATCGTGGACAACAGAGACATGTTCCAAGCTTACTTC

AAACCTTGCATCGAAGAAGACTTCGAAGGTCTGCCTGCTTTCCTGACTAAATCACCTCTG

CCTATCCTGAAATCAGGTAACTTCAACAGAGTGCCTCTGATCATCGGTTCAAACAACATC

GAAGGTTCAGTGCTGCAATACTACAACTCAGACTTCTACGCTGACTTCCAAAAATACAAC

GACAACGTGTGCCTGCTGGTGCCTAGATCACTGTCAAGATACGACACTAACGTGTCAAAA

AACATCGGTCACCAACTGCTGAGATTCTACGTGGGTGAAAACGAAAAACTGACTGAAAAC

ACTAAATCACAATACCTGCAAATGATCTCAGACTACTACTTCCTGTACTACATCAACAAA

ACTGTGAGACTGCACTCAGAATTCGCTCCTGAATGCGCTGTGTACTACTACATCGTGAAC

TGCGCTGGTGAATGGAACGTGCCTAAATCACTGTCATTCTTCAACACTCTGGGTCACTCA

ACTGAAATCCCTTACATCTTCAGAATCAAAACTCTGGAAACTCCTGAAATGAACTCAATG

GAATCATCAACTTACTCAGGTACTAGAGACTCAATCATCACTAGAACTAGAGTGGTGAAA

ATGTGGACTAACTTCGCTAAATACGGTAACCCTACTCCTGACGAAAACGACCAACTGCTG

CAAATCAAATGGGACCCTGTGGAACACAAAAACAAACTGAACTACCTGTCAATCGGTTCA

GAACTGACTAAAGGTAGAAACCCTTTCTACGAAAGAATGCAATTCTGGGAAGAACTGCAC

AAAAAATACTCACTGCTGAGAATGCTGGTGTACTTCAACGACATCGGTGTGTCATGG

11

>Contig5133

CCTGCTTCAAGAAGACTGGAACCTGTGGAAGTGTTCTGGGGTGTGCCTTACGCTGGTAGA

CCTCCTAGACTGGGTCCTCCTCCTCCTGCTCCTTCATGGCCTGGTACTAGACTGGCTGAC

TCATTCGCTCCTATCCAAGACGACGCTGGTGACCTGTCAGGTATCTCACAATACTGCTAC

AGAACTGCTGCTCTGTCAAAAATGCCTCTGGGTCTGTACAACGAACTGAAAGCTACTATC

CCTTTCCTGGCTAACCAATCAGAAGACTGCCTGTGCCTGAACATCTACGTGCCTGGTTCA

GGTGCTAGAGGTGTGGAAGCTCCTTACGCTGTGGTGGTGTGGGCTGGTGGTGCTTCACAC

GAATGGGGTTCAGGTAACGTGCTGGACGGTGCTTCATACATCTACTGCACTCTGTGCATG

TGCGACGACAAATTCGGTTCACAAACTGTGTCACTGGGTGCTTCACAATTCAACAGAAAC

TGCTACAGAATCGGTCTGGTGTCAAGAGTGCTGCTGCTGTCAGGTTCAGCTCTGTCACCT

ACTGCTCTGGCTCCTGACCCTGCTCTGGCTAGAGACCACGCTGCTCAAGCTCTGAGATGC

TCACCTGACGGTTCAAACGACGAAAACTGGCTGGCTGCTTGCGTGTCATCAAGAGCTCTG

CCTCTGCTGCTGGCTGTGGAAGCTCCTAGAGCTAGATTCCTGTCAGGTTGGGCTCCTGCT

GCTCCTGCTCCTGCTAGAGCTCTGCACGCTTCAGACGCTTTCCTGGACTGCGCTCTGGCT

GTGGTGGTGGCTACTACTGAATCATACCAAAACGTGTACAGATACCACAGAAACGAAATC

TTCGCTGCTGTGAGAAACGAATACACTGACTGGGAAAAACCTATCCAACACCCTATCAAC

ATCAGAGACGCTACTCTGGAATCACTGTCAGACGCTGCTGTGGCTGCTCCTGCTCTGAAA

AGACTGGGTTCAGTGACTGGTGAAACTCTGCCTTACTTCCTGGGTCTGCCTCTGGTGGGT

GGTACTACTTCAAACCCTAGAAACTACTCAAGAGGTGACGTGACTGTGGCTGAATCAGCT

GTGGCTCTGCTGGCTGCTTTCGCTAAAACTGGTGACCCTTCACCTAGAGGTGACGAAAGA

CACCACGAAGGTATGACTTGGCCTAGATACGAACTGAACACTCAACAATACCTGTCAATC

TCAACTAAAGTGAGAGTGAAATCACACTACAGAGGTCACAAAATGGCTCTGTGGCTGCAC

CTGGTGCCTCAACTGCACAGACCTGGTGCTGCTCCTAGACACCACCAATTCAGATCAGTG

CACCCTGACATGTTCGCTGGTCAA

12

>Contig9046

ATGCTGATGCTGACTATCGTGTACTTCATCACTCTGCCTGCTCTGACTTCAAAACTGGTG

AGAGGTGAAGCTACTATGGACGAAAAACAACAACAACCTATGATCTCATCAAGAGTGGTG

AGAACTAAATACGGTGACATCAGAGGTTTCATCGTGACTCCTGAATCAAGATTCCTGGAA

CCTGTGGAAGTGTTCAGAGGTGTGCCTTACGCTTCACCTCCTGTGGGTTCACTGAGATTC

ATGCCTCCTGTGTCAGGTGCTCAATGGTCAGGTGTGAAAATCGCTGAAGAATTCTCACCT

GTGTGCCCTCAAGTGCTGCCTGACATCAGAAACGAAACTGCTGTGCTGAAAAGAATCTCA

AAAGGTAGACTGGAATACCTGAAAAGAATCCTGCCTTTCCTGACTAACCAATCAGAAGAC

TGCCTGTACCTGAACATCTACGCTCCTGCTCAAGGTAAA

13

>Contig11486

ATGACTGAAGACTGCCTGGTGGCTAACGTGTACGTGCCTATCTCACAAGAAACTCACCTG

CCTGTGATCGTGTTCGTGCACGGTGGTGCTTACATCGGTGGTTCAGCTCTGAACGACTCA

CCTAAAAACCTGGTGAACACTCAAAAAGTGATCGCTGTGAACTTCAACTACAGACTGAAC

GTGCACGGTTTCCTGTGCCTGGGTACTGAAGACGCTCCTGGTAACGCTGGTATGAAAGAC

CAAGTGGCTCTGCTGAGATGGGTGAAAGAAAACATCGCTTCATTCGGTGGTAACCCTAAC

GACGTGACTATCACTGGTTGCTCAGCTGGTGGTTCAGCTGTGGACCTGCTGATGATCTCA

AAAATCACTGACGGTCTGTTCAACAAAGTGATCTCAAACTCAGGTTGCGGTATCTCAACT

TTCGGTACTCAACTGAACCCTCTGGAAAACGCTAAACTGCACGCTTCACTGCTGAACGCT

ACTAACGTGGACGACATCAAATCACTGGCTGAATTCTACAAAACTCACCCTCTGTCAGAA

CTGATCCAAGAAAGAGACCTGGGTCAAGAAATGTTCCTGGACGACGCTCCTATCAACATC

TACAAAAAAGGTGACTACAAAAAACTGCCTGTGGTGTACGGTTACGCTAACATGGAAGGT

CTGTTCAGACTGCCTCTGTTCTACCTGTGGAAAGACCTGATGAACGAAAAATTCTCAGAC

TTCCTGCCTGCTGACCTGCAATTCGAATCAGACGCTCAAAAAGAAGAAGTGGCTCTGTCA

ATCAAAAACTTCTACTTCGGTGACAAACCTGTGAACGAATCAACTATCCTGCAATACATC

GAACTGCACACTGACCTGCTGTTCCTGAACTCAATCTACAGAACTGTGACTCTGCAAGTG

CAAGCTGGTAACAACCAAGTGTACCTGTACGACTACTCATTCGTGACTAACTCAACTGCT

GTGGTGCCTCACACTGACTCAGTGAGAGGTGTGGACCACTGCTTCCAAACTGTGGGTCTG

ATGGACGTGCCTCCTGCTTACGAAGACCTGGTGGACGACGAATCACTGTACATGGCTAAC

ATCACTAGACAACTGTGGATCAACTTCATCACTACTGGTAACCCTACTCCTCCTGGTCAC

GAATCATCAGTGCCTACTTGGCTGCCTGCTGGTGAAAACAGATCACCTCACCTGTCAATC

GGTCAAGTGATCCAACTGGCTGGTGTGCCTGCTGAAAACAGATCACTGTTCTGGGACGGT

ATCTACGAAAGACACTACAGAATGCCTATGCCTCCTCCTAACCACTCAGGTGCTGCTTCA

GCTTACCTGATGTCACTGAACACTTCAGGTCCTCTGTCAGCTTCATCATGGGTGAACGTG

TACGACGCTGTGGACAAAGGTATCATCTGCCCTCAACCTACTATCAACAACCTGACTATC

GGTGGTTCAGTGAAACTGGTGGCTGACGAAAACTGCCTGATCGCTAACATCTACGTGCCT

GACACTGAAGAAAAAGACCTGCCTGTGATCGTGTACGTGCACGGTGGTGCTTACCAAGTG

GGTTTCGGTAACATCCTGCCTCCTAAAAACCTGGTGAAATCAGGTAAAGTGATCGCTGTG

ACTTTCAACTACAGACTGGGTATCCACGGTTTCCTGTGCCTGGGTACTACTGACGCTCCT

GGTAACGCTGGTATGAAAGACCAAGTGGCTCTGCTGAGATGGGTGAAAAACAACATCGCT

TCATACGGTGGTAACCCTGGTGACGTGACTATCGCTGGTTACTCAGCTGGTTCATCAGCT

GTGGACCTGCTGATGCTGTCACCTTCAGCTCAAGGTCTGTTCAACAAAGTGATCCCTGAA

TCAGGTGCTAACATCTCACCTTGGTCAGTGCAAGTGGACCCTATCGAAAACGCTAAAGAA

TTCGCTAGATCAGCTGGTCTGGAAGACACTGACGACCTGTACGCTCTGGAAGAATTCTAC

AAAACTGCTTCATTCAAACTGTTCACTTCAACTGGTGGTTTCTTCAACAGAACTGACGCT

AACTTCCTGTTCACTCCTTGCGTGGAAAGAGAAACTGGTGACGGTGCTTTCCTGACTGAC

GCTCCTTACAACATCCTGAAAAGAGGTGACTACAGAAAAGTGCCTGTGCTGTACGGTTTC

GCTAACATGGAAGGTCTGCTGAGAATCCCTGAATTCGACACTTGGAAAGACAAAATGAAC

GCTAACTTCTCAGCTTTCCTGCCTGCTGACCTGAAATTCGTGAACACTGAAGAAAGAAAC

GAAGTGTCAAAAAAAATCAAAGAATTCTACTTCTCAGACATGCCTGTGTCAGAAGACAAC

ATCCTGTCATACATCGACTTCCAATCAGACGTGTACTTCGGTTACTCATCACTGAAAGCT

GTGAGACTGCACGTGCAAACTGGTCACGACAAAATCTACCTGTACGAATACGACTACGTG

GACGAATCAACTCCTGCTATCCCTCACACTGACAACGTGAGAGGTGCTAACCACTGCGCT

CAAACTATGGCTGTGCTGCAACCTGAAGACACTCTGTCAGAAGACTCAAAAACTGTGTCA

AGAACTATGCAAGAAATCTGGCTGAACTTCATCACTACTGGTTCACCTGTGCCTGTGGGT

TCATCACTGCCTGCTTGGCCTCCTGCTGGTGCTGACGGTTCACCTCACATGAGACTGGGT

AGAACTGTGGAACTGAGAGGTGCTCTGCTGGCTGACAGAGTGAGATTCTGGGACTCAATC

TACGACCACCAACCTAGAGCTGCTCCTCCTCCTAGAAGACCTCTGATCATCCCTAACTAC

AAATACGAAAACAGAGTGGTGATGCTGAGAATCAACGCTACTTACGTGCTGTACTTCTAC

GACGTGGAATCAAAAAAAAGAAAAAAAAGAATCTACCCTCTGTCATGCATGTTCACTGCT

GTGCTGATCAAACTGGACCTGGTGTCACTGGACTTCTTCGCTTGGGGTACTAAAGACGTG

CCTGGTAACGCTGGTATGAAAGACCAAGTGGCTCTGCTGAGATGGGTGAACCACAACATC

GGTTCATACGGTGGTAACCCTGACGACGTGACTATCGCTGGTTCATCAGCTGGTTCATCA

TCAGTGGACCTGCTGATGCTGTCACCTTCAGCTCAAGGTCTGTTCAACAAAGTGATCCCT

GAATCAGGTGCTAACATCGCTTCATGGTCAGTGCAAATCGACCCTATCGCTAACGCTAAA

GAATTCGCTAGATCAGTGGGTTTCGAAGCTACTGACGACATCTACGCTCTGGAAGAATTC

TACAAAAAAGCTTCACTGGAACTGCTGACTTCAACTGGTGCTTTCTTCGACAGAACTTAC

GCTTCATTCGTGTTCTCACCTTGCGTGGAACAAGAAACTGTGGACGACGCTTTCCTGACT

GACGCTCCTTACAACATCCTGAAATCAGGTAACTACAGAAAATACCCTCTGCTGTACGGT

TTCGCTAACATGGAAGGTCTGCTGAGAATCGGTCAATTCTCAGCTTGGAAAGACAAAATG

AACGCTAACTTCTCAAACTTCCTGCCTATCGACCTGAAATTCGAATCACTGGAACAAAGA

AACGAAGTGTCAAAAAAAGTGAAAGAATTCTACTTCGGTGACAAACCTGTGGTGAAAAAC

AACGAACTGTCAGAAGACTTCAAAAACATCTCAAACATGATGCTGGACATCTGGATCAAC

TTCATGACTACTGGTTCACCTGTGCCTGAAGGTTCATCACTGCCTGCTTGGCCTCCTGCT

GGTGCTGGTGGTTCACCTCACATGAGACTGGGTAGAACTGTGGAACTGAGAGGTGCTCTG

CTGGCTGACAGAGTGAGATTCTGGGACTCAATCTACGACAGACACTACAGAGTGCCTGCT

CCTCCTCCTCCTCCTCCTGCTCAACACACTGAATTC

14

>Contig6379

ATGGTGTACATCCACGGTGGTTACTTCCAAATCGGTTTCGCTGGTAGATACCAATACGGT

CCTAAATACCTGGTGAAACACGACGTGATCCTGGTGACTCTGAACTACAGACTGGGTCCT

TACGGTTTCATGTGCCTGGACATCCCTGAAGTGCCTGGTAACCAAGGTCTGAAAGACCAA

ATCCTGGCTCTGAAATGGATCAAAGCTAACATCGACGCTTTCGGTGGTGACTCAGACAAA

ATCACTATCTTCGGTGTGTCAGCTGGTGCTCACTCAATCCACTTCCACCTGACTTACGGT

GACGAAACTCTGTTCAAATCAGCTATCCTGCAATCAGGTTCAATGTTCTCATCAACTGTG

ATCTCAGACCCTGTGAAAAACGCTCCTCAAAGAATCTCAACTTACCTGGGTTACTCAACT

GAATCAGCTACTGAAGCTATCTCACTGCTGGCTGTGACTGACCCTAAAATCATCGTGAAA

TCAGTGATCGACATGAACCTGGAATTCAAACCTTGCGTGGAAAAATACTTCGACAACGGT

CAACCTTTCATCGCTACTACTTGGATCAACAACATCAAACCTAAAGTGGCTTCAAAACCT

ATCATGATCGGTTTCTCAGAACTGGAACTGTTCGGTTCATTCACTGACCCTGACATCAAC

GTGTACAAATCAATCTTCAGATCAAACCTGAAACTGACTTTCAACTTCAACGACGAATAC

CTGAACCAAATGGAAGAACTGGTGAGAGAATTCTACCTGGACGACGAAATCAAATACAAA

GACACTAGAAAAGCTCTGATCAACTTCCACTCAGACTTCACTTACATCTACCCTACTCAC

AAATCAAACGTGCTGGCTGAACCTATCGTGAAAACTAAACTGGGTCTGATCAGAGGTATC

AACGCTGTGGACGGTGACTACTCAATGTACATGGGTATCCCTTTCGCTGGTCAACTGTCA

AACCCTTTCGGTGCTGCTCTGCCTCAAGAACCTTTCGACGGTATCTTCGACGCTTTCGAC

GACTCAGCTATCTGCCCTCAAATCGAAGAATTCAACAACACTATCGTGGGTTCACTGGAC

TGCCTGCAAATCAACATCTACGTGCCTAACAACGCTTCACCTTCAAACCCTGTGCCTGTG

ATGGCTATGATCTTCGGTGGTGCTTTCACTCTGGGTTTCCCTGGTAGATTCCTGTACGGT

CCTAAATACCTGGTGAGACACGACGTGATCCTGGTGACTTTCAACTACAGACTGGGTCCT

TACGGTTTCATGTGCCTGCAAACTCCTGAAGTGCCTGGTAACACTGGTCTGAAAGACCAA

CTGCTGGCTCTGAGATGGATCAAAGAAAACATCGGTGCTTTCGGTGGTGACTCAGGTAAC

GTGACTATCCTGGGTCACTCAGCTGGTGGTTTCTCAGTGGACTACCACCTGTACTCACCT

CTGGAAAAACTGTACAACAAAGTGATCCTGCAATCAGGTACTGCTCTGTCATCATCACCT

TCAACTCCTATCAGAAACGCTCCTGTGCTGCTGGCTAACCACCTGGGTTTCGACACTGAC

GACATCAACGAAGCTATCTCACTGCTGGCTCAAATCGAACCTAACCTGATCATCGCTGCT

ACTAAAGAACTGGGTCTGAAATTCCTGCCTTGCATCGAAAAAGAATTCGAAGGTGTGGAA

TCATTCTTCACTACTGACTGGATGAGAATGGACATCCCTAAAAAAGCTATCCCTCAAAAA

CCTTTCAACGACATCTTCGAAGCTCTGGACGACACTGCTATCTGCCCTCAAATCGAAGAA

TTCAACAAAACTTTCGCTGGTACTCTGGACTGCCTGCACCTGAACGTGTACGTGCCTACT

AAAGCTAACATGAACAACCCTAAAGCTGTGCTGGTGTGGATCTACGGTGGTGGTTTCGAA

ATCGGTTTCTCAGGTAGATACCTGTACGGTCCTAAATTCTTCGTGAGACACGACATCATC

CTGGTGACTCTGAACTACAGACTGGGTGCTTACGGTTTCATGTGCCTGGACACTCCTGAA

ATCCCTGGTAACCAAGGTCTGAAAGACCAACTGCTGGGTCTGAGATGGATCAAAAACAAC

ATCGCTGGTTTCGGTGGTGACCCTAACAAAATCACTGTGGTGGGTGAAGGTGCTGGTGCT

ATCTCAGCTGACTTCCACCTGCTGTCACCTCACGAAAAACTGTTCGACAAAATGATCATC

CAATCAGGTACTTCACTG

15

>Contig1615

ATGAACCTGCTGGTGCCTGAAGTGGCTTCAGAAACTAACCTGCTGCCTGTGGTGCTGTAC

ACTCACTCAGGTGCTTTCGCTGGTGGTTCAGGTTTCATGGGTAAACTGGACTACATCGCT

AGACACGACGTGATCGCTATCTCATTCAACTACAGACTGGGTGCTATCGGTTTCGCTTGC

CTGCACACTGAAGAAATCCCTGGTAACGCTGCTCTGAAAGACCTGCTGGCTGCTCTGAAA

TGGATCAACAAAAACATCAGAAAATTCGGTGGTGACCCTAACAAAGTGACTCTGGCTGGT

TACTCAGTGGGTGCTACTATGGCTGAACTGATCGCTCTGTCAGGTCACGCTGACGGTCTG

ATCGACAAACTGATCCTGGAATCAGGTTCAGCTCTGACTCCTTTCGCTATCAACAGACAC

CCTATCACTACTGCTAAAAACATCGCTACTGCTATGGGTTACAACGGTACTGGTAACATC

AACGACCTGAACGAATTCTACCTGAACGCTGAAGACGTGAACCTGGCTGCTCACTCACTG

CACCTGTACCTGACTAACTCAACTTTCGGTTTCGCTCCTTGCATCGACTCAAACAAAAAC

AACCCTCAACCTATCCTGGCTGAATCACCTCTGAAATCACTGCAAAAAAAAGAAAGAAAC

TTCGCTGTGCTGACTGGTTTCTCAAACATGGAAGGTATCTCAAGATCAATGAAATTCAAC

GAATGGTCAGACATGATGAACGGTGACTTCCTGGAATTCATCCCTGCTGACCTGATCTTC

AAAAACGAAAACGTGAAAAGAGACGCTGCTAAAGAAATCTACCAATACTACTTCAAAGAC

GAAGAAGTGACTCACGACAACCTGGAAGCTTACATCGACTACTTCTCAGACACTATGTTC

AAATACGCTATCCTGAAATCAGCTAAACTGCACGCTGACCCTACTGCTTACGAATCATCA

CTGATCAACATCCACTGGAAAAAATACCTGAACTCAACTCCTTACTACCTGTCAATCGGT

GACCAACTGCAACTGAGAGACAACATCTTCGAAGACAACAAACACTTCGACTTCTGGGAC

AACATCTACGAAAAATACTACTGGGACCCTGACATGACTAAACAA

16

>Contig11512

ATGGTGTACATCCACGGTGGTGCTTACATGGCTGGTTCAGGTGACTCAGACTCACTGGGT

CCTGAATTCCTGCTGCAACACGACGTGATCCTGGTGACTATCAACTACAGACTGGAAGTG

CTGGGTTTCCTGTGCCTGGACACTCCTGACGTGCCTGGTAACGCTGGTATGAAAGACCAA

GTGGCTGCTCTGAAATGGATCAAAAACAACATCGCTAAATTCGGTGGTGACCCTGACAAC

ATCACTCTGTTCGGTGAATCAGCTGGTTCATCATCAGTGACTTACCACCTGATCTCACCT

ATGTCAAAAGGTCTGTTCCAAAAAGCTATCGCTCAATCAGGTACTTGCCTGGAAGACTGG

GCTATCGCTAGAGACGCTAAACAAAGAGCTTTCAGAATCGGTAAATTCCTGGGTAAAGAA

ACTGACAACGTGGAAGAACTGCTGGAATTCCTGCAATCAGTGCCTGCTGTGCAACTGGCT

AACATCACTTTCAAAGTGAGAACTCCTGACGAAAAATCAAGAGGTCTGCCTATGTACTTC

GTGCCTGTGGTGGAAAAAAAATTCAACGAAGTGGACTCATTCATCACTGAAGAACCTGTG

GACCTGCTGCTGGGTAACAAAGTGAACAAAGTGCCTCTGATGATCGGTTACAACTCACAC

GAAGGTATGCTGATGATCGCTGACAAACTGAAAAAAGCTGACGTGTACAACAACAACGCT

TCATACCTGGTGCCTAGAGACATCATCTCAAGAGTGTCAGCTGACAAAGCTAAACTGTTC

GGTGACAAAATCATCAAATTCTACCTGGGTGACAAAGGTATGTCAAACGACACTGAAGAA

GGTGCTTGCCACGCTGACGACCTGTTCTACATCTTCTACGCTGGTCTGTCAGAAGACGTG

TACAAAAACGACGAAAACCTGAAAGAAATCGTGTACAAAGTGACTAAACTGTGGGCTAAC

TTCGCTTCAACTTCAAACCCTACTCCTGACAAATCACTGGGTGCTAAATGGTCACCTTTC

ACTAGAGTGGGTAAAGAATTCATGGACATCGACCTGCAACTGAAACCTGGTCACAACACT

GAAGGTGAAAGAGTGGACTTCTGGAACAGAATCTACTGCGAAGCTGGTGTGCCT

17

>Contig5490

ATGCTGTTCACTGGTCTGCTGTTCTCATGCCTGTACTGCGTGCAATACGTGGTGGGTGAA

CTGAGACTGGACCCTCTGGTGGACACTAAACTGGGTCTGATCAGAGGTGTGAGAGCTACT

GACGGTGGTTACTCAATGTTCATGGGTATCCCTTACGCTACTGTGGACGAAAACAACCCT

TTCGGTCTGTCATCACCTTACCCTAAATTCAACGGTACTTTCGAAGCTAACTACGACGGT

GCTATCTGCCCTCAACTGGAAACTTACAACAACACTATCACTGGTGTGATCGACTGCCTG

CACCTGAACATCTACGTGCCTAACACTGCTTCATTCCAAAACAAACTGCCTGTGATGGTG

TACATCCACGGTGGTTACTTCCAAATCGGTTTCGCTGGTAGATACCAATACGGTCCTAAA

TACCTGGTGAAACACGACGTGATCCTGGTGACTCTGAACTACAGACTGGGTCCTTACGGT

TTCATGTGCCTGGACATCCCTGAAGTGCCTGGTAACCAAGGTCTGAAAGACCAAATCCTG

GCTCTGAAATGGATCAAAGCTAACATCGACGCTTTCGGTGGTAACGTGCTGGCTGAACCT

ATCGTGAAAACTAAACTGGGTCTGATCAGAGGTATCAACGCTGTGGACGGTGACTACTCA

ATGTACATGGGTATCCCTTTCGCTAGAGTGAACTCATCAAACCCTTTCGGTTCAGGTACT

GCTCTGTCATCACCTTCAACTCCTATCAGAAACGCTCCTGTGCTGCTGGCTAACCACCTG

GGTTTCGACACTGACGACATCAACGAAGCTATCTCACTGCTGGCTCAAATCGAACCTAAC

CTGATCATCGCTGCTACTAAAGAACTGGGTCTGAAATTCCTGCCTTGCATCGAAAAAGAA

TTCGAAGGTGTGGAATCATTCTTCACTACTGACTGGATGAGAATGGACATCCCTAAAGTG

AAAAACATGCCTGTGCTGCTGGGTCACACTGACGACGAAGCTGGTTCAGCTATCTTCACT

ATGTCAAAAGAAACTCTGACTTCAAAACTGATCGACCAACTGGTGAGATCAAAATTCAAC

GCTTCAGACCCTGACTTCAAAGACCAACTGATGATCGACAGAATCACTGCTCTGTGGACT

AACTTCGCTAAATCAGGTAACCCTACTCCTGAAAGATCAGACCTGCTGCCTGTGGAATGG

AAACCTATCAGAAAAGGTTCACCTTACAACTGCCTGCACCTGGACAAAGAACTGTCACTG

ATCACTAGACCTTTCTACAACAGAATGGCTTTCTGGGACCTGTTCTACAAAGCTAACGAC

GACTTCCTGCTGCCTATCTCA

18

>Contig3284

GGTCTGTTCCACAGAGCTATCGCTCAATCAGGTCTGGCTCTGTCACCTTGGGCTCTGGCT

ACTTCACCTAGAGCTAGAGCTTTCGAACTGGGTAAAGAACTGGGTATCGACACTAACTCA

ACTGCTGAACTGCTGGGTGCTAGACTGGGTGGTGCTTCAGCTAGAGGTTCAGACCTGAGA

TCAACTGTGGCTCTGCCTTTCCTGCCTAGATGGTCAAGACCTAGACTGTCATTCCCTCAC

AGAAGACCTTCACACGCTGCTACTTGGTGCAGAAGACTGCAAAGAGACCCTAAACTGCTG

TCAGACCTGGAAAGAGAATTCAGAAGAGTGGTGCCTCCTGAACTGCTGACTAACGACGAC

CTGCTGGTGAGAAACATCACTAACGCTATCAGAACTTTCTACTTCCAACAAAGACCTGTG

GACATCAGAAACATCGACTCACTGATCGACCTGTTCACTGACGTGATGTTCCTGAGACCT

CTGCTGGAAACTGTGAGACTGTCAGTGGGTAGAATCTCACCTACTTACGTGTACAGATTC

GCTTTCGACGGTGCTCTGGGTCTGTTCAAAAGAATGCTGGGTATCTCACACCCTGGTGCT

TGCCACGGTGACGAAATGGGTTACCTGTTCTACTTCTCAAGACTGAACTACAGACTGGAC

GACGACTCAGCTGAACTGGCTGTGTCAAGAAGAATGGTGCAAATGTGGACTAACTTCGCT

AAAACTGGTAACCCTACTCCTACTCTGCCTGTGGACAACAAATCAATCATCGACTTCAAA

TGGCTGCCTGTGAACGACACTGTGAGAGTGAACTACCTGAACATCGACGGTAACTTCACT

CAAAAAGTGGAACCTGACGCTAAAAGAGTGAGATTCTGGGACTGGCTGTACAGAAACTAC

ACTAAAGCTCTGGCTATGAAAAACATCACTGCTGCTCCTTCACCTTCACCTACTATCACT

GTGACTCCTGACTCACCTACTTCAGAACCTGTGACTTCACCTGTGACTGAAACTCTGGCT

CCTCTGACTTCACCTGCTCCTGAAATCCTGGCTGCTGTGAACGACACTACTCCTGCTCCT

GCTGCT

19

>genemark-Contig12236

ATGAAACCTAAACTGATCGAATACAGACAAGACGCTCTGAGATGCAGACTGCAACCTTAC

CTGGGTCTGTTCGTGGCTCAAGTGTGCATCAGAAGACTGGAATCATTCCTGGAAGTGTTC

AGAAGATTCCTGAACTCAAACAAAGTGGACCTGAGATGCATCTTCTCAAGATACGACGTG

AACAACAGACTGATCGAAAGACCTAAATCAAGAGTGACTATGGTGACTGCTAGACCTTCA

CACAGAGCTAGACTGGAACTGCTGTCACAAAGAATCATCCAATGCGGTAGAACTATCACT

CTGAAATCATACACTATCAAAAGAGTGGTGATCGACATCCTGTTCTCACAAATCCCTGCT

CCTCCTGAACCTTGGGAAGGTGTGAGAGACGCTGCTAAAAACTGCAACATCTGCCCTCAA

TTCGACAAAGAAACTCTGTCAGTGGTGGGTGACGAAGAATGCCTGTTCCTGAACATCTAC

ACTCCTGAACTGCCTAAAAACGAAACTCCTCTGAGACCTGTGATGGTGTTCATCCACGGT

GGTGGTTTCCTGTTCGGTCACGGTACTGACGACTCAGCTCACGGTCCTGACTTCCTGGTG

GAAAAAGACGTGGTGGTGGTGTCATTCAACTACAGACTGGGTATCCTGGGTTTCCTGTCA

CTGGACCTGAAAGAAGCTCCTGGTAACATGGGTCTGAGAGACCAAAAATTCTCATCATTC

GGTGGTGACCCTAACAACGTGACTATCTTCGGTGTGTCAGCTGGTGGTGCTTCAGTGGAA

TACCTGCTGCTGTCACCTCTGGCTAAAGGTCTGTTCCACAAAGCTATCGCTCAATCAGGT

TCAACTCTGCTGCCTTGGGCTCACAACACTAACTCAAAAGAACTGGCTTACAAAATCGCT

GCTCTGAAAGGTGCTGTGGTGACTTCAGACGTGGAACTGCTGCAATTCCTGAAAAAAATC

CCTGCTAAAGAACTGATCCTGCTGACTATGCAAGTGCTGGCTTCAGACTCATGGAGAGGT

GGTATCAACTTCGGTTTCGTGCCTACTATCGAACACCCTGGTGACTGGGTGCCTTTCCTG

GACAAAAAACCTTACGACCTGCTGGCTAAAGGTGAATTCACTAAAGTGCCTTTCATCGCT

GGTGTGTGCACTAGAGAAGGTCTGCTGATGGTGTCACACGCTGCTCCTAGACTGGAAAAA

CTGCACAAAGAAAAAAAATTCATCGAATTCCTGCCTTTCCAAATCGACGACACTGAAAAA

GACGACGTGCAATCAAAACTGAAATCAACTTACCTGGAAGGTGAACAAAAATTCGACGAC

GAAGACGCTTTCGCTATCGACTTCTTCACTGACGTGGACTTCTTCGGTGGTGGTTACGTG

TCAACTACTCTGATCGCTAAACACAACTCACCTGTGTACTTCTACGAATTCGCTTACGAC

GGTGGTCTGAACTACCTGAAAAAAAAATACAACATCAACAGAAAAGGTGCTTGCCACGGT

GACGAAGGTGGTTACCTGATCAAATCATCAGTGCTGGACGGTGAAATCTCAAAAACTGAC

GAAATCATCCCTGTGCCTCCTGAACCTTGGGAAGGTGTGAGAGACGCTACTAAAAACTGC

AACATCTGCCCTCAATTCGACCAAGACACTAAATCAGTGATCGGTGACGAAGCTTGCCTG

TTCCTGAACATCTACACTCCTAGACTGCCTCAATTCGACACTTTCCTGAGACCTGTGATG

GTGTTCATCCACGGTGGTGGTTTCATCATCGGTCACGGTACTGACGACTCAGCTCACGGT

CCTGACTTCCTGGTGGAAAAAGACGTGGTGATGGTGTCATTCAACTACAGACTGGGTATC

CTGGGTTTCCTGTCACTGGACCTGAAAGACGCTCCTGGTAACATGGGTTTCAGAGACCAA

GTGCAAGCTCTGATCTGGGTGAAAGAAAACATCTCATCATTCGGTGGTGACCCTAACAAC

GTGACTATCTTCGGTGTGTCAGCTGGTGGTGCTTCAGTGGAATACCTGCTGCTGTCACCT

CTGGCTAAAGGTCTGTTCCACAAAGCTATCGCTCAATCAGGTTCAACTCTGCTGCCTTGG

GCTCACAACACTAACATCAAAGAACTGGCTTACAAAATCGCTTCACTGAAAGGTGCTGTG

ATCACTTCAGACGAAAAACTGCTGCAATTCCTGAAAAAACTGCCTACTAACGAACTGATC

CTGCTGGCTATGCAAGTGCTGGCTTCAGCTACTTGGAGAGGTGGTATCTACTTCGGTTTC

GTGCCTACTATCGAAAAACCTGGTGACTGGGAAGCTTTCCTGGACAAAAACCCTTACGAC

CTGCTGTCAAAAGGTGAATTCACTAAAGTGCCTTTCATGTCAGGTGTGTGCACTAGAGAA

GGTCTGCTGACTGTGCTGATCGCTGAATCAAGACTGGAAAAAAACCACAAAGAAAAAAAC

TTCATCGAATACCTGCCTTTCAACATCGACGAATCAGAAAAAGACGACGTGCAAACTAAA

CTGAAAGCTACTTACCTGGAAGGTGGTCAAAAATTCGACGACGAAGACGCTATCGCTATC

GACTTCTTCACTGACGTGGACTTCTTCGGTGGTGGTTACGTGTCATCAACTCTGATCTCA

AAATACTCATCACCTGTGTACTTCTACGAATTCGGTTACGACGGTGGTCTGAACTTCCTG

AAAAAAAAATTCAACATCGACAGAAAAGGTGCTTGCCACGGTGACGAAGGTGGTTACCTG

ATCAGATCATCAGAACTGGACGGTAAAATCTCAAAAACTGACGAAATCGTGAAAAACAGA

CTGGTGGAAATCTCATTCGACGTGAAAATGTCAATGGGTGGTAGAATCCCTTCACCTTCA

TCAGACCTGTCAGCTCTGACTCTGGCTCTGATCTCAATCATCGTGAAACTGACTTCAGAA

GCTTACCTGTGGGTGGTGTGCGGTGGTGACCAAGAATGCGCTAACACTATCACTGGTCAC

GACATCGCTAGAAGAGAA

20

>Contig10533

ATGTGGATCAAACTGTTCGCTTGCGTGCTGGTGGTGGTGGCTGGTCCTGCTTGCGCTCAA

ATGGAATCAAGAGTGGTGTCAATCAAACAAGGTCTGGTGAGAGGTTACAGAGACTACCTG

GACGTGATCTCATACTACGGTATCCCTTACGCTACTACTCCTAAAGGTGCTGACAAATTC

AAAGCTCCTCTGCCTGCTCCTGTGTGGGGTAACAACATCCTGGAAGCTACTAACAACAAC

ACTATCTGCCCTCAACTGGACTTCCAATCAGACAACCTGACTATGACTGAAGACTGCCTG

GTGGCTAACGTGTACGTGCCTATCTCACAAGAAACTCACCTGCCTGTGATCGTGTTCGTG

CACGGTGGTGCTTACATCGGTGGTTCAGCTCTGTCAGACTCACCTAAAAACCTGGTGAAC

ACTCAAAAAGTGATCGCTGTGAACTTCAACTACAGACTGAACGTGCACGGTTTCCTGTGC

CTGGGTACTGAAGACGCTCCTGGTAACGCTGGTATGAAAGACCAAGTGGCTCTGCTGAGA

TGGGTGAAAGAAAACATCGCTTCATTCGGTGGTAACCCTAACGACGTGACTATCACTGGT

TGCTCAGCTGGTGGTTCAGCTGTGGACCTGCTGATGATCTCAAAAATCACTGACGGTCTG

TTCAACAAAGTGATCTCAAACTCAGGTTGCGGTATCTCAACTTTCGGTACTCAACTGAAC

CCTCTGGAAAACGCTAAACTGCACGCTAGACTGCTGAACGCTACTAACGTGGACGACATC

AAATCACTGGCTGAATTCTACAAAACTCACCCTCTGTCAGAACTGATCCAAGGTCTGATC

CTGTCAGTGGGTCTGGCTCCTAACTCATACTTCTACTTCCAACCTTGCATCGAAAGAGAC

CTGGGTCAAGAAATGTTCCTGGACGACGCTCCTATCAACATCTACAAAAAAGGTGACTAC

AAAAAACTGCCTGTGGTGTACGGTTACGCTAACATGGAAGGTCTGTTCAGACTGCCTCAA

TTCTACCTGTGGAAAGACCTGATGAACGAAAAATTCTCAGACTTCCTGCCTGCTGACCTG

CAATTCGAATCAGACGCTCAAAAAGAAGAAGTGGCTCTGTCAATCAAAAACTTCTACTTC

GGTGACAAACCTGTGAACGAATCAACTATCCTGCAATACATCGAACTGCACACTGACCTG

CTGTTCCTGAACTCAATCTACAGAACTGTGACTCTGCAAGTGCAAGCTGGTAACAACCAA

GTGTACCTGTACGACTACTCATTCGTGACTAACTCAACTGCTGTGGTGCCTCACACTGAC

TCAGTGAGAGGTGTGGACCACTGCTTCCAAACTGTGGGTCTGATGGACGTGCCTCCTGCT

TACGAAGACCTGGTGGACGACGAACTGCTGTACATGGCTAACATCACTAGACAACTGTGG

ATCAACTTCATCACTACTGGTAACCCTACTCCTCCTGGTCACGAATCATCAGTGCCTACT

TGGCTGCCTGCTGGTGAAAACAGATCACCTCACCTGTCAATCGGTCAAGTGATCCAACTG

GCTGGTGTGCCTGCTGAAAACAGATCACTGTTCTGGGACGGTATCTACGAAAGACACTAC

AGAATGCCTATGCCTCCTCCTAACCACTCAGGTGCTGCTCCTGCTTACCTGATGTCACTG

AACACTTCAGGTCCTCTGTCAGCTTCATCATGGGTGAACGTGTACGACGCTGTGGACAAA

GGTATCATCTGCCCTCAACCTACTATCAACAACCTGACTATCGGTGGTTCAGTGAAACTG

GTGGCTGACGAAAACTGCCTGATCGCTAACATCTACGTGCCTGACACTGAAGAAAAAGAC

CTGCCTGTGATCGTGTACGTGCACGGTGGTGCTTACCAAGTGGGTTTCGGTAACATCCTG

CCTCCTAAAAACCTGGTGAAATCAGGTAAAGTGATCGCTGTGACTTTCAACTACAGACTG

GGTATCCACGGTTTCCTGTGCCTGGGTACTACTGACGCTCCTGGTAACGCTGGTATGAAA

GACCAAGTGGCTCTGCTGAGATGGGTGAAAAACAACATCGCTTCATACGGTGGTAACCCT

GGTGACGTGACTATCGCTGGTTACTCAGCTGGTTCATCAGCTGTGGACCTGCTGATGCTG

TCACCTTCAGCTCAAGGTCTGTTCAACAAAGTGATCCCTGAATCAGGTGCTAACATCTCA

CCTTGGTCAGTGCAAGTGGACCCTATCGAAAACGCTAAAGAATTCGCTAGATCAGCTGGT

CTGGAAGACACTGACGACCTGTACGCTCTGGAAGAATTCTACAAAACTGCTTCATTCAAA

CTGTTCACTTCAACTGGTGGTTTCTTCAACAGAACTGACGCTAACTTCCTGTTCACTCCT

TGCGTGGAAAGAGAAACTGGTGACGGTGCTTTCCTGACTGACGCTCCTTACAACATCCTG

AAAAGAGGTGACTACAGAAAAGTGCCTTGCTCATTCCTGCCTGCTGACCTGAAATTCGTG

AACACTGAAGAAAGAAACGAAGTGTCAAAAAAAATCAAAGAATTCTACTTCTCAGACATG

CCTGTGTCAGAAGACAACATCCTGTCATACATCGACTTCCAATCAGACGTGTACTTCGGT

TACTCATCACTGAAAGCTGTGAGACTGCACGTGCAAACTGGTCACGACAAAATCTACCTG

TACGAATACGACTACGTGGACGAATCAACTCCTGCTATCCCTCACACTGACAACGTGAGA

GGTGCTAACCACTGCGCTCAAACTATGGCTGTGCTGCAACCTGAAGACACTCTGTCAGAA

GACTCAAAAACTGTGTCAAGAACTATGCAAGAAATCTGGCTGAACTTCATCACTACTGGT

TCACCTGTGCCTGTGGGTTCATCACTGCCTGCTTGGCCTCCTGCTGGTGCTGACGGTTCA

CCTCACATGAGACTGGGTAGAACTGTGGAACTGAGAGGTGCTCTGCTGGCTGACAGAGTG

AGATTCTGGGACTCAATCTACGACCACCACTACAGAGTGCCTGCTCCTCCTCCTCCTCCT

ACTCCTTCACTGGACAACCACAACATCGAATCAATGAACAACCTGCTGCACAACGAAAAC

TGCCTGATCGCTAACATCTTCCTGCCTGAAACTGAAGAAAAAAACCTGCCTGTGGTGGTG

TACGTGCACGGTGGTGCTTACCAAGTGGGTTTCGGTAACCTGATGCCTCCTAGAAACCTG

GTGAGATCAAAAAAAGTGATCGTGGTGACTTTCAACTACAGACTGGGTATCCCTGGTTTC

CTGTGCCTGGGTACTAAAGACGTGCCTGGTAACGCTGGTATGAAAGACCAAGTGGCTCTG

CTGAGATGGGTGAACCACAACATCGGTTCATACGGTGGTAACCCTGACGACGTGACTATC

GCTGGTTCATCAGCTGGTTCATCATCAGTGGACCTGCTGATGCTGTCACCTTCAGCTCAA

GGTCTGTTCAACAAAGTGATCCCTGAATCAGGTGCTAACATCGCTTCATGGTCAGTGCAA

ATCGACCCTATCGCTAACGCTAAAGAATTCGCTAGATCAGTGGGTTTCGAAGCTACTGAC

GACATCTACGCTCTGGAAGAATTCTACAAAAAAGCTTCACTGGAACTGCTGACTTCAACT

GGTGCTTTCTTCGACAGAACTTACGCTTCATTCGTGTTCTCACCTTGCGTGGAACAAGAA

ACTGTGGACGACGCTTTCCTGACTGACGCTCCTTACAACATCCTGAAATCAGGTAACTAC

AGAAAATACCCTCTGCTGTACGGTTTCGCTAACATGGAAGGTCTGCTGAGAATCGGTCAA

TTCTCAGCTTGGAAAGACAAAATGAACGCTAACTTCTCAAACTTCCTGCCTATCGACCTG

AAATTCGAATCACTGGAACAAAGAAACGAAGTGTCAAAAAAAGTGAAAGAATTCTACTTC

GGTGACAAACCTGTGGGTGAAGACAACATCCTGTCATACATCAACTTCCAATCAGACGTG

TACTTCACTTACTCAACTCTGAGAGCTGTGAACCTGCACGTGCAAGCTGGTCACGACAAA

ATCTACCTGTACGAATACGACTTCGTGGAAGAATCAGCTCCTCCTATCCCTTACACTGAC

AACGCTAGAGGTGCTACTCACTGCGCTCAAACTTCAGCTGTGTTCAGAATGAAAGACCTG

AACATCGTGAAAAACAACGAACTGTCAGAAGACTTCAAAAACATCTCAAACATGATGCTG

GACATCTGGATCAACTTCATGACTACTGGTTCAAACGAACTGCCTACTCCTAAAAACGAA

GCTATCAAAGGTTACCTGGGTATGGACAGACAATTCCTGAGAGTGGTGGCTGCTGGTGTG

GCTGCTGGTGGTAGAGGTTCACCTCACATGAGACTGGGTAGAACTGTGGAACTGAGAGGT

GCTCTGCTGGCTGACAGAGTGAGATTCTGGGACTCAATCTACGACTCACACTACAGAGTG

CCTGCTCCTCCTCCTCCTCCTCCTGCTCAACACCAAAACCTGATCAAAGCTTTCAAC

21

>Contig938

ATGGAACTGCAATACGGTATCACTTCAGAAAACAAATACTACGGTAAAGAAAGATTCCTG

CCTGGTGAACCTTACGAACTGCTGAGAGAATGCGTGCACGAAGACGTGGACATCATCATC

GGTTACAACGAAGAAGAAGGTCTGTTCTGGTCAAAAACTGGTGTGGACGTGCCTGCTGTG

ATCGACAAAGTGAAAAGATTCGACGACTTCCTGGTGCCTAGACCTGTGGGTCTGCACGCT

TCAGAAATCACTAAACTGGAAATCGGTGAAAAAATGAAACAATACTACATCGGTAAAGAA

AACCTGTCAGAACAAGAAAACGCTCTGAGACTGTTCAAATACTACTCATTCGACCTGTTC

GTGTACGGTGTGGTGCAATGGGCTAAATTCTGGTCACAATACAGAAAAAACAACAAAACT

TACCTGTACAAATTCACTTCAAAATCAGAAAGAAACTTCATGACTCACTTCTTCGGTGCT

AGAAAATACCTGGGTGACAGAACTGTGGTGTGCCACGCTGACGAACTGCCTTACCTGTTC

CCTACTCAAATCTCAGACACTAAAGTGGACAAAACTTCAGAAGCTTTCAAACTGATCAAC

CAACTGACTACTCTGTGGACTAACTTCGCTAAATACGGTAACCCTACTCCTGACGAATCA

CTGGGTGCTAAATGGAACCCTTTCGACTCAGAAGACCAACAATACCTGGACATCGGTAAC

CAACTGAAAGCTGACTCATTCCCTGAAAAAGAAGAACTGGAATTCTGGGAAAACGTGCTG

AAAGAAGTGTACCCTAAACACGTGTTCTCAAAAAAC

22

>Contig10703

ATGTACGGTGGTGGTACTTCATACAACCCTCACAACTTCATGGACTGGGACGTGATCCTG

GTGACTCTGAACTACAGACTGGGTGCTCTGGGTTTCCTGTCAACTGGTGACGAAGTGGTG

CCTGGTAACAACGGTCTGAAAGACCAAGCTTTCGCTCTGCACTGGATCAAAGACAACATC

CACGCTTTCGGTGGTAACCCTGACCAAATCACTCTGACTGGTAACTCAGCTGGTGGTGCT

TCAGTGCACTACCACTACCTGTCACCTTTCGGTTCAGCTTTCGCTCCTTGGACTCTGGCT

ATGAAACCTGTGCAACACGCTAGAAGACTGGCTACTCTGGCTGGTTGCCCTTCATCATCA

TCAAGAGCTATGGTGGAATGCCTGATGGAAAAACCTGGTGAAGAAATCGCTCAAGCTCAA

TCACAAATCTACTACATCAACAAACCTCACCTGCTGCAAGACCTGGAAGTGAGATGGGAC

CAACTGGCTTCAGAAATCTTCGAATACAACGACACTCTGCCTCTGGAACAAAGATCAGAC

GTGGCTGCTCAAATCAGACAAAGATACCTGGGTGGTAGACCTGTGTCAGAAGAAACTTTC

CCTCACTGGTGCAAAGGTCTGGGTTACCCTCACGTGCACTCACTGCACACTACTGTGGAA

CCTGACTACCTGGGTCCTTTCTCACCTTCAACT

23

>Contig4177-0.23

ATGAGAGTGCTGCTGGCTGCTCTGACTGCTCTGGCTGCTAGAGTGCTGGCTGGTCCTCAC

GAACACAGAGCTAGACACCACGCTCCTGACCACGCTCCTCACTTCCCTGCTCCTCCTCCT

GAACCTTACCACGGTCACGGTGAAGCTGTGAGATACAACCCTGAACTGGACACTATCCTG

CCTAGAATCGAAGAACACGAAACTTCATCAAAAAGAGCTAAATTCGAAGACGCTGAAACT

TCATCAAAAAAAGCTAAATACGACGAATTCTACTCAAACCACGAAAGATCAGACGAATTC

ATGGCTGACGAACCTCAACTGGGTCCTGAAGAAGACGACCCTCTGGTGATCAGAACTAGA

AAAGGTAAAATCAGAGGTATCACTCTGACTGCTGCTACTGGTAAAAAAGTGGACGCTTGG

TTCGGTATCCCTTACGCTCAAAAACCTATCGGTGACCTGAGATTCAGACACCCTAGACCT

GTGGAATCATGGGGTGACGAAATCCTGAACACTACTACTCTGCCTCACTCATGCGTGCAA

ATCGTGGACACTGTGTTCGGTGACTTCCCTGGTTCAATGATGTGGAACCCTAACACTGAC

ATGCAAGAAGACTGCCTGTACATCAACATCGTGTCACCTAGACCTAGACCTCAAAACGCT

GCTGTGATGCTGTGGGTGTTCGGTGGTGGTTTCTACTCAGGTACTGCTACTCTGGACGTG

TACGACCCTAAAATCCTGGTGTCAGAAGAAAAAGTGGTGTACGTGTCAATGCAATACAGA

GTGGCTTCACTGGGTTTCCTGTTCTTCGACACTCCTGACGTGCCTGGTAACGCTGGTCTG

TTCGACCAACTGATGGCTCTGCAATGGGTGAAAGACAACATCGCTTACTTCGGTGGTAAC

CCTCACAACATCACTCTGTTCGGTGAATCAGCTGGTGCTGTGTCAGTGTCACTGCACCTG

CTGTCACCTCTGTCAAGAAACCTGTTCTCACAAGCTATCATGCAATCAGGTGCTGCTACT

GCTCCTTGGGCTATCATCTCAAGAGAAGAATCAATCCTGAGAGGTACTAGACTGGCTGAA

GCTGTGCACTGCCCTCACTCATCAACTGACATGGGTCCTATGATCGAATGCTCAAAAGAA

GAAAGA

24

>Contig11585

GCTCCTCAACCTGCTGAATCATGGGACGGTATCAGAGACGCTACTTCAGAAGGTAACATC

TCACCTCAAATCGACCCTGTGCTGCACAAAGGTTACGCTGGTGACGAAAACTGCCTGTAC

CTGAACATCTACACTCCTAACCTGGACGGTGCTTTCCTGCCTGTGATGGTGTACATCCAC

GGTGGTGCTTTCAAATACGGTTCAGGTTCATCATCACTGTACGGTGGTGACTACCTGGTG

GAAAAAGACGTGGTGGTGGTGACTGTGAACTACAGACTGGGTGCTCTGGGTTTCCTGTCA

CTGAACACTCCTGAAGTGCCTGGTAACGCTGGTCTGAAAGACATCGTGGCTGCTATCAAA

TGGGTGAAAGCTAACATCCACAACTTCGGTGGTAACTCAGGTAACCTGACTCTGTTCGGT

GAATCATGCGGTGGTATCTCAACTTCAATCCTGACTGCTTCACCTGCTTCAAAATCACTG

ATCAACAAAGCTATCATCCAATCAGGTACTGCTCTGTCATCAATGGCTTACCAAAAAAAA

CCTGTGGAAAACGCTAGAAAACTGGCTCACCTGCTGGGTTCAGAAGCTGAAGACGTGGAC

GAAATCCTGGACTTCCTGACTGCTACTCCTGCTAAAGAAATCGTGGAAGCTACTGAAAAA

CTGCAACCTCTGGACCTGCTGCAAGAAAACAACGGTAACTACTTCTCAATCATCGTGGAA

AAAGAATTCCCTGGTGTGGAAGCTGTGCTGACTGAATCATTCTACGACATGCTGATCTCA

GGTAGAGTGGCTGACATCCCTGTGATGGTGGGTTCAACTACTCTGGAACTGACTCTGGAA

AGAACTACTGACGACCTGCAAATCTTCATCCCTGACGACCTGAAAATCGAAAGAAACTCA

GAAGAATCACTGAGAATCGCTAACGAAATCAAAGGTCTGTACTTCAAAGGTTCACACACT

GGTGTGGAATCACTGAAAGAATACTTCGAACTGCTGTCAGACAAAGTGGTGAACGTGGAC

ACTTACAGATACATCAAACACCTGGTGCAAGTGTCAACTAAACCTATCTACTTCTACAAA

TTCGACTACGTGGGTGAACTGAACCTGACTAAAAAACTGCTGGCTTCACTGGGTCTGAAC

CACGCTGGTCACATGGACGAACTGGGTTACCTGAACCCTACTCCTGACGAAAACCACTAC

ATCACTGTGACTTGGCTGCCTGTGACTACTGACAACCTGTACTACCTGAACCTGGGTAAC

GAACTGTCACTGGGTTCAAACCCTGACCAACCTAAAATGGACTTCTGGGAACAACTGTAC

TCAAAACACTTCAGAATCTGGGAACACCCTTCAAGAAACACTGAAGAAGCTCCTAGAAAA

CAAGAACCTATCAAAATCGAAGACCCTGTGGAATCACCTGCTGTGCCTCCTGTGAACAAC

TCAGTGGTGATCGTGGAAACTCTGGCTGCTGAAGTGGTGGAAGACCAACCTGACGAAGCT

CCTGAACAAGAAATCGTGAACGAAGTGGTGCACGAAGAAATCAAAGAAGAAGAAGAAATC

AACGACAAAGTGGACATCAACGACAAAGTGGAAAACGGTAAAGAAATCGAAGACAAAATC

GAAAACGGTAAAGAAGTGGTGGAAAAAGAAGAAGTGCACATCAACGGTAACTCAAACGAA

TCAAGAAAACCTAGAACTTCAAACGAAATCAAAATGGTGAACAACGCTAACGGTGTGGCT

AAAGACGTGATCAGAGCTAACGACCCTCCTGAAACTATCTACCTGAGAATC

25

>genemark-Contig4302

ATGAACCACAGAAGAAGAGTGGCTTCACACCAAGGTGGTATCCTGTCACCTCTGCTGTTC

TCAGTGTTCATCAACATCATCACTTCACAAATCTCATGCTCATACCACCTGTACGCTGAC

GACCTGCAACTGTACGCTTCAGGTAGATGGGACGAACTGACTGAAGAAATCGCTGAACTG

AACGCTAACATCGACAGAATCTCACTGTGGGCTGGTAACGCTTTCCTGGGTCCTGACGTG

CTGCAAATGTTCATGGGTGGTGTGGTGACTTACCACCAAGTGAACCTGACTTCAGTGACT

GTGTCATCACAACACAAATCACTGGCTAACTTCAGAACTATCGGTTACAGATTCTACGCT

CTGTGCGCTAGAGTGCCTCTGAACTGGCAACCTACTGCTAACACTATGGGTAGATCACTG

TACTTCTCATACGACATCCTGGACACTGGTTCATACAGACCTGTGCCTTCATGGTGCGAA

ACTTCAGCTAGAACTCACTCAGGTTGGGTGTGCGGTAGAAGACTGAGATCAGTGACTGGT

ACTGAATACGCTTCATTCCTGGGTGTGCCTTACGCTAAACAACCTGTGGGTGACCTGAGA

TTCCAAGAACTGCAACCTCTGGAACCTTGGTCAGGTTTCCTGAACGCTATGTCAGAAGGT

CCTGTGTGCCCTCAAACTGACGTGGTGTACGGTCAACTGATGCAAGCTAAAGGTGGTATG

TCAGAAGCTTGCATCCACGCTAACGTGCACGTGCCTATCAAAGCTCTGCCTGCTGGTGAC

GTGGCTGTGGACCAAACTGCTCCTGGTCTGCCTATCATCGTGTTCATCCACGGTGGTGGT

TTCGCTTTCGGTTCAGGTGACACTGACATCCACGGTGCTGACTACTTCATCAACAAAGAC

GTGATCGTGATCACTTTCAACTACTCAATCCCTGGTAACAACGGTCTGAGAGACGCTATC

ACTCTGCTGTGCTGGGTGCACAGAAACGCTGCTGCTTTCGGTGGTAACCCTGACGACATC

ACTATCGGTGGTCAATCAGCTGGTGCTGCTCTGGCTCACATGCTGACTCTGTCACCTGCT

TCAGAAGGTCTGTTCAAA

26

>Contig5389

CTGTCAGAAATCAAAAAAGTGCAAATCCTGGCTAGAGACTACGAAACTCCTCTGGGTCCT

ACTGTGGCTGGTTCATTCATCCCTTCAGAACCTGCTAAAACTATGGAAGCTTTCCCTAAC

CTGCTGTCAAAATACCAACTGCTGTCAGGTGTGACTGAATTCGAATCATACCACGACTTC

GGTGTGCTGGAACTGGGTCAAGGTGTGCTGGAAGCTCAAAGAGACGAATTCATCACTAAA

TACGCTAAAGTGATCTTCGAAGGTGCTGAAGACGAAGCTCTGAAAGAAATCCTGAAACAA

TACGCTCCTTCACTGATCGACCCTCAAAGATGGTCAGTGGAAACTAACAGAGACGTGGTG

CTGAACCTGTTCTCAGACGCTAGAACTATCGCTCCTACTATCTCATTCGCTAACTACCAA

TCAAGATCAAACAGACAATCATACTTCTACGTGTTCGGTCACAACTCAATCTCAACTGAC

TACGCTCAACTGAACAAATCAGTGCACGGTCAAGAAATCCCTTACGTGCTGGGTGTGCCT

CTGGGTGGTGCTAACACTCACTTCACTAACGTGTACACTCAATCAGAAAAACTGCTGTCA

GAAGTGGTGATGAGACTGTGGACTAACTTCGTGAAAATCGGTACTCCTAACACTCAATCA

GTGAACAAATACTTCACTGCTGACAAAGAATCATGGAACCAATACAACGTGGAATGGCCT

GAATACGACGTGGACCACCAATCATACCTGAAACTGGCTATCCCTCCTCAAATCTCATCA

CTGTTCGTG

27

>genemark-Contig5389

ATGGCTAACGAAGGTGCTCCTGGTATGATCGGTGACACTAGATCACTGGGTGAAGACGGT

AAAAGACTGACTAGAGAATACCACCTGAGACAAGGTGCTCTGAGAGGTCTGATCGTGAAA

CCTTCAAGACACTACGACCTGCAAGCTGTGGAAATGTTCCTGGGTGTGCCTTACGCTGCT

CCTCCTACTGGTAACCTGAGATTCATGCCTCCTGTGTCAGCTGTGGTGGGTAGATCATCA

ATGGCTCAAAACCACAGATGGTGGTCAGACAGAGACATCTGGAGAAAAATGTCAGAAACT

TTCGTGCAACAATGGACTGCTATCGGTACTCCTCCTTACAAAGAAGGTGAATCAACTGAC

GTGGGTACTCCTGCTCTGCACGACAAAGCTGAAGGTTTCCCTCAAAAAAGAGTGAGAAGA

CTGTCACTGTTCAAACACCTGAGATCAGTGTCATCATCACAAAGATACCCTTGGACTGCT

CACCAAACTAACTCATCAATCCTGAAAGAACTGAACATCAAAGAAAGACTGTCATCAACT

GTGCAAATCAGAATCCTGAAAGCTTTCGAACACATCACTAGAGCTGAAGACTCAATCGAA

AGACTGGTGGTGCAAAGAAAACTGAAAGGTAACAACCTGGTGGGTGCTCTG

28

>genemark-Contig5389

ATGGCTTACGCTGTGGTGAGATCAACTGCTAGATGGTCATCAGGTAACCCTTACGACGGT

AAAATCCTGGCTTCATACGGTAACATCATGGTGATCACTATCAACTTCAGACTGGGTATC

CTGGCTGAATTCGAATCAAGATCAGGTAGACCT

29

>Contig9166

ATGAAAGTGTTCCTGTTCCTGCTGGCTGCTCTGGTGGCTGTGGAAGCTCAAAGAATCCCT

AAAGTGGTGCAAATCAAACAAGGTCTGGTGAGAGGTTACTCATTCGACCACTACGACGCT

TTCGCTTTCTTCGGTATCCCTTACGCTACTGCTCCTACTGGTGCTGAAAAATTCAAACCT

CCTCTGCCTCCTCCTACTTGGAACGGTATCTTCGAAGCTGTGGACAGATACGTGTTCTGC

CAACAACTGTCAATCTTCCCTGAAGGTCAATTCAGATTCGTGGAAGACTGCCTGGCTATC

AACGTGTTCGCTCCTAACACTAACCAAACTAACCTGCCTGTGGTGGTGGTGATCCACGGT

GGTGGTTTCCAATTCGGTTTCGGTAACACTGAAACTCCTGGTCAACTGATCACTGAAGAA

AAAAACGTGATCGGTGTGACTTTCAACTACAGACTGGGTCCTCACGGTTTCCTGTGCCTG

GGTACTCCTGACATCCCTGGTAACGCTGGTATGAGAGACCAAATCGCTGCTCTGAAATGG

GTGAAAGAAAACATCGCTGCTTTCGGTGGTAACCCTAACGACATCACTATCGCTGGTTGC

TCAGCTGGTGGTGCTTCAGTGGACCTGCTGATGCTGTCAAAAGCTGCTGACGGTCTGTTC

AACAAAGTGATCGGTCAATCAGGTGCTAACATCGGTGCTTTCGCTGTGCAAGTGGACCCT

ACTGAAAACGCTAGATGGTACGCTAAATCAATCAACTACGACGGTCCTGACGACATCGAA

GCTATCGAAGAATTCTACAAAAACATCTCATACAAACAACTGTTCTCATACGACCTGAAC

GCTCAAAAAGACGTGCACGTGGTGATGTCACCTTGCATGGAAAGAGACCTGGGTATCGAA

AGATTCCTGGACGACACTCCTATCAACATCCTGAAATCAGGTAACTACACTAGATACCCT

GTGCTGTACGGTTGGGCTGGTATGGAAGGTCTGTACAGAATCCAAAACTTCGACGACTGG

ATCATCCACATGAACCAAGACTTCACTCAATTCATCCCTACTGACCTGAAATTCGAATCA

CCTGAACAAAAACAACAAATCGCTGACAAAGTGAAAGCTTTCTACTTCGGTGACACTCCT

GTGAACAACTCAGACGTGCTGAACTACATCGACTTCAACTCAGACGTGATGTTCAACGTG

GCTATGCAAAGATCAGTGACTATGCAAGTGGAAGCTGGTAACAACGCTATGTACGTGGGT

GTGTACTCATTCACTGACAACAACACTAACATCATCCCTCACACTAACGTGAGAGGTGCT

AACCACTGCGAACAAACTAGAGCTGTGTTCGACCTGAACGACAACACTCTGACTCCTGAA

TACATCGAACTGAGAACTGCTATGAGAAAAATGTGGCTGAACTTCATCCACACTGGTGAC

CCTACTCCTGCTAACAACATCCCTCCTCTGTTCCCTAAATGGGAACCTGCTGCTGCTAAC

AGAACTCACGCT

30

>Contig5480  **(Resistance)**

ATGAAAGTGTTCCTGTGCGTGGTGTCAGCTCTGGCTGCTGTGCAAGCTCAAGACACTCCT

TCAAGACTGGTGAACATCACTCAAGGTCCTGTGAGAGGTTACAAAGAACCTGGTCTGGAC

GTGTTCTCATTCTACTCAATCCCTTACGCTAAAGCTCCTACTGGTCCTGACAAATACAAA

GAAAAAAACGTGATCGCTGTGACTTTCAACTACAGACTGGGTACTGTGGGTTTCCTGTGC

CTGGGTACTCCTGACATCCCTGGTAACGCTGGTATGAGAGACCAAGTGGCTGCTCTGAAA

TGGGTGCAACAAAACATCGCTTCATTCGGTGGTAACCCTAACGACGTGACTATGCTGGAC

GCTGTGCTGGAAGAAAGACAAAACCTGGCTAAAGACTTCGGTTACTCAGGTCCTGACAAC

ATCGAATCACTGGAAGCTTTCTTCAAAAACGTGTCATACGACACTATGAACGCTCTGTCA

CTGCACGAAAACAAAGACGTGGAAATCAAAATGTCACCTTGCCTGGAAAGAGACATCGGT

ATCGAAAGATTCCTGGAAGACACTCCTATCAACATCATCAAAAAAGGTGACTTCATCAGA

CTGCCTATCCTGTACGGTTGGACTAACATGGAAGGTATCTTCAGACTGTACGTGTTCGAC

ACTTGGAAAAACGAAATGAACGAAAACTTCACTGCTTTCCTGCCTTCAGACCTGAAATTC

GACTCAGAAGAACAAAGACAACAAATCGCTGAAAAAGTGAAACAATACTACTTCGGTGGT

CCTGTGGGTAACAACAACGTGCTGAGATTCATCGACTACATCACTGACGTGACTTTCAAC

GTGGCTATGCAAAGAGCTGTGACTATGCAAGTGGAAGCTGGTAACAACAACATGTACATC

GGTGTGTACTCATTCTCAGACAACCAAACTATGGTGATCCCTCACACTAACGAAAGAGGT

GCTAACCACTGCGACCAATCAGTGGCTATCCTGGACCTGGACGAATCAAACCTGACTTCA

GAATACCTGGAACTGAGAAAAGCTTGGAGAGCTGTGTGGCTGAACTTCATCACTAAAGGT

CCTCCTCTGCCTCCTCCTAACTGGACTACTCCTTTCGAAGCTACTCAAAGACACGTGATC

TGCCACCAATACAACATCTTCGGTCCTGGTCTGACTTTCCAAGAAGACTGCCTGGTGGCT

TCAGTGTTCGTGCCTGACACTAACATCACTAACCTGCCTGTGCTGGTGGTGATCCACGGT

GGTACTTTCCAATCAGAATGGGGTAACATCGAAACTCCTTCACAACTGGTGAACCAAGGT

AAAAACATCATCGCTGTGACTTTCAACTACAGACTGGGTCCTGTGGGTTTCCTGTGCCTG

GGTACTCCTGACGTGCCTGGTAACGCTGGTATGAGAGACCAAGTGGCTGCTCTGAAATGG

GTGAAACAAAACATCGCTGCTTTCGGTGGTAACCCTAACGACATCACTATCGCTGGTTGC

TCAGCTGGTGGTGCTTCAGTGGACCTGCACTTCATCGCTAAAGCTTCAAGAGGTCTGTTC

AACAAAGTGATCCCTCAATCAGGTGGTAACATCGGTGCTTTCGCTGTGCAAGTGGACCCT

ACTCTGAACGCTAGAAACTTCGCTCAAGACATCGGTTACAACGGTACTGACAACCTGGAA

TCACTGGAAGCTTTCTACAAATCAGCTTCATACGAACAACTGTTCTCAATCTCACTGCAC

GACAACAAAGACGTGGAAATCAAAATGGCTCCTTGCCTGGAAAGAGACACTGGTATCGAA

AGATTCCTGGAAGACACTCCTATCAACATCATCAAAAAAGGTGACTTCGTGCAAATGCCT

ATCCTGTACGGTTGGGCTGGTATGGAAGGTATCCTGAGACTGTACCTGTTCGAAACTTGG

AAAGTGCAAATGAACCAAGACTTCTCACAATTCCTGCCTTCAGACCTGAAATTCGAATCA

GCTCAACAAAAACAAGAACTGGCTGAAAAAGTGAAAAAATTCTACTTCGGTGACGCTGTG

TCAAACGAAAACATCCTGAGATACATCGACTTCCACTCAGACGTGATGTTCAACGTGGCT

ATGCAAAGAGCTGTGACTATGCAAGTGGAAGCTGGTAACAACAACATGTACATCGGTGTG

TACTCATTCACTGACAACCAAACTATGGTGATCCCTCACACTAACGAAAGAGGTGCTAAC

CACTGCGACCAAACTGTGGCTATCTTCGACCTGAACGAATCAACTATCACTCCTGAATAC

CTGGAAACTAGAAGAGCTTGGAGAGCTATCTGGCTGAACTTCATCACTACTGGTGAACCT

ACTCCTGACGGTAACATCCCTCCTCCTTTCCCTAAATGGGAAGCTGCTAGAGCTAACAGA

ACTCCTTGCATGGAAATCGGTAAAACTATCAGAGTGTACCCTGGTCCTTACGACCTGGAA

AGACAACAATTCTGGGACGAAATCTACGACCAACACAAAAAAGACCCTATCGCTCCTTAC

GACTTCAACAACGGTGCTCTGACTCTGTACAACGGTATCATCCTGACTTTCATGAACTTC

GACTCATTCTTCTGGACTCAAAACATGAGAATCATCCTGGGTATCATGGTGGCTCTGGCT

GCTGTGCAAGCTGAAGTGAACATCTCAAGACTGGTGACTATCTCACAAGGTCCTGTGAGA

GGTTACAAAGAACTGGGTCTGGACGTGTTCTCATTCCTGTCAATCCCTTACGCTACTGCT

CCTACTGGTGCTGACAAATTCAAACCTCCTCTGCCTCCTCCTAACTGGACTATCCCTTTC

GAAGCTACTAGAAGAAACGTGCTGTGCAACCAATTCAACTTCATGGGTTCAAACTTCCAA

ACTCAAGAAGACTGCCTGGTGGCTTCAGTGTTCGTGCCTGACACTAACAAAACTAACCTG

CCTGTGCTGATCGTGATCCACGGTGGTGCTTTCCAAAACGGTTGGGGTAACTCAAGAACT

CCTTCACAACTGGTGAACCAAGGTAAAAACATCATCGCTGTGACTTTCAACTACAGACTG

GGTCCTGTGGGTTTCCTGTGCCTGGGTACTCCTGACATCCCTGGTAACGCTGGTCTGAGA

GACCAACTGGCTGCTCTGAAATGGGTGAAACAAAACATCGCTGCTTTCGGTGGTAACCAA

AACGACATCACTCTGGCTGGTTGCTCAGCTGGTGGTGCTTCAGTGGACCTGCACCTGCTG

GCTAAAGCTTCAAGAGGTCTGTTCAACAAAGTGATCCCTCAATCATCATGCAACATCGGT

GCTTACTCAGTGCAAGTGGACCCTATCCTGAACGCTAGAAACATCGCTAAAGACCTGGGT

TACAAAGGTACTGACAACCTGGACAACCTGGAAGCTTTCTACAAAAACGCTTCATACGAA

CAACTGTTCTCAATCTCACTGGACGACAACAGAGACGTGGAAATCAAAATGGCTCCTTGC

CTGGAAAGAGACACTGGTATCGAAAGATTCCTGGAAGACACTCCTAGAAACATCCTGAAA

AAAAAAGACTTCGTGAGAATGCCTACTCTGTACGGTTGGGCTGGTATGGAAGGTATCCTG

AGACTGTCAAGATTCCAATCATGGAAAGTGCTGATGAACCAAGACTTCTCACAATTCCTG

CCTTCAGACCTGAAATTCGACTCAGCTCAACAAAAACAAGAACTGGCTGAAAAAGTGAAA

GAATTCTACTTCGGTGGTGCTGTGTCATCAGAAAACATCCTGAGATTCATCGACTTCAAC

TCAGACGTGATGTTCAACGTGGCTATCCAAAGAGCTGTGACTATGCAAGTGGAAGCTGGT

AACAACAACGTGTACATCGGTGTGTACTCATTCACTGACAACCAAACTATGGTGATCCCT

CACACTAACGAAAGAGGTGCTAACCACTGCGACCAAACTCTGGCTATCTTCGACCTGGAC

GAATCAACTCTGACTCCTGAATTCATCGAAACTAGAAGAGCTTGGAGAGCTATCTGGCTG

AACTTCATCACTACTGGTGAACCTACTCCTGAAGGTAACATCCCTCCTCCTTTCCCTAAA

TGGGAAGCTGCTAGAGCTAACAGAACTCCTTGCATGGAAATCGGTAAAACTATCAGAGTG

TACCCTGGTCCTTACGACCTGGAAAGACAACTGTTCTGGGACAAAATCTACGACCAACAC

AAAAAAGACCCTATGCCTCCTTACGACTTCAACAACGGTGCTCTGATCCTGCACAACGTG

GCTGCTCTGAAATGGGTGCAACAAAACATCGTGGCTTTCGGTGGTAACCCTAACGACGTG

ACTATCGCTGGTTGCTCAGCTGGTGGTGTGTCAGTGGACCTGCACATCCTGTCAAAACCT

TCAAGAGGTTTCTTCCACAAAGTGATCGGTCAATCAGACTCACTGGAAGCTCTGGAAGAA

TTCCACAAAACTGTGTCATACGACAAACTGCTGTCACTGCCTCTGTACGAATCAAAAGAC

GTGGAAATCCTGATGTCACCTTGCCTGGAAAGATACATCGGTATCGAAAGAATCCTGGAA

GAACCTAGACTGCAAACTATGAAAAGAAGAGACTTCCCTAAATACCCTATCCTGTACGGT

TGGGCTGCTATGGAAGGTCTGCTGAGACTGTACGCTTTCGAAAACTGGAAAAACGAAATG

AACGAAGACTTCGCTAAATTCCTGCCTTCAGACCTGATCTTCTACAACGACAACCACAAA

AAACAAGTGCCTGACAAAGTGAAAAACTTCTACTTCGGTAACTCAGTGGCTAACGAAAAC

ATCCTGAGATACATCGACTACAACTCAGACGTGATGTTCAACGTGGCTATGCAAAGAGCT

GTGACTATGCAAGTGGAAGCTGGTAACAACGCTATCGACCCTACTCCTAACGGTAACATC

CCTGCTCCTTTCCCTAAATGGGAACCTGCTAGAGCTAACAGAATCCCTTGCAAAGAAATC

GGTAACACTATCAGAATCTACCCTGGTCCTTACGACCTGGAAAGACAACAATTCTGGGAC

GAAATCTACGACCTGTACAAAAGAGACCCTATCGCTCCTTACGACTTCAACAACGCTATC

ATGCTGTACAACGGTATCCTGCTGAAAATGAAAGGTTTCTTCTACGTGATCGTGGCTCTG

GCTGCTGTGCAAACTGCTGAAAACCACTCAAAACTGGTGAACATCGACCAAGGTCCTGTG

AAAGGTTACAAAGTGCCTGGTACTGACGTGTTCGCTTTCTACTCAATCCCTTTCGCTAAA

GTGCCTACTGGTCCTGACAGATTCAAAATGCACCAACACGAATTCGGTCCTCCTCTGCCT

CCTCCTGTGTGGACTGAACCTTTCGAAGCTGTGAACAAAGACGTGCTGTGCCTGCAATAC

GACACTTTCCCTCCTGGTAAATACGAATTCGTGGAAGACTGCCCTTTCGGTTTCCTGTGC

CTGGGTACTCCTGACATCCCTGGTAACGCTGGTATGAGAGACCAACTGGCTGCTCTGAAA

TGGGTGCAACAAAACGTGGCTGCTTTCGGTGGTAACCCTAACGAAGTGACTATCGCTGGT

TGCTCAGCTGGTGGTGCTTCAGTGGACCTGCACATCCTGGCTAAAGCTTCAAACGGTCTG

TTCAACAAAGTGATCACTCAATCAGGTGCTAACATCGGTGCTTTCGCTGTGCAAACTGAC

CCTACTCTGAACGCTAGAAACTACGCTAAACAAAGAGACATCGGTATCGAAAGATTCCTG

GAAGACACTCCTATCAACATCATCAGAAAAGGTGAATTCACTAGATACCCTATCCTGTAC

GGTTGGGCTGACATGGAAGGTATCCTGAGAGTGTACTCATTCGACTCATGGAAAAACGAA

ATGAACGAAGAATTCACTAAATTCCTGCCTTCAGACCTGAAATTCGAATCAGAAGAACAA

AAACAACAAATCGCTGACAAAGTGAAACAATTCTACTTCGACGGTGACGTGACTAACGAC

AGAATCCTGAACTACATCGACTACTCATCAGACGTGATGTTCAACGTGGCTATGCAAAGA

GCTGTGACTATGCAAGTGGAAGCTGGTAACAACGCTATGTACGTGGGTGTGTACTCATTC

ACTGACAAAAACACTTCAATCATCCCTTACACTAACGAAAGAGGTGCTACTCACTGCGAC

CAAACTGTGGCTATCCTGGACCTGGACGAATCAACTCTGTCACCTGAATACCTGGAACTG

AGAAGAGCTTGGAGAGCTGTGTGGCTGAACTTCATCTCAACTGGTGACCCTACTCCTGAC

GGTAACATCCCTCCTCCTTTCCCTAAATGGGAAGCTGCTAGAGCTAACAGAACTCCTTGC

ATGGAAATCGGTAAAACTATCAGAGTGTACCCTGGTCCTTACGAACCTGAAAGACAACAA

CTGTGGGACGAAATCTACGACCAACACAAAAGACACCCTATCGCTCCTTACTACTTCAAC

GAAGAAGAATCAGTGGAC

31

>Contig5480

ATGAACGTGTGGCTGTGCTTCGTGGTGGCTCTGGCTGCTGTGCAAGCTCAAGACGACTCA

AAAATCGTGAACATCAAACAAGGTCCTGTGAAAGGTTACAGAACTCCTGGTTTCGACGTG

TTCGCTTACTACGACATCCCTTACGCTACTGCTCCTTCAGGTGTGAACAAATTCAAAATC

CTGACTCAAAAACTGACTGGTACTCCTGACATCCCTGGTAACGCTGGTATGAGAGACCAA

CTGGCTGCTCTGAAATGGGTGAAAGACAACATCGCTGCTTTCGGTGGTAACCCTGACGAC

ATCACTATCGCTGGTTGCTCAGCTGGTGGTGCTTCAGTGGACCTGCACATGCTGTCAAAA

GCTTCAAACGGTATCTTCAAAAGAGTGATCGGTCAATCAGGTGCTAACATCGGTTCATTC

GCTGTGCAAGTGGACCCTACTGAAAACGCTAGATTCCTGGCTAAACAAATCAACTACGAC

GGTCCTGACAACATCGAAGCTATCAACGAATTCTACCTGAACGTGCCTTACGAACAACTG

CTGTCATACTCACTGAACGAAGACGTGGCTGTGAAAATGTCACCTTGCATCGAAAGAGAC

CTGGGTTTCGAAAGATTCCTGGAAGACTCACCTATCAACATCATCAAAAAAGGTAACTAC

GTGAGATACCCTGTGCTGTACGGTTGGGCTGGTATGGAAGGTATCTACAGACTGGACCAC

TTCGAAGACTGGTCAGAACACATGAACGAAAACTTCGCTCAATTCCTGCCTTCAGACCTG

AAATTCGAATCAGAAGAACACAAACAAGAAGTGGCTAAAAAACTGAAATCATTCACTCTG

GTGACTCCTATGTCAGTGACTGAAGCTGGTAACAACGCTATGTACGTGTCAGTGTTCACT

TACACTGACAACTCAACTAGAATCATCCCTTACACTGACCTGAGAGGTGCTACTCACTGC

ACTCAATTCAAAGTGGTGTTCGACCTGGACGAATCAAAATACTCACAAGAATACATCGAC

CTGAGAGCTACTATGAGAGAAATCTGGCTGAACTTCATCCACACTTCAGCTGTGTTCGAA

TACCTGAGAATGAAAATCCTGTTCTGCATCTTCCTGACTCTGTCAGCTGCTCAAGCTAAA

GGTGACTCAAAAATCGTGAACATCGGTCAAGGTCCTGTGAAAGGTTACAGAGTGTCAGAA

CTGGACGTGTTCGCTTACTACTCAATCCCTTACGCTACTGCTCCTAAAGGTGTGGACAAA

TTCAAAGCTCCTCTGCCTCCTCCTGTGTGGACTGAACCTTTCGAAGCTGTGACTCAAGAA

GTGCTGTGCCTGCAACACGACGTGTTCAACAAAACTATCAAATTCGAAGAAGACTGCCTG

GTGGCTTCAGTGTTCGCTCCTGACACTGACGAAAAAAACCTGCCTGTGCTGGTGATCATC

CACGGTGGTGGTTTCCAAATCCTGTTCGGTAACTTCGAAACTCCTGCTAACCTGGTGAAC

CAACAAAAAAACATCGTGGCTGTGACTTTCAACTACAGACTGGGTCCTATCGGTTTCCTG

TGCCTGGGTACTCCTGACATCCCTGGTAACGCTGGTATGAGAGACCAACTGGCTGCTCTG

AAATGGGTGAGACAAAACATCGCTGCTTTCGGTGGTAACCCTGACGACATCACTATCGCT

GGTTGCTCAGCTGGTGGTGCTTCAGTGGACCTGCACATGCTGTCAAAAGCTTCAAACGGT

CTGTTCAACAAAGTGATCGGTCACTCAGGTGCTAACATCGGTTCATTCGCTGTGCAAGTG

GACCCTATCGAAAACGCTATCTTCCTGGCTAAACAAATCAGATACGACGGTCCTGACAAC

ATCGAAGCTCTGAACGAATTCTACAAAAACGTGCCTTACGAACAACTGCTGTCACACTCA

CTGAACGAAAACATCGACGTGCACGTGAAAATGTCACCTTGCATCGAAAGAGACCTGGGT

TTCGAAAGATTCCTGGAAGACACTCCTATCAACATCCTGAAAAAAGGTGACTACGTGAGA

TACCCTGTGTTCTACGGTTGGGCTGACATGGAAGGTCTGTACAGAATCAGATCATTCGAA

TACTGGAAAGACCTGATGAACGAAGACTTCTCACTGTTCCTGCCTACTGACCTGAAATTC

GAATCAAACGAACACAAACAAGAAGTGGCTAAAAAAGTGAAAAAATTCTACTTCGGTGAC

GACCCTGTGAACGGTGACACTATCCTGGGTTTCATCGACTACAACTCAGACGTGATGTTC

GTGGCTGCTATGCAAAGATCAATCACTATGCAAGTGGAAGCTGGTAACAACGCTATGTAC

GTGTCAGTGTTCTCATACACTGACAACGACACTTCACTGATCCCTTACACTCAAATGAGA

AGATCACACGCTTTCAACCCTACTCCTGAAGGTAAAATCCCTGCTGAAGTGCCTAAATGG

GAACCTGCTTCAGCTAACAGAATGCCTTGCATGGACATCGGTAAAACTATCAGAGTGCTG

CCTGGTGGTTACGAAGAAACTAGACAACTGTTCTGGGACGAACTGTACGACCTGTACAAA

TACGAACCTATCGCTCCTTACGACTTC

32

>Contig5480

ATGAAAGTGCTGTGCTGCATCTTCCTGGCTCTGTCAGCTGCTCAAGCTCAAAAAGACTCA

AAAATCGTGAACATCGGTCAAGGTCCTGTGAAAGGTTACAGAGTGTCAGACCTGGACGTG

TTCGGTTACTACTCAATCCCTTACGCTACTGCTCCTAAAGGTATCGACAAATTCAAAGCT

CCTCTGCCTCCTCCTGTGTGGACTGAACCTTTCGAAGCTGTGACTCAAGAAGTGCTGTGC

CTGCAACACGACGTGTACAACAAAACTAAAAAATTCGAAGAAGACTGCCTGGTGGCTTCA

GTGTTCGCTCCTGACACTGACGAAAAAAACCTGCCTGTGCTGGTGATCATCCACGGTGGT

GGTTTCCAAATCTACTTCGGTAACTCAGAAACTCCTGCTAACCTGGTGAACCAACAAAAA

AACATCGTGGCTGTGACTTTCAACTACAGACTGGGTCCTATCGGTTTCCTGTGCCTGGGT

ACTCCTGACATCCCTGGTAACGCTGGTATGAGAGACCAACTGGCTGCTCTGAAATGGGTG

AACAAAATCCTGCCTCTGCTGGTGAGAGACCTGGGTTTCGAAAGATTCCTGGAAGACACT

CCTATCAACATCCTGAAAAAAGGTGACTACGTGAGATACCCTGTGTTCTACGGTTGGGCT

GACATGGAAGGTCTGTACAGAATCAGATCATTCGAATACTGGAAAGACCTGATGAACGAA

GACTTCTCACTGTTCCTGCCTACTGACCTGAAATTCGAATCAAACGAACACAAACAAGAA

GTGGCTAAAAAAGTGAAAAAATTCTACTTCGGTGACGACCCTGTGAACGGTGACACTATC

CTGGGTTTCATCGACTACAACTCAGACGTGATGTTCGTGGCTGCTATGCAAAGATCAATC

ACTATGCAAGTGGAAGCTGGTAACAACGCTATGTACGTGTCAGTGTTCTCATACACTGAC

AACGACACTTCACTGATCCCTTACACTCAAATGAGAGGTGCTACTCACTGCGACCAATTC

GAAATCGTGTTCGACCTGGACGAAGGTAAAAGATCAAACGAATACAGAGACCTGAGAAAA

ACTCTGAGAGAAATCTGGATCAACTTCATCCACACTGGTAACCCTACTCCTGAAGGTAAA

ATCCCTGCTGCTGTGCCTAAATGGGAACCTGCTTCAGCTAACAGAATGCCTTGCATGGAC

ATCGGTAAAACTATCAGAGTGCTGCCTGGTGGTTACGAAGAAACTAGACAAAGATTCTGG

GACGAACTGTACGACCTGTACAAATACGAACCTATCGCTCCTTACGACTTC

33

>Contig5480

ATGGGTAGAAACGGTAGATACCTGAGACTGGAACACTTCGACAACTGGAAAATCGAAATG

AACGAAAACTTCAAAAACTTCCTGCCTTCAGACCTGGAATTCGAATCAGAAGAAGTGAAA

CAAAAAGTGGCTGACAGAGTGAAAGAATACTACTTCGGTGACGCTGTGACTAACGACAAA

GTGCTGAACTACATCGACTACCACTCAGACGTGATGTTCAACGTGGCTATGCAAAGAGCT

ATCACTATGCAAGTGGAAGCTGGTAACGACGCTATGTACGTGGGTGTGTACACTTACACT

GACAACAACACTATGGTGATCCCTTACGTGAACGCTAGAGGTGCTAACCACTGCGACCAA

ACTGCTGCTATCCTGGACCAATCAGACGACTCACACCTGACTGAAGAATTCATCGAACTG

AGAAAAGCTTGGAGAGAAATCTGGGTGAACTTCATCACTACTGGTTCAGACATCATCGGT

GCTCTGGCTGCTGTGCAAGCTCAAGGTAACTCATCAAAACTGGTGAACATCGCTCAAGGT

CCTGTGAGAGGTTACAAAATGCCTGGTCTGGACGTGTTCGCTTTCTACTCAATCCCTTAC

GCTAAAGCTCCTTCAGGTAGAGACAAATACAAATCACTGTCACTGCCTCCTCCTCTGCCT

GCTCCTACTTGGACTACTCCTTTCGAAGCTACTGAAAAAAACGTGCTGTGCCACCAATTC

AACATCTTCCCTCCTGGTATGTTCGAATACGTGGAAGACTGCCTGGTGGTGTCAGTGTAC

ACTCCTGACACTAACGCTACTAACCTGCCTGTGGTGGTGGTGATCCACGGTGGTGGTTTC

CAATCAGACTTCGGTAACTACGAATCACCTTCACAACTGGTGGACCAAGGTAAAAACATC

ATCGGTGTGACTCTGAACTACAGACTGGGTCCTATCGGTTTCCTGTGCCTGGGTACTCCT

GACGTGCCTGGTAACGCTGGTATGAGAGACCAACTGGCTGCTCTGAAATGGGTGCAACAA

AACATCGCTGCTTTCGGTGGTAACCCTAACGACGTGACTATCGCTGGTTGCTCAGCTGGT

GGTGCTTCAGTGGACCTGCACATGCTGTCAAAAGCTTCAAGAGGTCTGTTCCACAAAATC

ATCGGTCAATCAGGTGGTAACATCGGTGCTTTCGCTGTGCAAGTGGACCCTACTGCTTCA

GCTAGAAAACACGCTAGAGACATCGGTTACACTGGTCCTGACACTCTGGAAGGTGTGGAA

GAATTCTACAAATCAATCCCTTACGAACAACTGCTGTCAAAAGACCTGCACGAAAACAAA

GACGTGTCAATCGTGATGGCTCCTTGCAAAGGTGACTACATCAGATACCCTGTGCTGTAC

GGTTGGGCTGCTATGGAAGGTATCTTCAGACTGGAATACTTCGAAAACTGGAAAAAAGAA

ATGAACGAAGACTTCACTAACTTCATGCCTACTGACCTGCAATTCAACAACGAAGAACAC

AAAAGACAAGTGTCAGACAAAGTGAAACAATTCTACTTCGGTGGTAACGTGACTAACGAC

AACATCATCAGATTCGTGGACTACAACTCAGACGTGATGTTCAACGTGGCTATGCAAAGA

GCTGTGACTATGCAAGTGGAAAACGGTCACAACGCTATCAACCCTACTCCTGACGGTAAC

ATCCCTCCTCTGTTCCCTAAATGGGAAGCTGCTTCAGCTAACAGAACTCCTTGCATGGAC

ATCGGTAACCCTATCAGAATCTACCCTGGTCCTTTCGACCTGGAAAGACAAAAATTCTGG

GACGAAATCTACGACCAATACAAAAAAGAACCTGTGCCTCCTTACGACTTCAACGACCAA

TGC

34

>Contig5480

ATGAAACTGCTGCTGTCAGTGGCTCTGGCTTTCGTGGTGGTGCAAGCTCAAAACGACGCT

TCAAGACTGGTGAACATCAAACAAGGTCCTGTGAAAGGTTACAAAATGCCTGGTCTGGAC

GTGTTCGCTTTCTACTCAATCCCTTTCGCTAAAGTGCCTACTGGTGCTGACAAATACAAA

ACTAACCTGCCTGTGTTCGTGTACATCCACGGTGGTTCATTCGCTTCAGAATACGGTAAC

ATCGAACCTCCTGGTCAACTGGTGAACCAAGGTAAAAACTTCGTGGGTGTGACTTTCAAC

TACAGACTGGGTCCTATCGGTTTCCTGTGCCTGGGTACTCCTGACGTGCCTGGTAACGCT

GGTATGAGAGACCAAGTGACTGCTCTGAAATGGATCCAACAAAACATCGCTGCTTTCGGT

GGTAACCCTAACGACGTGACTATCGCTGGTTGCTCAGCTGGTGGTGCTTCAGTGGACCTG

CACATCCTGGCTAAAGCTTCAAAAGGTCTGTTCAAAAGAGTGATCGGTCAATCAGGTGCT

AACATCGGTGCTTTCGCTGTGCAAGCTGACCCTACTAGAAACGCTAGAGTGTACGCTAAA

TCACTGGGTTACAACGGTACTGACTCACTGGAAGCTATCAGAATCCTGCAAAACTCATTC

ATC

35

>Contig3114

ATGTTCGGTGCTCAATGGTGCCTGAGAGTGGTGGTGCTGGGTGGTTGGGTGTCACAAGTG

GCTGGTGTGGTGGGTGGTGGTCCTGCTGCTCCTCCTGAACCTGACGCTGCTGTGGTGTTC

ACTCAAAGACACGGTAGATCAGCTAGAATCGAAGGTCTGAAAGACAACACTCTGGGTTAC

TACTCATTCTTCGGTCTGAGATACGCTGAACCTCCTCTGGGTGCTAGAAGATTCCAAAGA

CCTCTGAGAAGATTCCTGGCTGGTGAACAAAACGCTACTGTGCCTTGCTCAATCTGCCCT

CAACCTGACCCTAACTTCCCTAAAAGAATCATCGGTCACGAAGACTGCCTGTGCCTGAAC

GTGTACACTCCTAAAATGCCTGGTGACGAAAGAGGTTCACCTGTGGTGTTCTTCATCCAC

GGTGGTAACTACAGATCAGGTTCAGCTACTCCTTACGGTTCAAAAACTACTNNNGAATTC

TTCGGTGGTGACCCTACTAGAGTGACTATCATGGGTCAAGGTTCAGGTGGTTCAGCTGCT

TCAGTGATGGCTCTGTCATCAGAAGGTAGATCAGCTCACGGTGTGGTGGCTCTGTCAGGT

ACTGCTCTGTCACCTGGTACTGTGAGATCAGACCCTGAAATGCACTCAAGAAAACTGGCT

AAAAGAACTGGTTGCCCTGAAACTCCTGTGGAAAGACTGGTGCTGTGCCTGAAAAAACAA

TCAGCTGAAAAAATCGTGATGGCTGACTCAGAAATCGCTTCAGACCTGGTGGACACTAAA

AAATTCCTGGAAGAAATCATCGGTAGAAACGGTGCTGGTGTGAGAGTGGAAGGTACTGAC

GACAAAAGAGGTCTGCCTCCTCTGGTGACTGAACAACCTTCAAACCACCTGAAAAGAAGA

AACAGACTGTCACACTGCCTGCAACTGCACCTGCCTAAACAATGCGTGCAATTCTGCAGA

AGAAAATACGCTAACGAACTGTCAAACCTGATGATGAACGTGAAAAACTTCATCAAAAAA

GACATCATCGGTGGTCTGAACGGTGTGGTGGAAGGTGTGGAAGGTCTGCTGCCTGTGAAA

AAAGCTGACCTGAAAAACATCCTGCCTCTGGGTGACTACGACGAATACTACGAAAGACTG

TCACAAGCTGCTAACGCTAACATCCTGGAAGGTCTGACTGCTATCGCTAGAGACACTGGT

ACTCACTGCCTGATCTCAAGAAGAACTAGAGTGTTCAGATCAGAAAGAGGTAGACCTGGT

ATCCCTCTGCAACTGAGAGTGTGCGGTAAACCTTACCAAGGTCTGTTCCTGCTGGCTAGA

TCAACTCCTTCATCACAAGACTCAGACGCTGACTCACTGAACGAATCAGACAACTCAAAC

TCACTGTACCAACCTAAAAACTCAGGTCCTTCACACGGTGACGAACTGGCTTACATCTTC

GAACCTCTGGACGTGCAAGGTAAATCAGTGGAAAAAGAAGCTTCAATCGGTGACTCAAGA

GTGAGAAGATCATTCGTGTCACTGATCGCTAAATTCACTCACGGTCTGCAAAAAACTACT

AACGCTACTAACCTGTTCAACCTGATCCCTTTCTCACTGGACAACAACGCTTTCATCAAA

ATCACTGACGAAATCAAACTGGAAGAATTCCAATCACAACTGGGTATCATCTTCAGATTC

TGCCAAATGGGTTTCTGGGGTAACATGGTGGACAGAATCGCTGGTTCACTGTGCACTAAC

GAAATCAAAGAACTGCTGCAACTGAAACTGCCTCTGGCTGACCTGGGTGTGACTCAACTG

AAACCTATCGCTGGTCTGGCTCAACAAGGTTCAAAACCTAAACCTAACTCAATCGACAAC

ATCCCTATCATCAACCTGAACCCTTTCGGTGGT

36

>Contig11205

ATGAGATGGGTGAACAAAAACATCGCTAACTTCGGTGGTGACCCTAACAACGTGACTATC

TTCGGTCAATCAGCTGGTGCTGCTTGCGTGACTTTCCACTGCGTGTCACCTATGACTAAA

GGTCTGTTCAAAAGAGCTATCGCTCAATCAGGTTGCTTCGCTAACTGGTGGGCTCAAACT

AACAGACCTAGAGAAAGATCAGAAGCTCTGGCTAGATCACTGGGTTGCCACTCAAAAGAC

GACAAAGAACTGTACGAATTCTTCAAAAACCAACCTAAAGAAAACCTGGTGGAAGCTCAC

CTGCCTATCACTATCAAAGAAAACGACTGCGACAAATACGAAACTAAAAACTCAGTGGTG

TCAGAAAAACTGTTCCCTAACGTGGAACCTTACTTCACTGGTAACATCTTCGACGCTCTG

AAAACTGACATCCACGAAGGTGTGGAACTGATGATCGGTTACAACGAAGACGAAGGTGTG

ATCAACCTGGGTGTGTCACTGATCCTGAAAAGACAATACTTCAAACTGACTCTGTCATGG

AACATCCTGTTCCAAGACTTCTCATGCACTACTGTGGTGCTGACTATCAACTGGATC

37

>Contig11205 (Resistance)

ATGCAAGTGAAAACTACTGAAGGTCTGCTGGAAGGTGAAATCGTGAACAACGACATCCTG

GGTACTTACTACTCATTCAAAGGTATCCCTTACGCTGCTCCTCCTCTGGGTGACCTGAGA

TTCAAAGCTCCTCAACCTCCTAAACCTTGGGAAGGTGTGAGATCAGCTAAAGAACACGGT

TCATCATCATACCAATTCAACAACTTCCTGACTAAACTGGAAAAA

38

>Contig13336

ATGAACAGACACATCTCAAAAAAAGTGAACTCAAACAAAGCTTTCAACCTGAAAGAAAAC

CAAAAAGACCAACCTTCAGACGACAACTACGAAGACCACGACCCTGTGCAAACTTCAGAA

AACAGAGACTACACTAAAGTGAACTTCGACCACAACCCTTACTACAACGAACACTTCGTG

CACAACAAATTCTACAACTCAGAAAACTCAGCTTACTCAAACTACGAAGACTACAAAAAC

TTCTTCCTGAACCCTTCAAACCCTATCCCTAAATCAAACGTGAAATTCAAAATCTCATCA

AGAATCGTGCAAACTAAATACGGTAAACTGCAAGGTATCGTGCTGGCTATGGACGAACAC

AGATACCTGTCACCTCTGGAAGTGTTCCTGGGTGTGCCTTACGCTACTCCTCCTGTGGGT

TCAAACAGATTCTCACCTACTAGAACTCCTTCACCTTGGGACGGTGTGAGAGTGTCAGAC

AGACCTGGTCCTGCTTGCCCTCAAAAACTGCCTGACCTGGACGACGAAAGAACTATCCTG

GAAAAAATGCCTAAAGGTAGAGTGGAATACCTGAAAAGACTGATGCCTTACCTGAAAAAC

CAATCAGAAGACCTGGACGAAACTAAACTGGCTCTGCCTGTGCTGGTGTACATCCACGGT

GAATCATACTCATGGTCATCATCAAACCCTTACGACGGTGCTGTGCTGGCTTCATACACT

GACCTGATCGTGGTGACTCTGAACTTCAGACTGGGTGTGCTGGGTTTCCTGAACGCTAAC

CCTGCTCCTAACTCAAAAGCTAGAGTGGCTAACTACGGTCTGATGGACCAAATCGCTGCT

CTGCACTGGGTGCAACAAAACATCGCTCTGTTCGGTGGTGACCCTACTAACATCACTCTG

ATGGGTCACGGTTCAGGTGCTGCTTGCATCAACTTCCTGATGATCTCACCTACTGTGATG

CCTGGTCTGTTCCACAGAGCTATCCTGCTGTCAGGTTCAGCTCTGTCATCATGGGCTATC

GTGGACGACCCTGTGTACTACTCACTGAAACTGGCTAAACACATGAACTGCTCAATCCCT

GAAGACCTGACTAAAGACCACGAAGTGATCGTGGACTGCCTGAGAGAAGCTTCAATCGAA

GAACTGCTGTCAGCTGACATCTCACCTCCTAACTTCCTGACTGCTTTCGGTCCTTCAGTG

GACGGTGTGGTGATCAAAACTGACTTCGGTAAAGACTTCCTGACTATGTACTCAACTGGT

GACTTCCCTTCATTCGGTCCTCTGAACAACATGAACATGAACCTGAACGTGCACAAAAAA

AGATCAGACTCAGGTAGAAGACTGTTCCAAAACAAATACGACCTGCTGTTCGGTGTGGTG

ACTTCAGAAGCTCTGTGGAAATTCTCAGCTCACGACGTGCAAAACGGTATCGAACCTGAC

AAAAGAGACAGAATGCTGAGAACTTACGTGAGAAACGCTTACACTTACCACCTGTCAGAA

ATCTTCTACACTGTGATCAACGAATACACTGACTGGGAAAAAACTGTGGAAAACCCTATC

AACACTAGAGACGCTACTGTGGCTGCTCTGTCAGACGCTCAATACGTGGCTCCTCTGGTG

CAATCAGGTGACCTGCTGTCAGGTGGTCCTAAACCTGCTCTGTCAGAAGAAGACGGTCCT

AGAAGACCTACTAAAACTTTCTTCTACGTGTTCGACTACCAAACTAAAGACGGTTACTAC

CCTCAA

39

>Contig4653

ATGGTGCAAGTGACTGTGAACGAAGGTATCCTGGAAGGTGAACTGGTGAACTCAAAATAC

GGTGAACCTTTCTACTCATTCAAAGGTATCCCTTACGCTGAACCTCCTGTGGGTGACCTG

AGATTCATGGTGGAAGTGCTGGGTTTCCTGTGCCTGGGTACTAAAGACATCCCTGGTAAC

GCTGGTATGAAAGACCAAGTGGCTGCTCTGAGATGGATCAACAAAAACATCTCACACTTC

GGTGGTGACCAAAACAACGTGACTATCTTCGGTTGCTCAGCTGGTTCAATGTCAGTGACT

TACCACCTGGTGTCACCTATGACTAAAGGTCTGTTCAAAAGAGCTATCGCTCAATCAGGT

ACTTCAACTTGCTGCATCGGTATCATGACTCACCCTAGAGAAAGAGCTCTGGCTCTGGCT

AAAAAACTGGGTCTGAACTCAGAAGACGACAAAAAATTCATGTCATTCTCAAACCTGTAC

CACCTGATCGACCTGCTGGAACTGAAAAGACCTGAAGAAATCTTCGACGACACTGAAACT

TTCTTCCACGGTGACCCTTGCACTGTGCTGAGAAACGGTATCCACGAAGGTGTGGAAGTG

ATCACTGGTTACACTTCAGACGAAGGTGTGCTGGCTATCGGTGCTGCTCCTAACATCGAC

GAAATCTTCAGACTGATCAACACTTTCAAAGACTACGTGGTGCCTAAACCTATCGCTGAA

AACTGCACTTCAAAAATCCAACTGGAAGCTGGTAGAAAAATCAACAAATTCTACTTCGAC

AACAAAAACGTGTCAATGGACTGCATCGACGACCTGATCAAATTCTACTCAATGTACCTG

TTCACTTTCGGTACTTACCAATGGGCTAAAATCTGCTCAGGTCTGAACAGAAACAAAGTG

TACCTGTACAAATTCACTTGCAAAACTGAAAGAAACATCTTCGCTAAAGTGCTGGGTGCT

CAAAAAGTGATCGGTTCAAAAAAAGTGGTGTCACACGCTGACGACGTGACTTACATCTTC

CCTATGAAAGACATGTCAAAACCTATCGACAAAGAATCAGAAACTTTCAAAATCATCGAC

AGAATCACTACTCTGTGGACTAACATGGCTAAATACGGTGCTAAAATGCAAGTGAAAATC

ACTGACGGTATCCTGGAAGGTGAAACTGTGAACAACGAAATCGGTGGTACTTTCTACTCA

TTCAAAGGTATCCCTTACGCTGCTCCTCCTCTGGGTAACCTGAGATTCAAAGCTCCTCAA

CCTCCTCTGCCTTGGGAAGGTGTGAGATCAGCTAAACAACACGGTGACTTCTCATACCAA

TTCAACTTCATGGTGAGAGCTATCGAATCAGGTTCAGAAGACTGCCTGTACCTGAACGTG

TACACTCCTAACACTAAACCTTCACAACTGATGCCTGTGATGATCTGGATCCACGGTGGT

GCTTTCTGCTGCGGTTCAGGTAACGACGACGTGTACGGTCCTGAATACCTGATCAGAAAC

GACGACAAAGAACTGTACGAATTCTTCAAAAACCAACCTAAAGAAAACCTGGTGGAAGCT

CACCTGCCTATCACTATCAAAGAAAACGACTGCGACAAATACGAAACTAAAAACTCAGTG

GTGTCAGAAAAACTGTTCCCTAACGTGGAACCTTACTTCACTGGTAACATCTTCGACGCT

CTGAAAACTGACATCCACGAAGGTGTGGAACTGATGATCGGTTACAACGAAGACGAAGGT

GTGATCAACCTGGGTGTGTCATTCGACATCAAAAAAACTATCTTCCAAGCTAACACTTTC

GTGGAATACTTCGTGCCTAGATTCCTGGTGTACTACTGCTCAACTGACAACCAACTGGAC

ATCGGTAAAGCTATGAAAGAATTCTACCTGAAAAACGAAATCCTGTCAGAAAACAACCTG

GAACCTCTGGCTAAATACTTCGCTGCTGACATGTACAAATTCGGTCTGTACACTCTGGCT

AAATACTTCTCAGTGAAAAACAAAGTGTACTTCTACAAATTCACTTGCAAAACTGAAAGA

AACTTCTTCTCAACTATCCTGGGTGTGTCAAAATACTTCGGTAACAGACCTCTGGTGGGT

CACATCGACGAAGTGCCTTACCTGTTCCCTGTGAAATCAATCTCACAAAGAGTGTCATCA

GAAACTCAAAAATCAATCAACACTGTGTCAAAACTGTGGACTAACTTCGCTAAAAAAGGT

ACTCAAGACCTGCTGGGTATCATCATCCACCCTACTCCTGACGCTACTCTGGGTGTGAAC

TGGAGACAATTCAGAGCTGACATGCTGCTGACTTGGATCTCAGGTATCCTGCTGTCAGAA

GAATCAGACCCTACTAGAAAATCATCAATCCTGGGTGAAAACCTGAGACAAATCCCTACT

GCTTACGGTCTGCCT

40

>Contig4653

ATGCAAGTGAAAACTACTGAAGGTCTGCTGGAAGGTGAAATCGTGAACAACGACATCCTG

GGTACTTACTACTCATTCAAAGGTATCCCTTACGCTGCTCCTCCTCTGGGTGACCTGAGA

TTCAAAGCTCCTCAACCTCCTCAACCTTGGGAAGGTGTGAGATCAGCTAAAGAACACGGT

TCATCATCATACCAATTCAACTTCCTGACTAAAACTAGAGAAATCGGTTCAGAAGACTGC

CTGTACCTGAACGTGTACACTCCTAACACTAAACCTTCAGAACCTCTGCCTGTGATGATC

TGGATCCACGGTGGTGCTTTCTGCTGCGGTTCAGGTAACGACGACATCTACGGTCCTGAA

TTCCTGATCAAACACGACGTGAGAAGAAGAATGTACCCTGGTAACGCTGGTATCAAAGAC

CAAGTGGCTGCTATGAGATGGGTGAACAAAAACATCGCTAACTTCGGTGGTGACCCTAAC

AACGTGACTATCTTCGGTCAATCAGCTGGTGCTGCTTGCGTGACTTACCACTGCATCTCA

CCTACTACTAAAGGTCTGTTCAAAAGAGCTATCGCTCAATCAGGTTCAATCATCAACTGG

TGGACTCAAGGTTTCAGACCTAGAGACAGAGCTGAAGCTCTGGCTAGAAAACTGGGTTGC

AACTCAAAAGACGACAAAGAACTGTACGAATTCTTCAAATCACAACCTGTGGAAAACCTG

GCTGAAATCCAAGTGCCTCTGACTTACAAAGAATACCACTGCGACAAATACGAAACTCAA

TTCTCACTGGTGGTGGAAAAAGAATTCCCTAACGTGGAATCATACTTCACTGGTAACATC

ATGGAAGCTCTGAAAACTGGTATCCACGAAGGTGTGGAACTGATCATCGGTTACAACGGT

GACGAAGGTATCATCAACGTGGGTGTGTCATTCGACATCAAAAAAACTATCTCACAAGCT

AACACTTTCCTGGAATACTTCGTGCCTAAATTCCTGATCTACCACTGCTCACCTGACCAA

CAACTGGAAATCGGTAAAGCTATGAAACAATTCTACATGAAAAACGACATCCTGGTGGAA

TCAAACCTGGAACCTCTGGTGAAATACTTCGCTACTGACATGTACAAATTCGGTCTGCTG

ACTATCGCTAAATACTTCTCACTGAAAAACAAAGTGTACTTCTACAAATTCACTTGCAAA

ACTGAAAGAAACTTCTTCTCAAAAATCATGGACGTGGCTAAATACCTGGGTAACAGACAA

GTGACTGGTCACATCGACGAAATCCCTTACCTGTTCCCTGTGAAATCAATCCACCAAAAA

GTGGGTTCAGAAACTCAAAAAAACCCTACTCCTGACGCTACTCTGGGTGTGAACTGGAAA

CAATTCAGAGCTGACATGCCTTCATTCCTGGAAATCGGTAACACTCTGGTGAGAGGTGTG

GCTCCTGACAAAGAAGAACTGACTTTCTGGGAAGGTATCTTCGAAAAATACCTGCCTTCA

ATGGTGTTCCACAAAGAAACTTCA

41

>Contig11331

ATGGGTAACATCCTGTCATTCATGGTGTACAGAATGCCTACTCTGCCTCAAGCTCTGACT

GACTCAAGATCAAAAAGATACAAATGCGTGAGACCTCTGGGTGTGGGTTTCGAAGGTACT

GAAGACTGCCTGGTGGCTAACATCTTCACTCCTTCACTGGGTGAAGCTTCATCACTGCCT

GTGATGGTGTGGGTGAAAGGTAGAGAATTCGACAGAGTGTACGAAAACGAACTGTCATTC

AAACACTTCGTGGAAAAAGACGTGATCGTGGTGTCACTGAACTTCAGAGAATCAATCTTC

GGTTTCCTGTGCCTGGGTACTGAAATCGCTCCTGGTAACGCTGGTCTGAAAGACATCATC

GCTGGTCTGACTTGGATCAAAGAAAACATCTCAGGTTTCGGTGACCTGGTGCACGGTGCT

ATCACTCAATCAGGTAACGCTCTGGCTCCTTGGGCTGTGTCAAGAGACAACCTGGCTTCA

GCTGTGAAAGTGGCTGAAGCTCTGGGTCACACTGTGAACACTATCGAAGAACTGTCAGAA

GTGTTCACTAGAGTGTCAGTGCCTGCTCTGATGGCTATCATCAACGAATTCGACCTGTCA

GACAACTCACTGGTGTTCGCTCCTTGCGTGGAAAGAGAAGAACTGGCTGAACCTGCTTTC

CTGGCTAAAACTCCTTTCGAAATCATCTCACAAGGTAACTTCCTGGACATCCCTTTCATC

ACTGGTCTG

42

>Contig2269

CTGGCTGTGATGGTGTGGATCCACGGTGGTGCTTTCACTATGGGTTCAGGTAACGCTTTC

CTGTACGGTCCTGACCACCTGGTGTCAGCTGGTGTGGTGCTGGTGACTCTGAACTACAGA

CTGGGTGCTCTGGGTTTCCTGTCACTGGAAAACGACGAAGTGCCTGGTAACATGGGTCTG

AAAGACCAAGTGATGGCTCTGAGATGGGTGAGAGACAACATCGAAGCTTTCGGTGGTGAC

TCAACTAGAGTGACTATCTTCGGTGAATCAGCTGGTGCTGCTTCAGTGCACCTGCACATG

CTGTCAAACGCTTCAAAAGTGTGCACTGTGAAATACGAAAAACAATTCCAAAGAAACGCT

AACATCATCTTCAAAAAATACCTGCTGAAA

43

>Contig9036

ATGCCTAGAGTGCAAGTGGAACAAGGTGAACTGGAAGACAGACTGAGAGCTCCTGAACCT

CCTGAACCTTGGACTGGTGTGAGAGACGCTTCACAACACGGTCCTGTGTGCCCTCAATAC

AACGAAAGACTGGACAGAATGGAAGAAGGTTCAGAAGACTGCCTGTACCTGAACCAAAAC

CCTTACCCTGTGATGATCTGGATCCACGGTGGTTCATTCTACACTGGTTCAGGTAACTCA

GACTTCTACGGTCCTGACTTCTTCATGGCTCACAACATCATCCTGGTGACTTTCAACTAC

AGACTGGAAGTGCTGGGTTTCCTGTGCCTGGACAACGAAGACGTGCCTGGTAACGCTGGT

CTGAAAGACCAAGTGGCTGCTATGAGATGGGTGAAAAAAAACGTGCACGCTTTCGGTGGT

GACACTGAAAACATCACTATCTTCGGTTGCTCAGCTGGTGGTGCTTCAACTTCACTGCCT

TTCTCAATCAAAAACTCAAAAGGTTGCTCAACTAAAAGATACGCTAAAGCTTCACTGGGT

AAACAACTGGGTATCGAAACTGACGACGAACAAGAACTGCTGCAATTCCTGAGAAACTCA

CCTGTGTCAGCTCTGGTGAAAATCAAACTGCCTCCTCTGAACATCACTGTGAACAGAGAC

ATCACTGACGGTCTGACTTTCATCCCTGTGATCGAAAAAAGAGACCTGACTGTGGAAAAA

TTCATCTCAGAACCTCCTCCTGAC

44

>Contig9036

ATGTACAGAGAATCAACTCCTGTGTACCTGAACGAATTCACTGCTGAAACTGAAAGAAAC

TACACTAAAAAACAATACAACATGGACTCAGTGAAAGGTGTGTGCCACGCTGACGAACTG

CACTACCAATTCCACGTGACTTGCCTGCCTGTGCCTCTGTCAGAAGAATCAGCTAAAATC

ATCAAACAATTCGTGAAACTGTGGGCTGACTTCGCTGCTACTGGTAACCCTACTCCTGAC

GAATCAAACCCTAACGTGCCTTGGAAACCTTTCACTGAAACTGAAAGAAACTGCCTGGTG

ATCGACAAAGAACTGAAATGCATCAGACTGGACAACGCTAAAAACATGAGATTCTGGGAA

GAACTGTACGAAGAAACTATCCTGAACATCGAAAACTCAAACAAAATCGAA

45

>Contig5484

ATGCTGCCTAGACTGCTGGGTGACACTGACTTCCAACTGCTGAAATCAACTACTGGTGGT

GACGGTAGAATCCAACCTATCGCTTGCGCTCTGGTGGCTCACAAACCTAAAATGAAAACT

CCTATGATCCCTACTACTAAAATCTCACTGAACCTGACTAACGCTTTCCAACCTGTGCCT

GACGACTTCCTGCCTGTGGCTGAACAATACTCACCTCTGCCTCTGGCTACTGCTCTGTCA

GCTACTAAACAACCTATGATGCAACTGGACCTGCTGCTGGGTTCAACTGACCTGGAAGAC

ATCAACCACTACGACAACAAATACCAAGCTCTGCTGGACCTGAACCAATCATACATCGCT

GACTACACTAACAACTACAGAATCCCTGACATCCTGAAAATGTTCTCACTGGGTCAATCA

GAAATCCTGCCTGCTCTGCTGCAAGCTATCAGATGGGAATTCTGGTCAATCAAATCATCA

AAAGAACCTTCAAAAGAAATCCTGGAAGCTGTGGAAGGTCTGGCTAGAATGGAATCATCA

GCTAAATGGTCAGTGGGTTCAGCTCTGCTGGCTGCTAGACTGGCTAGAAAAATCTCAAGA

CTGTACGTGTACAGATACCTGCAACCTTCAATCTCAGACCTGTACGGTAGAGACATCAAC

TTCACTGGTGCTGTGCACGGTTCAGACCTGATCGCTCTGCTGGGTGACTCACTGATGCTG

CAAATCGCTAGAAGAAGATCAACTCCTGAAGAAAAAAGAATCTCACTGATCTTCAGAGAA

TACGTGCTGAACTTCATCAAATTCGGTTCACCTGAACAAGAATCAAAATGGCAAAGATAC

AAAATCGGTGAAGCTCACGTGCACGGTGTGCAATCAGAAGAAATCAGATACAACAGAAGA

TCAGTGTCAAGAGACACTGCTTTCTGGCTGCAATACCTGCCTCAACTGCACGGTTCACTG

ATCTCAGTGTCACACGCTGAAGAACTGAACTCACAAAAA

46

>Contig12328

ATGAAATGGCTGGCTCTGATCACTCTGATCGTGGCTAACGTGGTGCAACTGCCTTCACCT

CTGGTGACTACTACTTCAGGTGTGGTGAGAGGTAGAGTGGGTGAAACTGGTAAATTCTAC

CAATACCTGGGTATCCCTTACGGTACTGTGGACGCTAGAAACAGATTCCAAGCTCCTCTG

CCTCCTCCTAAATGGTCAGGTATCTTCGACGCTGTGGACGAAAACACTTGGTGCCCTCAA

AGATACGGTTCAACTGTGATCGGTCACACTGACTGCCTGAAACTGAACGTGTACTCACCT

ATCTCAGCTGAAGCTGGTCTGAGACCTGTGATGGTGTTCGTGCACGGTGGTTGCTTCTTC

GAAGGTATCTCATCACCTTTCCTGTACGGTGGTGACTTCTTCGCTGAACACGGTGTGGTG

TTCGTGGGTATCAACTACAGACTGAACGTGGAAGGTTTCCTGTGCCTGGGTATCAAAGAA

GCTAGAGAATGCAGACCTCAAAGACCTGACTCATCATCAAAAGTGGACCAAAGA

47

>Contig12328

GCTCCTGGTCCTGAACCTATCTGGGACGGTGTGTTCGAAGCTATCGACGAAACTGTGAGA

TGCACTCAAGCTGTGGTGCTGGAATCAAAATACTCAATCGGTGAAGAAAACTGCATGAAA

CTGAACGGTTTCCTGTGCCTGGGTATCAAAGAAGCTCCTGGTAACGCTGGTCTGAAAGAC

CAAGTGGCTGCTCTGAGATGGGTGAAAAGAAACATCAGAGCTTTCGGTGGTGACCCTGAC

AACGTGACTATCTTCGGTGAATCAGCTGGTGCTTCATCAGTGTCATACCACATGCTGTCA

CCTATGTCAAAAGGTCTGTTCCACAAAGCTATCGTGCAATCAGGTTCATCACTGGCTGGT

TGGGCTTTCCAATACAAACCTGTGTACATCGCTTCACTGCTGGCTAAAGCTATGGGTCTG

GACTCAAACGACCCTAAAGAACTGTACGAATTCTTCATCAAACAAACTGACTCAGACCTG

GTGACTAAAAGAGTGCCTAGACAAGAAAACAACATCATCATCACTGAAATCCTGTTCGCT

CCTTGCGCTGAAAAATCATTCCAAGGTCAAGAATCATTCCTGACTGAACTGCCTTACGAA

CTGCTGTCAACTGGTAACTACAACAAAGTGCCTCTGATCATCGGTGCTAACACTGAAGAA

GGTATCACTCTGATCGGTCTGGAAGAAGACAACCTGGTGGAAAAAGTGAAATTCGAAAGA

GCTCTGCCTAAAGACCTGGTGTTCCTGTCAAACGAAACTAAACAAGCTGTGGCTAACAAA

CTGAAAGAATTCTACATGGGTTCAGACGAAGTGTCAAAAGACACTAAAGTGAAACTGTCA

AAACTGTACGGTGAACCTTACATCAACTACCCTGTGATGGAAGAAACTGACCTGATGCTG

AAAACTAACGACAAACCTATCTACCACTACCTGTTCGGTTACGACGGTAGAAGAAACATC

GTGAAAAAAACTCAAAACCTGCCTCTGAGAGAAATGGACGGTGCTTCACACGCTGACGAA

CTGTTCTACCTGTTCTCACAAACTCTGATCCCTGAATTCGCTGAAAAAGTGATGATCGAC

AAAATGACTACTATGTGGACTAACTTCGCTAAATACGGTAACCCTACTCCTCACACTACT

GAAAACCTGCCTCTGCTGTGGCCTCAAGTGACTACTGACAACCAACTGGCTCTGAAAATC

GACCAAGAATTCTCAACTATCCCTCTGTGGCACTCAGACTCACTGAAATACTGGAGAGAA

ATCTACTCAAACAGAAGAAGAAGATGCGCTTGGGCTAGACTGGCTGAAGGTTCAGTGGCT

CACTCAGGTACTCACGCTCACTACCTGGGTATCCCTTACGCTACTCTGAAAGGTAGATTC

CAAGCTCCTGGTCCTGAACCTAAATGGGAAAACACTTTCGAAGCTGTGGAAGAATACATC

AGATGCAGACAAGTGGTGGCTCTGGAAGCTAAATCAACTACTGGTCAAGAAGACTGCCTG

ATCCTGAACGTGTTCACTCCTCTGGCTACTTCAGACAAACCTCTGCCTGTGATGGTGTAC

ATCCACGGTGGTGGTTACCACGAAAACTCAGCTTCAAGAATGATCTACGGTCCTGAATAC

TTCATGTCAAAAGGTGTGGTGCTGGTGACTATCAACTACAGACTGAACATCCAAGGTTTC

CTGTGCCTGGGTATCAAAGAAGCTCCTGGTAACGCTGGTATGAAAGACCAAGTGGCTGCT

CTGAAATGGGTGCAAAGAAACATCAGAGCTTTCAACGGTGACCCTGACAACGTGACTCTG

TTCGGTGAATCAGCTGGTTCATCATCAGTGTCATTCCACGTGATCTCACCTATGTCAAAA

GGTCTGTTCCACAAAGCTATCATGCAATCAGGTTCATCACTGTCAGCTTGGGGTTACCAA

TACAAACCTGTGTACCTGGCTTCACTGCTGGCTAAATCAATGGGTTTCGAATCACAAAAC

CCTAGAGAACTGTACAAATTCTTCATGTCAAAAACTGACAACGACCTGGTGATCACTAGA

GTGCCTAGACCTGAAGGTAACACTATCGTGTCAGAAATCCTGTACGCTCCTTGCACTGAA

AAACAAATCGACGGTATCGAACCTTTCCTGACTGAACTGCCTTACGACATGCTGTCAAAA

GGTAAACTGAACAAAGTGCCTATCATCATCGGTTCAAACACTCAAGAAGGTATGCTGCTG

GCTGGTCTGGACAAAGACTACTACATCGAAGCTATCAAATTCGAAAACGCTCTGCCTAAA

GACCTGGTGTTCCCTAACAACGAAACTAGAAAAATGGTGGCTGACAAACTGAAAGAACTG

TACCTGGGTGACGACGAAATCGCTCCTAAAAACATCGACAAACTGTCAAACTGCATGGCT

TCAAGAATCCTGACTATCAGAAACGTGCTGAAACTGGTGTACGTGGGTTCAAACGCTTCA

TACCACCAAGTGAACCCTTACAGATGGCTGCCTGCTAACGCTGAAAACCCTCAAGCTCTG

CTGATCGAAAAAGAATTCAAACCTTACCCTTGCGGTACTGAATCACTGTGCTTCCTGAAA

GACGTGTACTCAAAATACAGAAGAAAACTGGAACACGGTTGGTGCTGCGGTAGATCAGGT

AGATACGGTCTGTGCGCTTCAAGAAGAAGAAGATGCGAATCAGCTGCTGCTGGTTGCGAA

GACCTGTGCCCTAACGAAGGTACTCACGCTCAATACCTGGGTGTGCCTTACGCTACTGTG

GTGAAAAGATTCCAAAGATTCACTTCATCATACATCAACGGTCAAGAAGACTGCCTGACT

CTGAACATCTACACTCCTCTGCAATCATCATCAAACCTGAGACCTGTGATGGTGTTCATC

CACGGTGGTGGTTTCAGAGACGGTTCAGGTTCACCTGTGCTGTACGGTCCTAACTACATC

ATCAACCACGACGTGATCCTGGTGACTTTCAACTACAGACTGGAAATCCTGGGTTTCCTG

TGCCTGGGTATCAAAGAAGCTCCTGGTAACATCGGTCTGAAAGACCAAGTGGCTGCTCTG

AAATGGGTGCAAAGAAACATCAAAACTTTCGGTGGTGACCCTGACAACGTGACTATCTTC

GGTGAATCAGCTGGTTCAGCTTCAGTGATGTACCACCTGATGTCACCTATGTCAAAAGGT

CTGTACCACAAAGCTATCATGCAATCAGGTTCAGCTATGTCACCTTGGTCATTCCAATAC

GACCCTCTGAAAACTGCTAGAGCTCTGGCTAAACAAATGGGTTACAAAACTCAAGACCCT

CACGAACTGCTGAAAATCTTCCTGAACAAAACTGCTGAAGAACTGCTGAAAACTAGAGTG

CCTAGAAAAGACGGTGACATCGTGCTGTCAGAAAACATCTTCGTGCCTTGCGTGGAAAAA

ATCCTGCCTAAAGAAGAACAATTCCTGACTGACACTCCTTACAACCTGATGTCACAAGGT

CTGTTCACTCAAGTGCCTATCATCATCGGTCACAACAACGCTGAAGGTTACATGTTCGCT

GGTAAAGAAAACGAAACTACTATCTCAAAAATCAACATCTACAACGCTCTGCCTAGAGAC

CTGAACTTCCCTAACGACAACGAAAAAGTGGAAACTGCTAAAAAACTGCAAGCTATCTAC

ATGAACGAAAACCCTAAAGACCACATCCTGAAAATCGCTGACTACGAAGGTGACTCATCA

ATCACTTTCCCTGTGGTGTTCGCTATCGACCTGTTCACTAAAACTTCAAAAGACGCTGTG

TACGCTTACAAATTCTCATACGACGGTTGGATGAACTTCGCTAAAAAATACTTCGGTTTC

ACTCACGCTCCTGGTGCTACTCACGCTGACGAACTGTTCTACATGTTCAAAATCAAAATC

CCTGTGCTGCTGCACGAATACATCGAATGGGACATCATCCAACAAGTGACTTCAATGTGG

ACTAACTTCGCTAAATACGGTGACCCTGTGCACAGAGCTACTCAATCACTGAAATGGCCT

CCTACTAAAAAAGAAGACCTGAAACTGCTGATCATCGACAAACAATTCTCACTGGCTCCT

GTGTGGTCAAACGAAGCTATGCTGCTGTGGAACACTACTTACACTAAATACAGAAGAAAA

ATC

48

>Contig3031

ATGGGTATCCTGACTTTCGCTGTGTCATGCGCTATCTTCGGTGCTGTGCTGGGTCTGGAA

GAAACTAGAATCGTGAACACTAAATACGGTAAAGTGGAAGGTTCACTGGCTTCAAACCAA

CTGTACTACGAATACCTGGGTATCAGATACGGTGTGCCTGTGAAATTCAGATTCCCTTGC

AACGACCCTCTGGCTGTGCCTTCAGACAACGAAGACTGCCTGGTGCTGAACGTGTACTCA

CCTTTCGGTGTGCTGAACTCAACTTTCCCTGTGATGGTGTACCTGCACGGTGGTGGTTTC

GGTGTGGACGCTCCTGGTAACGCTGGTCTGAAAGACATCAGAGCTGCTCTGAGATGGATC

AAAGACAACATCGCTAACTTCGGTGGTGACGTGGACAACATCACTGTGTTCGGTCAAGGT

TCAGGTGGTGTGGCTGCTCTGTACCTGACTATGTCAGAATCAACTAAAGGTCTGTTCCAC

AAAGTGATCTCAGAATCAGGTGCTCCTTTCACTCCTCAATCATTCGACGCTTACCCTCTG

GGTACTGCTTCACAAGTGGCTAAAGCTCTGGGTGTGTCATCAGTGGACCCTGAAGACCTG

TTCAGAATCTACTCAGAAACTCCTGTGAAAAAAGTGGAAGAAGCTATCTCAAAACAAATG

AACGCTAAAAAAGTGCACGGTGTGCCTATCATCGTGGGTCTGAACACTGTGGAAGGTCTG

CCTTCAACTCTGGACTACTTCACTATCACTTCACAAATGGACAGAATCAAAAACGAAGAC

TTCTCATGCCTGGACCAAAGATCACTGATCGTGCCTAAAGACGAAAAAGCTGACGTGAGA

GAAACTCTGAAAGACACTTACTTCGCTGACATCGACTCAGACGAAACTCTGATCGGTGGT

ATCATCAACCTGAACTCAGACTTCTCATACGTGGGTCCTATGTCACTGTTCTCAGAACTG

TACGCTAACTCAACTGACAAACCTGTGTACCAATACATCTTCAACTACATCGGTAACAGA

AACCTGGGTAGACTGCTGACTAACTCATCACTGCCTGCTACTGCTAACCAAGACGAACTG

TTCTACATCTTCGAACTGGAAAGACTGCCTCTGCCTATGGACGAAGAAGACGCTAGAATG

GTGACTTTCATGACTATGATGTGGACTAACTTCGCTAAATTCGGTTCACCTACTCCTGAA

ACTGGTAACGGTCAATGGCTGCCTTACCCTCACCACCTGGCTATCGACCTGGAACCTCAA

TACGTGAACCCTCTGACTCCTGAAAGAGCTTACTTCTGGAGAGCTTTCTACCTGAAATAC

GGTGCTGAACTGCCTAAAGAAAGAGAAAAC

49

>Contig3502

TTCCTGAACGCTAACCCTATCCCTCACCTGAAAGCTAGAGTGGCTAACTACGGTCTGATG

GACCAAATCGCTGCTCTGCACTGGGTGCAACAAAACATCGCTCTGTTCGGTGGTGACGCT

GGTAACGTGACTATGCTGGGTCACGGTTCAGGTGCTGCTTGCATCAACTTCCTGATGATC

TCACCTACTGTGATGCCTGAATTCGACTTCACTCTGTTCTTCGTGCTGGGTCTGTTCCAC

AGAGCTATCCTGCTGTCAGGTTCAGCTCTGTCATCATGGGCTCTGGTGGAAGACCCTGTG

TCATTCTCAGTGCAACTGGCTAAACAATCAAACTGCACTCTGCCTGAAGACATCGTGAAA

GACCACGAACTGATCGTGGACTGCCTGAGAGAAGTGCCTCTGGACGAACTGATGTCAGCT

GAAATCTCAACTCCTTCATACCTGACTGCTTTCGGTCCTTCAGTGGACGGTGTGGTGGTG

AAAACTGACTACGCTAAAGAACTGCTGACTTTCTTCATCCCTAACGACCTGCAAGGTTTC

ACTTCAGTGTCAGGTGTGAACAACATCAAAGCTGACAAAAGATCAGGTGACAGAATCTTC

GGTATCAGAGGTGGTCAAAACAAATACGACCTGCTGTTCGGTGTGGTGACTTCAGAAGCT

CTGTGGAAATTCTCAGCTCAAGACATCCAAAACGGTTTCGAAGGTGAAAGAAGAGACAAA

ATCATCAGAACTTACGTGAGAAACGCTTACACTTACCACCTGTCAGAAATCTTCTTCACT

ATCGTGAACGAATACACTGACTGGGAAAGAACTGTGCAACACCCTATCAACACTAGAGAC

GCTGCTGTGCTGGCTATCTCAGACGCTCAATACGTGGCTCCTCTGGTGCAAACTGGTGAC

TTCCTGTCAGTGGCTAAATCATCACCTGACTCAGGTCCTAACACTTTCTTCTACGTGTTC

GACTACCAAACTAAAGACGGTGACTACCCTCAAAGAATGGGTTCAGTGCACGGTGAAGAA

CTGCCTTACCTGTTCGGTGCTCCTCTGGTGGAAGGTCTGGGTCACTTCCCTAAAAACTAC

ACTAAATCAGAAGTGGCTCTGTCAGAAGCTTTCATCCTGTACATGGCTAACTTCATCAGA

ACTGGTAACCCTAACGAAGCTCAAAGACAAGAAGGTGTGCTGCCTATCTCAAGAGAAAGA

AACAGATACAAATCAATCGTGTGGGACGAATACGACACTCTGCACCAAAAATACCTGGAA

ATCGGTATGAAACCTAGAATGAAAAACCACTACAGAGCTCACCAACTGTCAGTGTGGCTG

AGACTGATCCCTGAAATCCACAGAGCTGGTATGAAAGACGTGGTGGCTAAACACAACCTG

TTCAGAAACCACAACGACCCTGAACTGTACGACGGTCTGGTGAGACCTGACCCTCTGACT

AGAGTGAACTACTTCGACCCTACTCTGGAAATGTACAGAAAACCTATCTACAACGTGACT

CTGGACATCCCTTCAACTACTATGGACACTTACGTGACTACTTGCATCTCAGTGATGTCA

GCTAGACCTGGTTCAGCTGTGACTCAATCACACGCTCAAAACAACACTTCAGCTCAAGAC

GTGTCAAACCTGGAAGTGGCTGGTTACACTGCTTACTCAACTGCTCTGTCAGTGACTATC

GCTATCGGTTGCTCACTGCTGATCCTGAACGTGCTGATCTTCGCTGGTGTGTACTACCAA

AGAGACAAAACTAGACTGCAAGTGAAAGCTCTGCAACAACAACAAAAAAGAAACCACAAC

TCAACTTTCGACTCAGTGTCATCAAAACACCCTCACTACTTCGTGGGTCACTCACAATCA

TCATCAACTATCGTGGACATCGACCACCAAGACAAAAACGCTATCATCGCTATGACTAAC

AGAGTGCCTCACTTCACTTCAGCTAACTGCCCTAACGTGTGCCACACTGGTATCCAAATG

TCAAACATGCCTCAAAAATCATCACCTCCTACTAACAGAGGTGGTCAATGCACTACTCTG

CCTAGAAAAGTGGGTTTCTCATACCAACAAACTAACCAAATCTGCAACGCTTCAAACTGC

ATGACTCTGCCTAAAAACGCTACTTTCATGTCATCAGCTTCAAACCTGCCTGACGTGCAA

GCTCAAACTGGTCAAAACCAAGGTCCTGGTAACGGTTCAGTGCTGCCTCCTCCTTCACCT

CCTCAACACTTCCAAAAATCAAGAGTGCCTCAAGCTGCTATGTCAGAAATGAACGTG

50

>Contig3502

ATGGACCCTGTGAAAGACGGTACTGCTGTGTTCGCTTCAAGATCAACTTGGAGAGGTCCT

TACGGTAAACTGCAAGGTGTGATCCTGCCTATGGACCAACACAAATACCTGAAACCTGTG

GAAGCTTACCTGGGTGTGCCTTACGCTACTCCTCCTACTGGTTCAAACAGA

51

>genemark-Contig3502-0.8

ATGCTGAGAATCTCAGTGTCAGAAATCGTGAGAGTGTACGAACTGATGTGGCAATGGACT

GGTCACATCGTGCACAGAACTGACGTGACTAAAAACAACAGAAGACCTACTCCTTACAAA

ATGGTGCTGGTGAAAGTGATGGCTAACATGGAACTGACTACTGGTGACAGATTCAGACTG

CCTGACATCACTAACGAAACTCTGGCTCTGGAAAGAATGCCTAAAGGTAGACTGGAATAC

CTGAGAAGACTGCTGCCTAGACTGAAAAACCAATCAGAAGACTGCCTGTACATGAACATC

TACACTCCTGTGCAAGCTTCAAAAATCACTGGTGCTGCTCTGATGGAAGACAACAACTGC

52

>Contig11243

ATGAACAGACACATCTCAAAAAAAGTGGACTCAAACAAAGCTTTCAACCTGAAAGAAAAC

CAAAAAGACCAACCTTCAGACGACAACTACGAAGACCACGACCCTGTGCAAACTTCAGAA

AACAGAGACTACACTAAAGTGAACTTCGACCACAACCCTTACTACAACGAACACTTCGTG

CACAACAAATTCTACAACTCAGAAAACTCAGCTTACTCAAACTACGAAGACTACAAAAAC

TTCTTCCTGAACCCTTCAAACCCTATCCCTAAATCAAACGTGAAATTCAAAATCTCATCA

AGAATCGTGCAAACTAAATACGGTAAACTGCAAGGTATCGTGCTGGCTATGGACGAACAC

AGATACCTGTCACCTCTGGAAGTGTTCCTGGGTGTGCCTTACGCTACTCCTTCATGGATC

CAACAACTGTTCGTGGAATTCAAACAATCAATCAGAGGTGCTGTGCTGGCTTCATACACT

GACCTGATCGTGGTGACTCTGAACTTCAGACTGGGTGTGCTGGGTTTCCTGAACGCTAAC

CCTGCTCCTAACTCAAAAGCTAGAGTGGCTAACTACGGTCTGATGGACCAAATCGCTGCT

CTGCACTGGGTGCAACAAAACATCGCTCTGTTCGGTGGTGACCCTACTAACATCACTCTG

ATGGGTCACGGTTCAGGTGCTGCTTGCATCAACTTCCTGATGATCTCACCTACTGTGATG

CCTGGTCTGTTCCACAGAGCTATCCTGCTGTCAGGTTCAGCTCTGTCATCATGGGCTATC

GTGGACGACCCTGTGTACTACTCACTGAAACTGGCTAAACACATGAACTGCTCAATCCCT

GAAGACCTGACTAAAGACCACGAAGTGATCGTGGACTGCCTGAGAGAAGCTTCAATCGAA

GAACTGCTGTCAGCTGACATCTCACCTCCTAACTTCCTGACTGCTTTCGGTCCTTCAGTG

GACGGTGTGGTGATCAAAACTGACTTCGGTAAAGACTTCCTGACTATGTACTCAACTGGT

GACTTCCCTTCATTCGGTCCTCTGAACAACATGAACATGAACCTGAACGTGCACAAAAAA

AGATCAGACTCAGGTAGAAGACTGTTCCAAAACAAATACGACCTGCTGTTCGGTGTGGTG

ACTTCAGAAGCTCTGTGGAAATTCTCAGCTCACGACGTGCAAAACGGTATCGAACCTGAC

AAAAGAGACAGAATGCTGAGAACTTACGTGAGAAACGCTTACACTTACCACCTGTCAGAA

ATCTTCTACACTGTGATCAACGAATACACTGACTGGGAAAAAACTGTGGAA

53

>Contig3119

ATGAACTACGTGAAAAACTGGCTGGCTGCTTGCGTGTCATCAAGAGCTCTGCCTCTGCTG

CTGGCTGTGGAAGCTCCTAGAGCTAGATTCCTGTCAGGTTGGGCTCCTGCTGCTCCTGCT

CCTGCTAGAGCTCTGCACGCTTCAGACGCTTTCCTGGACTGCGCTCTGGCTGTGGTGGTG

GCTACTACTGAATCATACCAAAACGTGTACAGATACCACAGAAACGAAATCTTCGCTGCT

GTGAGAAACGAATACACTGACTGGGAAAAACCTATCCAACACCCTATCAACATCAGAGAC

GCTACTCTGGAATCACTGTCAGACGCTGCTGTGGCTGCTCCTGCTCTGAAAAGACTGGGT

TCAGTGACTGGTGAAACTCTGCCTTACTTCCTGGGTCTGCCTCTGGTGGGTGGTACTACT

TCAAACCCTAGAAACTACTCAAGAGGTGACGTGACTGTGGCTGAATCAGCTGTGGCTCTG

CTGGCTGCTTTCGCTAAAACTGGTGACCCTTCACCTAGAGGTGACGAAAGACACCACGAA

GGTATGACTTGGCCTAGATACGAACTGAACACTCAACAATACCTGTCAATCAAACTGAAA

TCACCTATCAGACACGAAGGTCCTTCAGAAGAACCTCTGCCTAGACACAAAATGGCTCTG

TGGCTGCACCTGGTGCCTCAACTGCACAGACCTGGTGCTGCTCCTAGACACCACCAATTC

AGATCAGTGCACCCTGACATGTTCGCT

54

>Contig8236

ATGAAAGACCAACCTTGCCCTACTATGGGTCCTGAACACATCATGATGTTCGGTGGTAAC

CCTGACTCAGTGACTCTGTCAGGTTGCTCAGCTGGTGGTGCTGTGTGCATCCACTACCTG

TCACACTACCTGAGAGGTATCGCTTTCTCAGGTTCAGCTTTCGCTTCATGGACTCACGCT

GTGAAACCTGCTCAAAAAGCTAAAACTCTGGCTGGTATCGTGGGTTGCCCTACTGGTACT

AACAGAGAACTGGTGGACCTGTACCAAACTGACCCTTCACTGCTGCCTGACCTGGAAGCT

AGATGGGAACAACTGGCTTCAAACATCTTCGAATACAACGACACTCTGCCTCTGCACCTG

AGACCTGCTGTGGCTGCTAAAATCAAACAAAAATACCTGGGTGGTAACCCTGCTCTGGGT

GACAGACTGTTCTCAGCTGAAGTGGGTAGACTGGCTGGTGTGTCACACGCTGACGACGTG

CTGCAAGTGTTCAAATTCCCTGCTCTGGACTTCGCTTCATCAGAAGACAAACAAATGAAA

AACCTGCTGATCGACATCATCTACAACTTCGCTACTAACGGT

55

>Contig11175

ATGATGTCACCTATGTCAAAAGGTCTGTACAAAAGAGCTATCGCTATGTCAGGTGTGCCT

CTGTCAGAATGGCACTCACACCTGGGTACTAAAGCTTTCTTCTTCTCATGCGCTTCAAGA

AGAAAAAGATTCTGGACTGGTAAAATCCTGGTGGAAACTCCTACTGAATCAATCATCAAC

GGTAACGTGAGAGACCAAGAACTGATCATCGGTCTGACTTCAATGGAATTCATCTACGCT

ATCCCTTGGCTGGAAGACGACCACGTGGCTAGATTCAACCTGGCTCTGGTGATCATCCTG

TTCTTCATCCACGGTGGTGCTTGGAAATCAGGTTCAGGTAACGACGACAACTACGGTCCT

GACTTCCTGATGCACTACGACATCATCCTGGTGACTATCAACTACAGACTGGACGTGCTG

GGTTTCCTGTCACTGGACACTAAAGACGTGCCTGGTAACGCTGGTAACGACCACGGTCAA

TCAGCTGGTGGTGCTTCAGTGATCTACCACATGATGTCAACTGTGTCAAGAGGTCTGTTC

AAAAGAGCTATCTCAATGTCAGGTGTGCCTCTGTCAGACTGGGCTTTCCCTTTCGAACAA

ACTACTAGAGCTTACGAACTGGCTAAAACTATGGGTCAAAACATCGACAACTCAGAAGAC

CTGCTGAACTACCTGCAAAACGTGCCTGCTCACGACCTGCTGAACACTAAACCTGCTGTG

CTGTCATCAGAAGTGTACTGGAACAACGGTATCAAAATGTTCTCATTCGTGCCTGTGATC

GAAAAAGACTTCGGTAAAGAACAATTCCTGGTGGAACCTATCAAAGAAGCTCTGGTGAAC

GGTAACATCGAAGACACTGACCTGCTGATCGGTTACACTGACCTGGAATACCTGATCGCT

GTGCCTCTGCTGGAATCATACGGTCTGGCTGACTACAACATGTTCAAAGAACTGCTGGTG

CCTAGAGAAATCCTGCTGGAAATCTCACCTAAAAGAATGCTGTCACTGGCTGAAAAAATC

CACAAAGCTTACTTCGGTAACCAATACCTGAAAAACGCTGAAAAATACAACATCCACGGT

TGCGCTCACACTGACGACCTGATGTACCTGTTCGACGCTAAACAAGAAGCTACTGCTATG

AACGTGGACTCAGACGAATACCAACTGATCAACCAAACTTGCACTCTGTTCACTAACTTC

GTGAAATACGGTTCACTGTGCCTGGTGAACCACGGTAAACTGCAAAAAAGATCATGCCTG

CTGTTCTTCATCTACGGTGGTGGTTTCAAATCAGGTTCAGGTAACGAAGACGAATACGGT

GCTGACTTCCTGGTGCAACACAACGTGATCGTGGTGACTGTGAACTACAGAATGGACGCT

CTGGGTTTCCTGTGCCTGAACATCAAAGAAGTGCCTGGTAACGCTGGTATGAAAGACCAA

TCACTGGCTCTGAAATGGGTGCACGACAACATCCAATACTTCGGTGGTAACCCTTACGAA

GTGACTATCATGGGTCAATCATCAGGTGCTGCTTCAGTGATCTACCACATGATGTCACCT

ATGTCAAGAGGTCTGTTCAAAAGAGCTATCTCAATGTCAGGTGTGCCTTTCTGCGACTGG

GTGATCCCTCTGGAACCTCAAAAAAGAGCTTTCGAACTGGGTAAAGCTCTGGGTAAAAAC

ACTAAAAACCCTTACGAACTGCTGGACTTCCTGCAATCAGTGCCTACTGAACAACTGGTG

AACACTGCTCCTCTGGTGATCTCACAAGAAATCATCTGGAACTTCCCTTTCAGAATGTTC

CCTTTCGTGCCTGTGGTGGAAAACGACTACGGTCAACAAAGATTCCTGGCTGAATCAATC

AAAGACGGTCTGACTATGGGTAACGTGGCTGACGTGGACCTGCTGATCGGTTACACTTCA

AACGAAAACCTGATGGTGATCCCTTTCCTGGAATCATACTACCACACTTACGACACTTAC

TCAGAACTGTTCGTGCCTAGAGAAGTGGCTCTGGAAATCTCACCTAAAGAAGTGCTGTCA

CTGGCTAAATACATCCTGAGAAGATACATCGGTAACCAACCTATCTCACTGTCAACTATC

CCTCAAATCGTGCAATGCCTGGACGACTACACTTACAAAAAAGAACCTCTGGTG

56

>Contig9306

GCTCCTCAAAAACTGAAACCTTGGGAAGGTGTGAGATCAGCTAAAGAATTCGGTCCTGTG

TGCTACCAATTCGACCTGGTGACTGAAAAAATCTTCGGTTCAGAAGACTGCCTGTACCTG

AACGTGTACACTCCTGAAATCACTCCTAAAGAACCTCTGCCTGTGATGGTGTGGATCCAC

GGTGGTGGTTTCATGTCAGGTTCAGGTAACGACGACGTGTACGGTCCTGAATTCCTGATC

AGACAAGGTGTGATCCTGGTGACTCTGAACTACAGACTGGAAGTGCTGGGTTTCCTGTGC

CTGGACTCAGAAACTGTGCCTGGTAACGCTGGTATGAAAGACCAAGTGGCTGCTCTGAGA

TGGGTGCAAGAAAACATCAAAAACTTCGGTGGTGACCCTAAAAACGTGACTATCTTCGGT

GAATCAGCTGGTGGTGTGTCAGTGACTGGTCACCTGCTGTCACCTATGTCAAAAGGTCTG

TTCAAAAGAGCTATCGCTCAATCAGGTGCTCTGACTTGCTGGTGGTCAAACCCTTACGGT

GCTAGAAAAGCTGCTCTGGGTCTGGCTAGAAAACTGGGTTGCAACTCAGAAGACTCAAAA

GAAATCTACGAACTGATGGAACTGCAATACGGTATCACTTCAGAAAACAAATACTACGGT

AAAGAAAGATTCCTGCCTGGTGAACCTTACGAACTGCTGAGAGAATGCGTGCACGAAGAC

GTGGACATCATCATCGGTTACAACGAAGAAGAAGGTCTGTTCTGGTCAAAAACTGGTGTG

GACGTGCCTGCTGTGATCGACAAAGTGAAAAGATTCGACGACTTCCTGGTGCCTAGACCT

GTGGGTCTGCACGCTTCAGAAATCACTAAACTGGAAATCGGTGAAAACGAAACTGTGCTG

CACAGAAAAGGTAAATTCAAAAACCCTACTCCTGACGAATCACTGGGTGCTAAATGGAAC

CCTTTCGACTCAGAAGACCAACAATACCTGGACATCGGTAACCAACTGAAAGCTGACTCA

TTCCCTGAAAAAGAAGAACTGGAATTCTGGGAAAACATCCTGAAAGAAGTGTACCCTAAA

CACGTGTTCTCAAAAAAC

57

>Contig7340

ATGAAAAACTCAGCTCAAGCTAAAGGTGACTCAAAAATCGTGAACATCGGTCAAGGTCCT

GTGAAAGGTTACAGAGTGTCAGAACTGGACGTGTTCGCTTACTACTCAATCCCTTACGCT

ACTGCTCCTAAAGGTGTGGACAAATTCAAAGCTCCTCTGCCTCCTCCTGTGTGGACTGAA

CCTTTCGAAGCTGTGACTCAAGAAGTGCTGTGCCTGCAACACGACGTGTTCAACAAAACT

ATCAAATTCGAAGAAGACTGCCTGGTGGCTTCAGTGTTCGCTCCTGACACTGACGAAAAA

AACCTGCCTGTGCTGGTGATCATCCACGGTTTCCTGTGCCTGGGTACTCCTGACATCCCT

GGTAACGCTGGTATGAGAGACCAACTGGCTGCTCTGAAATGGGTGAGACAAAACATCGCT

GCTTTCGGTGGTAACCCTGACGACATCACTATCGCTGGTTGCTCAGCTGGTGGTGCTTCA

GTGGACCTGCACATGCTGTCAAAAGCTTCAAACGGTCTGTTCAACAAAGTGATCGGTCAC

TCAGGTGCTAACATCGGTTCATTCGCTGTGCAAGTGGACCCTATCGAAAACGCTATCTTC

CTGGCTAAACAAATCAGATACGACGGTCCTGACAACATCGAAGCTCTGAACGAATTCTAC

AAAAACGTGCCTTACGAACAACTGCTGTCACTGCTG

58

>Contig8909

ATGTGCGGTGGTGTGAAATGGCTGGTGCTGTGGTCACTGTGGGCTGCTAGAGTGGTGAGA

CAACCTACTGCTGCTGTGAGAGTGGGTGGTGGTTGGCTGAGAGGTTCAGTGGCTCACTCA

GGTACTCACGCTCACTACCTGGGTATCCCTTACGCTACTCTGAAAGGTAGATTCCAAGGT

TTCCTGTGCCTGGGTATCAAAGACGCTCCTGGTAACGCTGGTATGAAAGACCAAGTGGCT

GCTCTGAAATGGATCAAAAAAAACATCAGAGCTTTCGGTGGTAACCCTGAAAACGTGACT

ATCTTCGGTGAATCAGCTGGTGGTGCTTCAATCTCATACCACGTGATCTCACCTATGTCA

AAAGGTCTGTTCCACAAAGCTATCATCCAATCAGGTTCATCACTGTCAGCTTGGGCTTTC

CAATACAAACCTGTGTACGCTGCTTCACTGCTGGCTAAAACTATGGGTTTCGTGTCAAAA

GACGCTCACGAACTGTACAACTTCCTGATCGCTCAATCAGACCACGACCTGATCGTGAAC

AGAGTGCCTAGATCAGAAAACAACACTATCCTGTCAGAAGTGCTGTTCACTCCTTGCGCT

GAACAAAACATCGAAGGTGTGGAACCTTTCCTGACTAACTCACCTTACGACTCATACATC

AAAGGTGACTTCAACCAAGTGCCTGTGCTGATGGGTGCTAACACTCAAGAAGGTCTGCTG

TTCGCTGGTATGGACAGAGAAGACATGATCGTGAACGTGAAAATCGACAAAGCTTTCCCT

AAAGACCTGGACTTCCCTACTAACGAAACTAGAATGGCTGTGGCTGCTGAAGTGCAAGAC

CTGTACATGGGTAACGACACTATCTCAGTGAAAACTATCGCTAAAATCTCAAAATTCCAC

GGTGAACCTTACATCAACTACCCTCTGCTGGAAGAAACTGAACTGCTGCTGAAAACTAAC

AAATACCCTATCTACCACTACCTGTTCGCTTACGAATCAGCTAGAAACATCCTGAAAAAA

CTGCTGTCATCAGAACTGAAAACTGTGAAAGGTGCTACTCACGCTGACGAACTGTTCTAC

CTGTTCTCACAACAACTGATCCCTTCATTCTTCCAAACTAACATGATCGAAAAAATGTCA

ACTATGTGGACTAACTTCGCTAAATACGGTTCATGCTCAATCTCAATGTGCGGTGGTGTG

AAATGGCTGGTGCTGTGGTCACTGTGGGCTGCTAGAGTGGTGAGACAACCTACTGCTGCT

GTGAGAGTGGGTGGTGGTTGGCTGAGAGGTTCAGCTCCTGGTCCTGAACCTATCTGGGAC

GGTGTGTTCGAAGCTATCGACGAAACTGTGAGATGCACTCAAGCTGTGGTGCTGGAATCA

AAATACTCAATCGGTGAAGAAAACTGCATGAAACTGAACGTGTACACTCCTCTGAACATC

CCTGACGACACTAAACTGCCTGTGATGGTGTTCATCCACGGTGGTGGTTTCTTCGAAGGT

TCATCATCAAGAATGATCTACGGTCCTGAATACCTGACTAGAAAAGGTATCATCCTGGTG

ACTATCAACTACAGACTGAACGTGCAAGGTTTCCTGTGCCTGGGTATCAAAGAAGCTCCT

GGTAACGCTGGTCTGAAAGACCAAGTGGCTGCTCTGAGATGGGTGAAAAGAAACATCAGA

GCTTTCGGTGGTGACCCTGACAACGTGACTATCTTCGGTGAATCAGCTGGTGCTTCATCA

GTGTCATACCACATGCTGTCACCTATGTCAAAAGGTCTGTTCCACAAAGCTATCGTGCAA

TCAGGTTCATCACTGGCTGGTTGGGCTTTCCAATACAAACCTGTGTACATCGCTTCACTG

CTGGCTAAAGTGATGGGTCTGGACTCAAACGACCCTAAAGAACTGTACGAATTCTTCATC

AAACAAACTGACTCAGACCTGGTGACTAAAAGAGTGCCTAGACAAGAAAACAACATCATC

ATCACTGAAATCCTGTTCGCTCCTTGCGCTGAAAAATCATTCCAAGGTCAAGAATCATTC

CTGACTGAACTGCCTTACGAACTGCTGTCAACTGGTAACTACAACAAAGTGCCTCTGATC

ATCGGTGCTAACACTGAAGAAGGTATCACTCTGATCGGTCTGGAAGAAGACAACCTGGTG

GAAAAAGTGAAATTCGAAAGAGCTCTGCCTAAAGACCTGGTGTTCCTGTCAAACGAAACT

AAACAAGCTGTGGCTAACAAACTGAAAGAATTCTACATGGGTTCAGACGAAGTGTCAAAA

GACACTAAAGTGAAACTGTCAAAACTGTACGGTGAACCTTACATCAACTACCCTGTGATG

GAAGAAACTGACCTGATGCTGAAAACTAACGACAAACCTATCTACCACTACCTGTTCGGT

TACGACGGTAGAAGAAACATCGTGAAAAAAACTCAAAACCTGCCTCTGAGAGAAATGGAC

GGTGCTTCACACGCTGACGAACTGTTCTACCTGTTCTCACAAACTCTGATCCCTGAATTC

GCTGAAAAAGTGATGATCGACAAAATGACTACTATGTGGACTAACTTCGCTAAATACGGT

AACCCTACTCCTCACACTACTGAAAACCTGCCTCTGCTGTGGCCTCAAGTGACTACTGAC

AACCAACTGGCTCTGAAAATCGACCAAGAATTCTCAACTATCCCTCTGTGGCACTCAGAC

TCACTGAAATACTGGAGAGAAATCTACTCAAAATACAGAAGAAAAAACATCTCATAC

59

>Contig8909

ATGCTGTCAAAAGGTAAACTGAACAAAGTGCCTATCATCATCGGTTCAAACACTCAAGAA

GGTATGCTGCTGGCTGGTCTGGACAAAGACTACTACATCGAAGCTATCAAATTCGAAAAC

GCTCTGCCTAAAGACCTGGTGTTCCCTAACAACGAAACTAGAAAAATGGTGGCTGACAAA

CTGAAAGAACTGTACCTGGGTGACGACGAAATCGCTCCTAAAAACATCGACAAACTGTCA

AAACTGTACGGTGAACCTTACTTCAACCACCCTGTGATGGAAGAAACTGTGCTGATCGTG

GAATCATCAGACCAACCTGTGTACCCTTACGTGTTCGGTTACGACGGTTTCAGAAACGTG

CTGAAACTGGGTCTGAGATCAGAACTGAGAAAAATCGCTGGTGCTACTCACGCTGAAGAC

ATCTTCTACCTGTTCTCACAACCTTTCCTGCCTGCTCTGTTCGAAACTGAAATGATCGAA

AGAATGACTACTATGTGGACTAACTTCGCTAAATACGGTGACCCTACTCCTGCTACTACT

AAACTGATCCCTTACAGATGGCTGCCTGCTAACGCTGAAAACCCTCAAGCTCTGCTGATC

GAAAAAGAATTCAAAACTATCCCTCTGTGGTACTCAGAATCACTGAGATTCCTGAAAGAC

GTGTACTCAAAATACAGAAGAAAACTGGAACAATAC

60

>Contig132

ATGCACCTGTTCATCCTGCTGTCAGTGCTGGCTCTGGCTCCTCTGCCTGCTAAAGCTTGG

AAAGGTACTAGAAACGCTACTGAACACGGTCCTATCTGCATCCAATACGACGTGGTGACT

AAACACATCCTGCCTGGTTCAGAAGACTGCCTGTTCCTGAACGTGTACACTCCTAAACTG

AAACCTACTAAACCTCTGCCTGTGCTGTTCTTCATCCACGGTGGTGGTTGGAAATCAGGT

TCAGGTAACGACGACGAATACGGTCCTGACTTCCTGGTGCAATACGACATCGTGCTGATC

ACTATCAACTACAGACTGGACGTGTTCGGTTTCCTGACTATGGACAACGAAGACGTGCCT

GGTAACGCTGGTATGAAAGACCAAGTGCTGGCTCTGAAATGGGTGAACAACAACATCAAA

TACTTCGGTGGTGACCCTGAAAACATCACTATCATGGGTCAATCAGCTGGTTCAGCTTCA

GTGATCTACCACATGATGTCACCTATGTCAAAAGGTCTGTACAAAAGAGCTATCGCTATG

TCAGGTGTGCCTCTGTCAGAATGGGCTCAACCTTTCGAAGCTCCTAGAAGATCATTCTCA

CTGGGTACTATCCTGGGTAACCCTACTACTGACCCTAAAGAACTGATCAAATTCCTGAGA

AACGTGCCTGCTGAAAACCTGCTGAACACTAAACCTGCTGTGATCTCACAAGAAGAACTG

TGGATCAACCCTATCAAAATGTACGCTCTGGTGCCTGTGGTGGAAAGAGACTTCGGTCAA

GAAAGATTCCTGGTGGAAACTCCTACTGAATCAATCATCAACGGTAACGTGAGAGACCAA

GAACTGATCATCGGTCTGACTTCAATGGAATTCATCTACGCTATCCCTTGGCTGGAAGAC

GACCACGTGGCTAGATTCAACCTGGCTCTGGACCTGCTGGTGCCTCTGGACCTGCTGAGA

GAAATCACTCCTAAAGAAGTGGGTCACTACAGAACTCCTCTGCCTAGAAAACCTTGGAAC

AAAACTAGATCAGCTAGAGAACACGGTCCTGTGTGCATCCAATACGACATCTTCACTGAC

AGATACCTGCCTGGTTCAGAAGACTGCCTGTTCCTGAACGTGTACACTCCTTGGATCAAC

CCTAAATCACCTCTGCCTGTGCTGTTCTTCATCCACGGTGGTGCTTGGAAATCAGGTTCA

GGTAACGACGACAACTACGGTCCTGACTTCCTGATGCACTACGACATCATCCTGGTGACT

ATCAACTACAGACTGGACGTGCTGGGTTTCCTGTCACTGGACACTAAAGACGTGCCTGGT

AACGCTGGTATGAAAGACCAAGTGCTGGCTCTGAAATGGGTGCACGACAACATCGGTAAC

TTCGGTGGTGACCCTAACCAAGTGACTATCATGGGTCAATCAGCTGGTGGTGCTTCAGTG

ATCTACCACATGATGTCAACTGTGTCAAGAGGTCTGTTCAAAAGAGCTATCTCAATGTCA

GGTGTGCCTCTGTCAGACTGGGCTTTCCCTTTCGAACAAACTACTAGAGCTTACGAACTG

GCTAAAACTATGGGTCAAAACATCGACAACTCAGAAGACCTGCTGAACTACCTGCAAAAC

GTGCCTGCTCACGACCTGCTGAACACTAAACCTGCTGTGCTGTCATCAGAAGTGTACTGG

AACAACGGTATCAAAATGTTCTCATTCGTGCCTGTGATCGAAAAAGACTTCGGTAAAGAA

CAATTCCTGGTGGAACCTATCAAAGAAGCTCTGGTGAACGGTAACATCGAAGACACTGAC

CTGCTGATCGGTTACACTGACCTGGAATACCTGATCGCTGTGCCTCTGCTGGAATCATAC

GGTCTGGCTGACTACAACATGTTCAAAGAACTGCTGGTGCCTAGAGAAATCCTGCTGGAA

ATCTCACCTAAAAGAATGCTGTCACTGGCTGAAAAAATCCACAAAGCTTACTTCGGTAAC

CAATTCATCAACAACACTACTATGCTGAACTTCCAAAACTACGGTGGTGACCACATCTTC

ATCAACTCAGTGCTGAGATTCCTGAGATACCTGCCTAAAGGTAGAGCTAAAAGATACCTG

TACAAATTCTTCACTGAATCATCAAGAAACAGATACCTGAAAAACGCTGAAAAATACAAC

ATCCACGGTTGCGCTCACACTGACGACCTGATGTACCTGTTCGACGCTAAACAAGAAGCT

ACTGCTATGAACGTGGACTCAGACGAATACCAACTGATCAACCAAACTTGCACTCTGTTC

ACTAACTTCGTGAAATACGGTAACCCTACTCCTGACGACGCTCTGGGTGTGACTTGGCCT

GAATACAACAACGACGACAGACCTTACCTGTCAATCAACAACAAACTGACTACTGGTACT

CACCTGGACTTCGACGAACCTGTGCCTAGAAAACCTTGGAAAGGTATCAGAGACGCTACT

CAACACGGTCCTGTGTGCTCACAATACGACTTCTTCGCTCAAATGTACCACCCTGGTTCA

GAAGACTGCCTGTTCCTGAACGTGTACACTCCTAACCTGGCTCCTAAAACTCTGCTGCCT

GTGATCTTCTTCATCTACGGTGGTGGTTTCAAATCAGGTTCAGGTAACGAAGACGAATAC

GGTGCTGACTTCCTGGTGCAACACAACGTGATCGTGGTGACTGTGAACTACAGAATGGAC

GCTCTGGGTTTCCTGTGCCTGAACATCAAAGAAGTGCCTGGTAACGCTGGTATGAAAGAC

CAATCACTGGCTCTGAAATGGGTGCACGACAACATCCAATACTTCGGTGGTAACCCTTAC

GAAGTGACTATCATGGGTCAATCATCAGGTGCTGCTTCAGTGATCTACCACATGATGTCA

CCTATGTCAAGAGGTCTGTTCAAAAGAGCTATCTCAATGTCAGGTGTGCCTTTCTGCGAC

TGGGTGATCCCTCTGGAACCTCAAAAAAGAGCTTTCGAACTGGGTAAAGCTCTGGGTAAA

AACACTAAAAACCCTTACGAACTGCTGGACTTCCTGCAATCAGTGCCTACTGAACAACTG

GTGAACACTGCTCCTCTGGTGATCTCACAAGAAATCATCTGGAACTTCCCTTTCAGAATG

TTCCCTTTCGTGCCTGTGGTGGAAAACGACTACGGTCAACAAAGATTCCTGGTGGAATCA

ATCAAAGACGGTCTGACTATGGGTAACATCGCTGACGTGGACCTGCTGATCGGTTACACT

TCAAACGAAAACCTGATGGCTATCCCTTTCCTGGAATCATACTACCACACTTACGACACT

TACTCAGAACTGTTCGTGCCTAGAGAAGTGGCTCTGGAAATCTCACCTAAAGAAGTGCTG

AAAGAACCTCTGGTG

61

>Contig132

ATGGTGAAACTGAGAGTGAAACAAGGTTGGCTGCAAGGTGAAGAACTGGAAACTTCAACT

GGTAACGTGAAATACTGCTCATTCAAAGGTATCCCTTTCGCTACTCCTCCTCTGGGTGAC

CTGAGATTCAAAACTCCTCTGCCTATGCAACCTTGGAAAGGTGTGAGAAACGCTACTGAA

CACGGTCACATGTGCCCTCAAAGAGACATCTTCACTGGTGAAATCATCCCTGGTTCAGAA

GACTGCCTGCACCTGAACGTGTACTCACCTAACTGCTGCACTGACTCAAAACTGACTGTG

ATGGTGTTCATCCACGGTGGTGGTTACAAATCAGGTTCAGGTAACATCCACCACTACGGT

CCTGACTACCTGGTGCAATACGGTGTGGTGGTGGTGACTATCAACTACAGAGTGGAAACT

TTCGGTTTCCTGTGCCTGGACACTGAAGACGTGCCTGGTAACGCTGGTCTGAAAGACCAA

GTGCTGGCTCTGCAATGGGTGAAAGACAACATCTCAGTGTTCGGTGGTGACCCTGAAAAA

GTGACTGTGTTCGGTGAATCAGCTGGTGGTGCTTCAGCTATCCTGCACACTCTGTCACCT

ATGTCAAAAGGTCTGATGAAAAGAGTGATCGCTATGTCAGGTGTGCCTACTTGCAACTGG

GGTGTGCCTTTCGAACCTAAAAGAAGAGCTTTCGTGCTGGGTAAACAAAGAGGTTTCAAC

ACTAACAACCCTTCACAACTGCTGAAATTCCTGCAATCAGTGCCTAAAGACGAACTGATC

GACTCAGGTCCTTGCGTGCTGACTTCAGAATTCATCATCCAAGACAACACTATCCAAATG

TACCACTTCACTCCTGTGTCAGAAAAAAACTGCGGTCAAAACCACTTCCTGACTGAAAGA

CCTCTGGACATCCTGAGATCAGGTAAAATGCACGAAGTGGACGTGATGATCGGTTACACT

TCAAAAGAAACTCTGCTGGGTGTGGACTGGTTCAAAAACAAATACCTGGCTGAATACGAC

CAATACCCTGAACTGTCAGTGCCTAGAAAAATCCTGCTGGAATGCCCTCCTAGAAAAATC

CTGCAAATCTCAGACAGAATCAAAGCTCACTACTTCGGTAACAACTCAATCACTATGAAA

CAC

62

>Contig132

ATGGTGAAAGTGCAAATCCAACACGGTTGGCTGGAAGGTGAAGAACTGGTGCCTTCAACT

GGTGAAGGTGTGTACTACTCATTCAAAGGTATCCCTTACGCTGCTCCTCCTGTGGGTAAA

CTGAGATTCAAAGCTCCTGTGCCTCCTAAACCTTGGGACGGTGTGAGAAAAGCTACTGAA

CACGGTCCTATCTGCTGCCAAAGAGACATCTTCACTCAACAAATCATCGCTGAATCAGAA

GACTGCCTGTACCTGAACGTGTACACTCCTAACCTGCAACCTTCAAAAGCTAAAGCTGTG

ATGGTGTTCATCCACGGTGGTGGTTACAAATCAGGTTCAGGTAACGTGGACCACTACGGT

CCTGACTTCCTGATGTCACACGACGTGGTGCTGGTGACTATCAACTACAGACTGGAAGCT

TTCGGTTTCCTGTGCCTGGACACTCCTGACGTGCCTGGTAACGCTGGTCTGAAAGACCAA

GTGCTGGCTCTGAAATGGGTGCAAGAAAACATCTCAAAATTCGGTGGTGACCAAAACAAC

GTGACTGTGTTCGGTGAATCAGCTGGTGGTGCTTCATCAGCTCTGCACATCCTGTCACCT

ATGTCAAGAGGTCTGTTCAAAAGAGCTATCCCTATGTCAGGTGTGCCTCTGTGCGGTTGG

TCACAACCTTTCGAACCTCAAAAAAGAGCTTTCGCTCTGGGTAAACTGCTGGGTCTGGAC

ACTGAAGACCCTCAAAAACTGCTGGACTTCCTGCAATCAGTGCCTGCTGAAAAATTCATC

GACGTGTACCCTTGCATCCTGACTTCAGAAGAAATCACTGGTAACCTGCTGAAAATGTTC

CACTTCACTCCTGTGGTGGAAAAAGACTTCGGTCAAGAAAGATTCCTGGCTGAATCACCT

GAATCAATCCTGAAAAAAGGTAAAGTGAACGACGTGGACATCCTGATCGGTTACACTTCA

TCAGAAACTCTGGTGGCTATCCCTTTCCTGGAAAACAAACTGCTGAACGACTACTCAAGA

TACTCAGACATGCTGGTGCCTACTGAAATCCTGAGAAAATGCTCACCTGGTAAAATCCTG

GAAATCTCATCAAGAATCAAATCACACTACTTCGGTAAAAAACCTATCTCAGTGGACACT

ATGAAAGAACTGCTGGCTTACCTGTCAGAATCAGTGTTCGCTTACGACATCTACAGATAC

ATCAAACTGCTGCCTAAATCAAACAACTCAAAAAGATACCTGTACAAATTCTCATGCGTG

TCAGGTAGAAACATCTACGGTCAACAAGGTCAAAAATACGGTCTGACTGGTGCTTCACAC

CTGGACGACCTGATGTACCTGTTCGACGCTAAACACGCTAACCTGAACCTGGAAAAAAAC

TCAAAAGAATACAAACTGGTGAAACTGGTGTGCACTCTGTTCACTAACTTCGCTAAACAC

GGTACTCCTACTCCTGACGCTTCACTGGGTGCTACTTGGCCTGAATACGGTGACAAAGAA

TACTTCTGCGACATCTCAGACGAACTGACTGTGGGTCAATCACTGGACAAAGAAGTGATC

GACTTCTGGAAAACTATCTACGAATTCGCTTCAACTGAA

63

>Contig3224

ATGAAAGTGTTCCTGTTCATCCTGGGTGCTCTGGCTGCTGTGCAAGCTCAAGGTAACTCA

TCAAAACTGGTGAACATCGCTCAAGGTCCTGTGAGAGGTTACAAAATGCCTGGTCTGGAC

GTGTTCGCTTTCTACTCAATCCCTTACGCTAAAGCTCCTTCAGGTAGAGACAAATACAAA

TGCGTGTACTCAAGATACAAATGCCACGAACTGCCTGTGGTGGTGGTGATCCACGGTGGT

GGTTTCCAATCAGACTTCGGTAACTACGAATCACCTTCACAACTGGTGGACCAAGGTAAA

AACATCATCGGTGTGACTCTGAACTACAGACTGGGTCCTATCGGTTTCCTGTGCCTGGGT

ACTCCTGACGTGCCTGGTAACGCTGGTATGAGAGACCAACTGGCTGCTCTGAAATGGGTG

CAACAAAACATCGCTGCTTTCGGTGGTAACCCTAACGACGTGACTATCGCTGGTTGCTCA

GCTGGTGGTGCTTCAGTGGACCTGCACATGCTGTCAAAAGCTTCAAGAGGTCTGTTCCAC

AAAATCATCGGTCAATCAGGTGGTAACATCGGTGCTTTCGCTGTGCAAGTGGACCCTACT

GCTTCAGCTAGAAAACACGCTAGAGACATCGGTTACACTGGTCCTGACACTCTGGAAGGT

GTGGAAGAATTCTACAAATCAATCCCTTACGAACAACTGCTGTCAAAAGACCTGCACGAA

AACAAAGACGTGTCAATCGTGATGGCTCCTTGCGTGGAAAGAGACATCGGTATCGAAAGA

TTCCTGGAAGAAACTCCTATCTCAATCATCAAAAAAGGTGACTACATCAGATACCCTGTG

CTGTACGGTTGGGCTGCTATGGAAGGTATCTTCAGACTGGAATACTTCGAAAACTGGAAA

AAAGAAATGAACGAAGACCTGCAAATCTCATGCCTGAGAATCTACTCACTGATCATGAAA

AACACTAACGCTAGATGCCTGACTAAAAACCCTACTCCTGACGGTAACATCCCTCCTCTG

TTCCCTAAATGGGAAGCTGCTTCAGCTAACAGAACTCCTTGCATGGACATCGGTAACCCT

ATCAGAATCTACCCTGGTCCTTTCGACCTGGAAAGACAAAAATTCTGGGACGAAATCTAC

GACCAATACAAAAAAGAACCTGTGCCTCCTTACGACTTCAACGTGACTAGAAAAATGAAA

GTGTTCCTGTTCCTGCTGGCTGCTCTGGTGGCTGTGGAAGCTCAAAGAATCCCTAAAGTG

GTGCAAATCAAACAAGGTCTGGTGAGAGGTTACTCATTCGACCACTACGACGCTTTCGCT

TTCTTCGGTATCCCTTACGCTACTGCTCCTACTGGTGCTGAAAAATTCAAACCTCCTCTG

CCTCCTCCTACTTGGAACGGTATCTTCGAAGCTGTGGACAGATACGTGTTCTGCCAACAA

CTGTCAATCTTCCCTGAAGGTCAATTCAGATTCGTGGAAGACTGCCTGGCTATCAACGTG

TTCGCTCCTAACACTAACCAAACTAACCTGCCTGTGGTGGTGGTGATCCACGGTGGTGGT

TTCCAATTCGGTTTCGGTAACACTGAAACTCCTGGTCAACTGATCACTGAAGAAAAAAAC

GTGATCGGTGTGACTTTCAACTACAGACTGGGTCCTCACGGTTTCCTGTGCCTGGGTACT

CCTGACATCCCTGGTAACGCTGGTATGAGAGACCAAATCGCTGCTCTGAAATGGGTGAAA

GAAAACATCGCTGCTTTCGGTGGTAACCCTAACGACATCACTATCGCTGGTTGCTCAGCT

GGTGGTGCTTCAGTGGACCTGCTGATGCTGTCAAAAGCTGCTGACGGTCTGTTCAACAAA

GTGATCGGTCAATCAGGTGCTAACATCGGTGCTTTCGCTGTGCAAGTGGACCCTACTGAA

AACGCTAGATGGTACGCTAAATCAATCAACTACGACGGTCCTGACGACATCGAAGCTATC

GAAGAATTCTACAAAAACATCTCATACAAACAACTGTTCTCATACGACCTGAACGCTCAA

AAAGACGTGCACGTGGTGATGTCACCTTGCATGGAAAGAGACCTGGGTATCGAAAGATTC

CTGGACGACACTCCTATCAACATCCTGAAATCAGGTAACTACACTAGATACCCTGTGCTG

TACGGTTGGGCTGGTATGGAAGGTCTGTACAGAATCCAAAACTTCGACGACTGGATCATC

CACATGAACCAAGACTTCACTCAATTCATCCCTACTGACCTGAAATTCGAATCACCTGAA

CAAAAACAACAAATCGCTGACAAAGTGAAAGCTTTCTACTTCGGTGACACTCCTGTGAAC

AACTCAGACGTGCTGAACTACATCGACTTCAACTCAGACGTGATGTTCAACGTGGCTATG

CAAAGATCAGTGACTATGCAAGTGGAAGCTGGTAACAACGCTATGTACGTGGGTGTGTAC

TCATTCACTGACAACAACACTAACATCATCCCTCACACTAACGTGAGAGGTGCTAACCAC

TGCGAACAAACTAGAGCTGTGTTCGACCTGAACGACAACACTCTGACTCCTGAATACATC

GAACTGAGAACTGCTATGAGAAAAATGTGGCTGAACTTCATCCACACTGGTGACCCTACT

CCTGCTAACAACATCCCTCCTCTGTTCCCTAAATGGGAACCTGCTGCTGCTAACAGAACT

CCTTGCCTGGACATCGGTAGAACTATCCAAGTGCTGAACACTCCTTACGACGAAGAAAGA

CAAAGATTCTGGGACGACATCTACGACAGATTCAAAAGAGAACCTGTGGCTCCTTACTCA

TTCTCATCAGCTTCATCACTGGTGTACGGTGGTGTGCTGTTCTCATCATTCCTGGCTATC

ATCACTATGATCATCTCA

64

>Contig2209

ATGACTTACGAAGGTGAAGTGAACGCTACTGACGACGGTCCTGCTTGCCCTCTGCCTGCT

CCTCCTACTTACCCTGTGGACGAAGACTGCCTGACTATCAACGTGTACACTCCTGGTCAC

AACAGATCAGAACCTCTGCCTGTGATCTTCTTCATCCACCCTGGTGGTTTCTACTCAATG

ACTGGTAGATCAGACCTGGCTGGTCCTCACTACCTGCTGGACAAAGACGTGGTGCTGGTG

ACTATCAACTACAGACTGGGTTCACTGGGTTTCCTGTCAACTGGTGACGAACTGGCTCCT

GGTAACAACGGTTTCAAAGACCAAGTGGTGGCTCTGAAATGGGTGCAAAGAAACATCAGA

ACTTTCGGTGGTAACCCTGACCTGGTGACTATCACTGGTTCATCAGCTGGTTCATTCTCA

GTGTTCTTCCACATGATCTCACCTATGTCAAAAGGTCTGTTCCACAGAGGTATCTCAATG

TCAGGTTCACCTATCGGTAACAGACCTCTGCCTAGAGACCTGTACCACCTGGCTGTGAAA

CAAGCTGAACTGGTGGGTTGCCCTGCTGACAACTCATCAGTGATCATCTCATGCCTGAAA

ACTAAAACTTTCAGAGAACTGGGTAACTCACTGTTCGGTTTCAGACAATTCGGTTGGGAC

CCTATCGGTATCTGGGACCCTGTGGTGGAATGGGACTCAGCTAGAAACGTGTCATGCACT

TCATCACCTACTAAACACACTAAATGGCCTACTGCTCACTCATCAACTCTGCACCACCAA

CCTAACCAC

65

>Contig12484

CTGGCTGTGATGGTGTGGATCCACGGTGGTGCTTTCACTATGGGTTCAGGTAACGCTTTC

CTGTACGGTCCTGACCACCTGGTGTCAGCTGGTGTGGTGCTGGTGACTCTGAACTACAGA

CTGGGTGCTCTGGGTTTCCTGTCACTGGAAAACGACGAAGTGCCTGGTAACATGGGTCTG

AAAGACCAAGTGATGGCTCTGAGATGGGTGAGAGACAACATCGAAGCTTTCGGTGGTGAC

TCAACTAGAGTGACTATCTTCGGTGAATCAGCTGGTGCTGCTTCAGTGCACCTGCACATG

CTGTCAAACGCTTCAAAAGGTCTGTTCCACAGAGCTATCGCTCAATCAGGTCTGGCTCTG

TCACCTTGGGCTCTGGCTACTTCACCTAGAGCTAGAGCTTTCGAACTGGGTAAAGAACTG

GGTATCGACACTAACTCAACTGCTGAACTGCTGGGTTACCTGAGAGCTACTCCTTCAGAA

CTGCTGGTGAAAGCTGGTGCTAGACTGGGTGGTGCTTCAGCTAGAGGTTCAGACCTGAGA

TCAACTGTGGCTCTGCCTTTCCTGCCTAGAGCTGGTGCTGTGCTGGCTTGGTCATTCCCT

CACAGAAGACCTTCACACGCTGCTACTTGGTGCAGAAGAGCTGCTGCTCACTGGGTG

66

>Contig7851

ATGGCTCTGAGATGGGTGAGAGACAACATCGAAGCTTTCGGTGGTGACTCAACTAGAGTG

ACTATCTTCGGTGAATCAGCTGGTGCTGCTTCAGTGCACCTGCACATGCTGTCAAACGCT

TCAAAAGGTCTGTTCCACAGAGCTATCGCTCAATCAGGTCTGGCTCTGTCACCTTGGGCT

CTGGCTACTTCACCTAGAGCTAGAGCTTTCGAACTGGGTAAAGAACTGGGTATCGACACT

AACTCAACTGCTGAACTGCTGGGTTACCTGAGAGCTACTCCTTCAGAACTGCTGGTGAAA

GCTGGTGCTAGACTGGGTGGTGCTTCAGCTAGAGGTTCAGACCTGAGATCAACTGTGGCT

CTGCCTTTCCTGCCTGCTCTGGAACCTTCATCACCTGGTGCTTTCCTGACTGTGGACCCT

AGAACTCTGCTGCCTGGTGCTGACGTGCCTCTGCTGACTGGTTACAACGCTCAAGAAGGT

ATCATCCTGTTCAAAAGACTGCAAAGAGACCCTAAACTGCTGTCAGACCTGGAAAGAGAA

TTCAGAAGAGTGGTGCCTCCTGAACTGCTGACTAACGACGACCTGCTGGTGAGAAACATC

ACTAACGCTATCAGAACTTTCTACTTCCAACAAAGACCTGTGGACATCAGAAACATCGAC

TCACTGATCGACCTGTTCACTGACGTGATGTTCCTGAGACCTCTGCTGGAAACTGTGAGA

CTGTCAGTGGGTAGAATCTCACCTACTTACGTGTACAGATTCGCTTTCGACGGTGCTCTG

GGTCTGTTCAAAAGAATGCTGGGTATCTCAACTAGAGCTCCTGCTACTGCTACTAGATGG

GCTACTTGCTCAACTTCAAGAGACTCAACTCCTACT

67

>Contig7851

ATGTTCCTGCTGAGAAAACTGAAATCATTCCTGCTGCTGCCTAAACAAGACCAACTGGTG

ACTTCAGCTGCTACTATGTCACACCCTATCGTGACTGTGCAACAAGGTCAACTGAAAGGT

GCTACTAAAAACCTGCTGGACGGTTCACCTTACTACTCATTCAAAGGTGTGCCTTACGCT

CAACCTCCTGTGGGTAAACTGAGATTCAAAGCTCCTCTGCCTCCTAAACCTTGGAACGGT

GTGTACGACGCTTCAGAACACGGTCCTGTGTGCCCTCAAGTGGACCTGTTCACTATGTCA

ATCCTGGAAGGTTCAGAAGACTGCCTGTTCCTGAACATCTACACTAAATCACTGAAACCT

GACTCAAAAATCCCTGTGATGGTGTACATCCACGGTGGTGCTTACATGGCTGGTTCAGGT

GACTCAGACTCACTGGGTCCTGAATTCCTGCTGCAACACGACGTGATCCTGGTGACTATC

AACTACAGACTGGAAGTGCTGGGTTTCCTGTGCCTGGACACTCCTGACGTGCCTGGTAAC

GCTGGTATGAAAGACCAAGTGGCTGCTCTGAAATGGATCAAAAACAACATCGCTAAATTC

GGTGGTGACCCTGACAACATCACTCTGTTCGGTGAATCAGCTGGTTCATCATCAGTGACT

TACCACCTGATCTCACCTATGTCAAAAGGTCTGTTCCAAAAAGCTATCGCTCAATCAGGT

ACTTGCCTGGAAGACTGGGCTATCGCTAGAGACGCTAAACAAAGAGCTTTCAGAATCGGT

AAATTCCTGGGTAAAGAAACTGACAACGTGGAAGAACTGCTGGAATTCCTGCAATCAGTG

CCTGCTGTGCAACTGGCTAACATCACTTTCAAAGTGAGAACTCCTGACGAAAAATCAAGA

GGTCTGCCTATGTACTTCGTGCCTGTGGTGGAAAAAAAATTCAACGAAGTGGACTCATTC

ATCACTGAAGAACCTGTGGACCTGCTGCTGGGTAACAAAGTGAACAAAGTGCCTCTGATG

ATCGGTTACAACTCACACGAAGGTATGCTGATGATCGCTGACAAACTGAAAAAAGCTGAC

GTGTACAACAACAACGCTTCATACCTGGTGCCTAGAGACATCATCTCAAGAGTGTCAGCT

GACAAAGCTAAACTGTTCGGTGACAAAATCATGAAATTCTACCTGGGTGACAAAGGTATG

TCAAACGACACTGAAGAAGGTATCGCTCACATGCAAACTGACATCAACTTCGCTTACAAC

ACTCACAGATTCGTGCACTTCTACTCATCAGCTCCTATCTACCTGTACAAATTCTCATGC

GACACTGAACTGAACATGGTGAAAAAACTGTCAAACCTGTCACACCTGAAAGGTGCTTGC

CACGCTGACGACCTGTTCTACATCTTCTACGCTGGTCTGTCAGAAGACGTGTACAAAAAC

GACGAAAACCTGAAAGAAATCGTGTACAAAGTGACTAAACTGTGGGCTAACTTCGCTTCA

ACTTCAAACCCTACTCCTGACAAATCACTGGGTGCTAAATGGTCACCTTTCACTAGAGTG

GGTAAAGAATTCATGGACATCGACCTGCAACTGAAACCTGGTCACAACACTGAAGGTGAA

AGAGTGGACTTCTGGAACAGAATCTACTGCGAAGCTGGTGTGCCTTGCATCACTAAATCA

ACTCTG

68

>Contig2295

ATGCCTGTGCAAGTGACTGTGAACCAAGGTACTATCGAAGGTGAAAAAAGAACTACTTTC

TGCGGTAAAACTTACTACTCATTCGAAGGTGTGCCTTACGCTAGACCTCCTATCGGTAAA

CTGAGATTCAGAGACCCTCAACCTCTGGAATCATGGACTGGTGTGAGAGACTGCACTAGA

CCTGGTAACAAATGCGCTCAAATCAACCCTTTCTCAAAAAAAGGTTTCGAAGGTTCAGAA

GACTGCCTGTACCTGAACGTGTACACTCCTTCACTGCCTGCTGTGAAACTGCAAAAACTG

CCTGTGCTGTACTTCGTGCACGGTGGTAGACTGACTTTCGGTTACGGTGACTACTACAAA

CCTGACTACATCGTGAAACACGACGTGATCCTGGTGACTCTGAACTACAGACTGAACATC

CTGGGTTTCCTGTCACTGGACACTCACCTGGTGCCTGGTAACGCTGGTCTGAAAGACTGC

GTGGCTGCTCTGAGATGGGTGAAATCAAACATCGGTGTGTTCAACGGTGACTCAAACAAC

ATCACTGTGTCAGGTGAATCAGCTGGTGCTGCTATCGTGACTGCTTTCCTGACTTCAAAA

ATGGCTGTGGGTCTGTACCAAAAACTGATCTCACAATCAGGTAACGTGCTGGCTGACGTG

TTCATGTGCAACCAAGACCACATCGCTGAAGCTGGTAGAGTGGCTGAAAAACTGGGTGCT

AAAATCGCTCAAAAAGAAGAACTGTACGGTTTCCTGCAAAACGTGGCTGTGGAAGACCTG

GTGAAAGCTTACTCATCAACTAAATCACACAGACTGCTGAACCCTGTGCTGATGGCTGTG

GTGGAAAAAAAATTCCCTGGTGTGGAAAGATTCATCCACGAACCTCCTATCGTGTCATTC

ATCAACAACAACTTCGCTAACGTGCCTACTATCGTGGGTTCAAACTCATACGAAGGTGGT

GCTTTCGTGAACAAAGGTAAAAACGGTATCAAATACCAAAAAGACTTCAGAAAATACGTG

CCTCACTACATGTACCTGGAAGAAACTGACCCTAAATGCATGAAAATCGCTAAACAAATC

AAAAAATACTACTTCCAAGACAGAGAAGTGGACGACGACACTAAAATCGAATACATCAGA

CTGCTGTCAGACGCTTTCTTCAACAGAGACACTACTAACTTCACTGAATTCATCGCTAAA

AACAACGAAAACAACTGCTACTCATACAAATTCAAATACGTGGGTTCACTGAACATCGGT

ATCATGAAAGAACTGGGTGTGCACGCTACTACTCACGGTGACTCAATCCAATACCAATTC

TACAGAAAAAACCAAGAACTGAAAACTACTGAAAGAGACAGAGTGATCATCGACTTCTTC

TCAGAAACTAAAGGTTTCGAAGGTTCAGAAGACTGCCTGTACCTGAACGTGTACACTCCT

TCACTGCCTGTGGAAAAACTGCAAAAACTGCCTGTGCTGTTCTTCGTGCACGGTGGTAGA

CTGACTTTCGGTTACGGTGACTACTACCAACCTGACTACTTCCTGAGACACGACGTGATC

CTGGTGACTATCAACTACAGACTGGGTATCCTGGGTTTCCTGTCACTGGACACTCCTGAA

TCAGACGCTTACCTGTACATCCACGACCACGTGGAAGTGGCTAGAAGAATCGCTGCTAAC

CTGGGTGAAGACGTGAAAGACAAAGCTGAACTGTACAACTTCCTGCAACAAGCTCCTGTG

GAAAACCTGGTGGCTGTGAACGCTATGACTAAACCTGAAAAATTCCTGAACCCTCTGCTG

ATGCCTGTGATCGAAAAAAGATTCCCTGGTGTGGAATCATTCATGGACGAAGCTCCTATC

AACTCATTCAGAACTAACAACTTCATGAAAGTGCCTATCATCGCTGGTTCATCATCATAC

GAAGGTGCTGCTTTCGTGTACAAAGGTAAAAACGGTATCAAATACGAAAGAGACCTGACT

ACTTACGTGCCTGCTTACATGTACCTGAACAAATCAGACCCTAAATGCCTGAAAATCGCT

AACTCAATCAAAGAATACTACTTCAAAAACAAAGAAATCAACGACGACACTAAAATGGAA

TACATCCAAATGATCTCAGACGCTTGCTTCAACAGAGACGTGACTAACTTCGCTGAAATC

TACTCAAAATCAGTGGACAAATTCTACTCATACAAATTCAAATTCGTGGGTTCACTGAAC

CTGGGTATCATGAACAACCTGGGTGTGAAAGGTTCAACTCACGGTGACTCAATGCAATAC

CAATTCTTCAGAAAAAACCAATACGAAAAATCAACTCAAAGAGACAGAGACATGATCGAC

TTCTTCTCAGAAGCTTGGACTTCATTCGCTAAAAAC

69

>Contig6196

ATGATCAACGTGATCCTGGTGATGGTGCTGTGGCTGGCTAAAACTGTGCAACCTGAAGAA

GTGGTGACTGCTCAAGGTGTGCTGAAAGGTGAAATGCACCAAGGTTACATCTCATACAAC

GGTCTGGCTCCTACTTGGCACGGTATCAGAGTGTCACAACCTACTAGATGCTCATCATCA

ACTAAACCTGAAGAATGCCTGAAACTGGACGTGCACGTGCCTACTGCTGACGCTCCTTGG

CCTGTGCTGGTGTGGATCGCTGGTGGTTCAGGTAGATACAACCCTGGTAAACTGACTCAA

CAAGGTATCGTGGTGGTGGTGATCTACCACAGATTCGGTCCTCTGGGTTTCCTGTGCCTG

GGTGAAGAAAAAATCCCTGGTAACGCTGGTCTGAAAGACGCTGTGCTGGGTCTGAGATGG

GTGAGAGACAACATCTCAGCTTTCAAAGGTAACCCTTTCAAAGTGGTGGTGGCTGGTCAA

GGTTTCGGTGCTGCTATGGTGGAAACTCTGCTGCTGACTCCTATGGCTGAAAACCTGTTC

CACGGTGCTATCATGCAATCAGGTTCAATCCTGTGCCCTTGGGCTTTCAACTTCGACGCT

AAAGAAAGAGCTCTGGTGCTGTACCAAATGCTGAACAACGCTGACATCGAAGACATGGCT

CTGGTGAACGCTTCAATCCAAGACCTGGCTACTAACTCAGACAAAATCGAATTCCCTTAC

ATCCCTTTCGGTATCTGCGTGGAAAAATCATTCAAATACGAAGAAAGACTGCTGACTGAA

TCACCTTACAGATCACTGTCAAACAAAAACAGAAGAGTGCCTCTGATCATGGGTTACAAC

TCAGACGAAGCTTACATCTTCCTGTCACTGCTGAGAAGAAACAAACTGAGAAAAAGAACT

CCTAAAAGAATCGCTAGACTGCACGCTGCTTCAGCTCTGCCTGTGTACCTGTACAGATTC

TCACACGCTTCACAACCTGGTGTGACTCCTGACCACGGTATCAAAAAAACTGGTGCTGCT

CACTCAGACGAACTGGCTTACCTGTTCCCTGACAAAGCTAAAGACATGAGAGACGACGAC

GGTGTGGTGCAACAAAACCTGAGAGCTCCTTCAAGAGGTGCTAAAGTGGGTGCTAGAGAC

AGAGTGAGACCTCCTACTCCTGGTTACTTCCACAGAGCTGGTGACGCTGCTTGCAGAGAC

AACAGACACCTGGGTAGATACCTGAGAACTGTGCTGCAATGGGAAACTTCAAACATCTAC

CTGAGAATCATGTTCGGTAACAACGGTGCTGACACTTGCAGATACAGAGCTCTGACTGAA

GGTGGTTGGGTGTGCGGTACTACTAAACACGCTCAAGACGGTTCAAGATACGTGTCATTC

AGAGGTGTGCCTTACGCTGAACAACCTCTGGGTGAACTGAGATTCAAAGTGCTGGGTTTC

CAACTGAACGCTACTGTGGAAGGTCCTATCTGCCCTCACCACGACGTGTTCTACGGTAAA

CTGATGACTCCTCAAGGTGTGTCAGAAGCTTGCATCCACGCTAACGTGCACGTGCCTCTG

AAAGCTCTGCCTAAACCTGGTAAAGAAGTGAAAAAACCTCTGCCTATCCTGGTGTACATC

CACGGTGGTGGTTGGATCTGCGGTTCAGGTGACGCTGACCTGAACGGTCCTGAATACCTG

GTGTCAAAAGGTGTGATCATCATCACTTTCAACTACTCAAAAGTGCCTGGTAACAACGGT

CTGAGAGACATGGTGACTCTGCTGAGATGGGTGCACAGAAACGCTAGATACTTCGGTGGT

GACCCTGACCAAATCACTCTGGCTGGTCAATCAGCTGGTGCTTCATCAGCTCACATCCTG

TCACTGTCACCTGCTGCTAAAGGTTGCCTGAAAGGTATCCCTCTGGCTTGGCTGATGTCA

GGTACTGGTATCCAATCATACTACACTGCTTCACCTGCTTTCTCAAAACTGGTGACTGAC

TCATTCCTGGCTACTCTGTCACTGAACTCAACTGACATGGACACTGTGCACGACCAACTG

GCTAAAATGCCTCTGGACCAAATCAACACTGCTTCAGGTGTGGTGCTGGCTAAACTGGGT

ATCACTTCATTCGTGCCTGTGGTGGAATCACCTCTGCCTAACGTGACTGCTATCCTGCCT

GAAGACCCTGAAGCTCTGATCGCTAGAGGTATCGGTAAAAACGTGTCACTGGTGATCGGT

TTCACTTCAAACGAATGCGAAGCTAACAGACCTAGACTGACTGAAATCAACCTGGTGGGT

GTGTACCAAACTAACCCTACTGCTGCTCTGTCACCTCACGTGGTGTTCACTATGCCTTCA

AACCTGACTTCAAAAATGGCTAAAAAACTGGACGACAGATACTTCTCAGGTGACGTGAAC

ATGGACAAATTCATCCAACTGTGCACTGACGAATTCTTCGCTTACCCTGCTCTGAAACTG

GCTAGAATCAGAGCTGCTTCAGGTGGTGCTCCTGTGTACCTGTACAAATTCGGTTACAAC

GCTAAATCATCAGTGTTCAAAACTGCTTTCAACCTGACTTACCAAGGTGCTGGTCACTCA

GAAGACCAAAACAGAATCCTGAAAGTGAACTCAATGTGCCCTAGAGACGAAGACCTGGTG

ATGTCAGACTGGATGTCAGACATGGTGGCTAACTTCATCAAAACTGGTTCAGACCCTCAA

ACTACTAAAGACGAAACTACTCCTGCTGGTTCAATCGACAACTCAAACACTGCTAACACT

TACACTTCAACTGTGACTACTACTAAATACACTATCGCTGCTGCTACTCAACCTTCAGCT

ACTACTGTGACTCAAACTATCGACTCAAACGACGACTTCGTGGCTCACACTGAAAACGGT

CCTGTGAGAGGTTTCCCTTGGCAAGGTTCAGCTGAAATCCTGGCTTACATCGACATCCCT

TACGGTCAATTCGAATCAGTGTTCGAAGAACCTACTGGTCCTAACTCATGGGACATGGTG

ATCGAAGCTAAAGAACACACTAAAAGATGCCCTCAACTGGACGGTGACCAATACGTGGGT

ACTGCTGACTGCCTGACTCTGTCAATCTTCACTCCTAGATCAGCTAAACACACTTCAGTG

CTGTTCTACATCCACGAATCAAACTTCATCTCAGGTTCAGGTGACCCTGCTATCTACGGT

AACGCTGGTCTGAAAGACCTGGCTCTGGCTCTGAACTGGACTAAAAACAACATCAACCTG

TTCGGTGGTAACCCTGAAAACATCGCTGTGTCAGGTGACGGTACTTCAGCTGCTCTGGCT

GAATACCTGGCTATCTCACCTAAATCAAAACACCTGGTGCAAAAAGTGATCTCAGACTCA

GGTTCAGTGCTGTCACACTGGGCTATCGACAGAAACCCTGACACTGTGGGTAAAGAATTC

ATCAAAAAAGTGGCTAACCAAACTGGTAAAGAAATCGGTGACGACATCTTCAAAGACGTG

GACATGGAAATCCTGCTGGTGGCTGCTAGAGGTCTGGTGATGAGACCTTGCGTGGAACTG

GACGGTTACCTGACTGACACTCCTTGGGACCTGGTGGACAAACAATCACTGTCAAACATC

TCATTCATCATCGGTTCAGCTAACCACGCTGGTCTGCTGGAAGCTCTGGAACACAACGAA

GCTTCAATCAACAACCTGAAAAACAACTTCGCTTCACTGCTGCCTAACGACCTGACTTTC

TCAAACACTAAAGACAAAAACTACATCGGTGACTCATTCAGAAAACAATACTTCGGTGAC

GGTGACATCTCACTGAACCACTCAAAAGAATTCTCACTGTACCACACTGACGTGTCATAC

CTGGGTCCTTCAATCAGATTCGCTAGAGCTCTGGTGAACGCTGGTGCTACTGTGTACATC

GGTGGTGAAGCTTGGAGATCATCAGGTGGTTTCTCAAGAGTGTTCGTGCACACTAGACTG

ACTCACACTGCTACTGAAACTAAAGAAAAACAAATGGTGGAACTGATCGCTGACCTGTGG

GTGTCATTCCTGAAAAACTCAGAACCTTCATCACACGGTGTGACTTGGAGACCTCTGAAC

GCTACTATCGACTCAGACGAAGAATGGCTGTCAATCGGTGAAGAAACTTCATCACAAAGA

GGTCTGCACCCTGACAGACTGAGACTGTGGACTAGAGTGTACAGAGACCACTTCGTGGAC

ACTAACTTCTCAGGTAGACTGATGACTACTAACATCCTGACTGTGCTGTACTTCGTGATG

CTGACTAAACTGGTG

70

>genemark-Contig6196

ATGGAAAGAGTGCCTGAAAGAGACGTGAGAAGACCTGTGTGCCCTCAAACTGACGTGGTG

TACGGTCAACTGATGCAAGCTAAAGGTGGTATGTCAGAAGCTTGCATCCACGCTAACGTG

CACGTGCCTATCAAAGCTCTGCCTGCTGGTGACGTGGCTGTGGACCAAACTGCTCCTGGT

CTGCCTATCATCGTGTTCATCCACGGTGGTGGTTTCGCTTTCGGTTCAGGTGACACTGAC

ATCCACGGTGCTGACTACTTCATCAACAAAGACGTGATCGTGATCACTTTCAACTACTCA

ATCCCTGGTAACAACGGTCTGAGAGACGCTATCACTCTGCTGTGCTGGGTGCACAGAAAC

GCTGCTGCTTTCGGTGGTAACCCTGACGACATCACTATCGGTGGTCAATCAGCTGGTGCT

GCTCTGGCTCACATGCTGACTCTGTCACCTGCTTCAGAAGGTCTGTTCAAAAGATCAGAA

TTCTTCTCATCATCACCTGTGTTCGCTAAAACTATCGCTGACCAATTCCTGGCTATCGTG

AACATCGACCCTACTCTGGACCCTGAAGAAATCCACCAAAGACTGGTGCTGATGCCTCTG

GAACAAATCATGGCTGCTAACAAAGCTATGATCGACGTGTTCGGTCTGACTACTTTCATG

CCTGTGGTGGAATCACCTCACCCTGGTGTGACTACTGTGCTGGACGACGACCCTGAAGCT

CTGGTGGAAAAAGGTAACGGTAAAGACATCCCTCTGATCGAAATCGACATCGCTGGTAGA

CTGCTGTCAACTCCTGTGCTGGCTGTGCCTCCTAGACTGGCTTACAAAGCTTCACTGGCT

GCTGTGCCTCTGCTGATCAAAACTGTGAACTCAAGATACTTCAACGGTACTGACGCTCTG

GACGACTTCGTGAACTACTGCTCAGACGCTTTCTACAAATACCCTGCTCTGAAACTGGCT

GAAAAAAGAACTGCTATCGGTGGTGCTCCTGTGTTCCTGTACGAATTCTCATACGGTGGT

GAACACTCAGTGATCAAAGAAGCTTTCAACCTGTCATTCAAAGGTGCTGGTCACATCGAA

GACCTGACTTACATCTTCAAAGTGAACTCATTCCAAGGTCCTACTTCAGAAAGAGACGAC

GCTATGAAAGACCTGATGACTAACCTGTTCGTGAACTTCATGCAATACGGTTCACCTCTG

GCTGTGGCTGGTAGAGTGGCTGGTGTGGGTGCTAGACTGCCTGTGCTGGTGGTGAGAGCT

CCTAGAGACCTGTCACTGCCTGGTTCAGCTAAACTGCTGCAAGCTCCTCTGCCTCACCAA

CCTGTGGAACTGGCTGAATCA

71

>Contig6196

ATGATGGACGTGTTCAAAGTGGGTAAATCATGGGGTACTTTCCTGCCTCCTTCAAAACTG

CTGAACCAAGGTTTCGTGGTGGTGACTTTCAACTACAGACTGGGTGCTCTGGGTTTCCTG

TGCCTGGGTACTCCTGACGCTCCTGGTAACGCTGGTCTGAAAGACCAAGTGGCTGCTTTC

TACTGGATCAACAGAAACATCGCTAACTTCGGTGGTAACCCTGACGACATCACTGTGTAC

GGTGTGGGTGCTGGTGCTACTTCAGTGCAACTGATCCTGCTGTCAGGTGCTGCTAACGGT

CTGTTCCAAAAAGTGATCCTGGAATCAGGTTCAGCTCTGTCACCTCTGGCTCTGGCTTAC

GACCCTCTGCAACACGCTTACGAAATCGCTATCACTCTGGGTTACACTGGTGACTCAGAC

GCTAAAGCTCTGGGTAAATTCTACAGAGAACAACCTTTCAGAAAACTGGTGCAATCAACT

TCAATGTTCCTGCCTTGCGTGGAAAACAACCTGGTGGCTACTCACTCACTGCTGGAATTC

GACCCTCTGATCATCTACCAACAAGAAATCTCACACGACGTGCCTATCGTGATCGCTTAC

AACAACATCCTGGGTGCTAAAATCATCTCAGAAATCCCTATCTCATCAATCCCTGACTCA

TTCGACAACATGCTGCCTAACAACCTGATCTTCGACAACGACCACATCAAACAAAAAGTG

GTGGAAGTGGTGAGAACTTTCTACCTGGGTCAAAACACTCTGGGTGACGACAACACTCAA

GACTACGCTGACTACCTGAACGACGTGATCCTGGAATACCCTGTGATCAAATCAGCTGTG

CTGCACGCTAACAAATCATTCTCACCTATCTACCTGATGAAATTCTCAATCAACAACGTG

AAAAACGAAAAATACTTCAGATACAGAATCATCGACTACTTCTACGAACAAGACGACCTG

ACTGCTGACATCCAAGTGACTTCAGAACAACTGATGACTCTGTGGTCAAACTTCATCAAA

ATCGGTTACATCTTCTACATCTTCCTGACTAGAACTATCCTGTACTTCGTGTTCGACTAC

TCAGACCCTACTCCTATCATGTCATCAGTGGTGCCTGTGGTGTGGCCTCCTATCAGAGCT

AACGAAGACAACGTGAGAATCAAATCAGTGACTGGTCTGATCTTCTCAAACAAACTGCAA

ATCACTAACAAAATGGCTCACGAAAGAATCATCTTCTGGGACCAAATCTACAAAGAATTC

TACAAACCTCACATCGCTGCTCACAAAAACGAAGAATTCGCT

72

>Contig3761

ATGAAATTCACTGTGGTGATCCTGCTGCTGCTGGTGATCGTGTCAGGTGAATTCGAAATC

GACGACTTCATCTCAGTGAACGTGAAAGGTTGCAAAGTGATCGGTAGAAAACTGTACACT

ATCATCGAAGACAAACCTTTCGTGGCTTTCTGGGACATCCCTTACGCTGAACCTCCTCTG

AGAGAACTGAGATTCAAAGTGTCAATCTTCGTGTACAACCCTAAATACATCGTGTACCTG

CAACCTCCTAAACCTAAATACTGGCACAACGAAACTCACGACTACTCAAACCCTAGAAAA

ACTACTTGCTGCTACAACGAAGACTGCCTGTACCTGTCAGTGTACATGCCTATCTCACAC

TCACAAACTTGGAACATCCCTGTGATCGTGTGGCTGGAAAACGGTCCTAAAGAAAACAGA

CCTGACTTCATCCTGGAAGCTAACCTGGTGGTGGTGGTGATCTCATACAGAAAAAACATC

TTCGGTTTCCTGAACACTTCAGACGAATTCGCTATGGGTAACATGGGTGCTAAAGACGTG

GTGCTGGCTCTGAAATGGATCAGAGACCACATCGCTTCATTCTACGGTGACTACAACAGA

GTGACTGTGATGGGTTTCGGTTCAGCTGCTGTGATCGTGTCATCACTGGTGCTGACTCAA

ATCGCTGAAGGTCTGTTCGCTAGAATCGTGATCATCGGTGGTTCAGCTATCTCACCTGCT

GACTACAGAAAATACCACTTCGACGTGATGGAAAAACTGTACTGGAACCTGCAAGGTCCT

TTCGAAAAACTGAACAGAACTAGACTGTACGAAATCCTGGGTAACTACTCAACTTCAGAA

ATCATGCTGGCTTCAAAAAACCTGTACGACTCAACTGAAGTGAGAGACAACCAAAGACTG

ATCAACACTTTCGGTCCTGGTATCGAAAACTCAAACAACGGTTTCATGAACAAACTGCCT

CTGGACGTGTACAAAGCTGGTCTGACTAACAACAACGTGGAAGTGATGATGGGTTACACT

TCATTCGAATCACTGTACAAACTGGAAGGTTTCGTGAAAAACAAAAAACTGCTGAAATAC

CTGAACTACAACTTCCAATACCTGCTGCCTTTCGACGGTGTGAAAGACGAATACGAATCA

AAAAGATACAGAAAAATCAAAAAAGCTATCAAAGAATTCTACTTCGTGAACGGTACTATC

GGTGAAAGATCACTGAGAAGATACGCTAAATACGTGTCAGACCAAGTGATCTACCCTCTG

CTGAGACAAGCTAGACTGCAATCATGCGTGTCATGCAAATCAGTGTACCTGTTCAGATTC

ACTTTCAAAGGTGCTTTCAACGTGGCTTGGGACTTCTCAGTGAGAAACCTGAACTGGTCA

GGTGCTACTTCAGGTGACGAAATCTGCTACCTGTTCAGATGCAAATCAGTGAACGACATG

TACAACTCAGCTAAAGCTTCAAACGAAAGACACTTCATCAAAAAAATGGCTACTCTGCTG

GCTAACTTCGCTAAATGCGGTGACCCTACTCCTGACACTACTGACGAAGTGCTGGGTAAA

CTGAAATGGGAACCTTTCACTCCTGAAAAAATCATCAGAGCTATGAACTTCGGTCTGAGA

TTCAAAATGGTGGACGTGCCTGAACAAAGAAGAGTGAAATTCTGGGACGAACTGAAATCA

GAATACTTCCCTGAAAAACTGGCTAAAGACGAACTG

**73**

>Contig3761

ATGATGGTGCACCTGCCTATCAGAACTAACTTCAGATTCGACAGAAACGACTCATCATTC

GAAGACTACAAAGAAGAAATCCTGAAATTCTACCTGAAAGGTAAACAACTGAAATACTAC

AACATCCTGGACTACGCTGTGTACGTGGCTGACATCGTGGAAAACTACTCACTGAACATC

GCTGCTAGAAAACTGTCAAGAGAACTGGAATCACCTACTTACTACTACATGTTCGACTTC

AACGGTCTGCTGAACGAAAACATGATCTACCTGTCAAAAAACACTAGAGGTGGTACTAAC

AACTGGGGTGCTTCAGTGGGTGACGAAATCTGCTACCTGCACCTGTGCTCAAGAATCAGA

GACAACTACCTGGAAATCAAATCACTGGTGTCACAACAAAACGAATTCAAACTGGTGAGA

AAAATGATCAGACTGTGGGCTAACTTCGCTAAAACTGGTAACCCTACTCCTCAAGCTGAA

GACGACGTGCTGAAAAACTTCATCTGGAAACCTATCGACAAAAAATCAGAAAACCTGCCT

TACGCTCACATCACTAAAAGAATCAAAATGCTGGAAAACCCTCTGGAAAAAAGAGAAATC

TTCTGGAACAACCTGCTGGACAAATACTCAAAACTGGCTGTGAACGGTGTGGTGAGAAAA

ATCGAAGCTCACGACGAACTG

**74**

>Contig3761

ATGTACAGAAACATCATCATCGTGCTGTTCACTTTCCTGGCTCTGAAACTGACTTGCATC

AACGCTAGAGGTCCTATCGTGGAAACTACTCACGGTAAAGTGGAAGGTAAAGTGGTGAAA

ACTCTGCTGAAAAACGTGAAATACCACGCTTACATGGGTATCCCTTACGCTGCTCCTCCT

ATCAAAGACCTGAGATTCCTGCTGCTGAAAGAACTGAACGACCTGCCTGCTTCAGACCTG

CTGTCAAAAGAACTGAGAGCTGCTCCTGACGACTACTTCAAAGAAGAACAAAGAGGTACT

ATCTCATTCGCTCCTATCGTGGAAAAAGACCCTAACGGTCTGATCACTAAATACCCTGAA

GACTCAGAAGACGGTATCAACATCCCTATCATGATCGGTTTCAACTCAAGAGAAGGTATC

GACGCTTCAATCCACTACCTGGAAGAACCTAGATTCCTGTCATTCGTGCAAAAAGACTTC

CCTTTCTTCATCCCTATCAGAGCTAAATTCAAATTCAACCCTAACGACGAAATCTGCTAC

GAAGCTATCAACGAAATCAAAGACCTGAACCCTACTCCTGACGGTGACGCTGCTTCAAAA

GAACTGAAATGGCCTACTTACAACTCAGAATCAAAAAACTACCTGCACATCACTAAAACT

ATCGAACACTTCAAAATCGCTTACAACGCTTCAAAAGAATACGGTCCTGACTTCTTCATG

AAAGACGTGATCGTGGTGACTCTGCAACACAGACTGGGTTGCCTGGGTTTCCTGTCATTC

GAAGACGACCTGCTGCCTGGTAACAACGGTCTGAGAGACGTGCTGCTGGCTCTGAAATGG

ATCCAAACTAACATCGGTTCACTGGAAGTGATCCCTACTTCAAACTCAAACGGTAACGAC

GGTGGTGCTGTGATCGTGGACCTGCTGCTGCACTCACCTAAAGCTAAAGGTCTGTTCCAC

AGAGCTATCCTGCAATCAGGTTCATCATGGTACCCTGTGTACCTGGGTGACAAACCTAGA

GAAAGAGCTATCGCTTTCTCAAAAGAACTGGACGAACACTCAAAAGCTAACTCATCAAGA

GACAAAGTGGAATGGAAACCTGCTACTAAAGAAAACAAAGAATGCCTGGTGATCTCAGAC

GACCTGTCACTGAAAACTAGACTGCACGACGAAAGAATCTCATACTGGGACACTTTCCTG

GAAACTTACGGTAAACTGGCTGTGGACGGTGTGATCAAAGACATCAAAGACGAACTG

**75**

>Contig3203

ATGAAATTCACTGTGGTGATCCTGCTGCTGCTGGTGATCGTGTCAGGTGAATTCGAAATC

GACGACTTCATCTCAGTGAACGTGAAAGGTTGCAAAGTGATCGGTAGAAAACTGTACACT

ATCATCGAAGACAAACCTTTCGTGGCTTTCTGGGACATCCCTTACGCTGAACCTCCTCTG

AGAGAACTGAGATTCAAAGTGAAAAAAAAACTGTACACTAACTTCGTGTTCAGAGACCCT

ACTCCTGACACTACTGACGAAGTGCTGGGTAAACTGAAATGGGAACCTTTCACTGCTGAA

AAAATCATCAGAGCTATGAACTTCGGTCTGAGATTCAAAATGGTGGACGTGCCTGAACAA

AGAAGAGTGAAATTCTGGGACGAACTGAAATCAGAATACTTCCCTGAAAAACTGGCTAAA

GACGAACTG

**76**

>Contig3203

ATGACTTTCTCACTGCTGCCTAAAAGAAAAAGACACGGTCAACCTTTCGGTGTGTTCGGT

GTGGAAGACTGCCTGTACCTGGACATCTTCTCACCTAAAACTGACGGTACTCTGTACCCT

GTGATCGTGTTCCTGTACAACGAAAACTTCTCAGGTTCATACAACAAAACTAAAGACTAC

TCACCTGACTTCTTCATCGAAGAAGACGTGGTGGTGGTGACTCTGTCACACAGACTGTCA

GTGATCGGTTTCCTGTCACTGGACGACGAAATCGTGCCTGGTAACTCAGGTCTGAAAGAC

ATCGTGGCTGCTCTGGAATGGACTAAAAACAACATCGTGAAAATCACTCTGATGGGTTCA

CTGGGTGGTGCTACTGCTATCGACCTGCTGATCAACTCAAACGCTAAACACCTGTTCCAC

TCAGCTATCCTGCAATCAGGTACTTCATGGGCTTCAACTTACCTGCAAGAAAACCTGAGA

GAAAGAGCTTACAAACTGGCTGAAGTGCTGGAAAGATCATCATCATCAAACGGTAAACTG

CTGAAAGAACTGAACGACCTGCCTGCTTCAGACCTGCTGTCAAAAGAACTGAGAGCTGCT

CCTGACGACTACTTCAAAGAAGAACAAAGAGGTACTATCTCATTCGCTCCTATCGTGGAA

AAAGACCCTAACGGTCTGATCACTAAATACCCTGAAGACTCAGAAGACGGTATCAACATC

CCTATCATGATCGGTTTCAACTCAAGAGAAGGTATCGACGCTTCAATCCACTACCTGGAA

GAACCTAGATTCCTGTCATTCGTGCAAAAAGACTTCCCTTTCTTCATCCCTATCAGAGCT

AAATTCAAATTCAACCCTAACGACGAAATCTGCTACGAAGCTATCAACGAAATCAAAGAC

CTGTACTACAAAAAAGGTAAAGTGACTATCAAATCAATCGCTGAACACATCACTTACCTG

GGTGACGTGCTGACTGCTTACCCTGTGGACTACACTGCTAGAGTGTACTCACAAAGATCA

TCAAAATCACTGTACTACTACTACTTCGACTACTTCTCAGACCTGAACGAAAACAAAAAC

AACCTGATGAAAATCTCAAAAGTGGCTGAAGGTACTTGGGACGGTGACGCTGCTTCAAAA

GAACTGAAATGGCCTACTTACAACTCAGAATCAAAAAACTACCTGCACATCACTAAAATC

ATCGAAGTGAAATCAGACCTGTACAAAGAAAGATTCGAATTCTGGGACAACTTCCTGAAA

AAATGGAAACAAAAAGCTGCTCCTAAACCTCACTCAGGTTGGACTGACGTGCTGCAAGCT

AAAAAAGAAAAAAAACCTTGCGCTCACTTCAACCTGCCTATGAAACACATCGAAAACTAC

GGTTTCGCTGGTATCGAAGACTGCCTGCACCTGTCAGTGCACACTCCTACTCTGCCTTCA

AAAGACCACAACCCTTCACTGCCTATCATCGTGTTCCTGTACAACCAACACTTCAAAATC

GCTTACAACGCTTCAAAAGAATACGGTCCTGACTTCTTCATGAAAGAAGACGTGATCGTG

GTGACTCTGCAACACAGACTGGGTTGCCTGGGTTTCCTGTCATTCGAAGACGACCTGCTG

CCTGGTAACAACGGTCTGAGAGACGTGCTGCTGGCTCTGAAATGGATCCAAACTAACATC

GGTTCATTCGGTGGTGACCCTAACAGAGTGACTCTGATGGGTAACGACGGTGGTGCTGTG

ATCGTGGACCTGCTGCTGCACTCACCTAAAGCTAAAGGTCTGTTCCACAGAGCTATCCTG

CAATCAGGTACTTCATGGTACCCTGTGTACCTGGGTGACAAACCTAGAGAAAGAGCTATC

GCTTTCTCAAAAGAACTGGACGAACACTCAGTGTCATCATCATACCTGCTGAAAAGACTG

GGTCCTCTGGACGCTAACGTGCTGACTGAAGCTGAAGACAGATCAATCCACGCTGACGAA

GCTAGAGCTATCCAAAAAGGTATCCTGGCTTTCGGTCCTATCGTGGAACACAAACACGAC

GACGCTATCATCACTGAATACCCTGAACACAAACCTACTATCGACATCGCTGTGCCTATC

ATGATCGGTTACAACTCAAGAGAAGCTACTACTGGTGACGAACTGTGCTACCTGTTCGTG

TGCAAATCACTGAAAAAAACTTACAAAAAAGGTATGGAAGAAGAAGACTCAGAAGAAATG

AAAGTGCTGAGAAACATGCCTCCTAAACCTTCAGAAGGTTGGGCTGACCTGTACAAAGCT

TACGACGACAAACCTACTTGCGTGCAATTCTCATTCAAAAGAAGAAACGGTGAAAAAATC

GGTATCTCAGGTTCAGAAGACTGCCTGCACCTGTCAGTGTTCACTCCTAACCTGGAAGGT

TCAAGAGCTGTGATCGTGTTCGACTACCACGACTCATACCAAACTGGTTTCAACGGTACT

AAAACTTACTCACCTGAATTCCTGCCTGAAGAAGACGTGGTGATCGTGTCAATCTCACAC

AGACTGGGTCTGCTGGGTTACCTGACTACTGAAGACGAAGTGATCCCTGCTAACAACGGT

CTGAGAGACTACATCCTGGCTCTGGAATGGATCAAAAACAACATCAAACACTTCGGTGGT

GACCCTGACAGAGTGACTCTGATGGGTTGCAAAGGTGGTGGTGCTCTGGCTAACGTGCTG

CTGTACTCAAAAGCTGCTAAAGGTCTGTTCAACGCTGTGACTATCCAAGGTGGTACTGCT

CACGAATCACTGTACTTCCCTAAAAAACCTAGAGAATTCGCTTACAAACTGGGTAACATC

CTGGGTATCGGTGCTAACGAATCATCAAACCTGCTGAAAGAACTGCAAAACGTGGACTTC

ACTGCTCTGCTGACTAAATCAGGTGAAGTGGTGAACCCTGAAGAATTCCTGGACTACCAA

ATCTCAGTGTACCACTCACACCAATCA

**77**

>Contig9545

ATGCCTATGGTGTTCCACCTGATGCTGTACTCAGAAAACATCAAAATCATCAACGCTCCT

TCAATCGGTGACGTGGACGAATTCTCATTCTCAATCTCAAAAAACCTGTACATCTTCTTC

CAAAGAACTTCAACTTCACTGTGGGGTGAATGGATGGGTGTGATGCACGGTGACGAAATG

GAATACGTGTTCGGTCACCCTCTGAACATGTCACTGCAATACCACACTAGAGAAAGAGAC

CTGGCTGCTCACATCATGCAATCATTCACTAGATTCGCTCTGACTGGTAAACCTCACAAA

CCTGACGAAAAATGGCCTCTGTACTCAAGAGCTTCACCTCACTACTACACTTACACTGCT

GACGGTCCTTCAGGTCCTGCTGGTCCTAGAGGTCCTAGAGCTTCAGCTTGCGCTTTCTGG

AACGACTTCCTGAACAAACTGAACGAACTGGAACACGTGCCTTGCGACAGAGCTGTGACT

GGTCCTTACTCATCAGTGGCTGGTACTACTCTGCCTGTGCTGCTGCTGACTGCTCTGGCT

ACTACTATCGCTCTG

**78**

>Contig9545

ATGGGTAGAGAAGTGCACATCTTCACTGGTATCCCTTTCGCTAAACCTCCTCTGGGTCCT

CTGAGATTCAGAAAACCTGTGCCTATCGACCCTTGGCACGGTGTGCTGGAAGCTACTTCA

ATGCCTAACTCATGCTACCAAGAATCAGAAGAAGCTCCTGGTAACATGGGTCTGTGGGAC

CAACAACTGGCTATCAGATGGATCAAAGACAACGCTAGAGCTTTCGGTGGTGACCCTGAA

CTGATCACTCTGTTCGGTGAATCAGCTGGTGGTGGTGTGTCACTGCTGCTGGCTGACCCT

TCACTGGTGATGGACTGCATGAGAGGTGTGGACGCTAAAACTATCTCAGTGCAACAATGG

AACTCATACACTGGTATCCTGGGTTTCCCTTCAGCTCCTACTGTGGACGGTGTGTTCCTG

CCTAAAGACCCTGACACTATGATGAAAGAAGGTAACTTCCACAACGCTGAAGTGCTGCTG

GGTTCAAACCAAGACGAAGGTACTTACTTCCTGCTGTACGACTTCCTGGACTACTTCGAA

AAAGACGGTCCTTCATTCCTGCAAAGAGAAAAATTCCTGGAAATCGTGGACACTATCTTC

AAAGACTTCTCAAAAATCAAAAAAGAAGCTATCGTGTTCCAATACACTGACTGGGAAGAA

ATCACTGACGGTTACCTGAACCAAAAAATGATCGCTGACGTGGTGGGTGACTACTTCTTC

GTGTGCCCTACTAACTACTTCGCTGAAATCCTGGCTGACTCAGGTGTGGACGTGTACTAC

TACTACTTCACTCACGTGTCAACT

**79**

>Contig6927

ATGACTACTAGAAGAGGTAGACCTCTGCAAGCTTACAGAGGTATCAGATACGCTGAAAAC

CCTACTGGTGAACTGAGATTCCAACCTCCTAAACCTATCACTGCTTACTCAGCTGAAGTG

GACGCTAGAGCTGAAGGTCCTGCTTGCCCTCAACCTGTGGTGGACCCTGACTTCGTGGTG

GACGAAGACTGCCTGAGACTGAACGTGTACACTCCTTCAAACAACAGATCAAAACCTCTG

CCTGTGGTGGTGTACATCCACTCAGGTGGTTTCTACTCAATCTCAGGTAGATCAGACGTG

GCTGGTCCTTCATACCTGCTGGACAGAGACCTGGTGCTGGTGACTATCAACTACAGACTG

GCTTCACTGGGTTTCATGTCAACTGGTGACAAATACGCTCCTGGTAACAACGGTTTCAAA

GACCAAGTGGCTGCTCTGAAATGGGTGAGAAGAAACATCAGATCATTCGGTGGTGACCCT

AACCTGGTGACTATCGCTGGTTGCTCAGCTGGTGCTTTCTCAGTGATGCTGCACATGCTG

TCACCTATGTCAAAAGGTCTGTTCCACAGAGCTTACTCAATCTCAGGTTCACCTATCTCA

CAAGTGCCTGAAAGACACCACCAAAAATACCTGGCTGAAAGACAAGCTAGACTGCTGGGT

TGCCCTACTACTTCATCAAAAGAAATCGTGGACTGCCTGAAAACTAAACCTTGGAGAGAA

ATCGGTAACACTCTGGACGACTTCTTCGACTTCGGTTACGACCCTGTGCTGGTGTGGACT

CCTGTGATCGAACAAGACTTCGGTCAAGAAAGATTCCTGTCATCATCACCTCTGAGAGCT

GTGCAAGAAGAAACTTGCACTCAATCACCTACTTCATCAGCTAGACTGCTGGGTTCATCA

TCAGGTAGACCTTCAACTTCATCAACTACTAGAAGA

**80**

>Contig6608

ATGAACGTGTGGCTGTGCTTCGTGGTGGCTCTGGCTGCTGTGCAAGCTCAAGACGACTCA

AAAATCGTGAACATCAAACAAGGTCCTGTGAAAGGTTACAGAACTCCTGGTTTCGACGTG

TTCGCTTACTACGACATCCCTTACGCTACTGCTCCTTCAGGTGTGGACAAATTCAAACTG

GTGCACCACAACAAATCAATCATCGGTGTGACTTTCAACTACAGACTGGGTCCTGTGGGT

TTCCTGTGCCTGGGTACTCCTGACATCCCTGGTAACGCTGGTATGAGAGACCAACTGGCT

GCTCTGAAATGGGTGAAAGACAACATCGCTGCTTTCGGTGGTAACCCTGACGACATCACT

TGCTGGCTGCAATGCTGGTGGTGCATCGGTAGATCAACTTACGTGATCGAATCAAAATTC

CTGGAAGACTCACCTATCAACATCATCAAAAAAGGTAACTACGTGAGATACCCTGTGCTG

TACGGTTGGGCTGGTATGGAAGGTATCTACAGACTGGACCACTTCGAAGACTGGTCAGAA

CACATGAACGAAAACTTCGCTCAATTCCTGCCTTCAGACCTGAAATTCGAATCAGAAGAA

CACAAACAAGAAGTGGCTAAAAAACTGAAAATCGCTATGCAAAGAGCTATCACTATGCAA

ACTGAAGCTGGTAACAACGCTATGTACGTGTCAGTGTTCACTTACACTGACAACTCAACT

AGAATCATCCCTTACACTGACCTGAGAGGTGCTACTCACTGCACTCAATTCAAAGTGGTG

TTCGACCTGGACGAATCAAAATACTCACAAGAATACATCGACCTGAGAGCTACTATGAGA

GAAATCTGGCTGAACTTCATCCACACTTCAGCTGTGTTCGAATACCTGAGAATGAAAATC

CTGTTCTGCATCTTCCTGACTCTGTCAGCTGCTCAAGCTCAAGGTGACTCAAAAATCGTG

AACATCGGTCAAGGTCCTGTGAAAGGTTACAGAGTGTCAGAACTGGACGTGTTCGCTTAC

TACTCAATCCCTTACGCTACTGCTCCTAAAGGTGTGGACAAATTCAAAGCTCCTCTGCCT

CCTCCTGTGTGGACTGAACCTTTCGAAGCTGTGACTCAAGAAGTGCTGTTCATCATCCAC

GGTGGTGGTTTCCAAATCCTGTTCGGTAACTTCGAAACTCCTGCTAACCTGGTGAACCAA

CAAAAAAACATCGTGGCTGTGACTTTCAACTACAGACTGGGTCCTATCGGTTTCCTGTGC

CTGGGTACTCCTGACATCCCTGGTAACGCTGGTATGAGAGACCAACTGGCTGCTCTGAAA

TGGGTGAGACAAAACATCGCTGCTTTCGGTGGTAACCCTGACGACATCACTATCGCTGGT

TGCTCAGCTGGTGGTGCTTCAGTGGACCTGCACATGCTGTCAAAAGCTTCAAACGGTCTG

TTCAACAAAGTGATCGGTCACTCAGGTGCTAACATCGGTTCATTCGCTGTGCAAGTGGAC

CCTATCGAAAACGCTATCTTCCTGGCTAAACAAATCAGATACGACGGTCCTGACAACATC

GAAGCTCTGAACGAATTCTACAAAAACGTGCCTTACGAACAACTGCTGTCACACTCACTG

AACGAAAACATCGACGTGCACGTGAAAATGTCACCTTGCATCGAAAGAGACCTGGGTTTC

GAAAGATTCCTGGAAGACACTCCTATCAACATCCTGAAAAAAGGTGACTACGTGAGATAC

CCTGTGTTCTACGGTTGGGCTGACATGGAAGGTCTGTACAGAATCAGATCATTCGAATAC

TGGAAAGACCTGATGAACGAAGACTTCTCACTGTTCCTGCCTACTGACCTGAAATTCGAA

TCAAACGAACACAAACAAGAAGTGGCTAAAAAAGTGAAAAAATTCTACTTCGGTGACGAC

CCTGTGAACGGTGACACTATCCTGGGTTTCATCGACTACAACTCAGACGTGATGTTCGTG

GCTGCTATGCAAAGATCAATCACTATGCAAGTGGAAGCTGGTAACAACGCTATCGTGCCT

AAATGGGAACCTGCTTCAGCTAACAGAATGCCTTGCATGGACATCGGTAAAACTATCAGA

GTGCTGCCTGGTGGTTACGAAGAAACTAGACAAAGATTCTGGGACGAACTGTACGACCTG

TACAAATACGAACCTATCGCTCCTTACGACTTC

**81**

>Contig8445

ATGGAATTCATCAAACCTTACTTCGCTGACAACTCATACGCTGAACTGTCAAAATGCAAC

GTGAGAGCTAGAACTGAATACGGTTGGGTGTGCGGTCTGCAAAGATGGTCAGAAGGTGGT

CAAATCTACGCTTCATTCAGAGGTGTGCCTTACGCTAAACAACCTGTGGGTGAACTGAGA

TTCAAAGAACTGCAACCTCCTGAACCTTGGACTCACGACTACGACGCTAGAGAAGAAGGT

CCTGTGTGCCCTCAACACGACGAAATCTACAACGACCTGACTCAACCTAAATACGGTATG

AACGAAGCTTACTCAAAAAAAATCACTGAATCAAAAAACAAACCTCTGGAACCTGAAAAC

TCAGAACAAGCTGCTAAAAGACAAGTGGAAAGACAAAACAGATCACTGAACGACGCTAGA

CCTGAAAACCACGAAAAATCAGAAGAAGGTGAATTCTCATCAAACAAAAAACACAGATCA

GTGAAACTGCTGCCTGTGCTGGTGTACATCCACGGTGGTGGTTTCCTGGGTGGTTCAGGT

GACGCTGACCTGCACGGTCCTGAATACCTGGTGTCAAAAGACGTGATCGTGATCACTTTC

AACTACAGAATCAACGTGTTCGGTTTCCTGTCACTGAACACTCCTTCAATCCCTGGTAAC

AACGGTCTGAGAGACCAAGTGACTCTGCTGAGATGGGTGCAAAGAAACGTGGAATACTTC

GGTGGTGACAGAAACGACGTGACTCTGATGGGTCAATCAGCTGGTGCTTCATCAGCTCAC

CTGCTGTCACTGTCAGACACTACTAAAGGTCTGTTCAAAAGAGTGATCCTGATGTCAGGT

GGTACTGCTTACGAAGCTTTCTACACTACTTCACCTCTGTTCACTGCTGCTGTGAACGCT

GCTTTCTTCGCTAACCTGGGTATCATGTCAGTGGACCCTGAAGAAATCCACAGAGAACTG

ACTAACATGCCTCTGAGAAGAATCATCGCTGCTTACTACCTGCTGCAAGCTCAAACTGGT

CTGATCACTTTCCCTCCTGTGGTGGAAACTAGATACCCTGGTATCACTACTGTGCTGGAC

AGAGACCCTGAAATCATGCTGGCTGAAGGTAAAGGTAACGAATACCCTCTGATCGTGGGT

TTCGCTTCAGCTGAAGTGGAAACTTTCAGAAGAATGCTGGACTACAAAAACTTCGTGTCA

AGAATCGAAGCTAACCCTAGACTGATCCTGCCTGTGGACATCACTTTCTCAACTCCTCCT

AACGTGTCACTGGCTCTGGGTGAAAAATCAGCTCACAGATGCATGGAACACAAATCACTG

CTGGACGCTGTGGTGATGTGCTACTCAGAAGGTCTGTTCCAATACCAACCTTTCAGACTG

GTGAGAACTAGAGCTAACATGAAAGCTGCTCCTTCATTCCTGTACCAATTCTCATACGAA

TCAGAAACTAGAGTGTTCCAAAAAGCTCTGAGACTGCAAGACTACAGAGGTGCTGCTCAC

ATCGAAGACCTGACTTACGTGTTCAGATCAAACTCAGTGCTGCCTCCTAACTCATCATTC

CCTCCTAAAGACAAAGACGACATGATGAAAGACTGGATGACTTCATTCGTGGTGAACTTC

ATGTACTGCTCAGACCCTACTTGCTCAAAAAACGGTTCAAAATCAGGTTGGCCTCAAGTG

ACTAAAGACCACCTGGTGTACGAAGACATCGAATCACCTTACGTGTACAAATTCAAAAAC

CCTTCAGACAACCTGCAAGACATGATCGAATTCTACGACAAAATCTACGAAGTGAGATAC

AAA

**82**

>Contig9544

ATGCTGAAAACTGTGGACAACATGGAAGAAAACGCTGACTCAAAACTGGACGGTGGTCCT

GTGAAAGCTGCTCTGTCAGGTGACATCACTGAACAAGGTAGAGAAGTGAAACCTAAATTC

ATCCCTATCGGTGCTATCAAAATGCCTGGTTTCTTCATGAAAAACTCAGACAGAGACAAA

TCAAAAGACGAAGACCAAATCGAAAAAGACGCTGACAACGAAAAAACTGACGAAGAACCT

AAAATCAAACACAACAGACTGCAATTCCTGCACACTTGCCCTTTCACTCAATTCCTGCAC

CACCCTAAAAAAGGTTCATCAGAAACTAGAAAAGGTGTGTTCAACATCAACTACCCTAAA

ATCTTCCAAAAAAGATCAGACACTAACCCTGAAGCTACTCTGGCTTCAATGGAAACTCTG

GAAGACAAACTGGACACTCCTAACGACGGTATGGAAAACGTGAAACTGGACATGGAAACT

GAAGAAGGTAAAGTGGCTACTAAACTGCCTCTGAAAGAAAGAATCAGACAAAAAAAATTC

ATCATCGACGACATCGTGGTGTGCGGTCTGGTGCTGCTGGTGCTGCTGATCGTGATCATC

GGTATCGTGGTGGGTGCTAGAGCTGGTCCTCCTGCTGAAAGACCTCTGAGACTGGGTAGA

TACATCACTACTATCACTACTTGCGGTCCTGTGGAAGGTACTCTGGACGAAGGTGTGTAC

AACTTCTACAACGTGCCTTACGCTATGCCTCCTGTGGCTGAAAAAAGATTCACTTACGCT

CAACCTCTGAACAACATCTCAATGTGCTGGAACGGTACTTACAAAGCTCACACTCCTGGT

CCTCTGTGCATCCAATTCCTGGAAAACGGTACTATCGTGGGTGAAGAAGACTGCCTGACT

CTGGACGTGGTGACTCCTCACGTGAGATACGACACTCCTCTGCCTGTGGTGGTGCTGATC

GGTGCTAACTCACTGGCTGGTGGTATCTCACCTGCTCAACCTTCAGCTCTGTACGCTAGA

ACTAAAGAAGTGGTGTTCGTGAGACCTAACTTCAGACTGGGTCCTTTCGGTTTCCTGGCT

CTGGACATCCTGTCAAACTCAAAATACCCTCAAACTTCAGGTAACTACGCTCTGTCAGAC

CTGCTGGCTGCTCTGCAATGGGTGAAATACAACATCGAACACTTCGGTGGTGACCCTGCT

TCAGTGACTCTGCTGGGTCACAGAGCTGGTGCTACTCTGACTGCTGCTCTGACTACTGTG

CCTAAAGTGCAAAAACTGTACTCAAGAGTGTGGCTGTCATCAGCTTCAGTGATCTTCCCT

GGTGAATCACTGGAACAATCACAAAAAAACAACGAACCTTTCAAACAAAGATCAAAATGC

GAAGACATCGAATGCCTGCAAAGAATCTCAACTACTGAAATCCTGTCAGCTACTCCTGAC

ATCTGGCTGGGTTCATCAATCGGTACTCTGCCTCTGACTGACGAAAACCAACACTCATGG

CTGGTGCTGGACGGTTACTACCTGAGACTGCACGCTTACGAAACTTGGAACGCTCAAAAA

GAAGCTAAAAACTCAGGTAAAGAAAAAATCTTCAAACCTATGGTGTTCGGTACTACTCAA

CACTCAGGTCACTCAGAACTGCTGCTGAAAAAACACCTGAACTGGACTTCAGACATCGTG

GAAGAACTGGTGAACAAATCAGTGATCGGTGAAAAAAACCTGACTGCTGCTGTGTTCAAC

CACTTCAACAAATCATACGAAGGTCTGGTGGAACTGATCTCATCAGTGAGAACTCTGTGC

CCTCTGGTGTCACTGGCTAGACTGAGACTGGCTGCTCCTATGTACGTGGTGATGGGTTCA

GGTGCTGGTGCTGGTGTGTCAGGTTCAGGTATCGCTGGTATCAACGCTGACGTGGAAGCT

ATCCTGGGTACTTTCGACTCAGAAGTGCCTGAACAAAGAAGATTCATGGCTGCTATGCAA

CAACTGTTCTACTACTTCGTGTGGCACGGTCACCTGCCTGGTCCTGAATCAGGTCTGATC

GCTGTGGAACAAGACCTGCTGCCTCTGCACGGTCTGCCTGCTTGCGACCTGCTGATCCAA

GAAGACCTGGTGCCTAGATACGCTCACATCGAC

**83**

>Contig2126

ATGTGGTCACTGTCAACTATCATCCTGCTGTGCGTGATCGGTTCAGTGCTGGGTAACGAC

GTGGCTTTCCTGCCTATGGTGTCAACTGCTCAAGGTGCTGTGGTGGGTTCAGTGGCTTCA

GACGGTGAATACTTCGAATTCTACGGTATCCCTTACGCTGACTCAACTGCTGGTACTCAC

AGATTCAAAGCTCCTCTGCCTCCTCCTTCATACACTGACGCTTTCATCGCTGACAGAAAA

GACATCAAATGCGTGAGACCTCTGGGTGTGGGTTTCGAAGGTACTGAAGACTGCCTGGTG

GCTAACATCTTCACTCCTTCACTGGGTGAAGCTTCATCACTGCCTGTGATGGTGTGGGTG

AAAGGTAGAGAATTCGACAGAGTGTACGAAAACGAACTGTCATTCAAACACTTCGTGGAA

AAAGACGTGATCGTGGTGTCACTGAACTTCAGAGAATCAATCTTCGGTTTCCTGTGCCTG

GGTACTGAAATCGCTCCTGGTAACGCTGGTCTGAAAGACATCATCGCTGGTCTGACTTGG

ATCAAAGAAAACATCTCAAGATTCGGTGGTAACCCTAACAACGTGACTCTGTTCGGTCAC

GGTTCAGGTGCTGCTGCTGTGGACCTGATCACTCTGTCACCTATGTCAAAAGACCTGGTG

CACGGTGCTATCACTCAATCAGGTAACGCTCTGGCTCCTTGGCTG

**84**

>Contig11588

ATGGTGCAAGTGACTGTGAACGAAGGTATCCTGGAAGGTGAACTGGTGAACTCAAAATAC

GGTGAACCTTTCTACTCATTCAAAGGTATCCCTTACGCTGAACCTCCTGTGGGTGACCTG

AGATTCATGGCTCCTAGACCTCCTAAAGCTTGGGAAGGTGTGAGATCAGCTAAAGAATTC

GGTTCAATCTGCTACCAAGCTGGTAGACCTGGTGAACCTAACATCGGTTCAGAAGACTGC

CTGTACCTGAACGTGTACACTCCTGACCTGCAACCTGACCAACCTCTGCCTGTGATGGTG

TGGATCCACGGTGGTGCTTTCAAATGCGGTTCAGGTAACGACGACTCATACGGTCCTGAA

TTCCTGGTGAGACACAACGTGATCCTGGTGACTTTCAACTACAGACTGGAAGTGCTGGGT

TTCCTGTGCCTGGGTACTAAAGACATCCCTGGTAACGCTGGTATGAAAGACCAAGTGGCT

GCTCTGAGATGGATCAACAAAAACATCTCACACTTCGGTGGTGACCAAAACAACGTGACT

ATCTTCGGTTGCTCAGCTGGTTCAATGTCAGTGACTTACCACCTGGTGTCACCTATGACT

AAAGGTCTGTTCAAAAGAGCTATCGCTCAATCAGGTACTTCAACTTGCTGCATCGGTATC

ATGACTCACCCTAGAGAAAGAGCTCTGGCTCTGGCTAAAAAACTGGGTCTGAACTCAGAA

GACGACAAAGAAGTGTACGAATTCTTCAAATCACTGCCTCTGGACAGACTGATCAGAGCT

CAAACTCCTGTGAGAACTGCTGAAACTGTGAGATCACAAGAAGAAATCATCCTGCTGATC

ACTTCAGAAGAAATCTTCGACGACACTGAAACTTTCTTCCACGGTGACCCTTGCACTGTG

CTGAGAAACGGTATCCACGAAGGTGTGGAAGTGATCACTGGTTACACTTCAGACGAAGGT

GTGCTGGCTATCGGTGCTGCTCCTAACATCGACGAAATCTTCAGACTGATCAACACTTTC

AAAGACTACGTGGTGCCTAAACCTATCGCTGAAAACTGCACTTCAAAAATCCAACTGGAA

GCTGGTAGAAAAATCAACAAATTCTACTTCGACAACAAAAACTCATACAACAACAACCTG

AACAACTCAGCTAAAATGCAAGTGAAAATCACTGACGGTATCCTGGAAGGTGAAACTGTG

AACAACGAAATCGGTGGTACTTTCTACTCATTCAAAGGTATCCCTTACGCTGCTCCTCCT

CTGGGTAACCTGAGATTCAAAGCTCCTCAACCTCCTCTGCCTTGGGAAGGTGTGAGATCA

GCTAAACAACACGGTGACTTCTCATACCAATTCAACTTCATGGTGAGAGCTATCGAATCA

GGTTCAGAAGACTGCCTGTACCTGAACGTGTACACTCCTAACACTAAACCTTCACAACTG

ATGCCTGTGATGATCTGGATCCACGGTGGTGCTTTCTGCTGCGGTTCAGGTAACGACGAC

GTGTACGGTCCTGAATACCTGATCAGAAACGACGACAAAGAACTGTACGAATTCTTCAAA

AACCAACCTAAAGAAAACCTGGTGGAAGCTCACCTGCCTATCACTATCAAAGAAAACGAC

TGCGACAAATACGAAACTAAAAACTCAGTGGTGTCAGAAAAACTGTTCCCTAACGTGGAA

CCTTACTTCACTGGTAACATCTTCGACGCTCTGAAAACTGACATCCACGAAGGTGTGGAA

CTGATGATCGGTTACAACGAAGACGAAGGTGTGATCAACCTGGGTGTGTCATTCGACATC

AAAAAAACTATCTTCCAAGCTAACACTTTCGTGGAATACTTCGTGCCTAGATTCCTGGTG

TACTACTGCTCAACTGACAACCAACTGGACATCGGTAAAGCTATGAAAGAATTCTACCTG

AAAAACGAAATCCTGTCAGAAAACAACCTGGAACCTCTGGCTAAATACTTCGCTGCTGAC

ATGTACAAATTCGGTCTGTACACTCTGGCTAAATACTTCTCAGTGAAAAACAAAGTGTAC

TTCTACAAATTCACTTGCAAAACTGAAAGAAACTTCTTCTCAACTATCCTGGGTGTGTCA

AAATACTTCGGTAACAGACCTCTGGTGGGTCACATCGACGAAGTGCCTTACCTGTTCCCT

GTGAAATCAATCTCACAAAGAGTGTCATCAGAAACTCAAAAATCAATCAACACTGTGTCA

AAACTGTGGACTAACTTCGCTAAAAAAGGTAACCCTACTCCTGACGCTACTCTGGGTGTG

AACTGGAGACAATTCAGAGCTGACATGCCTTCATACCTGGACATCGGTAACACTCTGGTG

AGAGGTGTGGGTCCTGACAAAGAAGAACTGAACTTCTGGGAAAAAATCTTCGACAAATAC

CTGCCTCACATGGTGTACAGAAAAGAAACT

**85**

>Contig11588

ATGCAAGTGAAAACTACTGAAGGTCTGCTGGAAGGTGAAATCGTGAACAACGACATCCTG

GGTACTTACTACTCATTCAAAGGTATCCCTTACGCTGCTCCTCCTCTGGGTGACCTGAGA

TTCAAAGCTCCTCAACCTCCTAAACCTTGGGAAGGTGTGAGATCAGCTAAAGAACACGGT

TCATCATCATACCAATTCAACTTCCTGACTAAAACTAGAGAAATCGGTTCAGAAGACTGC

CTGTACCTGAACGTGTACACTCCTAACACTAAACCTTCAGAACCTCTGCCTGTGATGATC

TGGATCCACGGTGGTGCTTTCTGCTGCGGTTCAGGTAACGACGACATCTACGGTCCTGAA

TTCCTGATCAAACACGACGTGGTGCTGGTGACTATCAACTACAGACTGGAAATCCTGGGT

TTCCTGTGCCTGGAAACTGAAGACGTGCCTGGTAACGCTGGTATCAAAGACCAAGTGGCT

GCTATGAGATGGGTGAACAAAAACATCGCTAACTTCGGTGGTGACCCTAACAACGTGACT

ATCTTCGGTCAATCAGCTGGTGCTGCTTGCGTGACTTACCACTGCATCTCACCTACTACT

AAAGGTCTGTTCAAAAGAGCTATCGCTCAATCAGGTTCAATCATCAACTGGTGGACTCAA

GGTTTCAGACCTAGAGACAGAGCTGAAGCTCTGGCTAGAAAACTGGGTTGCAACTCAAAA

GACGACAAAGAACTGTACGAATTCTTCAAATCACAACCTGTGGAAAACCTGGCTGAAATC

CAAGTGCCTCTGACTTACAAAGAATACCACTGCGACAAATACGAAACTCAATTCTCACTG

GTGGTGGAAAAAGAATTCCCTAACGTGGAATCATACTTCACTGGTAACATCATGGAAGCT

CTGAAAACTGGTATCCACGAAGGTGTGGAACTGATCATCGGTTACAACGGTGACGAAGGT

ATCATCAACATCATGGACGTGGCTAAATACCTGGGTAACAGACAAGTGACTGGTCACATC

GACGAAATCCCTTACCTGTTCCCTGTGAAATCAATCCACCAAAAAGTGGGTTCAGAAACT

CAAAAAAACCCTACTCCTGACGCTACTCTGGGTGTGAACTGGAAACAATTCAGAGCTGAC

ATGCCTTCATTCCTGGAAATCGGTAACACTCTGGTGAGAGGTGTGGCTCCTGACAAAGAA

GAACTGACTTTCTGGGAAGGTATCTTCGAAAAATACCTGCCTTCAATGGTGTTCCACAAA

GAAACTTCA

**86**

>Contig2256

ATGGAAAAAATCACTGAAGAAACTTTCTACTTCAAAGTGGAAGGTTACCCTACTATGGTG

TACTTCCACGGTGGTGACTTCGTGAGAGGTTCACCTCAAAACATCAACCCTTTCCAACTG

GTGCTGAAACAAAAAGTGATCTTCGTGTCAATCGCTTACAGACTGAACATCTTCGGTTTC

TTCTCAACTCTGGACCACGAAGCTACTGGTAACTTCGGTCTGCTGGACCAAGTGGCTGCT

CTGACTTGGGTGCAACACAACATCGAATCATTCGGTGGTGACCCTAACAACGTGTGCATC

TTCGGTCACGACGCTGGTGCTGTGTCAGTGGGTCTGCACCTGATCTCATCATACTCAGCT

GGTCTGTACCAAAAAGCTATCGCTATGTCAGGTAACGTGCTGTCACCTGAAACTGTGAAC

ACTCCTAGAAAAGAAGCTGTGACTGTGGACAAAGTGGCTAACGCTTTCTCATGCTTCAGA

AGACCTACTTTCCAACTGCTGGACTGCCTGAGAAGAGTGCCTGCTAACGCTCTGCTGGAC

ATCGGTGAACCTGTGGCTGAATGGAAACCTATCGTGGACTCAGGTTTCTCAAACACTTCA

TCACCTTTCCTGCCTGACATCCCTTCAAAAATGTTCAAAGACAAAATCTTCTCAACTGTG

CCTGTGCTGACTGGTTACACTAACATGGAAGACGCTCTGCTGCTGCACAACGAAGGTGAC

GAATCAGGTATCTCACAAAAAGAATACGACCTGATGAGAGAAGAAGTGGTGCTGTCAGAC

ATCACTATCGACAACTCATCATGCTTCACTAACCAACACCACATCCAAGACGCTGTGGCT

TTCTTCTACAAACCTATCCCTCCTACTACTAACGAAACTATCCTGAGAAAACTGTTCCTG

GACTTCTACACTGACAAAGTGCACGGTGCTACTACTTACCAACTGGCTAAACACATCTCA

GAACAAGCTCCTGTGTACCTGTACAGATTCGACCTGAAACCTTTCTCAGACATCGCTAAC

GAAGGTATCCCTGAATGGATCGGTGTGCCTCACAACTTCGACCTGATCTTCACTTTCGGT

CTGCCTCACCTGGCTCTGCCTGAAGACCTGTCAAAATGGGACTACAGAGACAAATCAATC

TCAGAAATCATCATGAAAATGTGGTCAAACTTCGCTTGGTACTCAAACCCTACTAACTCA

GGTGTGATCATCGACTGGGAACCTTTCGAAATCGAAAAACCTGGTTACCTGATCATCGAC

AGACAAAACTTCACTATGTCAACTCCTGACACTATCAACTACAAAGCTTTCGAATTCTGG

ACTGACTTCTACCCTAAAGTGGTGGAAATCGGTACTAAATGCTGCAAAGAAATCGCTGAC

GACTCAGGTGCTGACTCAGTGATCGCTAAATCAGTGATCAACTCACTGCTGGGTCTGAAC

CTGGTGCTGATCCTGCTGATG

**87**

>Contig3107

ATGTGGTCACTGTCAACTATCATCCTGCTGTGCGTGATCGGTTCAGTGCTGGGTAACGAC

GTGGCTTTCCTGCCTATGGTGTCAACTGCTCAAGGTGCTGTGGTGGGTTCAGTGGCTTCA

GACGGTGAATACTTCGAATTCTACGGTATCCCTTACGCTGACTCAACTGCTGGTACTCAC

AGATTCAAAGTGACTATCAGAGAAGCTTCATCACTGCCTGTGATGGTGTGGGTGAAAGGT

AGAGAATTCGACAGAGTGTACGAAAACGAACTGTCATTCAAACACTTCGTGGAAAAAGAC

GTGATCGTGGTGTCACTGAACTTCAGAGAATCAATCTTCGGTTTCCTGTGCCTGGGTACT

GAAATCGCTCCTGGTAACGCTGGTCTGAAAGACATCATCGCTGGTCTGACTTGGATCAAA

GAAAACATCTCAGGTCTGGGTGACCTGGTGCACGGTGCTATCACTCAATCAGGTAACGCT

CTGGCTCCTTGGGCTGTGTCAAGAGACAACCTGGCTTCAGCTGTGAAAGTGGCTGAAGCT

CTGGGTCACACTGTGAACACTATCGAAGAACTGTCAGAAGTGTTCACTAGAGTGTCAGTG

CCTGCTCTGATGGCTATCATCAACGAATTCGACCTGTCAGACAACTCACTGGTGTTCGCT

CCTTGCGTGGAAAGAGAAGAACTGGCTGAACCTGCTTTCCTGGCTAAAACTCCTTTCGAA

ATCATCTCACAAGGTAACTTCCTGGACATCCCTTTCATCACTGGTTTCGTGGACTACGAA

GGTACTATCAGAGCTGAAGAAGTGATCGAATCACAATGGCTGGAAAGAATGGACACTAAA

TTCACTGACTTCCTGCAAGCTGACCTGGAATTCCAAACTGACGACGTGATGAACACTGTG

GCTGCTAACGTGAGATCATTCTACTTCGCTAACAACTCAATCCAAGACAACCTGAAATCA

TTCATCCACTACCAAGGTGACACTATGATCCTGGTGTCAGCTCTGAGAGAAGTGAACCTG

AGATCAAAATCATCATCATCACCTCTGTACCTGTACCAATTCTCATACAAAGGTACTCTG

GGTCAACCTTTCGTGGGTCCTATCGAAGTGGACTCAGCTGCTCACTCAGAAGAACTGGCT

TACCTGTTCTACGAAATCCCTATCATCCTGGAAAACGACGACATCGCTGACAAAGACCTG

ACTGTGGCTGACATCCTGATCGAAAGATGGACTAACTTCGCTAAATACGGT

88

>Contig10960

ATGAAACTGCACTGGGCTTACTGCGGTAGAGTGTTCTACTCATCAGAACCTGCTAAAACT

ATGGAAGCTTTCCCTAACCTGCTGTCAAAATACCAACTGCTGTCAGGTGTGACTGAATTC

GAATCATACCACGACTTCGGTGTGCTGTCATCAGCTAAAGAATGCCTGAAACCTAAAAGA

AGAATCTACCACAAATGCAAAGTGATCTTCGAAGGTGCTGAAGACGAAGCTCTGAAAGAA

ATCCTGAAACAATACGCTCCTTCACTGATCGACCCTCAAAGATGGTCAGTGGAAACTAAC

AGAGACGTGGTGCTGAACCTGTTCTCAGACGCTAGAACTATCGCTCCTACTATCTCATTC

GCTAACTACCAATCAAGATCAAACAGACAATCATACTTCTACGTGTTCGGTCACAACTCA

ATCTCAACTGACTACGCTCAACTGAACAAATCAGTGCACGGTCAAGAAATCCCTTACGTG

CTGGGTGTGCCTCTGGGTGGTGCTAACACTCACTTCACTAACGTGTACACTCAATCAGAA

AAACTGCTGTCAGAAGTGGTGATGAGACTGTGGACTAACTTCGTGAAAATCGGTACTCCT

AACACTCAATCAGTGAACAAATACTTCACTGCTGACAAAGAATCATGGAACCAATACAAC

GTGGAATGGCCTGAATACGACGTGGACCACCAATCATACCTGAAACTGGCTATCCCTCCT

CAAATCTCATCACTGTTCAGAGTGAACTACACTAAATTCTGGACTGAAACTCTGCCTAAA

AAAATGAGAAGATACGTGGTGGACCCTATGTTCGACTACACTCCTTCAACTGCTAGACCT

AAACCTACTCACAGATCAACTAACTCAAACAGAAAAATGTTCGCTCCTATCGAAATCGAC

TCACCTCACAACAACGCTGCTGCTCCTCCTAGATTCTCAGCTCCTGCTCCTCCTCACTAC

TCAGCTCCTTACGGTACTGTGCACTCATACTCAGCTCCTGTGTACAAACCTGACTCAGAC

GCTATCTACAGACAAATCCAATCAATCATGACTCCTAAAAGATCACCTCCTCCTTCATTC

ATCGAAAAAACTCCTGCTAGATCAACTACTGAAAAACCTAAACCTATCCCTGTGAAAATC

TCATCAGCTACTATGACTCTGGTGATCTCACTGGTGGTGCTGTTCCTGGTGGTGAACATC

GGTATCTGCTCAATCCTGTACTTCAAAAGAAGAAAATCAAGAGCTCTGCACAACAACAAC

ATCGAAGTGTCACACCACTCAAGAGCTGACGTGGGTGAAGTGGACGTGATCGGTCAAAAA

TCATCAAAAGACGACAAATCAACTCTGCAAACTCTGAAAAACTCATGCTCAGTGATCAAA

TCACTGAGATTCTCAAAACTGAAAACTAACAACAAAAAAGGTAAAAAAAAACCTGAACCT

TGCAAAACTCCTAAATCAGACGACTCAGGTGGTTTCAGAGAAAGATTCCAACTGAGAAGA

CACCTGTCAACTTCAACTCTGGACGCTAACACTAAAGTGAGAGACTGGATCGCTAACGAA

GTGATGCACAGATGCTCACCTGGTATCCTGAGAAACCTGACTAGACCTAAATAC

89

>Contig10162

GTGGAAAGACCTTGGTTCTCAATCTCAGGTATCCAAGAACACTCAAGACAACAAAGACTG

GAAGGTAGATCAGACAGAGCTCCTGTGGCTGCTGAAGAAAGACAAGTGTTCAGAAGAAGA

GACCCTGACCAAGTGACTCTGGGTGGTCAATCAGCTGGTGGTGTGATGGCTCACATCCTG

TCAATCTCACCTGCTTCAAAAGGTCTGATCAACAGAGTGATCGCTCTGTCAGGTAACGCT

ATCTCAAACTTCTACTCATACTCAAGACTGTACTCAAAACTGCTGGCTAAAATCTTCCTG

ACTGTGATGGGTATCAACGTGTTCGACGACCCTGAAGACATCCACGAACAACTGATCGCT

GCTCCTATCGAAAACCTGATCGCTGCTAACAAATTCCTGCTGGACCAACTGGGTCTGCTG

TCATTCGCTCCTGTGATCGAAACTCCTCAACTGCAATTCACTACTGTGCTGCCTAGAGAC

CCTGAATACCTGCTGAACGAAGGTTCAGGTAACAAATACCCTCTGCTGCTGGGTTTCACT

TCAAACGAATGCCAAATCTTCGTGCCTAGACTGAAAAAAGTGAACATCGAAACTAAACTG

CAACTGCTGCCTCTGCTGATCGTGCCTACTAACCTGCTGTTCTCATCAGACCCTCTGACT

GTGCCTACTCTGGTGCTGAAAGTGTACGAAGAATACTTCAACTCAACTTCAATCACTCTG

GAAAAATTCCTGCCTGACTGCACTGACTCATACTACAAATACCCTGCTCTGAGACTGATC

GAAAAAAGACTGGCTTCAAACGCTGAAGCTACTTTCCTGTACGAATTCGGTTACAGAGGT

GAAAACTCACCTGTGAAACAAGCTATCATGGTGGACTTCCCTGGTGCTGGTCACCTGGAA

GACGTGACTTACTTCTTCAGAGTGAACTCAATCCTGGGTCCTATGCTGGAAAACGAACTG

ATCCAACACAACGCTGACTCAGCTATGAAAAACTGGATGACTACTGTGATCGCTAACTTC

ATCACTAACGGT

90

>Contig10162

GAACTGCAACCTTACGGTCAATTCCCTGGTGTGTTCAACGCTTCAGAAGTGGGTCCTATC

TGCCCTCAAATCGACATCATCTACGGTCCTATCCAACAACCTCTGTCAATGGGTGAAGAC

TGCATCAGAGTGAACGTGAACGTGCCTCTGGAAGCTCTGCCTGACCTGGGTTCACACACT

AAACCTCTGCTGCCTATCCTGATGTGGATCCACGGTGGTTCATTCTCATACGGTTCAGTG

TCAGGTGACGAAGAAAGAGACTCATGCTACGTG

91

>Contig1270

ATGGTGTACATCCACGGTGGTTACTTCCAAATCGGTTTCGCTGGTAGATACCAATACGGT

CCTAAATACCTGGTGAAACACGACGTGATCCTGGTGACTCTGAACTACAGACTGGGTCCT

TACGGTTTCATGTGCCTGGACATCCCTGAAGTGCCTGGTAACCAAGGTCTGAAAGACCAA

ATCCTGGCTCTGAAATGGATCAAAGCTAACATCGACGCTTTCGGTGGTGACTCAGACAAA

ATCACTATCTTCGGTGTGTCAGCTGGTGCTCACTCAATCCACTTCCACCTGACTTACGGT

GACGAAACTCTGTTCAAATCAGCTATCCTGCAATCAGGTTCAATGTTCTCATCAACTGTG

ATCTCAGACCCTGTGAAAAACGCTCCTCAAAGAATCTCAACTTACCTGGGTTACTCAACT

GAATCAGCTACTGAAGCTATCTCACTGCTGGCTGTGACTGACCCTAAAATCATCGTGAAA

TCAGTGATCGACATGAACCTGGAATTCAAACCTTGCGTGGAAAAATACTTCGACAACGGT

CAACCTTTCATCGCTACTACTTGGATCAACAACATCAAACCTAAAGTGGCTTCAAAACCT

ATCATGATCGGTTTCTCAGAACTGGAACTGTTCGGTTCATTCACTGACCCTGACATCAAC

GTGTACAAATCAATCTTCAGATCAAACCTGAAACTGACTTTCAACTTCAACGACGAATAC

CTGAACCAAATGGAAGAACTGGTGAGAGAATTCTACCTGGACGACGAAATCAAATACAAA

GACACTAGAAAAGCTCTGATCAACTTCCACTCAGACTTCACTTACATCTACCCTACTCAC

AAATCAAACGTGCTGGCTGAACCTATCGTGAAAACTAAACTGGGTCTGATCAGAGGTATC

AACGCTGTGGACGGTGACTACTCAATGTACATGGGTATCCCTTTCGCTAGAGTGAACTCA

TCAAACCCTTTCGGTGCTGCTCTGCCTCAAGAACCTTCAATGGACATCAGATGCGTGGAC

GACTCAGCTATCTGCCCTCAAATCGAAGAATTCAACAACACTATCGTGGGTTCACTGGAC

TGCCTGCAAATCAACATCTACGTGCCTAACAACGCTTCACCTTCAAACCCTGTGCCTGTG

ATGGCTATGATCTTCGGTGGTGCTTTCACTCTGGGTTTCCCTGGTAGATTCCTGTACGGT

CCTAAATACCTGGTGAGACACGACGTGATCCTGGTGACTTTCAACTACAGACTGGGTCCT

TACGGTTTCATGTGCCTGCAAACTCCTGAAGTGCCTGGTAACACTGGTCTGAAAGACCAA

CTGCTGGCTCTGAGATGGATCAAAGAAAACATCGGTGCTTTCGGTGGTGACTCAGGTAAC

GTGACTATCCTGGGTCACTCAGCTGGTGGTTTCTCAGTGGACTACCACCTGTACTCACCT

CTGGAAAAACTGTACAACAAAGTGATCCTGCAATCAGGTACTGCTCTGTCATCATCACCT

TCAACTCCTATCAGAAACGCTCCTGTGCTGCTGGCTAACCACCTGGGTTTCGACACTGAC

GACATCAACGAAGCTATCTCACTGCTGGCTCAAATCGAACCTAACCTGATCATCGCTGCT

ACTAAAGAACTGGGTCTGAAATTCCTGCCTTGCATCGAAAAAGAATTCGAAGGTGTGGAA

TCATTCTTCACTACTGACTGGATGAGAATGGACATCCCTAAAGTGAAAAACATGCCTGTG

CTGCTGGGTCACACTGACGACGAAGCTGGTTCAGCTATCTTCACTATGTCAAAAGAAACT

CTGACTTCAAAACTGATCGACCAACTGGTGAGATCAAAATTCAACGCTTCAGACCCTGAC

TTCAAAGGTATGGACACTCTGGTGAGACACTTCTACGTGGGTGACAACGAAGTGAACTCA

GAAAACCTGTCAACTGTGGGTATCGACTTCGGTAACGCTCTGTCAGACATCTACTTCATC

TACTCAGTGTACAGATCAATCGGTAAATACCTGGACAACAACGCTGGTGACATCTACTAC

TACCTGTACTCATACGTGGGTGGTAGAAACTTCATCAGAAACAAACTGAACATCACTTCA

GGTGGTGCTGTGCACGCTGACGAACTGGGTTACCTGTACGACATCTCATACTACAAAGAC

ACTCCTACTGTGGAAGACCAACTGATGATCGACAGAATCACTGCTCTGTGGACTAACTTC

GCTAAATCAGGTAACCCTACTCCTGAAAGATCAGACCTGCTGCCTAAAGCTATCCCTCAA

AAACCTTTCAACGACATCTTCGAAGCTCTGGACGACACTGCTATCTGCCCTCAAATCGAA

GAATTCAACAAAACTTTCGCTGGTACTCTGGACTGCCTGCACCTGAACGTGTACGTGCCT

ACTAAAGCTAACATGAACAACCCTAAAGCTGTGCTGGTGTGGATCTACGGTGGTAGATTC

GAAATCGGTTTCTCAGGTAGATACCTGTACGGTCCTAAATTCTTCGTGAGACACGACATC

ATCCTGGTGACTCTGAACTACAGACTGGGTGCTTACGGTTTCATGTGCCTGGACACTCCT

GAAATCCCTGGTAACCAAGGTCTGAAAGACCAACTGCTGGGTCTGAGATGGATCAAAGAC

AACATCGCTGGTTTCGGTGGTGACCCTAACAAAGTGACTGTGGTGGGTGAATCAGCTGGT

GCTATCTCAGCTGACTTCCACCTGCTGTCACCTCACGAAAAACTGTTCGACAAAATGATC

ATCCAAATCGAACCTAAATCAGTGATCGTGGCTACTAAAGAACTGGGTCTGTCATTCAGA

GTGTGCGTGGAAAAAAAATTCGACGGTGTGGAAAACATCATCGTGGAAGACTGGCTGAGA

GCTGAAATGCCTAAAGCTAAAAGAATGCCTATCCTGATCGGTTTCAACGAAGACGAAGCT

ATCCTGAACCCTACTCCTACTAAATCAGACCTGCTGCCTGTGGAATGGCAACCTATCAAA

AAAGACTCAGCTTTCTACTACATGGACCTGGGTTCAGAACTGAAAGTGAAATCAAGACCT

TTCCACGGTAGAATGTCATTCTGGGACCTGGTGTACAGAGTGAACGACAACCTGCTGAGA

CTGTACGGTGAAGGTAAC

**92**

>Contig40

ATGGAATCAACTAGAGACCTGCCTACTATGAGAACTGTGCTGTTCGTGGCTATCGCTCTG

GCTTGCGCTCACGCTCACGCTCACGGTAAAAGAGCTGACTCACCTCCTCAAGGTGACCCT

GTGACTGTGACTCCTTCAGGTCCTATCAGAGGTTCATGGATGACTACTAGAAGAGGTAGA

CCTCTGCAAGCTTACAGAGGTATCAGATACGCTGAAAACCCTACTGGTGAACTGAGATTC

CAACCTCCTAAACCTATCACTGCTTACTCAGCTGAAGTGGACGCTAGAGCTGAAGGTCCT

GCTTGCCCTCAACCTGTGGTGGACCCTGACTTCGTGGTGGACGAAGACTGCCTGAGACTG

AACGTGTACACTCCTTCAAACAACAGATCAAAACCTCTGCCTGTGGTGGTGTACATCCAC

TCAGGTGGTTTCTACTCAATCTCAGGTAGATCAGACGTGGCTGGTCCTTCATACCTGCTG

GACAGAGACCTGGTGCTGGTGACTATCAACTACAGACTGGCTTCACTGGGTTTCATGTCA

ACTGGTGACAAATACGCTCCTGGTAACAACGGTTTCAAAGACCAAGTGGCTGCTCTGAAA

TGGGTGAGAAGAAACATCAGATCATTCGGTGGTGACCCTAACCTGGTGACTATCGCTGGT

TGCTCAGCTGGTGCTTTCTCAGTGATGCTGCACATGCTGTCACCTATGTCAAAAGGTCTG

TTCCACAGAGCTTACTCAATCTCAGGTTCACCTATCTCACAAGTGCCTGAAAGACACCAC

CAAAAATACCTGGCTGAAAGACAAGCTAGACTGCTGGGTTGCCCTACTACTGCTCCTAGA

AAATCACTGACTGCT

**93**

>Contig40

ATGACTTCAAGAAGAGGTAGACAATTCCAAGCTTTCAGAGGTATCAAATACGCTCAACCT

CCTGTGGGTGAACTGAGATTCAAACCTCCTCAAGCTATCATGACTTACGAAGGTGAAGTG

AACGCTACTGACGACGGTCCTGCTTGCCCTCTGCCTGCTCCTCCTACTTACCCTGTGGAC

GAAGACTGCCTGACTATCAACGTGTACACTCCTGGTCACAACAGATCAGAACCTCTGCCT

GTGATCTTCTTCATCCACCCTGGTGGTTTCTACTCAATGACTGGTAGATCAGACCTGGCT

GGTCCTCACTACCTGCTGGACAAAGACGTGGTGCTGGTGACTATCAACTACAGACTGGGT

TCACTGGGTTTCCTGTCAACTGGTGACGAACTGGCTCCTGGTAACAACGGTTTCAAAGAC

CAAGTGGTGGCTCTGAAATGGGTGCAAAGAAACATCAGAGCTTTCGGTGGTAACCCTGAC

CTGGTGACTATCACTGGTTCATCAGCTGGTTCATTCTCAGTGGGTTCACCTATCGGTAAC

AGACCTCTGCCTAGAGACCTGTACCACCTGGCTGTGAAACAAGCTGAACTGGTGGGTTGC

CCTGCTGACAACTCATCAGTGATCATCTCATGCCTGAAAACTAAAACTTTCAGAGAACTG

GGTAACTCACTGTTCGGTTTCAGATGGGACTTCGGTCAAGAAAGATTCCTGCACATCCAA

CCTGACGAAGCTATCAGAAACGGTCAACTGCTGACTCCTGTGCCTTACATCATCTCACAA

ACTACTGACGAATTCTTCTGGGGTGGTTACAAATGGGAAAGAATCGCTGCTATCTCATTC

CAACTGCCTCAAGAAAACCTGACTGTGGTGGCTAGAAGACTGAAAAAAGAATACCTGGAC

GACAAACCTCTGGTGAACGACGAAGCTTCAGCTAAAGCTCTGGGTAAAATCTACGGTGAC

TCAATCATCGGTTTCAACACTCACAGAGACCCTAACTCACCTACTTCAAAAACTTGGCCT

GTGCAATGGCCTCCTATGGTGCCTCAAGACAGAAAATACCTGAGAGTGGGTGACGACCTG

ACTATCAGATCAAACATGTTCGAAGACAGATTCCAACTGTGGGAAGAACTGTACCCTATG

AAATACTCAAGATCAGTG

**94**

>Contig1812

ATGTCAGGTCTGGTGGCTGTGTCAACTAAACAACCTAACTTCGACAAACCTTTCGACGCT

TTCGACGCTACTGTGAAATGCCCTCAAGCTGCTCTGGCTGCTGAAGGTACTCTGCAATGC

CTGAGACTGAACATCTACGTGCCTCACCACCAAAACGGTACTAAACTGCCTGTGTTCGTG

TGGTTCCACGGTGGTGGTTTCTTCTTCGGTTCAGCTGGTGACTACGACGGTCAACACCTG

ATCAAACAAGACATCATCGTGGTGACTGCTAACTACAGACTGGGTCCTTACGGTTTCCTG

TGCCTGAACGACCCTGAAGTGCCTGGTAACCAAGGTCTGAAAGACCAAGTGGACGCTCTG

AAATGGGTGAAAGACAACATCGACGCTTTCGGTGGTGACGCTTCAAAAGTGACTATCGCT

GGTGAATCATACGGTGGTGGTTCAGTGGAACTGCACCTGTACTCAGAATTCGACAAATAC

TTCGACAAAGCTATCATCCAATCAGGTGCTGCTGGTGTGGAAGCTATGTTCGTGAAACCT

GACCACAAAGCTGCTATCAAACTGGCTAAACTGTTCAACTACACTGCTACTGACAACTCA

CAAGCTCTGGAATTCCTGTCAGAAGTGGACCCTATCGAACTGATGAAAACTTTCACTACT

TCAGGTATCATCCTGAGAGCTTGCAAAGAAAAAAAATTCAGAAACGTGCAAAACTTCCTG

ACTAACGACCCTTTCCACCTGTACAAACCTAAAAAAATCAAAGACACTCCTGTGATCATC

GGTTACAACTCAAAAGAAGCTTTCGTGGACAAACCTAGATACTGCCTGGACAAACTGAAA

TCATACTTCAACCTGAAATCAAAAGAACTGAGAAGACTGGCTAACATCACTCAAAAATTC

TACCTGGGTTCAAAACCTGTGTCAAAAGAAAACCAACTGAACCTGGTGGACTTCATGTCA

GACTTCATGCTGAACAACGCTGCTGAAAAAGCTGTGACTAGACTGGTGGACCAAGACGCT

AAAGTGTACAAATACCTGTTCTCATACACTGGTAACTCACCTTACAAAAACATGACTGGT

GTGGGTGCTTACCACACTGAAGAACTGCAATACCTGTTCCAAATGAAAGCTGTGCTGACT

GAACCTGAACACTTCCTGATGAGAGACAGAATGACTACTATGTGGGCTAACTTCGCTAAA

TTCGGT
