# Supplementary material for: Genome Mining and Expression Analysis of Carboxylesterase and Glutathione S-Transferase Genes Involved in Insecticide Resistance in Eggplant Shoot and Fruit Borer, Leucinodes orbonalis (Lepidoptera: Crambidae)
Source: Front Physiol. 2020 Nov 19;11:594845. doi: 10.3389/fphys.2020.594845 (PMC7713791; doi:10.3389/fphys.2020.594845)
Supplement: Supplementary Table 2 — Sequence information GST and CE from L. orbonalis. [file Table_2.DOC]

**RESITANCE GENES OF GLUTATHIONE S-TRANSFERASE**

**Total number of GST genes in Leucinodes orbonalis is 33**

**Total number of insecticide response genes predicted by BLASTX is 26.**

**The below given sequences are matching with the insecticide resistance in NCBI-BLASTX hit**

**1 >evm-Contig7206_pilon-processed-gene-0.56-mRNA-1**

ATGGCTAAATCATCATTCAGAAACAACCCTGTGCTGTACTCATACTGGAGATCATCATGC

TCATGGAGAGTGAGAATCGCTCTGAACCTGAAAGAAATCCCTTACGACATCAAAGCTGTG

TCACTGATCAAAGGTGGTGGTGAACAACACTGCAACGAATACAGAGAAGTGAACCCTATG

GAACAAGTGCCTTCACTGTGCATCGACGGTCACACTCTGGTGGAATCACTGTCAATCATG

CACTACCTGGAAGAAACTAGACCTCAAAGACCTCTGAT GCCTCAAGACTGCATGAAAAGA

GCTAAAGTGAGAGAAATCTGCGAAGTGATCTCATCAGGTATCCAACCTCTGCAAAACCTG

ATCGTGCTGATCTACGTGGGTGAAGAAAAAAAAAAAGAATGGGCTCAACACTGGATCAAC

AGAGGTTTCAGAGCTGTGGAAAAACTGCTGTCAGCTTCAGCTGGTAAATACTGCGTGGGT

GACGAAATCACTCTGGCTGAC TGCTGCCTGGTGCCTCAAGTGTTCAACGCTAGAAGATTC

CACGTGGACCTGAGACCTTTCCCTATCATCCTGAGAATCGACAGAGAACTGGAAAACCAC

CCTGCTTTCAGAGCTGCTCACCCTTCAGCTCAACCTGACTGCCCTCCTGAAGTGGCTAAA

**2 >evm-Contig2323_pilon-processed-gene-0.22-mRNA-1**

ATGGTGCTGACTCTGTACAAAAAAGACACTTCACCTCCTTGCAGATCAGTGTTCATGACT

ATCCACGCTCTGCACATCACTGACGTGAACTACATCGACGTGTACCTGCCTGCTGGTGAA

CACCTGACTGAAGAATTCATCAAACTGAACCCTCAACACACTGTGCCT CTGCTGAAAGAC

GGTGACTTC CTGATCTGGGACTCACACGCTATCTGCGGTTACCTGGTGTCAAAATACGGT

GAAAACGACCACCTGTACCCTAGAGACCCTAAAAAAAGAGCTATCGTGGACCAAAGACTG

CACTTCGACTCAGGTGTGCTGTTCAACGCTCTGAAAACTACTGTGGTGCCTCTGCTGTAC

GCTGGTGAAAAACTG

**3 >maker-Contig8855_pilon-augustus-gene-0.81-mRNA-1**

CTGTACAAAATCAACCTGGGTGCTGCTTCAAGAACTGCTATGATGGTGCTGGACATCTTC

AACGTGCCTGTGGAACTGATCGAAGTGTCACTGCTGAAAAAAGAAAACCTGACTCCTGAA

TTCCTGCAAGTGAACCCTTGCCACACTGTGCCTGTGCTGGTGGACGAAGACATCACTATC

AGAAACTCACACGCTATCTGCATCTACATCACTGAAGTGTACGGTCAAAACTCAAACCTG

TACCCTAAAGACCTGATCCAAAGAACTAACGTGAACCAACTGCTGTTCTACAACGAATCA

GTGCTGATCTCAAGATTCGGTAAACTGTCAGTGTCAGTGTTCAGAGGTGCTAACGAAATC

TCAGAAACTCAAGTGAACGAAATCAAAGAATCATACGACATCCTGAACACTCTGCTGACT

AAAACTCAATTCCTGGCTTGCGACTTCATCACTATCGCTGACATCGCTGCTTTCTCAAAC

GTGTCAGGTCTGGTGGAAATCGTGCCTCTGGACCTGTCAAGAAACGCTAAACTGGACA CT

TGGGTGAGACAATGGAGAA ACAACCTGATCGCTAAAAAAATCAACACTCCTGCTACTAAA

CTGTTCAAAAAAAACCCTAAAGCTAGACTGAACCTGTACCAAGTGAGAAAACTGGTG

**4 >evm-Contig2841_pilon-processed-gene-0.35-mRNA-1**

ATGTCAGAAAAACACCTGAAATCAGGTGACGTGCTGCCTCAATACAACGGTAAACTGAGA

CTGTTCGCTATGAGATTCTGCCCTTACGCTGAAAGATCAGTGCTGGTGCTGAACTCAAAA

GGTCTGGACTACGACCTGGTGTTCGTGAACCTGGACCACAAACC TGAATGGCTGTTCGAC

TTCTC ACCTAAAGGTACTGTGCCTGCTCTGGAATACGAACAAGGTAAAGCTATCTTCGAC

TCAAACATCATCAACGTGTACCTGGACGAAAAATACCCTGAAAAACCTCTGCAAGCTGCT

GACCCTCTGAGAAGAGCTCAAGACAAACTGATCGTGGAAAACTTCGCTGCTGCTCAATCA

GCTTACTACACTGCTGCTTTCAACTCACAAGCTCTGCAACCTTCACACATCGAAAACTAC

CACAAAGGTCTGGAAGGTCTGCAAAAAGAACTGGAAGTGAGAGGTACTAAATTCCTGCAC

GGTGACGACGCTGGTTGGGTGGACCTGACTATCTGGCCTTTCCTGGAAAGATTCCTGGCT

CTGCCTCTGCTGGGTAAACCTGAATTCGCTATCGACAAATCAAAATACGGTCTGCTGTCA

ACTTACATCGAAAACATGAAAAACGTGCCTGCTGTGAAATCATACTGCCTGGCTCCTGAA

ACTCACGCTAAATTCACTGAATCAAGAGCTAAAGGTGACCCTGACTACAACATGCTGGAC

ACTTCAGCTGTGTGCTGCATGAGACCTAGAAAAAAAAAAGAA

**5 >augustus_masked-Contig2596_pilon-processed-gene-0.0-mRNA-1**

ATGTCACTGAAACTGTACCACTTCCCTATCGCTGTGCACCACGCTGTGCTGTACCTGCTG

CAAGAACAATCAGGTTTCAGATCAAAAACTAACTCAGAATTCATCAAAAAAGAACAACTG

TCAGACTCATTCATCAAAATCAACCCTCAACACTGCGTGCCTACTCTGGAAGACGACGGT

TTCGTGCTGTGGGAATCAAGAGCTATCGCTTGCTACCTGGTGGACAAATTCGCTAAAGAC

GACCAACTGTACCCTAAAGACCTGGAAAAAAGAGCTCTGGTGAACCAAAGACTGTACTTC

GACTCATCATCACTGTACGTGAAAATCAGAGCTATCTGCTTCCCTATCCTGTTCCTGGGT

GAAACTGAAATCAAAAAAAACCTGAAAGACGACCTGAACGGTACTCTGGTGTTCCTGGAC

CAATTCCTGAACGACACTAAATGGG TGGCTGGTGACTCAATCACTATCGCTGACACTTCA

ATCTACGCTTCACTGTCATCAATCCTGGCTGTGGGTTGGGACATCACTAAATTCCCTAAC

ATCCAAAGATGGTCAAGAATCGTGAACCTGCCTGGTTACGCTGAAAACGAAGAAGGTGCT

AAAGCTTTCGGTGAAGCTGTGAAAAAAAACCTGAAACAA

**6 >maker-Contig14202_pilon-augustus-gene-0.24-mRNA-1**

ATGCACCTGATCTTCTTCTTCTCAGGTGACCCTCTGCCTCCTTACAACGGTACTCTGAGA

GTGTACAACATGAGATTCTGCCCTTTCGCTCAAAGAACTATCCTGGCTCTGAACGCTAAA

CAAGTGCACTACGAAGTGGTGAACATCAACCTGATGAACAAACCTGACTGGCTGTTCGAA

AAATCACCTTTCGGTAAAGTGCCTGCTCTGGAAATCAAAGAAGGTCAAACTATCTTCGAA

TCACTGGTGACTGTGGAATACCTGGACGA AGTGTACCCTAACAGACAACTGCTGTCAAAA

GACCCTGTGGAAAGAGCTTGCGACAGAATGACTGTGGAAGCTTCAGTGGCTATCCACAAC

CTGTTCTTCAAACTGGTGAAAAGACCTGAAGAAGTGTCAGACGAAAACATCGCTGCTTTC

AGAAAAGCTCTGGACTTCATCCAAGCTAAACTGGTGAAAAGAGGTTCAAAATTCCTGTTC

GGTAAAGAACCTGGTTACGTGGACTACATGATCTGGCCTTGGTTCGAAAGACTGCAAGCT

CTGGAAGACTCAAGAGCTCAACTGGACAAACAAAAATACGACACTCTGCTGAAATACATC

AACAACATGCTGCTGGACCCTTCAGTGTCAAAATACCTGGTGCCTGGTGACGTGTTCAAA

AAATTCCACTCAGGTTTCCTGCTGTCATCAGGTCCTGACTACGAACTGCTGAACAAA

**7 >maker-Contig1970_pilon-augustus-gene-0.34-mRNA-1**

CTGTACCCTGAAGAAGCTAAAGCTAGAGCTCTGGTGGACCAAAGACTGTACTTCGACATC

GGTACTCTGTACCAAAGATTCTCAGACTACTTCCTGAACCCTCAACACACTGTGCCTACT

CTGGTGGACGACGGTTTCTCAATCTGGGAATCAAGAGCTATCATCACTTACCTGGTGAAC

AAATACGGTAAAGGTAACCCTCTGTACCCTGAAGAAGCTAAAGCTAGAGCTCTGGTGGAC

CAAAGACTGTACTTCGACATCGGTACTCTGTACCAAAGATTCTCAGACTACTTCGAAAAA

CTGGCTAAAGTGGACGACGCTCTGAGACTGTTCGACGTGATCCTGGAAGGTCAAAAATAC

TCAGCTGGTAACAACCTGACTGTGGCTGACCTGTCACTGGTGGCTTCAATCTCATCATTC

GAAGCTTCAGACATCAACTTCCTGAAATACAACAACGTGAAAAGATGGTACGAAACTGTG

AAATCAACTGCTCCTGGTTACGAAG AAGCTAACGGTAAAGGTCTGCAAGCTTTCAAAGAA

CTGGTGAACAACATGCTGAAAAAA

**8 >evm-Contig1510_pilon-processed-gene-0.48-mRNA-1**

ATGGACAAATCAGTGGCTGCTAAAATCCAATCAGCTGACAACGCTGTGGCTAGATCAAAA

AACAAAATGCCTACTCAACCTATCAAACTGTACTACCTGCCTCCTTCACCTCCTTGCAGA

GCTGTGATGATGACTGCTAGAGTGCTGGGTATCGAACTGGAACTGATCGTGACTAACCTG

ATGGAAGGTGCTCACCTGGAACCTGAATTCGTGAAAATGAACCCTCAACACACTATCCCT

ACTATGGACGACTCAGGTTTCATCCTGTGGGAATCAAGAGCTATCATGACTTACCTGGTG

AACGCTTACGGTAGAGACGACACTCTGTACCCTAAAAACCCTAGACTGAGAGCTATCGTG

GACCAAAGACTGAACTTCGACCTGGGTACTCTGTTCATGAGATACATCAACCTGTACGGT

CCTATGCTGTTCAAAGGTGACAAACTGGACGACGAAAAAGCTGCTAAACTGAACGAAGCT

ATCGGTTGGCTGAACACTATGCTGGACGGTAAAGCTTTCGTGGCTGGTGACAACATGACT

GTGGCTGACATCTCAATGGTGGTGGTGTTCACTTGCCTGGAAGCTTTCGAATACGACTTC

TCACAATACGAAAACGTGACTAAATGGTTCGGTGTGATGAAAAAATCACTGGAACCTTAC

GGTTACTCAGAAATCGACGAAGCTGGTGCTCAAATGCTGGCTACTTT CCTGAAATCACAC

GCTAAC

**9 >evm-Contig2478_pilon-processed-gene-0.25-mRNA-1**

ATGGTGCTGACTCTGTACAAAAAAGACACTTCACCTCCTTGCAGATCAGTGTTCATGACT

ATCCACGCTCTGCACATCACTGACGTGAACTACATCGACGTGTACCTGCCTGCTGGTGAA

CACCTGACTGAAGAATTCATCAAACTGAACCCTCAACACACTGTGCCTCTGCTGAAAGAC

GGTGACTTCCTGATCTGGGACTCACACGCTATCTGCGGTTACCTGGTGTCAAAATACGGT

GAAAACGACCACCTGTACCCTAGAGACCCTAAAAAAAGAGCTATCGTGGACCAAAGACTG

CACTTCGACTCAGGTGTGCTGTTCAACGCTCTGAAAACTACTGTGGTGCCTCTGCTGTAC

GCTGGTGAAAAATCATTCAGACCTGAAAACCTGGCTCAAAT GAAAACTGCTTACGAATTC

ATGGAAAAATTCCTGACTACTCCTTGGCTGGCTGGTAAAGAAGTGACTCTGGCTGACATC

TGCTGCGTGTCATCAATCTCATCAATGAACGAAATCTTCCCTATCGAAGAATCAATCTAC

CCTCACCTGACTGGTTGGCTGAAAAGATGCTCAGAACAAGAATTCTTCGTGAAAGGTAAC

TCAAAACAAATCCTGCTGTTCAGAGAAATGATCACTAACAAACTGTCACAAAAC

**10 >maker-Contig3755_pilon-augustus-gene-0.39-mRNA-1**

ATGGTGCTGAAACTGTACGGTGTGTCAGACGGTCCTCCTTCACTGTCAGTGAGACAAGCT

CTGACTCACCTGCAAATCCCTTTCGAACTGATCAACATGAACCCTCAAAAAGAAATCCCT

GTGCTGGACGACGAAGGTTTCTACCTGGGTGAATCAAACGCTATCATGCAATACGTGTGC

GACAAATACAAACCTTCATCACCTCTGTACCCTCAACAACCTAAAGCTAGAGCTCTGGTG

AACCACAGACTGTGCTTCAACCTGTCAACTTACTACGCTCACATCTCAGCTTACACTATG

GCTCCTATCTTCTTCGACTACGAAAGAACTCCTCTGGGTCT GAAAAAAGTGCACATCGCT

CTGGACGTGTTCGAAACTTACCTGGAAAGACTGGGTACTGGTCACGCTGCTGCTGACCAC

CTGACTATCGCTGACTTCCCTCTGATCAACTCAACTATGACTCTGGAAGCTATCGACTTC

GACTTCAAAAAATACAAAAGAATCCACAAATGGTACAACGACTTCAAATCAAACTACCCT

GAACTGTGGAAAATCTCAGAAGGTGCTCTGAAAGAAATCCAACACTTCGCTGCTAACCCT

CCTGACCTGACTCACATGGACCACCCTATCCACCCTATCAGAAAAAACAACAAA

**11 >augustus_masked-Contig3537_pilon-processed-gene-0.3-mRNA-1**

ATGGAAGAAACTACTCTGTTCCCTTTCGAAAGAATCGCTTCACCTTCAGCTCTGCCTTAC

GGTCAACTGCCTCTGTACGAAGAAGGTGACAAATCACTGAACCAATCACTGGCTATCGCT

AGATACCTGGCTAACAAATTCAACCTGCTGCCTATCGACCCTTGGCAACAAGCTGTGCTG

GACGCTGTGGTGCTGAACATCTACGACTTCTGGGGTAAAATCCTGCCTTACTTCAGAGAA

CAAGACCCTGTGAAAAAAGCTGCTATCAGAGAAGAAATCATCAACGAAGTGATCCCTTTC

TACTTCTCAAGATTCGAAAGAGAACTGAAAGAAGCTAACGGTCACTTCGCTGGTAAACTG

TCATGGGTGATCTCATCACTGCTGGAACTGCTGAAACAACTGACTGCTACTGAAACTTGC

CAC

**12 >genemark-Contig3847_pilon-processed-gene-1.59-mRNA-1**

ATGACTATCACTAACGCTGGTGGTATCCCTGGTTCATCACCTCCTCCTCCTCTGGGTGAC

CAACTGAGACTGTACCACGTGGACATGAACCCTTACGGTCACAGAGTGCTGCTGATCCTG

GAAGCTAAAAAAGTGAAACACGAAGTGTACAGACTGGACCCTCTGAGACTGCCTGAATGG

TTCAGAGCTAGAAACCCTAGACAC**GACGACGGTGCTACTCACAA**ATTCCTGTTCGAATCA

GTGGTGATCTGCGACTACCTGGACGAAAGATACCCTAGAAACCCTAGACACTCAAAAGAC

CCTTACGTGAAAGCTAGAAGATTCATCCACAGAGAACTGATCAAAGGTTCACTGGAATGC

TTCGACACTAACTTCGCTTTCGGTTCAGAACAAATCGTGCAAACTCTGGACATCTTCGAA

AAAGAACTGGCTCTGAGAGGTATGCTGGACTACATGATCTGGCCTTGGGTGGAAAGACTG

TACCTGCTGAGATGCCTGAACGAAAAAAAATTCGACGAAAAAAGATCATTCATGCTGAAA

TGGCAATGGGCTGGTCACATCTGCAGAAGAACTTCAGGTAGATGGTCAAGAAGAGTGCTG

GAATGGAGACCTAGAAGAGGTAAACCTTCAGTGGGTCCTATGGACGGTAGACTGGGTAAA

GGTGGTTGGATCAGAAAAGCTGAAGACAGAGACATCTGGAGAGAACTGAGACTGCTGAGA

GACTCAAGAATGTCACAAAACAACGGTAGATGGTCAAGAAGAGTGTGGTCAGGTGACCCT

TTCGGTCAAAGATCAGTGGCTAGAAGAGACATCTGCGCTGAATGGGTGCTGTCAATCACT

ATCTCATACGCTATGATCTCAAAACTGAAATGGCAATGGGCTGGTCACATCGTGAGAAGA

ACTGACGGTAGATGGGGTGGTAAAGTGCTGGAATGGAGACC TAGAAACGGTAAAAGATCA

GTGGGTAGACCTCTGACTAGATGGACTGACCTGGTGAAAGTGGCTGGTTCAAGATGGATC

TCAACTGCTAGAGACAGATCAGCTTGGAAATCACTGGGTGAAGCTTTCGTGCAACAATGG

ACT

**13 >maker-Contig10769_pilon-augustus-gene-0.30-mRNA-1**

ATGGCTAGAAAACTGCAATACTTCAACCTGAACGGTCTGGCTGAACCTATCAGACTGATG

CTGCACTACGCTGGTTCAGAATTCGAAGACGTGAGATACGACTTCAAATCATGGCCTATC

AAATCAGTGAAAGACTCACTGCCTTACGGTCAAATGCCTATCTACGAAGAAAACGGTAGA

GTGCTGAACCAATCACTGGCTATCGCTAGATACATCGCTTCACAAAACGGTCTGCTGCCT

ACTGACTCATGGGACCAAGCTATCCTGGACGCTACTGTGAACAACATCTTCGACTTCTGG

TCAAACG TGGTGTCATACGTGAAAGAAACTGACGCTGAAAAAAAAAAAGCTATCAAAAAA

CAAATCCTGGAAGAACTGGTGGACTACTACTTCTCAAGATTCGAAAAAGAACTGAACGCT

CACGGTGGTTTCTTCGTGGGTCAACTGTCATGGGCTGAATTCGTGCTGGTGGGTATCATG

GAATCATGCGACCTGTTCCTGGAAGAAAAAATCTCATACCAAAACTACCCTGCTGTGCAA

GGTCTGTTCAACAAAATCCTGTCACTGCCTGGTGTGAAAGAATACGTGGCTTCAAGACCT

CCTTACGTGTGGCCTCAAAAC

**14 >evm-Contig1010_pilon-processed-gene-1.18-mRNA-1**

ATGGGTATCGACCTGTACTACGTGCCTGGTTCAGCTCCTTGCAGAGCTGTGCTGCTGACT

GCTAGAGCTCTGAACATCAACCTGAACCTGAAACTGGTGGACCTGCACCACGGTGAACAC

CTGAAACCTGAATACCTGAAACTGAACCCTCAACACACTGTGCCTACTCTGGTGGACGAC

GGTTTCTCAATCTGGGAATCAAGAGCTATCATCACTTACCTGGTGAACAAATACGGTAAA

GGTAACCCTCTGTACCCTGAAGAAGCTAAAGCTAGAGCTCTGGTGGACCAAAGACTGTAC

TTCGACATCGGTACTCTGTACCAAAGATTCTCAGACTACTTCTACCCTCAAGTGTTCGGT

GGTGCTCCTGCTGACAAAGAAAAACTGGCTAAAGTGGACGACGCTCTGAGACTGTTCGAC

GTGATCCTGGAAGGTCAAAAATACTCAGCTGGTAACAACCTGACTGTGGCTGACCTGTCA

CTGGTGGCTTCAATCTCATCATTCGAAGCTTCAGACATCAACTTCCTGAAATACAACAAC

GTGAAAAGATGGTACGAAACTGTGAAATCAACTGCTCCTGGTTACGAAG AAGCTAACGGT

AAAGGTCTGCAAGCTTTCAAAGAACTGGTGAACAACATGCTGAAAAAA

**15 >evm-Contig9166_pilon-processed-gene-0.22-mRNA-1**

ATGGACAAATCAGTGGCTGCTAAAATCCAATCAGCTGACAACGCTGTGGCTAGATCAAAA

AACAAAATGCCTACTCAACCTATCAAACTGTACTACCTGCC TCCTTCACCTCCTTGCAGA

GCTGTGATGATGACTGCTAGAGTGCTGGGTATCGAACTGGAACTGATCGTGACTAACCTG

ATGGAAGGTGCTCACCTGGAACCTGAATTCGTGAAAATGAACCCTCAACACACTATCCCT

ACTATGGACGACTCAGGTTTCATCCTGTGGGAATCAAGAGCTATCATGACTTACCTGGTG

AACGCTTACGGTAGAGACGACACTCTGTACCCTAAAAACCACGAC

**16 >maker-Contig6423_pilon-augustus-gene-0.33-mRNA-1**

ATGTCACTGAAACTGTACCACTTCCCTATCTCAGGTCCTTCAAGAGGTGCTCTGCTGGCT

GCTAGAGCTATCGGTGTGCCTATCCAAATCCAAATCGTGAACCTGTTCAAAAAAGAACAA

CTGTCAGACTCATTCATCAAAATCAACCCTCAACACTGCGTGCCTACTCTGGAAGACGAC

GGTTTCGTGCTGTGGGAATCAAGAGCTATCGCTTGCTACCTGGTGGACAAATTCGCTAAA

GACGACCAACTGTACCCTAAAGACCTGGAAAAAAGAGCTCTGGTGAACCAAAGACTGTAC

TTCGACTCATCATCACTGTACGTGAAAATCAGAGCTATCTGCTTCCCTATCCTGTTCCTG

GGTGAAACTGAAATCAAAAAAAACCTGAAAGACGACCTGAACGGTACTCTGGTGTTCCTG

GACCAATTCCTGAACGACACTAAATGGG TGGCTGGTGACTCAATCACTATCGCTGACACT

TCAATCTACGCTTCACTGTCATCAATCCTGGCTGTGGGTTGGGACATCACTAAATTCCCT

AACATCCAAAGATGGGTGAAAGACTGCGAATCACTGCCTGGTTACGCTGAAAACGAAGAA

GGTGCTAAAGCTTTCGGTGAAGCTGTGAAAAAAAACCTGAAACAA

**17 >maker-Contig199_pilon-augustus-gene-0.91-mRNA-1**

ATGCCTAAAGTGAAATACTCATACTTCCCTGTGAAAGCTCTGGGTGAATCAGCTAGACTG

CTGCTGTCATACGGTGGTCAAGAATTCGAAGACAACAGAGTGTCAATGGAAGACTGGCCT

CAATTCAAACCTAAAATCGACGGTAAAAAATACGCTCAATCAGGTGCTATCGCTAGATAC

CTGGGTAGAAAATACGGTATCGCTGGTTCATCAATCGAAGAAGACCTGGAAATCGACATG

AACGTGGACTTC ATCAACGACATCAGAGCTAAAGCTGCTCTGGTGGCTTACGAAGCTGAC

CCTGAACTGAAAAAAAAAAAACACGAAGACTTCTCAAAAAACGTGTACCCTCAACTGCTG

AAAAGACTGTCAGAACTGTTCGAATCAAACAACGGTCACCTGACTTGGGGTGACTTCGTG

TTCGCTGGTATGTACGACTACCTGAAAATGATGATGCAATCACCTGACCTGGACGCTCAA

TACCCTATCTTCAAAAAAGTGGCTGACAACGTGTACTCACTGCCTCAACTGCAATCATAC

CTGGCTGCTGCTCCTAAAACTGACTTC

**18 >augustus_masked-Contig8048_pilon-processed-gene-0.1-mRNA-1**

ATGCTGGACACTTACCAACACAACACTGTGTTCGGTCAACTGCCTCTGCTGGAAATCGAC

GGTAAAAAATACGCTCAATCAGGTGCTATCGCTAGATACCTGGGTAGAAAATACGGTATC

GCTGGTTCATCAATCGAAGAAGACCTGGAAATCGACATGAACGTGGACTTCATCAACGAC

ATCAGAGCTAAAGCTGCTCTGGTGGCTTACGAAGCTGACCCTGAACTGAAAAAAAAAAAA

CACGAAGACTTCTCAAAAAACGTGTACCCTCAACTGCTG AAAAGACTGTCAGAACTGTTC

GAATCAAACAACGGTCACCTGACTTGGGGTGACTTCGTGTTCGCTGGTATGTACGACTAC

CTGAAAATGATGATGCAATCACCTGACCTGGACGCTCAATACCCTATCTTCAAAAAAGTG

GCTGACAACGTGTACAAACTGCCTCAACTGCAATCATACCTGGCTGCTGCTCCTAAAACT

GACTTC

**19 >maker-Contig11878_pilon-exonerate_protein2genome-gene-0.11-mRNA-1**

ATGTCACTGAAACTGTACCACTTCCCTATCTCAGGTCCTTCAAGAGGTGCTCTGCTGGCT

GCTAGAGCTATCGGTGTGCCTATCCAAATCCAAATCGTGAACCTGTTCAAAAAAGAACAA

CTGTCAGACTCATTCATCAAAATCAACCCTCAACACTGCGTGCCTACTCTGGAAGACGAC

GGTTTCGTGCTGTGGGAATCAAGAGCTATCGCTTGCTACCTGGTGGACAAATTCGCTAAA

GACGACCAACTGTACCCTAAAGACCTGGAAAAAAGAGCTCTGGTGAACCAAAGACTGTAC

TTCGACTCATCATCACTGTACGTGAAAATCAGAGCTATCTGCTTCCCTATCCTGTTCCTG

GGTGAAACTGAAATCAAAAAAAACCTGAAAGACGACCTGAACGGTACTCTGGTGTTCCTG

GACCAATTCCTGAACGACACTAAATGGG TGGCTGGTGACTCAATCACTATCGCTGACACT

TCAATCTACGCTTCACTGTCATCAATCCTGGCTGTGGGTTGGGACATCACTAAAGTGTCA

**20 >evm-Contig7006_pilon-processed-gene-0.0-mRNA-1**

ATGGCTAAAAAACTGCACTACTTCCACCTGAACGGTATCGCTGAATCAATCAGATACATC

CTGCACTACGCTGGTGAAAAATTCGAAGACGTGAGATACGAAATCTCAGCTTGGCCTATC

CAATCAGTGAAAGAATCACTGCCTTACGGTCAACTGCCTCTGTACGAAGAAGGTGACAAA

TCACTGAACCAATCACTGGCTATCGCTAGATACCTGGCTAACAAATTCAACCTGCTGCCT

ATCGACCCTTGGCAACAAGCTGTGCTGGACGCTGTGGTGCTGAACATCTACGACTTCTGG

G GTAAAATCCTGCCTTACTTCAGAGAACAAGACCCTGTGAAAAAAGCTGCTATCAGAGAA

GAAATCATCAACGAAGTGATCCCTTTCTACTTCTCAAGATTCGAAAGAGAACTGAAAGAA

GCTAACGGTCACTTCGCTGGTAAACTGTCATGGGCTGACTTCATCCTGGTGGGTATCGTG

GAAGCTGCTAACCTGCTGCTGAAACACGTGATCGAAAAAGACTACCCTGTGGTGGAATCA

CTGATCAAAAAAGTGCAATCACTGCCTGGTGTGAAAGAATACATCGCTTCAAGAAAACCT

TACGCT

**21 >evm-Contig3023_pilon-processed-gene-0.16-mRNA-1**

ATGGTGATCAGACTGTACTACGACCTGATGTCACAACCTTCAAGAACTCTGTACATCCTG

TTCAAAACTATCAAATGCGACTGGGAACCTAAATACGTGAACCTGAGAAAAGGTGAACAC

TACTCAGACGACTTCACTAAAATCAACAAAATCCAAAGAGTGCCTGTGATCGACCACAAC

GGTTTCATCCTGTCAGAATCAGTGGCTATCGTGAAATACCTGTCAAGAGAAAACATCATC

CCTGAATCACTGTACCCTAGAGAATCAAAACTGAGAGCTAGAGTGGACGAATTCCTGGAA

TGGCACCACATCGGTCTGAGACTGCACTGCGCTATGTACTTCAGAATCAAATACATGGAC

CCTATCCTGTTCGGTAGAAAAACTGAAGCTAAACAAATCGCTGCTTACGAAGGTAGAATG

GTGAAAGCTCTGGAAGACTTCGACGAAAAATGGCTGGGTAGAGGTAACCAATACGTGGTG

GGTGACACTATCACTATCGCTGACCTGTTCGCTGCTACTGAACTGGAACAACCTAGAATG

GCTGGTTACGACCCTAAAGAACACTTCCCTGC TATCGGTACTTGGGCTAAAAAAGTGAGA

GAACACTTCAACCCTCACTACGACGAAGCTCACGTGGTGGTGAACAAAATCATCGAAAAA

CAAAGACAATCAAAACTG

**22 >augustus_masked-Contig2596_pilon-processed-gene-0.0-mRNA-1**

ATGTCACTGAAACTGTACCACTTCCCTATCGCTGTGCACCACGCTGTGCTGTACCTGCTG

CAAGAACAATCAGGTTTCAGATCAAAAACTAACTCAGAATTCATCAAAAAAGAACAACTG

TCAGACTCATTCATCAAAATCAACCCTCAACACTGCGTGCCTACTCTGGAAGACGACGGT

TTCGTGCTGTGGGAATCAAGAGCTATCGCTTGCTACCTGGTGGACAAATTCGCTAAAGAC

GACCAACTGTACCCTAAAGACCTGGAAAAAAGAGCTCTGGTGAACCAAAGACTGTACTTC

GACTCATCATCACTGTACGTGAAAATCAGAGCTATCTGCTTCCCTATCCTGTTCCTGGGT

GAAACTGAAATCAAAAAAAACCTGAAAGACGACCTGAACGGTACTCTGGTGTTCCTGGAC

CAATTCCTGAACGACACTAAATGGG TGGCTGGTGACTCAATCACTATCGCTGACACTTCA

ATCTACGCTTCACTGTCATCAATCCTGGCTGTGGGTTGGGACATCACTAAATTCCCTAAC

ATCCAAAGATGGTCAAGAATCGTGAACCTGCCTGGTTACGCTGAAAACGAAGAAGGTGCT

AAAGCTTTCGGTGAAGCTGTGAAAAAAAACCTGAAACAA

**23 >maker-Contig2478_pilon-exonerate_protein2genome-gene-0.15-mRNA-1**

TCATCACACGCTATCACTACTTACCTGATCACTAAATACGGTAAAGACGACTCACTGTAC

CCTTCAGACCCTAAAATCAGAGCTAGAATCGACCAAAGACTGC ACTTCGACTCAGGTGTG

CTGTTCGCTACTCTGAGAAACGCTGTGGGTTCAATCCTGTACCAAGGTGAAACTACTCTG

ACTCCTGAACACTCAGAAAAAATCAAAAACGCTTACGAATTCACTGAAAAATTCCTGACT

TCACAATGGCTGGTGGGTGACCACGTGACTATCGCTGACATCTGCTGCGTGGCTACTATC

TCATCAATGAACGTGCTGCTGCCTATCGACGAAGGTGTGTACCCTAACCTGGCTGCTTGG

GTGGAAAGATGCTCAGCTCTGGACTCAACTCTGAAAGAAACTAACCTGGACCTGAACAAC

TCACTGAACTCACTGAAAGCTAACTGCGACAAATTC

**24 >Contig3291-0.7**

ATGTCAGACATCAGAGTGGAACTGCCTCCTGAACAAAGAAGATCATCATCAAGATTCGAA

ATCTACCTGTCAGAAAAATTCAACTACGGTGTGACTCTGGTGGAATCAGTGGCTATCATC

CAATACCTGGACGACACTAGACCTAAACCTGCTCTGTTCCCTCAATCACCTCTGCAAAAA

GCTAGAACTATCGTGTCAGGTATCCAACCTCTGCAAAACGTGGGTCTGAAATCATCATTC

GAAAGAGAAGAAGACTTCCAAAAATTCTCAAAATCATGGTGCGACAGAGGTCTGCAAACT

GTGGAAGAACTGCTGAAAGCTTCAGCTGGTTCATACTGCGTGGGTGACCAACTGACTCTG

GCTGAC CTGTGCCTGGTGCCTCAAATCTTCAACGCTACTACT

**25 >Contig4841-1.1**

GGTGTGACTCTGGTGGAATCAGTGGCTATCATCCAATACC TGGACGACACTAGACCTAAA

CCTGCTCTGTTCCCTCAATCACCTCTGCAAAAAGCTAGAACTATCGTGTCAGGTATCCAA

C CTCTGCAAAACGTGGGTCTGAAATCATCATTCGAAAGAGAAGAAGACTTCCAAAAATTC

TCAAAATCATGGTGCGACAGAGGTCTGCAAACTGTGGAAGAACTGCTGAAAGCTTCAGCT

GGTTCATACTGCGTGGGTGACCAACTGACTCTGGCTGACCTGTGCCTGGTGCCTCAAATC

TTCAACGCTACTACTAGATACCTGAAC

**26 >evm-Contig2323_pilon-processed-gene-0.23-mRNA-1**

ATGGTGCTGACTCTGTACAAACTGGACGCTTCACCTCCTGTGAGAGCTGTGAACATGGTG

ATCGAAGCTCTGGGTCTGGAAGTGGAAACTGTGACTGTGAACATCCTGAACAAAGACAAC

CACAAAGACGAATACCTGCAAATGAACCCTCAACACACTATCCCTACTCTGAAAGACGAC

GACTTCGTGATCTGGGACTCACACGCTATCACTACTTACCTGATCACTAAATACGGTAAA

GACGACTCACTGTACCCTTCAGACCCTAAAATCAGAGCTAGAATCGACCAAAGACTGCAC

TTCGACTCAGGTGTGCTGTTCGCTACTCTGAGAAACGCTGTGGGTTCAATCCTGTACCAA

GGTGAAACTACTCTGACTCCTGAACACTCAGAAAAAATCAAAAACGCTTACGAATTCACT

GAAAAATTCCTGACTTCACAATGGCTGGTGGGTGACCACGTGACTATCGCTGACATCTGC

TGCGTGGCTACTATCTCATCAATGAACGTGCTGCTGCCTATCGACGAAGGTGTGTACCCT

AACCTGGCTGCTTGGGTGGAAAGATGCTCAGCTCTGGACTTCTACATCAAAGGTAACAAA

CCTGGTCTGGAACAATTCACTCAACTGATCAAATCAAAACTGAGA

**RESITANCE GENES OF CORBOXYLESTERASE**

**Total number of COE genes in *Leucinodes orbonalis* is 94**

**Total number of insecticide response genes predicted by BLASTX is 16.**

**The below given sequences are matching with the insecticide resistance in NCBI-BLASTX hit**

**1>augustus_masked-Contig8459_pilon-processed-gene-0.14-mRNA-1**

ATGTCAGCTCTGCTGATGTCACTGGACTACAAAGGTGTGCTGTGCTGCTTCCTGGAACCT

GACTGCATCTTCGGTATCAACACTGACCCTCTGGTGCTGATCCAACAAGGTCTGGTGAGA

GGTCAAAGAGCTACTGACGGTGACTACACTACTTTCCTGGGTATCCCTTACGCTGTGGTG

GACGAAGACAACCCTTTCGGTCCTGCTACTCCTCACACTGCTTTCGAAGAACAAGTGTAC

AAAGCTTACAACGGTTCAATCGCTTGCCCTCAAACTCCTCTGTTCAACCACATCCCTGAA

GGTGGTGCTCAATCACTGGACTGCCTGAGACTGAACATCTACGTGCCTAACGAAGCTACT

TCAAAAGCTCCTCTGCCTGTGCTGGCTTGGATCCACGGTGGTCTGTTCGGTTACGGTTTC

GGTGGTGAATTCGGTGTGAGAGACCTGGTGAAACAAGGTATCCTGGTGGTGACTGTGAAC

TACAGACTGGGTCCTTACGGTTTCATGTGCCTGAACACTCCTACTATGCCTGGTAACCAA

GGTCTGAAAGACCAATTCATGGCTCTGAGATGGATCAGAAACAACATCGCTTCATTCGGT

GGTAACCCTTACAACGTGACTCTGGCTGGTCAATCAGCTGGTGCTTGCTCAGCTATCCTG

CACCTGTACTCATCAAGAGAAAAACTGTACCACAAAGTGATCGCTGAATCAGGTACTCCT

CTGAACCCTGGTCTGTTCGTGGACGGTGACGCTAACGCTGCTATCAAACTGGCTAACTAC

CTGGGTATGAACACTTCAGACACTGACTCAGCTCTGAAATTCCTGGCTACTTCACCTCAC

ACTCTGGTGACTGGTGCTGCTTCAGCTCTGAACATCCAATTCAGAGCTTGCAAAGAAAAA

TCATTCTCAGGTGTGGACCACTTCGTGGACTCAGACCCTTACTCAATGACTAACGAAAGA

AAAATCAGAAACACTCCTATCCTGATCGGTCACACTTCAAAAGAATCAATCAACACTATC

AAAAACTTCCACGACTACTTCAACAGAGACCCTTTCTTCGAAAACGTGGCTAACAACTTC

AACTTCGACTCAGACAAACTGAACAAAGTGGCTTCAACTATCAGACACTTCTACATCGGT

GACAAAGGTATCACTGAACAAGTGACTTCAAAACTGGAAGACTTCGAATCAGACTTCGTG

TTCAACCACCCTATCCAAGTGACTGCTATGAACCTGCTGAAAGAAAACGCTAACCCTGTG

TACGAATACATGTTCTCATACGTGGGTGTGAAAAGAGCTAAACCTCTGCTGGACTCAGAA

CTGCTGGTGCCTGTGGCTGAATTCGCTGGTGCTGTGCACGCTAGACAATACGAAGCTAAC

CAATACGTGTCAAACGTGTTCAGAATCGACCCTCTGGTGCTGATCCAACAAGGTCTGGTG

AGAGGTCAAAGAGCTACTGACGGTGACTACACTACTTTCCTGGGTA TCCCTTACGCTGTG

GTGGACGAAGACAACCCTTTCGGTCCTGCTACTCCTCACACTGCTTTCGAAGAACAAGTG

TACAAAGCTTACAACGGTTCAATCGCTTGCCCTCAAACTCCTCTGTTCAACCACATCCCT

GAAGGTGGTGCTCAATCACTGGACTGCCTGAGACTGAACATCTACGTGCCTAACGAAGCT

ACTTCAAAAGCTCACTACAGATCATGGCACGGTTTCATGGCTGTGTACTCAGTGATGGAC

TCAGGTGAAAACTCAGTGAACAACATCGCTTCATTCGGTGGTAACCCTTACAACGTGACT

CTGGCTGGTCAATCAGCTGGTGCTTGCTCAGCTATCCTGCACCTGTACTCATCAAGAGAA

AAACTGTACCACAAAGTGATCGCTGAATCAGGTACTCCTCTGAACCCTGGTCTGTTCGTG

GACGGTGACGCTAACGCTGCTATCAAACTGGCTAACTACCTGGGTATGAACACTTCAGAC

ACTGACTCAGCTCTGAAATTCCTGCAACTGGCTCCTACTCTGGTGACTGGTGCTGCTTCA

GCTCTGAACATCCAATTCAGAGCTTGCAAAGAAAAATCATTCTCAGGTGTGGACCACTTC

GTGGACTCAGACCCTTACTCAATGACTAACGAAAGAAAAATCAGAAACACTCCTATCCTG

ATCGGTCACACTTCAAAAGAATCAATCAACACTATCAAAAACTTCCACGACTACTTCAAC

AGAGACCCTTTCTTCGAAAACGTGGCTAACAACTTCAACTTCGACTCAGACAAACTGAAC

AAAGTGGCTTCAACTATCAGACACTTCTACATCGGTGACAAAGGTATCACTGAACAAGTG

ACTTCAAAACTGGAAGACTTCGAATCAGACTTCGTGTTCAACCACCCTATCCAAGTGACT

GCTATGAACCTGCTGAAAGAAAACGCTAACCCTGTGTACGAATACATGTTCTCATACGTG

GGTGACTCAGGTGAAGAAGGTGCTGGTCACTCATCAGAACTGAACTACCTGTTCAACATG

CTGGGTAAAACTCAAAGAACTGCTGAAGACCAACTGATCGCTGACAGAATCACTTCACTG

TGGGCTAACTTCGTGAAATTCGGTAACCCTACTCCTAGACACAACAACCCTCTGTCAGTG

ACTTGGAACCCTATCTCAACTGTGACTAGACCTTACCTGGTGATCGACAAAGACATCAGA

CTGGAATCAAGAGTGTACAACGAAAGAATGGCTTTCTGGGAACTGTTCCACGACAACTAC

GGTACTTACAACAAACACAAAAGACAATGCACTATC

**2 >maker-Contig12636_pilon-augustus-gene-0.8-mRNA-1**

ATGCACCTGTTCATCCTGCTGTCAGTGCTGGCTCTGGCTCCTCTGCCTGCTAAAGCTTGG

AAAGGTACTAGAAACGCTACTGAACACGGTCCTATCTGCATCCAATACGACGTGGTGACT

AAACACATCCTGCCTGGTTCAGAAGACTGCCTGTTCCTGAACGTGTACACTCCTAAACTG

AAACCTACTAAACCTCTGCCTGTGCTGTTCTTCATCCACGGTGGTGGTTGGAAATCAGGT

TCAGGTAACGACGACGAATACGGTCCTGACTTCCTGGTGCAATACGACATCGTGCTGATC

ACTATCAACTACAGACTGGACGTGTTCGGTTTCCTGACTATGGACAACGAAGACGTGCCT

GGTAACGCTGGTATGAAAGACCAAGTGCTGGCTCTGAAATGGGTGAACAACAACATCAAA

TACTTCGGTGGTGACCCTGAAAACATCACTATCATGGGTCAATCAGCTGGTTCAGCTTCA

GTGATCTACCACATGATGTCACCTATGTCAAAAGGTCTGTACAAAAGAGCTATCGCTATG

TCAGGTGTGCCTCTGTCAGAATGGGCTCAACCTTTCGAAGCTCCTAGAAGATCATTCTCA

CTGGGTACTATCCTGGGTAACCCTACTACTGACCCTAAAGAACTGATCAAATTCCTGAGA

AACGTGCCTGCTGAAAACCTGCTGAACACTAAACCTGCTGTGATCTCACAAGAAGAACTG

TGGATCAACCCTATCAAAATGTACGCTCTGGTGCCTGTGGTGGAAAGAGACTTCGGTCAA

GAAAGATTCCTGGTGGAAACTCCTACTGAATCAATCATCAACGGTAACGTGAGAGACCAA

GAACTGATCATCGGTCTGACTTCAATGGAATTCATCTACGCTATCCCTTGGCTGGAAGAC

GACCACGTGGCTAGATTCAACCTGGCTCTGGACCTGCTGGTGCCTCTGGACCTGCTGAGA

GAAATCACTCCTAAAGAAGTGGGTGCTCTGTCAGACGTGATCAGAAAAAGATACTTCGGT

GACAAACCTATCACTAACTCATCAATCGTGGGTTTCGTGCACCTGTTCCACGACACTCAA

TACGTGCACTCAACTAACAGATACCTGAAATACCTGCAACCTGGTAAATCAGACAAATAC

TTCTACAACTTCTTCACTGTGTCAGGTAGAAACCTGTACACTAAATACGGTGACAAATAC

GGTATCCACGGTTGCGGTCACACTGACGACCTGAGATACCTGTTCGACGCTAAATCATCA

AAAACTTCAGTGGACCCTAACTCAATCGAATACAGACTGATCAAACAAA CTTGCACTCTG

TTCACTAACTTCGTGAAATACGGTGAAGACTGCCTGTTCCTGAACGTGTACACTCCTTGG

ATCAACCCTAAATCACCTCTGCCTGTGCTGTTCTTCATCCACGGTGGTGCTTGGAAATCA

GGTTCAGGTAACGACGACAACTACGGTCCTGACTTCCTGATGCACTACGACATCATCCTG

GTGACTATCAACTACAGACTGGACGTGCTGGGTTTCCTGTCACTGGACACTAAAGACGTG

CCTGGTAACGCTGGTATGAAAGACCAAGTGCTGGCTCTGAAATGGGTGCACGACAACATC

GGTAACTTCGGTGGTGACCCTAACCAAGTGACTATCATGGGTCAATCAGCTGGTGGTGCT

TCAGTGATCTACCACATGATGTCAACTGTGTCAAGAGGTCTGTTCAAAAGAGCTATCTCA

ATGTCAGGTGTGCCTCTGTCAGACTGGGCTTTCCCTTTCGAACAAACTACTAGAGCTTAC

GAACTGGCTAAAACTATGGGTCAAAACATCGACAACTCAGAAGACCTGCTGAACTACCTG

CAAAACGTGCCTGCTCACGACCTGCTGAACACTAAACCTGCTGTGCTGTCATCAGAAGTG

TACTGGAACAACGGTATCAAAATGTTCTCATTCGTGCCTGTGATCGAAAAAGACTTCGGT

AAAGAACAATTCCTGGTGGAACCTATCAAAGAAGCTCTGGTGAACGGTAACATCGAAGAC

ACTGACCTGCTGATCGGTTACACTGACCTGGAATACCTGATCGCTGTGCCTCTGCTGGAA

TCATACGGTCTGGCTGACTACAACATGTTCAAAGAACTGCTGGTGCCTAGAGAAATCCTG

CTGGAAATCTCACCTAAAAGAATGCTGTCACTGGCTGAAAAAATCCACAAAGCTTACTTC

GGTAACCAATTCATCAACAACACTACTATGCTGAACTTCCAAAACTACGGTGGTGACCAC

ATCTTCATCAACTCAGTGCTGAGATTCCTGAGATACCTGCCTAAAGGTAGAGCTAAAAGA

TACCTGTACAAATTCTTCACTGAATCATCAAGAAACAGATACCTGAAAAACGCTGAAAAA

TACAACATCCACGGTTGCGCTCACACTGACGACCTGATGTACCTGTTCGACGCTAAACAA

GAAGCTACTGCTATGAACGTGGACTCAGACGAATACCAACTGATCAACCAAA CTTGCACT

CTGTTCACTAACTTCGTGAAATACGGTAACCCTACTCCTGACGACGCTCTGGGTGTGACT

TGGCCT GAATACAACAACGACGACAGACCTTACCTGTCAATCAACAACAAACTGACTACT

GGTACTCACCTGGACTTCGACGTGACTAACTTCTGGGACGACATCTACAGATCATGCGAC

ATCGAACTGCCT

**3 >augustus_masked-Contig11486_pilon-processed-gene-0.8-mRNA-1**

ATGACTGAAGACTGCCTGGTGGCTAACGTGTACGTGCCTATCTCACAAGAAACTCACCTG

CCTGTGATCGTGTTCGTGCACGGTGGTGCTTACATCGGTGGTTCAGCTCTGAACGACTCA

CCTAAAAACCTGGTGAACACTCAAAAAGTGATCGCTGTGAACTTCAACTACAGACTGAAC

GTGCACGGTTTCCTGTGCCTGGGTACTGAAGACGCTCCTGGTAACGCTGGTATGAAAGAC

CAAGTGGCTCTGCTGAGATGGGTGAAAGAAAACATCGCTTCATTCGGTGGTAACCCTAAC

GACGTGACTATCACTGGTTGCTCAGCTGGTGGTTCAGCTGTGGACCTGCTGATGATCTCA

AAAATCACTGACGGTCTGTTCAACAAAGTGATCTCAAACTCAGGTTGCGGTATCTCAACT

TTCGGTACTCAACTGAACCCTCTGGAAAACGCTAAACTGCACGCTTCACTGCTGAACGCT

ACTAACGTGGACGACATCAAATCACTGGCTGAATTCTACAAAACTCACCCTCTGTCAGAA

CTGATCCAAGAAAGAGACCTGGGTCAAGAAATGTTCCTGGACGACGCTCCTATCAACATC

TACAAAAAAGGTGACTACAAAAAACTGCCTGTGGTGTACGGTTACGCTAACATGGAAGGT

CTGTTCAGACTGCCTCTGTTCTACCTGTGGAAAGACCTGATGAACGAAAAATTCTCAGAC

TTCCTGCCTGCTGACCTGCAATTCGAATCAGACGCTCAAAAAGAAGAAGTGGCTCTGTCA

ATCAAAAACTTCTACTTCGGTGACAAACCTGTGAACGAATCAACTATCCTGCAATACATC

GAACTGCACACTGACCTGCTGTTCCTGAACTCAATCTACAGAACTGTGACTCTGCAAGTG

CAAGCTGGTAACAACCAAGTGTACCTGTACGACTACTCATTCGTGACTAACTCAACTGCT

GTGGTGCCTCACACTGACTCAGTGAGAGGTGTGGACCACTGCTTCCAAACTGTGGGTCTG

ATGGACGTGCCTCCTGCTTACGAAGACCTGGTGGACGACGAATCACTGTACATGGCTAAC

ATCACTAGACAACTGTGGATCAACTTCATCACTACTGGTAACCCTACTCCTCCTGGTCAC

GAATCATCAGTGCCTACTTGGCTGCCTGCTGGTGAAAACAGATCACCTCACCTGTCAATC

GGTCAAGTGATCCAACTGGCTGGTGTGCCTGCTGAAAACAGATCACTGTTCTGGGACGGT

ATCTACGAAAGACACTACAGAATGCCTATGCCTCCTCCTAACCACTCAGGTGCTGCTTCA

GCTTACCTGATGTCACTGAACACTTCAGGTCCTCTGTCAGCTTCATCATGGGTGAACGTG

TACGACGCTGTGGACAAAGGTATCATCTGCCCTCAACCTACTATCAACAACCTGACTATC

GGTGGTTCAGTGAAACTGGTGGCTGACGAAAACTGCCTGATCGCTAACATCTACGTGCCT

GACACTGAAGAAAAAGACCTGCCTGTGATCGTGTACGTGCACGGTGGTGCTTACCAAGTG

GGTTTCGGTAACATCCTGCCTCCTAAAAACCTGGTGAAATCAGGTAAAGTGATCGCTGTG

ACTTTCAACTACAGACTGGGTATCCACGGTTTCCTGTGCCTGGGTACTACTGACGCTCCT

GGTAACGCTGGTATGAAAGACCAAGTGGCTCTGCTGAGATGGGTGAAAAACAACATCGCT

TCATACGGTGGTAACCCTGGTGACGTGACTATCGCTGGTTACTCAGCTGGTTCATCAGCT

GTGGACCTGCTGATGCTGTCACCTTCAGCTCAAGGTCTGTTCAACAAAGTGATCCCTGAA

TCAGGTGCTAACATCTCACCTTGGTCAGTGCAAGTGGACCCTATCGAAAACGCTAAAGAA

TTCGCTAGATCAGCTGGTCTGGAAGACACTGACGACCTGTACGCTCTGGAAGAATTCTAC

AAAACTGCTTCATTCAAACTGTTCACTTCAACTGGTGGTTTCTTCAACAGAACTGACGCT

AACTTCCTGTTCACTCCTTGCGTGGAAAGAGAAACTGGTGACGGTGCTTTCCTGACTGAC

GCTCCTTACAACATCCTGAAAAGAGGTGACTACAGAAAAGTGCCTGTGCTGTACGGTTTC

GCTAACATGGAAGGTCTGCTGAGAATCCCTGAATTCGACACTTGGAAAGACAAAATGAAC

GCTAACTTCTCAGCTTTCCTGCCTGCTGACCTGAAATTCGTGAACACTGAAGAAAGAAAC

GAAGTGTCAAAAAAAATCAAAGAATTCTACTTCTCAGACATGCCTGTGTCAGAAGACAAC

ATCCTGTCATACATCGACTTCCAATCAGACGTGTACTTCGGTTACTCATCACTGAAAGCT

GTGAGACTGCACGTGCAAACTGGTCACGACAAAATCTACCTGTACGAATACGACTACGTG

GACGAATCAACTCCTGCTATCCCTCACACTGACAACGTGAGAGGTGCTAACCACTGCGCT

CAAACTATGGCTGTGCTGCAACCTGAAGACACTCTGTCAGAAGACTCAAAAACTGTGTCA

AGAACTATGCAAGAAATCTGGCTGAACTTCATCACTACTGGTTCACCTGTGCCTGTGGGT

TCATCACTGCCTGCTTGGCCTCCTGCTGGTGCTGACGGTTCACCTCACATGAGACTGGGT

AGAACTGTGGAACTGAGAGGTGCTCTGCTGGCTGACAGAGTGAGATTCTGGGACTCAATC

TACGACCACCAACCTAGAGCTGCTCCTCCTCCTAGAAGACCTCTGATCATCCCTAACTAC

AAATACGAAAACAGAGTGGTGATGCTGAGAATCAACGCTACTTACGTGCTGTACTTCTAC

GACGTGGAATCAAAAAAAAGAAAAAAAAGAATCTACCCTCTGTCATGCATGTTCACTGCT

GTGCTGATCAAACTGGACCTGGTGTCACTGGACTTCTTCGCTTGGGGTACTAAAGACGTG

CCTGGTAACGCTGGTATGAAAGACCAAGTGGCTCTGCTGAGATGGGTGAACCACAACATC

GGTTCATACGGTGGTAACCCTGACGACGTGACTATCGCTGGTTCATCAGCTGGTTCATCA

TCAGTGGACCTGCTGATGCTGTCACCTTCAGCTCAAGGTCTGTTCAACAAAGTGATCCCT

GAATCAGGTGCTAACATCGCTTCATGGTCAGTGCAAATCGACCCTATCGCTAACGCTAAA

GAATTCGCTAGATCAGTGGGTTTCGAAGCTACTGACGACATCTACGCTCTGGAAGAATTC

TACAAAAAAGCTTCACTGGAACTGCTGACTTCAACTGGTGCTTTCTTCGACAGAACTTAC

GCTTCATTCGTGTTCTCACCTTGCGTGGAACAAGAAACTGTGGACGACGCTTTCCTGACT

GACGCTCCTTACAACATCCTGAAATCAGGTAACTACAGAAAATACCCTCTGCTGTACGGT

TTCGCTAACATGGAAGGTCTGCTGAGAATCGGTCAATTCTCAGCTTGGAAAGACAAAATG

AACGCTAACTTCTCAAACTTCCTGCCTATCGACCTGAAATTCGAATCACTGGAACAAAGA

AACGAAGTGTCAAAAAAAGTGAAAGAATTCTACTTCGGTGACAAACCTGTGGTGAAAAAC

AACGAACTGTCAGAAGACTTCAAAAACATCTCAAACATGATGCTGGACATCTGGATCAAC

TTCATGACTACTGGTTCACCTGTGCCTGAAGGTTCATCACTGCCTGCTTGGCCTCCTGCT

GGTGCTGGTGGTTCACC TCACATGAGACTGGGTAGAACTGTGGAACTGAGAGGTGCTCTG

CTGGCTGACAGAGTGAGATTCTGGGACTCAATCTACGACAGACACTACAGAGTGCCTGCT

CCTCCTCCTCCTCCTCCTGCTCAACACACTGAATTC

**4 >evm-Contig4177_pilon-processed-gene-0.23-mRNA-1**

ATGAGAGTGCTGCTGGCTGCTCTGACTGCTCTGGCTGCTAGAGTGCTGGCTGGTCCTCAC

GAACACAGAGCTAGACACCACGCTCCTGACCACGCTCCTCACTTCCCTGCTCCTCCTCCT

GAACCTTACCACGGTCACGGTGAAGCTGTGAGATACAACCCTGAACTGGACACTATCCTG

CCTAGAATCGAAGAACACGAAACTTCATCAAAAAGAGCTAAATTCGAAGACGCTGAAACT

TCATCAAAAAAAGCTAAATACGACGAATTCTACTCAAACCACGAAAGATCAGACGAATTC

ATGGCTGACGAACCTCAACTGGGTCCTGAAGAAGACGACCCTCTGGTGATCAGAACTAGA

AAAGGTAAAATCAGAGGTATCACTCTGACTGCTGCTACTGGTAAAAAAGTGGACGCTTGG

TTCGGTATCCCTTACGCTCAAAAACCTATCGGTGACCTGAGATTCAGACACCCTAGACCT

GTGGAATCATGGGGTGACGAAATCCTGAACACTACTACTCTGCCTCACTCATGCGTGCAA

ATCGTGGACACTGTGTTCGGTGACTTCCCTGGTTCAATGATGTGGAACCCTAACACTGAC

ATGCAAGAAGACTGCCTGTACATCAACATCGTGTCACCTAGACCTAGACCTCAAAACGCT

GCTGTGATGCTGTGGGTGTTCGGTGGTGGTTTCTACTCAGGTACTGCTACTCTGGACGTG

TACGACCCTAAAATCCTGGTGTCAGAAGAAAAAGTGGTGTACGTGTCAATGCAATACAGA

GTGGCTTCACTGGGTTTCCTGTTCTTCGACACTCCTGACGTGCCTGGTAACGCTGGTCTG

TTCGACCAACTGATGGCTCTGCAATGGGTGAAAGACAACATCGCTTACTTCGGTGGTAAC

CCTCACAACATCACTCTGTTCGGTGAATCAGCTGGTGCTGTGTCAGTGTCACTGCACCTG

CTGTCACCTCTGTCAAGA AACCTGTTCTCACAAGCTATCATGCAATCAGGTGCTGCTACT

GCTCCTTGGGCTATCATCTCAAGAGAAGAATCAATCCTGAGAGGTACTAGACTGGCTGAA

GCTGTGCACTGCCCTCACTCATCAACTGACATGGGTCCTATGATCGAATGCTCAAAAGAA

GAAAGA

**5 >maker-Contig5480_pilon-augustus-gene-0.2-mRNA-1**

ATGAAAGTGTTCCTGTGCGTGGTGTCAGCTCTGGCTGCTGTGCAAGCTCAAGACACTCCT

TCAAGACTGGTGAACATCACTCAAGGTCCTGTGAGAGGTTACAAAGAACCTGGTCTGGAC

GTGTTCTCATTCTACTCAATCCCTTACGCTAAAGCTCCTACTGGTCCTGACAAATACAAA

GAAAAAAACGTGATCGCTGTGACTTTCAACTACAGACTGGGTACTGTGGGTTTCCTGTGC

CTGGGTACTCCTGACATCCCTGGTAACGCTGGTATGAGAGACCAAGTGGCTGCTCTGAAA

TGGGTGCAACAAAACATCGCTTCATTCGGTGGTAACCCTAACGACGTGACTATGCTGGAC

GCTGTGCTGGAAGAAAGACAAAACCTGGCTAAAGACTTCGGTTACTCAGGTCCTGACAAC

ATCGAATCACTGGAAGCTTTCTTCAAAAACGTGTCATACGACACTATGAACGCTCTGTCA

CTGCACGAAAACAAAGACGTGGAAATCAAAATGTCACCTTGCCTGGAAAGAGACATCGGT

ATCGAAAGATTCCTGGAAGACACTCCTATCAACATCATCAAAAAAGGTGACTTCATCAGA

CTGCCTATCCTGTACGGTTGGACTAACATGGAAGGTATCTTCAGACTGTACGTGTTCGAC

ACTTGGAAAAACGAAATGAACGAAAACTTCACTGCTTTCCTGCCTTCAGACCTGAAATTC

GACTCAGAAGAACAAAGACAACAAATCGCTGAAAAAGTGAAACAATACTACTTCGGTGGT

CCTGTGGGTAACAACAACGTGCTGAGATTCATCGACTACATCACTGACGTGACTTTCAAC

GTGGCTATGCAAAGAGCTGTGACTATGCAAGTGGAAGCTGGTAACAACAACATGTACATC

GGTGTGTACTCATTCTCAGACAACCAAACTATGGTGATCCCTCACACTAACGAAAGAGGT

GCTAACCACTGCGACCAATCAGTGGCTATCCTGGACCTGGACGAATCAAACCTGACTTCA

GAATACCTGGAACTGAGAAAAGCTTGGAGAGCTGTGTGGCTGAACTTCATCACTAAAGGT

CCTCCTCTGCCTCCTCCTAACTGGACTACTCCTTTCGAAGCTACTCAAAGACACGTGATC

TGCCACCAATACAACATCTTCGGTCCTGGTCTGACTTTCCAAGAAGACTGCCTGGTGGCT

TCAGTGTTCGTGCCTGACACTAACATCACTAACCTGCCTGTGCTGGTGGTGATCCACGGT

GGTACTTTCCAATCAGAATGGGGTAACATCGAAACTCCTTCACAACTGGTGAACCAAGGT

AAAAACATCATCGCTGTGACTTTCAACTACAGACTGGGTCCTGTGGGTTTCCTGTGCCTG

GGTACTCCTGACGTGCCTGGTAACGCTGGTATGAGAGACCAAGTGGCTGCTCTGAAATGG

GTGAAACAAAACATCGCTGCTTTCGGTGGTAACCCTAACGACATCACTATCGCTGGTTGC

TCAGCTGGTGGTGCTTCAGTGGACCTGCACTTCATCGCTAAAGCTTCAAGAGGTCTGTTC

AACAAAGTGATCCCTCAATCAGGTGGTAACATCGGTGCTTTCGCTGTGCAAGTGGACCCT

ACTCTGAACGCTAGAAACTTCGCTCAAGACATCGGTTACAACGGTACTGACAACCTGGAA

TCACTGGAAGCTTTCTACAAATCAGCTTCATACGAACAACTGTTCTCAATCTCACTGCAC

GACAACAAAGACGTGGAAATCAAAATGGCTCCTTGCCTGGAAAGAGACACTGGTATCGAA

AGATTCCTGGAAGACACTCCTATCAACATCATCAAAAAAGGTGACTTCGTGCAAATGCCT

ATCCTGTACGGTTGGGCTGGTATGGAAGGTATCCTGAGACTGTACCTGTTCGAAACTTGG

AAAGTGCAAATGAACCAAGACTTCTCACAATTCCTGCCTTCAGACCTGAAATTCGAATCA

GCTCAACAAAAACAAGAACTGGCTGAAAAAGTGAAAAAATTCTACTTCGGTGACGCTGTG

TCAAACGAAAACATCCTGAGATACATCGACTTCCACTCAGACGTGATGTTCAACGTGGCT

ATGCAAAGAGCTGTGACTATGCAAGTGGAAGCTGGTAACAACAACATGTACATCGGTGTG

TACTCATTCACTGACAACCAAACTATGGTGATCCCTCACACTAACGAAAGAGGTGCTAAC

CACTGCGACCAAACTGTGGCTATCTTCGACCTGAACGAATCAA CTATCACTCCTGAATAC

CTGGA AACTAGAAGAGCTTGGAGAGCTATCTGGCTGAACTTCATCACTACTGGTGAACCT

ACTCCTGACGGTAACATCCCTCCTCCTTTCCCTAAATGGGAAGCTGCTAGAGCTAACAGA

ACTCCTTGCATGGAAATCGGTAAAACTATCAGAGTGTACCCTGGTCCTTACGACCTGGAA

AGACAACAATTCTGGGACGAAATCTACGACCAACACAAAAAAGACCCTATCGCTCCTTAC

GACTTCAACAACGGTGCTCTGACTCTGTACAACGGTATCATCCTGACTTTCATGAACTTC

GACTCATTCTTCTGGACTCAAAACATGAGAATCATCCTGGGTATCATGGTGGCTCTGGCT

GCTGTGCAAGCTGAAGTGAACATCTCAAGACTGGTGACTATCTCACAAGGTCCTGTGAGA

GGTTACAAAGAACTGGGTCTGGACGTGTTCTCATTCCTGTCAATCCCTTACGCTACTGCT

CCTACTGGTGCTGACAAATTCAAACCTCCTCTGCCTCCTCCTAACTGGACTATCCCTTTC

GAAGCTACTAGAAGAAACGTGCTGTGCAACCAATTCAACTTCATGGGTTCAAACTTCCAA

ACTCAAGAAGACTGCCTGGTGGCTTCAGTGTTCGTGCCTGACACTAACAAAACTAACCTG

CCTGTGCTGATCGTGATCCACGGTGGTGCTTTCCAAAACGGTTGGGGTAACTCAAGAACT

CCTTCACAACTGGTGAACCAAGGTAAAAACATCATCGCTGTGACTTTCAACTACAGACTG

GGTCCTGTGGGTTTCCTGTGCCTGGGTACTCCTGACATCCCTGGTAACGCTGGTCTGAGA

GACCAACTGGCTGCTCTGAAATGGGTGAAACAAAACATCGCTGCTTTCGGTGGTAACCAA

AACGACATCACTCTGGCTGGTTGCTCAGCTGGTGGTGCTTCAGTGGACCTGCACCTGCTG

GCTAAAGCTTCAAGAGGTCTGTTCAACAAAGTGATCCCTCAATCATCATGCAACATCGGT

GCTTACTCAGTGCAAGTGGACCCTATCCTGAACGCTAGAAACATCGCTAAAGACCTGGGT

TACAAAGGTACTGACAACCTGGACAACCTGGAAGCTTTCTACAAAAACGCTTCATACGAA

CAACTGTTCTCAATCTCACTGGACGACAACAGAGACGTGGAAATCAAAATGGCTCCTTGC

CTGGAAAGAGACACTGGTATCGAAAGATTCCTGGAAGACACTCCTAGAAACATCCTGAAA

AAAAAAGACTTCGTGAGAATGCCTACTCTGTACGGTTGGGCTGGTATGGAAGGTATCCTG

AGACTGTCAAGATTCCAATCATGGAAAGTGCTGATGAACCAAGACTTCTCACAATTCCTG

CCTTCAGACCTGAAATTCGACTCAGCTCAACAAAAACAAGAACTGGCTGAAAAAGTGAAA

GAATTCTACTTCGGTGGTGCTGTGTCATCAGAAAACATCCTGAGATTCATCGACTTCAAC

TCAGACGTGATGTTCAACGTGGCTATCCAAAGAGCTGTGACTATGCAAGTGGAAGCTGGT

AACAACAACGTGTACATCGGTGTGTACTCATTCACTGACAACCAAACTATGGTGATCCCT

CACACTAACGAAAGAGGTGCTAACCACTGCGACCAAACTCTGGCTATCTTCGACCTGGAC

GAATCAACTCTGACTCCTGAATTCATCGAAACTAGAAGAGCTTGGAGAGCTATCTGGCTG

AACTTCATCACTACTGGTGAACCTACTCCTGAAGGTAACATCCCTCCTCCTTTCCCTAAA

TGGGAAGCTGCTAGAGCTAACAGAACTCCTTGCATGGAAATCGGTAAAACTATCAGAGTG

TACCCTGGTCCTTACGACCTGGAAAGACAACTGTTCTGGGACAAAATCTACGACCAACAC

AAAAAAGACCCTATGCCTCCTTACGACTTCAACAACGGTGCTCTGATCCTGCACAACGTG

GCTGCTCTGAAATGGGTGCAACAAAACATCGTGGCTTTCGGTGGTAACCCTAACGACGTG

ACTATCGCTGGTTGCTCAGCTGGTGGTGTGTCAGTGGACCTGCACATCCTGTCAAAACCT

TCAAGAGGTTTCTTCCACAAAGTGATCGGTCAATCAGACTCACTGGAAGCTCTGGAAGAA

TTCCACAAAACTGTGTCATACGACAAACTGCTGTCACTGCCTCTGTACGAATCAAAAGAC

GTGGAAATCCTGATGTCACCTTGCCTGGAAAGATACATCGGTATCGAAAGAATCCTGGAA

GAACCTAGACTGCAAACTATGAAAAGAAGAGACTTCCCTAAATACCCTATCCTGTACGGT

TGGGCTGCTATGGAAGGTCTGCTGAGACTGTACGCTTTCGAAAACTGGAAAAACGAAATG

AACGAAGACTTCGCTAAATTCCTGCCTTCAGACCTGATCTTCTACAACGACAACCACAAA

AAACAAGTGCCTGACAAAGTGAAAAACTTCTACTTCGGTAACTCAGTGGCTAACGAAAAC

ATCCTGAGATACATCGACTACAACTCAGACGTGATGTTCAACGTGGCTATGCAAAGAGCT

GTGACTATGCAAGTGGAAGCTGGTAACAACGCTATCGACCCTACTCCTAACGGTAACATC

CCTGCTCCTTTCCCTAAATGGGAACCTGCTAGAGCTAACAGAATCCCTTGCAAAGAAATC

GGTAACACTATCAGAATCTACCCTGGTCCTTACGACCTGGAAAGACAACAATTCTGGGAC

GAAATCTACGACCTGTACAAAAGAGACCCTATCGCTCCTTACGACTTCAACAACGCTATC

ATGCTGTACAACGGTATCCTGCTGAAAATGAAAGGTTTCTTCTACGTGATCGTGGCTCTG

GCTGCTGTGCAAACTGCTGAAAACCACTCAAAACTGGTGAACATCGACCAAGGTCCTGTG

AAAGGTTACAAAGTGCCTGGTACTGACGTGTTCGCTTTCTACTCAATCCCTTTCGCTAAA

GTGCCTACTGGTCCTGACAGATTCAAAATGCACCAACACGAATTCGGTCCTCCTCTGCCT

CCTCCTGTGTGGACTGAACCTTTCGAAGCTGTGAACAAAGACGTGCTGTGCCTGCAATAC

GACACTTTCCCTCCTGGTAAATACGAATTCGTGGAAGACTGCCCTTTCGGTTTCCTGTGC

CTGGGTACTCCTGACATCCCTGGTAACGCTGGTATGAGAGACCAACTGGCTGCTCTGAAA

TGGGTGCAACAAAACGTGGCTGCTTTCGGTGGTAACCCTAACGAAGTGACTATCGCTGGT

TGCTCAGCTGGTGGTGCTTCAGTGGACCTGCACATCCTGGCTAAAGCTTCAAACGGTCTG

TTCAACAAAGTGATCACTCAATCAGGTGCTAACATCGGTGCTTTCGCTGTGCAAACTGAC

CCTACTCTGAACGCTAGAAACTACGCTAAACAAAGAGACATCGGTATCGAAAGATTCCTG

GAAGACACTCCTATCAACATCATCAGAAAAGGTGAATTCACTAGATACCCTATCCTGTAC

GGTTGGGCTGACATGGAAGGTATCCTGAGAGTGTACTCATTCGACTCATGGAAAAACGAA

ATGAACGAAGAATTCACTAAATTCCTGCCTTCAGACCTGAAATTCGAATCAGAAGAACAA

AAACAACAAATCGCTGACAAAGTGAAACAATTCTACTTCGACGGTGACGTGACTAACGAC

AGAATCCTGAACTACATCGACTACTCATCAGACGTGATGTTCAACGTGGCTATGCAAAGA

GCTGTGACTATGCAAGTGGAAGCTGGTAACAACGCTATGTACGTGGGTGTGTACTCATTC

ACTGACAAAAACACTTCAATCATCCCTTACACTAACGAAAGAGGTGCTACTCACTGCGAC

CAAACTGTGGCTATCCTGGACCTGGACGAATCAACTCTGTCACCTGAATACCTGGAACTG

AGAAGAGCTTGGAGAGCTGTGTGGCTGAACTTCATCTCAACTGGTGACCCTACTCCTGAC

GGTAACATCCCTCCTCCTTTCCCTAAATGGGAAGCTGCTAGAGCTAACAGAACTCCTTGC

ATGGAAATCGGTAAAACTATCAGAGTGTACCCTGGTCCTTACGAACCTGAAAGACAACAA

CTGTGGGACGAAATCTACGACCAACACAAAAGACACCCTATCGCTCCTTACTACTTCAAC

GAAGAAGAATCAGTGGAC

**6 >evm-Contig11205_pilon-processed-gene-0.1-mRNA-1**

ATGAGATGGGTGAACAAAAACATCGCTAACTTCGGTGGTGACCCTAACAACGTGACTATC

TTCGGTCAATCAGCTGGTGCTGCTTGCGTGACTTTCCACTGCGTGTCACCTATGACTAAA

GGTCTGTTCAAAAGAGCTATCGCTCAATCAGGTTGCTTCGCTAACTGGTGGGCTCAAACT

AACAGACCTAGAGAAAGATCAGAAGCTCTGGCTAGATCACTGGGTTGCCACTCAAAAGAC

GACAAAGAACTGTACGAATTCTTCAAAAACCAACCTAAAGAAAACCTGGTGGAAGCTCAC

CTGCCTATCACTATCAAAGAAAACGACTGCGACAAATACGAAACTAAAAACTCAGTGGTG

TCAGAAAAACTGTTCCCT AACGTGGAACCTTACTTCACTGGTAACATCTTCGACGCTCTG

AAAACTGACATCCACGAAGGTGTGGAACTGATGATCGGTTACAACGAAGACGAAGGTGTG

ATCAACCTGGGTGTGTCACTGATCCTGAAAAGACAATACTTCAAACTGACTCTGTCATGG

AACATCCTGTTCCAAGACTTCTCATGCACTACTGTGGTGCTGACTATCAACTGGATC

**7 >augustus_masked-Contig4653_pilon-processed-gene-0.41-mRNA-1**

ATGG TGCAAGTGACTGTGAACGAAGGTATCCTGGAAGGTGAACTGGTGAACTCAAAATAC

GGTGAACCTTTCTACTCATTCAAAGGTATCCCTTACGCTGAACCTCCTGTGGGTGACCTG

AGATTCATGGTGGAAGTGCTGGGTTTCCTGTGCCTGGGTACTAAAGACATCCCTGGTAAC

GCT GGTATGAAAGACCAAGTGGCTGCTCTGAGATGGATCAACAAAAACATCTCACACTTC

GGTGGTGACCAAAACAACGTGACTATCTTCGGTTGCTCAGCTGGTTCAATGTCAGTGACT

TACCACCTGGTGTCACCTATGACTAAAGGTCTGTTCAAAAGAGCTATCGCTCAATCAGGT

ACTTCAACTTGCTGCATCGGTATCATGACTCACCCTAGAGAAAGAGCTCTGGCTCTGGCT

AAAAAACTGGGTCTGAACTCAGAAGACGACAAAAAATTCATGTCATTCTCAAACCTGTAC

CACCTGATCGACCTGCTGGAACTGAAAAGACCTGAAGAAATCTTCGACGACACTGAAACT

TTCTTCCACGGTGACCCTTGCACTGTGCTGAGAAACGGTATCCACGAAGGTGTGGAAGTG

ATCACTGGTTACACTTCAGACGAAGGTGTGCTGGCTATCGGTGCTGCTCCTAACATCGAC

GAAATCTTCAGACTGATCAACACTTTCAAAGACTACGTGGTGCCTAAACCTATCGCTGAA

AACTGCACTTCAAAAATCCAACTGGAAGCTGGTAGAAAAATCAACAAATTCTACTTCGAC

AACAAAAACGTGTCAATGGACTGCATCGACGACCTGATCAAATTCTACTCAATGTACCTG

TTCACTTTCGGTACTTACCAATGGGCTAAAATCTGCTCAGGTCTGAACAGAAACAAAGTG

TACCTGTACAAATTCACTTGCAAAACTGAAAGAAACATCTTCGCTAAAGTGCTGGGTGCT

CAAAAAGTGATCGGTTCAAAAAAAGTGGTGTCACACGCTGACGACGTGACTTACATCTTC

CCTATGAAAGACATGTCAAAACCTATCGACAAAGAATCAGAAACTTTCAAAATCATCGAC

AGAATCACTACTCTGTGGACTAACATGGCTAAATACGGTGCTAAAATGCAAGTGAAAATC

ACTGACGGTATCCTGGAAGGTGAAACTGTGAACAACGAAATCGGTGGTACTTTCTACTCA

TTCAAAGGTATCCCTTACGCTGCTCCTCCTCTGGGTAACCTGAGATTCAAAGCTCCTCAA

CCTCCTCTGCCTTGGGAAGGTGTGAGATCAGCTAAACAACACGGTGACTTCTCATACCAA

TTCAACTTCATGGTGAGAGCTATCGAATCAGGTTCAGAAGACTGCCTGTACCTGAACGTG

TACACTCCTAACACTAAACCTTCACAACTGATGCCTGTGATGATCTGGATCCACGGTGGT

GCTTTCTGCTGCGGTTCAGGTAACGACGACGTGTACGGTCCTGAATACCTGATCAGAAAC

GACGACAAAGAACTGTACGAATTCTTCAAAAACCAACCTAAAGAAAACCTGGTGGAAGCT

CACCTGCCTATCACTATCAAAGAAAACGACTGCGACAAATACGAAACTAAAAACTCAGTG

GTGTCAGAAAAACTGTTCCCTAACGTGGAACCTTACTTCACTGGTAACATCTTCGACGCT

CTGAAAACTGACATCCACGAAGGTGTGGAACTGATGATCGGTTACAACGAAGACGAAGGT

GTGATCAACCTGGGTGTGTCATTCGACATCAAAAAAACTATCTTCCAAGCTAACACTTTC

GTGGAATACTTCGTGCCTAGATTCCTGGTGTACTACTGCTCAACTGACAACCAACTGGAC

ATCGGTAAAGCTATGAAAGAATTCTACCTGAAAAACGAAATCCTGTCAGAAAACAACCTG

GAACCTCTGGCTAAATACTTCGCTGCTGACATGTACAAATTCGGTCTGTACACTCTGGCT

AAATACTTCTCAGTGAAAAACAAAGTGTACTTCTACAAATTCACTTGCAAAACTGAAAGA

AACTTCTTCTCAACTATCCTGGGTGTGTCAAAATACTTCGGTAACAGACCTCTGGTGGGT

CACATCGACGAAGTGCCTTACCTGTTCCCTGTGAAATCAATCTCACAAAGAGTGTCATCA

GAAACTCAAAAATCAATCAACACTGTGTCAAAACTGTGGACTAACTTCGCTAAAAAAGGT

ACTCAAGACCTGCTGGGTATCATCATCCACCCTACTCCTGACGCTACTCTGGGTGTGAAC

TGGAGACAATTCAGAGCTGACATGCTGCTGACTTGGATCTCAGGTATCCTGCTGTCAGAA

GAATCAGACCCTACTAGAAAATCATCAATCCTGGGTGAAAACCTGAGACAAATCCCTACT

GCTTACGGTCTGCCT

**8 >augustus_masked-Contig132_pilon-processed-gene-0.69-mRNA-1**

ATGCACCTGTTCATCCTGCTGTCAGTGCTGGCTCTGGCTCCTCTGCCTGCTAAAGCTTGG

AAAGGTACTAGAAACGCTACTGAACACGGTCCTATCTGCATCCAATACGACGTGGTGACT

AAACACATCCTGCCTGGTTCAGAAGACTGCCTGTTCCTGAACGTGTACACTCCTAAACTG

AAACCTACTAAACCTCTGCCTGTGCTGTTCTTCATCCACGGTGGTGGTTGGAAATCAGGT

TCAGGTAACGACGACGAATACGGTCCTGACTTCCTGGTGCAATACGACATCGTGCTGATC

ACTATCAACTACAGACTGGACGTGTTCGGTTTCCTGACTATGGACAACGAAGACGTGCCT

GGTAACGCTGGTATGAAAGACCAAGTGCTGGCTCTGAAATGGGTGAACAACAACATCAAA

TACTTCGGTGGTGACCCTGAAAACATCACTATCATGGGTCAATCAGCTGGTTCAGCTTCA

GTGATCTACCACATGATGTCACCTATGTCAAAAGGTCTGTACAAAAGAGCTATCGCTATG

TCAGGTGTGCCTCTGTCAGAATGGGCTCAACCTTTCGAAGCTCCTAGAAGATCATTCTCA

CTGGGTACTATCCTGGGTAACCCTACTACTGACCCTAAAGAACTGATCAAATTCCTGAGA

AACGTGCCTGCTGAAAACCTGCTGAACACTAAACCTGCTGTGATCTCACAAGAAGAACTG

TGGATCAACCCTATCAAAATGTACGCTCTGGTGCCTGTGGTGGAAAGAGACTTCGGTCAA

GAAAGATTCCTGGTGGAAACTCCTACTGAATCAATCATCAACGGTAACGTGAGAGACCAA

GAACTGATCATCGGTCTGACTTCAATGGAATTCATCTACGCTATCCCTTGGCTGGAAGAC

GACCACGTGGCTAGATTCAACCTGGCTCTGGACCTGCTGGTGCCTCTGGACCTGCTGAGA

GAAATCACTCCTAAAGAAGTGGGTCACTACAGAACTCCTCTGCCTAGAAAACCTTGGAAC

AAAACTAGATCAGCTAGAGAACACGGTCCTGTGTGCATCCAATACGACATCTTCACTGAC

AGATACCTGCCTGGTTCAGAAGACTGCCTGTTCCTGAACGTGTACACTCCTTGGATCAAC

CCTAAATCACCTCTGCCTGTGCTGTTCTTCATCCACGGTGGTGCTTGGAAATCAGGTTCA

GGTAACGACGACAACTACGGTCCTGACTTCCTGATGCACTACGACATCATCCTGGTGACT

ATCAACTACAGACTGGACGTGCTGGGTTTCCTGTCACTGGACACTAAAGACGTGCCTGGT

AACGCTGGTATGAAAGACCAAGTGCTGGCTCTGAAATGGGTGCACGACAACATCGGTAAC

TTCGGTGGTGACCCTAACCAAGTGACTATCATGGGTCAATCAGCTGGTGGTGCTTCAGTG

ATCTACCACATGATGTCAACTGTGTCAAGAGGTCTGTTCAAAAGAGCTATCTCAATGTCA

GGTGTGCCTCTGTCAGACTGGGCTTTCCCTTTCGAACAAACTACTAGAGCTTACGAACTG

GCTAAAACTATGGGTCAAAACATCGACAACTCAGAAGACCTGCTGAACTACCTGCAAAAC

GTGCCTGCTCACGACCTGCTGAACACTAAACCTGCTGTGCTGTCATCAGAAGTGTACTGG

AACAACGGTATCAAAATGTTCTCATTCGTGCCTGTGATCGAAAAAGACTTCGGTAAAGAA

CAATTCCTGGTGGAACCTATCAAAGAAGCTCTGGTGAACGGTAACATCGAAGACACTGAC

CTGCTGATCGGTTACACTGACCTGGAATACCTGATCGCTGTGCCTCTGCTGGAATCATAC

GGTCTGGCTGACTACAACATGTTCAAAGAACTGCTGGTGCCTAGAGAAATCCTGCTGGAA

ATCTCACCTAAAAGAATGCTGTCACTGGCTGAAAAAATCCACAAAGCTTACTTCGGTAAC

CAATTCATCAACAACACTACTATGCTGAACTTCCAAAACTACGGTGGTGACCACATCTTC

ATCAACTCAGTGCTGAGATTCCTGAGATACCTGCCTAAAGGTAGAGCTAAAAGATACCTG

TACAAATTCTTCACTGAATCATCAAGAAACAGATACCTGAAAAACGCTGAAAAATACAAC

ATCCACGGTTGCGCTCACACTGACGACCTGATGTACCTGTTCGACGCTAAACAAGAAGCT

ACTGCTATGAACGTGGACTCAGACGAATACCAACTGATCAACCAAACTTGCACTCTGTTC

ACTAACTTCGTGAAATACGGTAACCCTACTCCTGACGACGCTCTGGGTGTGACTTGGCCT

GAATACAACAACGACGACAGACCTTACCTGTCAATCAACAACAAACTGACTACTGGTACT

CACCTGGACTTCGACGAACCTGTGCCTAGAAAACCTTGGAAAGGTATCAGAGACGCTACT

CAACACGGTCCTGTGTGCTCACAATACGACTTCTTCGCTCAAATGTACCACCCTGGTTCA

GAAGACTGCCTGTTCCTGAACGTGTACACTCCTAACCTGGCTCCTAAAACTCTGCTGCCT

GTGATCTTCTTCATCTACGGTGGTGGTTTCAAATCAGGTTCAGGTAACGAAGACGAATAC

GGTGCTGACTTCCTGGTGCAACACAACGTGATCGTGGTGACTGTGAACTACAGAATGGAC

GCTCTGGGTTTCCTGTGCCTGAACATCAAAGAAGTGCCTGGTAACGCTGGTATGAAAGAC

CAATCACTGGCTCTGAAATGGGTGCACGACAACATCCAATACTTCGGTGGTAACCCTTAC

GAAGTGACTATCATGGGTCAATCATCAGGTGCTGCTTCAGTGATCTACCACATGATGTCA

CCTATGTCAAGAGGTCTGTTCAAAAGAGCTATCTCAATGTCAGGTGTGCCTTTCTGCGAC

TGGGTGATCCCTCTGGAACCTCAAAAAAGAGCTTTCGAACTGGGTAAAGCTCTGGGTAAA

AACACTAAAAACCCTTACGAACTGCTGGACTTCCTGCAATCAGTGCCTACTGAACAACTG

GTGAACACTGCTCCTCTGGTGATCTCACAAGAAATCATCTGGAACTTCCCTTTCAGAATG

TTCCC TTTCGTGCCTGTGGTGGAAAACGACTACGGTCAACAAAGATTCCTGGTGGAATCA

ATCAAAGACGGTCTGACTATGGGTAACATCGCTGACGTGGACCTGCTGATCGGTTACACT

TCAAACGAAAACCTGATGGCTATCCCTTTCCTGGAATCATACTACCACACTTACGACACT

TACTCAGAACTGTTCGTGCCTAGAGAAGTGGCTCTGGAAATCTCACCTAAAGAAGTGCTG

AAAGAACCTCTGGTG

**9 >maker-Contig3761_pilon-augustus-gene-0.1-mRNA-1**

ATGATGGTGCACCTGCCTATCAGAACTAACTTCAGATTCGACAGAAACGACTCATCATTC

GAAGACTACAAAGAAGAAATCCTGAAATTCTACCTGAAAGGTAAACAACTGAAATACTAC

AACATCCTGGACTACGCTGTGTACGTGGCTGACATCGTGGAAAACTACTCACTGAACATC

GCTGCTAGAAAACTGTCAAGAGAACTGGAATCACCTACTTACTACTACATGTTCGACTTC

AACGGTCTGCTGAACGAAAACATGATCTACCTGTCAAAAAACACTAGAGGTGGTACTAAC

AACTGGGGTGCTTCAGTGGGTGACGAAATCTGCTACCTGCACCTGTGCTCAAGAATCAGA

GACAACTACCTGGAAATCAAATCACTGGTGTCACAACAAAACGAATTCAAACTGGTGAGA

AAAATGATCAGACTGTGGGCTAACTTCGCTAAAACTGGTAACCCTACTCCTCAAGCTGAA

GACGACGTGCTGAAAAACTTCATCTGGAAACCTATCGACAAAAAATCAGAAAACCTGCCT

TACGCTCACATCACTAAAAGAATCAAAATGCTGGAAAACCCTCTGGAAAAAAGAGAAATC

TTCTGGAACAACCTGCTGGACAAATACTCAAAACTGGCTGTGAACGGTGTGGTGAGAAAA

ATCGAA GCTCACGACGAACTG

**10 >maker-Contig3761_pilon-augustus-gene-0.2-mRNA-1**

ATGTACAGAAACATCATCATCGTGCTGTTCACTTTCCTGGCTCTGAAACTGACTTGCATC

AACGCTAGAGGTCCTATCGTGGAAACTACTCACGGTAAAGTGGAAGGTAAAGTGGTGAAA

ACTCTGCTGAAAAACGTGAAATACCACGCTTACATGGGTATCCCTTACGCTGCTCCTCCT

ATCAA AGACCTGAGATTCCTGCTGCTGAAAGAACTGAACGACCTGCCTGCTTCAGACCTG

CTGTCAAAAGAACTGAGAGCTGCTCCTGACGACTACTTCAAAGAAGAACAAAGAGGTACT

ATCTCATTCGCTCCTATCGTGGAAAAAGACCCTAACGGTCTGATCACTAAATACCCTGAA

GACTCAGAAGACGGTATCAACATCCCTATCATGATCGGTTTCAACTCAAGAGAAGGTATC

GACGCTTCAATCCACTACCTGGAAGAACCTAGATTCCTGTCATTCGTGCAAAAAGACTTC

CCTTTCTTCATCCCTATCAGAGCTAAATTCAAATTCAACCCTAACGACGAAATCTGCTAC

GAAGCTATCAACGAAATCAAAGACCTGAACCCTACTCCTGACGGTGACGCTGCTTCAAAA

GAACTGAAATGGCCTACTTACAACTCAGAATCAAAAAACTACCTGCACATCACTAAAACT

ATCGAACACTTCAAAATCGCTTACAACGCTTCAAAAGAATACGGTCCTGACTTCTTCATG

AAAGACGTGATCGTGGTGACTCTGCAACACAGACTGGGTTGCCTGGGTTTCCTGTCATTC

GAAGACGACCTGCTGCCTGGTAACAACGGTCTGAGAGACGTGCTGCTGGCTCTGAAATGG

ATCCAAACTAACATCGGTTCACTGGAAGTGATCCCTACTTCAAACTCAAACGGTAACGAC

GGTGGTGCTGTGATCGTGGACCTGCTGCTGCACTCACCTAAAGCTAAAGGTCTGTTCCAC

AGAGCTATCCTGCAATCAGGTTCATCATGGTACCCTGTGTACCTGGGTGACAAACCTAGA

GAAAGAGCTATCGCTTTCTCAAAAGAACTGGACGAACACTCAAAAGCTAACTCATCAAGA

GACAAAGTGGAATGGAAACCTGCTACTAAAGAAAACAAAGAATGCCTGGTGATCTCAGAC

GACCTGTCACTGAAAACTAGACTGCACGACGAAAGAATCTCATACTGGGACACTTTCCTG

GAAACTTACGGTAAACTGGCTGTGGACGGTGTGATCAAAGACATCAAAGACGAACTG

**11 >maker-Contig9545_pilon-augustus-gene-0.25-mRNA-1**

ATGCCTATGGTGTTCCACCTGATGCTGTACTCAGAAAACATCAAAATCATCAACGCTCCT

TCAATCGGTGACGTGGACGAATTCTCATTCTCAATCTCAAAAAACCTGTACATCTTCTTC

CAAAGAACTTCAACTTCACTGTGGGGTGAATGGATGGGTGTGATGCACGGTGACGAAATG

GAATACGTGTTCGGTCACCCTCTGAACATGTCACTGCAATACCACACTAGAGAAAGAGAC

CTGGCTGCTCACATCATGCAATCATTCACTAGATTCGCTCTGACTGGTAAACCTCACAAA

CCTGACGAAAAA TGGCCTCTGTACTCAAGAGCTTCACCTCACTACTACACTTACACTGCT

GACGGTCCTTCAGGTCCTGCTGGTCCTAGAGGTCCTAGAGCTTCAGCTTGCGCTTTCTGG

AACGACTTCCTGAACAAACTGAACGAACTGGAACACGTGCCTTGCGACAGAGCTGTGACT

GGTCCTTACTCATCAGTGGCTGGTACTACTCTGCCTGTGCTGCTGCTGACTGCTCTGGCT

ACTACTATCGCTCTG

**12 >maker-Contig9545_pilon-augustus-gene-0.26-mRNA-1**

ATGGGTAGAGAAGTGCACATCTTCACTGGTATCCCTTTCGCTAAACCTCCTCTGGGTCCT

CTGAGATTCAGAAAACCTGTGCCTATCGACCCTTGGCACGGTGTGCTGGAAGCTACTTCA

ATGCCTAACTCATGCTACCAAGAATCAGAAGAAGCTCCTGGTAACATGGGTCTGTGGGAC

CAACAACTGGCTATCAGATGGATCAAAGACAACGCTAGAGCTTTCGGTGGTGACCCTGAA

CTGATCACTCTGTTCGGTGAATCAGCTGGTGGTGGTGTGTCACTGCTGCTGGCTGACCCT

TCACTGGTGATGGACTGCATGAGAGGTGTGGACGCTAAAACTATCTCAGTGCAACAATGG

AACTCATACACTGGTATCCTGGGTTTCCCTTCAGCTCCTACTGTGGACGGTGTGTTCCTG

CCTAAAGACCCTGACACTATGATGAAAGAAGGTAACTTCCACAACGCTGAAGTGCTGCTG

GGTTCAAACCAAGACGAAGGTACTTACTTCCTGCTGTACGACTTCCTGGACTACTTCGAA

AAAGACGGTCCTTCATTCCTGCAAAGAGAAAAATTCCTGGAAATCGTGGACACTATCTTC

AAAGACTTCTCAAAAATCAAAAAAGAAGCTATCGTGTTCCAATACACTGACTGGGAAGAA

ATCACTGACGGTTACCTGAACCAAAAAATGATCGCTGACGTGGTGGGTGACTACT TCTTC

GTGTGCCCTACTAACTACTTCGCTGAAATCCTGGCTGACTCAGGTGTGGACGTGTACTAC

TACTACTTCACTCACGTGTCAACT

**13 >augustus_masked-Contig11588_pilon-processed-gene-0.37-mRNA-1**

ATGCAAGTGAAAACTACTGAAGGTCTGCTGGAAGGTGAAATCGTGAACAACGACATCCTG

GGTACTTACTACTCATTCAAAGGTATCCCTTACGCTGCTCCTCCTCTGGGTGACCTGAGA

TTCAAAGCTCCTCAACCTCCTAAACCTTGGGAAGGTGTGAGATCAGCTAAAGAACACGGT

TCATCATCATACCAATTCAACTTCCTGACTAAAACTAGAGAAATCGGTTCAGAAGACTGC

CTGTACCTGAACGTGTACACTCCTAACACTAAACCTTCAGAACCTCTGCCTGTGATGATC

TGGATCCACGGTGGTGCTTTCTGCTGCGGTTCAGGTAACGACGACATCTACGGTCCTGAA

TTCCTGATCAAACACGACGTGGTGCTGGTGACTATCAACTACAGACTGGAAATCCTGGGT

TTCCTGTGCCTGGAAACTGAAGACGTGCCTGGTAACGCTGGTATCAAAGACCAAGTGGCT

GCTATGAGATGGGTGAACAAAAACATCGCTAACTTCGGTGGTGACCCTAACAACGTGACT

ATCTTCGGTCAATCAGCTG GTGCTGCTTGCGTGACTTACCACTGCATCTCACCTACTACT

AAAGGTCTGTTCAAAAGAGCTATCGCTCAATCAGGTTCAATCATCAACTGGTGGACTCAA

GGTTTCAGACCTAGAGACAGAGCTGAAGCTCTGGCTAGAAAACTGGGTTGCAACTCAAAA

GACGACAAAGAACTGTACGAATTCTTCAAATCACAACCTGTGGAAAACCTGGCTGAAATC

CAAGTGCCTCTGACTTACAAAGAATACCACTGCGACAAATACGAAACTCAATTCTCACTG

GTGGTGGAAAAAGAATTCCCTAACGTGGAATCATACTTCACTGGTAACATCATGGAAGCT

CTGAAAACTGGTATCCACGAAGGTGTGGAACTGATCATCGGTTACAACGGTGACGAAGGT

ATCATCAACATCATGGACGTGGCTAAATACCTGGGTAACAGACAAGTGACTGGTCACATC

GACGAAATCCCTTACCTGTTCCCTGTGAAATCAATCCACCAAAAAGTGGGTTCAGAAACT

CAAAAAAACCCTACTCCTGACGCTACTCTGGGTGTGAACTGGAAACAATTCAGAGCTGAC

ATGCCTTCATTCCTGGAAATCGGTAACACTCTGGTGAGAGGTGTGGCTCCTGACAAAGAA

GAACTGACTTTCTGGGAAGGTATCTTCGAAAAATACCTGCCTTCAATGGTGTTCCACAAA

GAAACTTCA

**14 >augustus_masked-Contig5480_pilon-processed-gene-0.52-mRNA-1**

ATGGGTAGAAACGGTAGATACCTGAGACTGGAACACTTCGACAACTGGAAAATCGAAATG

AACGAAAACTTCAAAAACTTCCTGCCTTCAGACCTGGAATTCGAATCAGAAGAAGTGAAA

CAAAAAGTGGCTGACAGAGTGAAAGAATACTACTTCGGTGACGCTGTGACTAACGACAAA

GTGCTGAACTACATCGACTACCACTCAGACGTGATGTTCAACGTGGCTATGCAAAGAGCT

ATCACTATGCAAGTGGAAGCTGGTAACGACGCTATGTACGTGGGTGTGTACACTTACACT

GACAACAACACTATGGTGATCCCTTACGTGAACGCTAGAGGTGCTAACCACTGCGACCAA

ACTGCTGCTATCCTGGACCAATCAGACGACTCACACCTGACTGAAGAATTCATCGAACTG

AGAAAAGCTTGGAGAGAAATCTGGGTGAACTTCATCACTACTGGTTCAGACATCATCGGT

GCTCTGGCTGCTGTGCAAGCTCAAGGTAACTCATCAAAACTGGTGAACATCGC TCAAGGT

CCTGTGAGAGGTTACAAAATGCCTGGTCTGGACGTGTTCGCTTTCTACTCAATCCCTTAC

GCTAAAGCTCCTTCAGGTAGAGACAAATACAAATCACTGTCACTGCCTCCTCCTCTGCCT

GCTCCTACTTGGACTACTCCTTTCGAAGCTACTGAAAAAAACGTGCTGTGCCACCAATTC

AACATCTTCCCTCCTGGTATGTTCGAATACGTGGAAGACTGCCTGGTGGTGTCAGTGTAC

ACTCCTGACACTAACGCTACTAACCTGCCTGTGGTGGTGGTGATCCACGGTGGTGGTTTC

CAATCAGACTTCGGTAACTACGAATCACCTTCACAACTGGTGGACCAAGGTAAAAACATC

ATCGGTGTGACTCTGAACTACAGACTGGGTCCTATCGGTTTCCTGTGCCTGGGTACTCCT

GACGTGCCTGGTAACGCTGGTATGAGAGACCAACTGGCTGCTCTGAAATGGGTGCAACAA

AACATCGCTGCTTTCGGTGGTAACCCTAACGACGTGACTATCGCTGGTTGCTCAGCTGGT

GGTGCTTCAGTGGACCTGCACATGCTGTCAAAAGCTTCAAGAGGTCTGTTCCACAAAATC

ATCGGTCAATCAGGTGGTAACATCGGTGCTTTCGCTGTGCAAGTGGACCCTACTGCTTCA

GCTAGAAAACACGCTAGAGACATCGGTTACACTGGTCCTGACACTCTGGAAGGTGTGGAA

GAATTCTACAAATCAATCCCTTACGAACAACTGCTGTCAAAAGACCTGCACGAAAACAAA

GACGTGTCAATCGTGATGGCTCCTTGCAAAGGTGACTACATCAGATACCCTGTGCTGTAC

GGTTGGGCTGCTATGGAAGGTATCTTCAGACTGGAATACTTCGAAAACTGGAAAAAAGAA

ATGAACGAAGACTTCACTAACTTCATGCCTACTGACCTGCAATTCAACAACGAAGAACAC

AAAAGACAAGTGTCAGACAAAGTGAAACAATTCTACTTCGGTGGTAACGTGACTAACGAC

AACATCATCAGATTCGTGGACTACAACTCAGACGTGATGTTCAACGTGGCTATGCAAAGA

GCTGTGACTATGCAAGTGGAAAACGGTCACAACGCTATCAACCCTACTCCTGACGGTAAC

ATCCCTCCTCTGTTCCCTAAATGGGAAGCTGCTTCAGCTAACAGAACTCCTTGCATGGAC

ATCGGTAACCCTATCAGAATCTACCCTGGTCCTTTCGACCTGGAAAGACAAAAATTCTGG

GACGAAATCTACGACCAATACAAAAAAGAACCTGTGCCTCCTTACGACTTCAACGACCAA

TGC

**15 >maker-Contig5480_pilon-augustus-gene-0.3-mRNA-1**

ATGAAACTGCTGCTGTCAGTGGCTCTGGCTTTCGTGGTGGTGCAAGCTCAAAACGACGCT

TCAAGACTGGTGAACATCAAACAAGGTCCTGTGAAAGGTTACAAAATGCCTGGTCTGGAC

GTGTTCGCTTTCTACTCAATCCCTTTCGCTAAAGTGCCTACTGGTGCTGACAAATACA AA

ACTAACCTGCCTGTGTTCGTGTACATCCACGGTGGTTCATTCGCTTCAGAATACGGTAAC

ATCGAACCTCCTGGTCAACTGGTGAACCAAGGTAAAAACTTCGTGGGTGTGACTTTCAAC

TACAGACTGGGTCCTATCGGTTTCCTGTGCCTGGGTACTCCTGACGTGCCTGGTAACGCT

GGTATGAGAGACCAAGTGACTGCTCTGAAATGGATCCAACAAAACATCGCTGCTTTCGGT

GGTAACCCTAACGACGTGACTATCGCTGGTTGCTCAGCTGGTGGTGCTTCAGTGGACCTG

CACATCCTGGCTAAAGCTTCAAAAGGTCTGTTCAAAAGAGTGATCGGTCAATCAGGTGCT

AACATCGGTGCTTTCGCTGTGCAAGCTGACCCTACTAGAAACGCTAGAGTGTACGCTAAA

TCACTGGGTTACAACGGTACTGACTCACTGGAAGCTATCAGAATCCTGCAAAACTCATTC

ATC

**16 >maker-Contig11205_pilon-augustus-gene-0.4-mRNA-1**

ATGCAAGTGAAAACTACTGAAGGTCTGCTGGAAGGTGAAATCGTGAACAACGACATCCTG

GGTACTTACTACTCATTCAAAGGTATCCCTTACGCTGCTCCTCCTCTGGGTGACCTGAGA

TTCAAAGCTCCTCAACCTCCTAAACCTTGGGAAGGTGTGAGATCAGCTAAAGAACACGGT

TCATCATCATACCAA TTCAACAACTTCCTGACTAAACTGGAAAAA
