# Supplementary material for: Genome Mining and Expression Analysis of Carboxylesterase and Glutathione S-Transferase Genes Involved in Insecticide Resistance in Eggplant Shoot and Fruit Borer, Leucinodes orbonalis (Lepidoptera: Crambidae)
Source: Front Physiol. 2020 Nov 19;11:594845. doi: 10.3389/fphys.2020.594845 (PMC7713791; doi:10.3389/fphys.2020.594845)
Supplement: Supplementary Table 5 — Detailed sequence information of GST from L. orbonalis. [file Table_5.DOCX]

| NCBI_ID | **Description** |
| --- | --- |
| NP_648237.1 | **glutathione S transferase O1 [Drosophila melanogaster]** |
| NP_649894.1 | **glutathione S transferase Z1 [Drosophila melanogaster]** |
| NP_725653.1 | **glutathione S transferase S1 [Drosophila melanogaster]** |
| NP_572886.2 | **glutathione S transferase T4 [Drosophila melanogaster]** |
| NP_611964.1 | **glutathione S transferase E12 [Drosophila melanogaster]** |
| NP_524326.1 | **glutathione S transferase D1 [Drosophila melanogaster]** |

Table 1: NCBI ID and description for the Reference protein taken in GST phylogeny.

Table 2: NCBI ID and description for the Reference protein taken in Carboxylesterases phylogeny

| **Cricklet** |  |
| --- | --- |
| NP_536784.1 | cricklet [Drosophila melanogaster] |
| NP_001303565 | uncharacterized protein Dmel_CG6414, isoform B [Drosophila  melanogaster] |
| **Alpha esterase** |  |
| NP_001246962.1 | alpha-Esterase-10, isoform C [Drosophila melanogaster] |
| NP_001262345.1 | alpha-Esterase-2, isoform B [Drosophila melanogaster] |
| NP_524269.3 | alpha-Esterase-1 [Drosophila melanogaster] |
| AAF54006.3 | alpha-Esterase-5, isoform A [Drosophila melanogaster] |
| NP_524261.1 | alpha-Esterase-7 [Drosophila melanogaster] |
| AAF54014.2 | alpha-Esterase-9, isoform C [Drosophila melanogaster] |
| NP_524259.2 | alpha-Esterase-8 [Drosophila melanogaster] |
| NP_524267.2 | alpha-Esterase-3, isoform A [Drosophila melanogaster] |
| NP_524262.1 | alpha-Esterase-6 [Drosophila melanogaster] |
| NP_524266.1 | alpha-Esterase-4 [Drosophila melanogaster] |
| **Gliotactin** |  |
| NP_723929.1 | gliotactin, isoform D [Drosophila melanogaster] |
| **Neurolignin** |  |
| NP_001261749.1 | esterase 6, isoform B [Drosophila melanogaster] |
| NP_788501.1 | esterase P [Drosophila melanogaster] |
| NP_001139209.1 | neuroligin 4 precursor [Apis mellifera] |
| NP_731170.2 | neuroligin 3, isoform C [Drosophila melanogaster] |
| AAF52450.2 | neuroligin 2, isoform A [Drosophila melanogaster] |
| **Juvenile Hormone esterase** |  |
| NP_001188759.1 | uncharacterized protein Dmel_CG3841, isoform B [Drosophila  melanogaster] |
| AAL41023.1 | juvenile hormone esterase [Tenebrio molitor] |
| NP_523758.3 | juvenile hormone esterase, isoform A [Drosophila melanogaster] |
| NP_611085.2 | juvenile hormone esterase duplication [Drosophila melanogaster] |
| **Glutactin** |  |
| NP_611881.1 | uncharacterized protein Dmel_CG10339 [Drosophila melanogaster] |
| NP_609244.1 | uncharacterized protein Dmel_CG9287 [Drosophila melanogaster] |
| NP_477504.1 | glutactin, isoform A [Drosophila melanogaster] |
| **Acetylcholine esterase** |  |
| AAF49416.1 | neurotactin, isoform A [Drosophila melanogaster] |
| NP_476953.1 | acetylcholine esterase, isoform A [Drosophila melanogaster] |
