# Supplementary material for: Genome Mining and Expression Analysis of Carboxylesterase and Glutathione S-Transferase Genes Involved in Insecticide Resistance in Eggplant Shoot and Fruit Borer, Leucinodes orbonalis (Lepidoptera: Crambidae)
Source: Front Physiol. 2020 Nov 19;11:594845. doi: 10.3389/fphys.2020.594845 (PMC7713791; doi:10.3389/fphys.2020.594845)
Supplement: Supplementary Presentation 1 — Graphical representation of the manuscript. [file Presentation_1.PPT]

## Slide 1
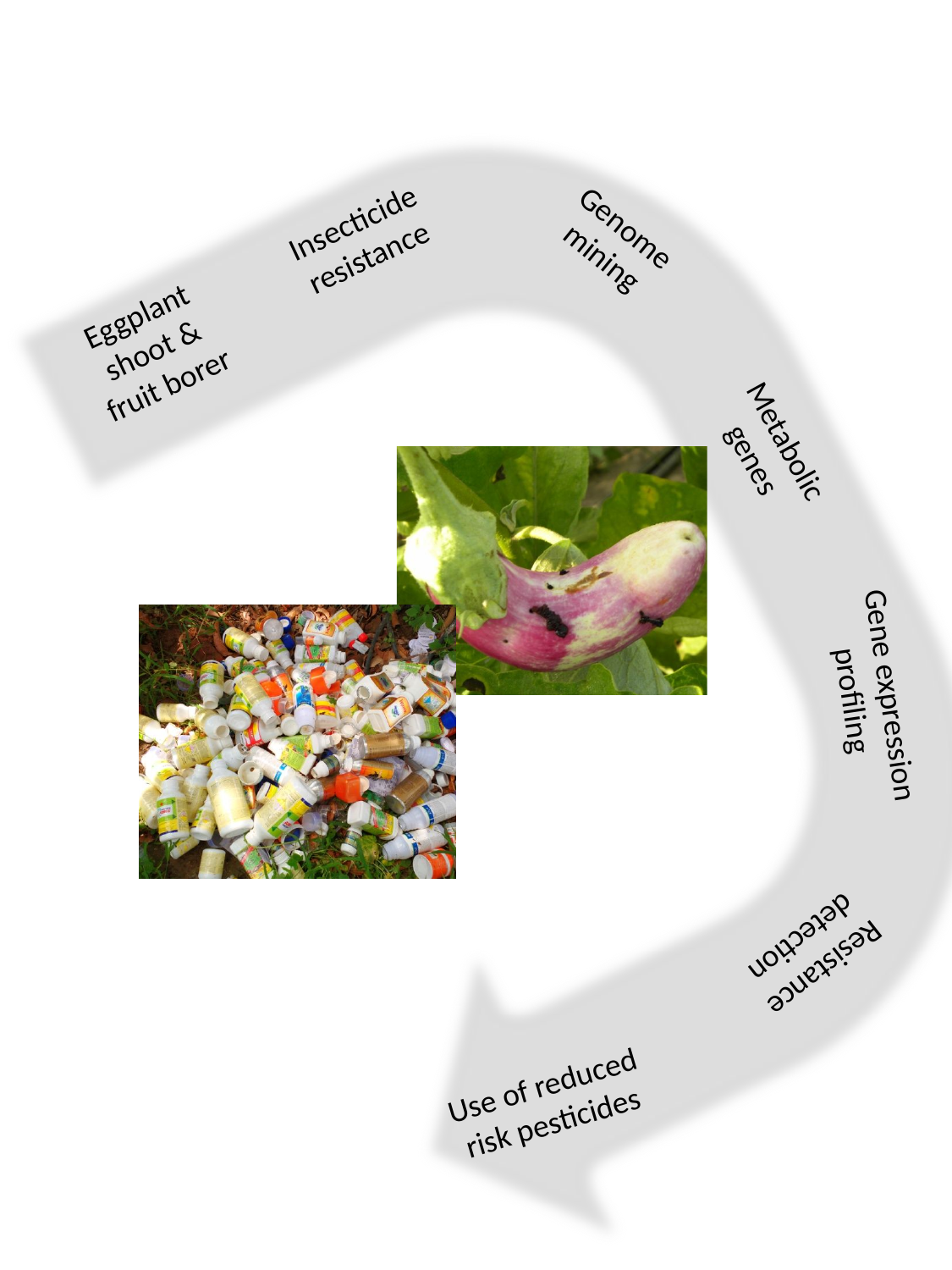

Insecticide resistance
Genome mining
Eggplant shoot & fruit borer
Metabolic genes
Gene expression profiling
Resistance detection
Use of reduced risk pesticides
